# A cross-sectional analysis of male versus female flourishing among 202,898 participants across 22 countries on 73 variables in the global flourishing study

**DOI:** 10.1038/s41598-026-40963-z

**Published:** 2026-02-22

**Authors:** Tim Lomas, R. Noah Padgett, Meg A. Warren, Byron R. Johnson, Tyler J. VanderWeele

**Affiliations:** 1 Department of Epidemiology, Harvard T.H. Chan School of Public Health, Boston, MA USA; 2 Human Flourishing Program, Harvard University, Cambridge, MA USA; 3 College of Business and Economics, Western Washington University, Bellingham, WA USA; 4 Institute for Studies of Religion, Baylor University, Waco, TX USA; 5 Chan School of Public Health, Department of Biostatistics, Boston, MA, USA

**Keywords:** Wellbeing, Flourishing, Sex, Gender, Male, Female, Psychology, Psychology, Sociology

## Abstract

A prominent debate in modern society is how males or females are faring relative to each other. Despite a vast literature, most research is constrained by two issues: a limited conceptualization and assessment of what it means to do well; and a relatively narrow, often Western-centric coverage. To address these issues, this paper examines male-female differences on 73 items relating to all aspects of wellbeing in the Global Flourishing Study, with data from 202,898 participants across 22 countries. When organizing the items into six domains according to VanderWeele’s flourishing framework, females do slightly better on three (happiness and satisfaction, social relationship quality, and meaning and purpose), and males on two (self-rated health, and financial and material security), and with character/virtue equal. While all domains are weighted equally, since females are only marginally higher on three, whereas the gaps on the two male-led ones are much bigger, males fare slightly better on an overall flourishing index. There is also considerable country-level variation throughout the findings however, showing these general trends are not universal but contingent on local socio-cultural dynamics.

**Supplementary Information:**

The online version contains supplementary material available at 10.1038/s41598-026-40963-z.

## Introduction

A perennial point of discussion in modern society is the question of how males and females are faring relative to each other. To explore this question, this paper leverages the first wave of data from the Global Flourishing Study (GFS), an intended five-year panel study investigating the predictors of human flourishing across more than 200,000 participants from 22 geographically and culturally diverse countries. Although some aspects of flourishing have received considerable attention vis-à-vis male-female differences—especially physical and mental health—other dimensions remain relatively under-studied and less-well understood. Hence the value of the GFS, which involves a 109-item questionnaire with over 70 items that cover myriad aspects of flourishing. In this Introduction, we review some relevant research and contextualize the GFS itself. First though, it is necessary to explain how we conceptualize and use key terms in this area, especially sex and gender.

### Sex and gender

Defining one’s central terms is, of course, good practice in any academic work, but is especially necessary in this topic, given that it has become among the most contentious areas of public discourse in recent years^1^. A large part of the debate involves contestation precisely around definitions of what it means to be male or female, and a man or woman, and who can or should be assigned these labels. It is beyond our scope to exhaustively cover this debate, and here we can merely clarify how *we* are using these terms, and how they have been used in the context of current data collection, while acknowledging that other people may have different definitions and perspectives. Given the international scope of the study, we draw on definitions from the WHO^2^ to ensure global relevance and applicability, with their position—as stated on their website at the time of publication—being that “It can be helpful to think of sex as a biological characteristic and gender as a social construct.” The WHO define sex as “a set of biological attributes in humans and animals … [that is] mainly associated with physical and physiological features including chromosomes, gene expression, hormone level and function, and reproductive and sexual anatomy … [and which] is often categorized as females and males, but there are variations of sex characteristics called intersex” (and for a more thorough discussion on sex, please see the Supplementary File). By contrast, gender is defined as “socially constructed characteristics of women and men – such as norms, roles and relations of and between groups of women and men.”

There is much one could say about these definitions and distinctions, which have become increasingly contested in some societies. While we cannot wade into these complexities here, we offer some reflections in the Supplementary File, and also below when we consider how sex and gender have been assessed in the context of the GFS. For now we just observe that, across the world, the sex categories of male and female usually lay the foundation for dividing people into girls and boys (in childhood) and subsequently women and men (in adulthood). This division then has ramifications ranging from norms around emotions and clothes, to practical and legal divisions in spheres of activity as diverse as the legal system and sports. As the WHO articulates it, gender norms and roles “are often upheld and reproduced in the values, legislation, education systems, religion, media and other institutions of the society in which they exist.” For a study of wellbeing, social constructions and norms thus play a critical role.

For instance, an important component of wellbeing is the experience and expression of positive and negative emotion. There is a widespread expectation across many cultures that females are, and moreover *should be*, more emotionally expressive, while conversely males are and ought to be emotionally “tougher” and more reserved^3^. Whether these expectations are accurate or helpful is another matter, but they are nevertheless examples of “socially constructed characteristics” widely assigned to females and males, often referred to as “femininity” and “masculinity” respectively. Of note though, gender norms and roles evolve and change. Even within a given society at a particular time, there can be competing gender ideals and practices, hence Connell’s^4^ influential work on masculinit*ies* (as opposed to a more monolithic masculinity). Nevertheless, Connell argued that certain norms/roles tend to become “hegemonic” (i.e., dominant), enforced through social processes such as conformity and ostracization^5^. With many societies upholding a hegemonic norm of masculine “toughness,” for example, a man who appears especially emotionally expressive might experience censure and bullying, perhaps compelling him to suppress his emotionality^6^.

A further complication when considering and discussing this topic is that, despite the WHO stating that sex and gender “are distinct and should not be used interchangeably,” they are indeed often treated as synonymous, with people frequently using “gender” when they are in fact referring to sex. This conflation is even evident in the work of Gallup, which is obviously especially pertinent to the present paper. When the Gallup World Poll (GWP) was established in 2005, this included recording participants’ sex, with the main response options being “male” and “female.” But the item itself has always been labelled and reported as being about “gender.” Moreover, while in face-to face interviews sex was assessed/judged by the interviewer, rather than asked directly, in phone interviews people were asked about their “gender.” This methodology is still in place, in part for longitudinal consistency, but also because of the need for applicability across numerous cultures where the sex/gender distinction may not be necessarily recognized by all survey respondents. Following the GWP, this approach has been incorporated into the GFS, as outlined in the Methods.

Two additional points are worth noting. First, Gallup themselves have recognized that using sex and gender interchangeably is increasingly regarded as problematic, especially in places like the USA, and have piloted new ways of asking about these topics^7–9^. Secondly, however, one must be wary about generalizing from debates and trends in countries that are relatively “WEIRD” (Western, Educated, Industrial, Rich, Democratic)^10^, notably the USA, to the rest of the world. Such caution is good practice generally, but is especially necessary when conducting global research. In surveys in the USA, it is increasingly common to ask participants how they self-identify regarding gender. This aligns with broader new cultural trends such as explicitly asking for people’s pronouns, as opposed to simply assuming pronouns based on people’s visually identifiable sex characteristics^11^. Yet even if the concepts and labels of sex and gender have become increasingly contested in the USA, this does not apply to all countries and languages. While sex and gender are distinct in English, this conceptual distinction is not necessarily found in other languages, and even when they *are* their usages are not necessarily problematic. Moreover, in some countries, it is actually not permissible, legally and/or culturally, to suggest or explicitly ask about sex/gender beyond two categories (e.g., male and female). As such, whether motivated by expressing cultural sensitivity, protecting research participants, or collecting useful data, it makes sense that the GWP still retains its original wording around sex/gender. Nevertheless, we still regret that because of the way the demographic item on sex is presented in the GFS questionnaire, it poses an inherent limitation in explicitly collecting data from those who do not identify with binary categories in some way, thereby limiting understanding of their flourishing and of the nuances around the gendered and sexed nature of flourishing more broadly.

### Sex differences in flourishing

Our primary interest in this paper, both in the literature and the GFS data, is in sex differences in flourishing, and so we will generally refer to “males” and “females” (rather than the now potentially more ambiguous and contested terms “men” and “women”). Sex differences are nevertheless intertwined with issues relating to gender. We briefly delve into some of these dynamics through the prism of mental health, and specifically depression and anxiety, known together as “common mental disorders” (CMDs). Although by no means the only aspects of flourishing, as elucidated below, they are among the outcomes that have received the most attention vis-à-vis sex differences. Overall, females are generally regarded as faring worse. Regarding depression, the WHO^12^ estimates this is experienced by approximately 5% of adults globally, but with figures of 4% for males and 6% for females. The WHO^13^ likewise report that anxiety, experienced by around 4% of the population, is higher among females, and although the WHO don’t provide specific sex estimates, organizations like the National Institutes of Health suggest that females are around 1.6 times more likely to be diagnosed with anxiety disorders^14^.

One must note that these broad trends are complicated by various demographic factors, including race/ethnicity^15^, age^16^, and socio-economic status^17^. Males appear to be more affected by poverty than females, for instance: the EHRC^18^ estimated that males in the poorest quintile of the population are almost three times more likely to have a CMD than those in the richest, while for females the ratio is only two to one. The intersectionality paradigm^19^ therefore cautions against generalizing by sex alone, as variation among males and females is produced by the way sex and gender intersect with the myriad other demographic and identity categories^20^. Intersectionality notwithstanding though, higher rates of CMDs in females, particularly depression, is considered “one of the most widely documented findings in psychiatric epidemiology”^21^.

The picture is further complicated though by sex and gender differences in the way that people *experience* and *report* conditions like depression, possibly leading to their underdiagnoses in males. A standard symptom of depression is rumination, for instance, and one conventional explanation for higher rates of depression in females is that they have a greater tendency towards this cognitive style, whether due to genetic factors, gendered socialization, or some combination of both^22^. However, scholars have raised concerns that the generic depression diagnostic criteria reflect the kind of “internalizing” responses seemingly favoured by and/or encouraged in females, and that males may experience and express distress and depression in other ways. Above we noted that hegemonic masculinity norms tend to pressure males into trying to be tough and stoic, which can often lead to emotional disconnection or suppression^6^. As a result, males are thought to be more likely to “externalize” their distress, especially though destructive behaviours, both towards the self and others^23^. Consider that the suicide rate among males is estimated by the CDC^24^ to be almost four times higher than among females (23.0 versus 5.9 per 100,000 people), that males are nearly three times more likely to die from alcohol^25^, and that FBI statistics show that males commit 77% of violent crime (with females at 18% and 5% unknown)^26^. Scholars therefore suggest this pattern of responding may be a form of depression that is more common among males but which is currently being overlooked by standard diagnostic criteria^27^. More generally, the key point is that apparent sex differences in wellbeing can be complicated, given that gender-related factors may not only influence prevalence rates of various issues but even the way such issues might be expressed, self-reported, and assessed. As ever, more research is needed to better understand these sex- and gender-related dynamics of mental health.

While sex differences in physical and mental health have received considerable attention, other aspects of flourishing are far less well understood. There are many ways of conceptualizing flourishing, but the framework we will draw upon is that of VanderWeele^28^, who defines flourishing expansively as “the relative attainment of a state in which all aspects of a person’s life are good, including the contexts in which that person lives.” Specifically, he identifies five key domains of flourishing: happiness and life satisfaction; mental and physical health; meaning and purpose; character and virtue; and close social relationships. A sixth domain of financial and material stability is added as an important means for “secure flourishing” over time. These domains are not exhaustive of flourishing, and others may also be important; indeed, the GFS questionnaire includes several other dimensions, such as religion and spirituality^29^. Nevertheless, VanderWeele argues that whatever one’s view, all six in the framework are “arguably at least a part of what we mean by flourishing.” To that end, VanderWeele has also presented a 12-item Secure Flourish Index^28^, with two items per dimensions—with subsequent evidence supporting its psychometrics^30–32^—which serves as a foundation for the GFS questionnaire. From this perspective, mental health is only one aspect of the second domain (alongside physical health), with five other domains also constituting the multidimensional portrait of flourishing.

When it comes to understanding sex differences though, these other five domains have received far less attention than mental and physical health. There is nevertheless some relevant research into the other domains, with perhaps the most attention being paid to the first domain of happiness and life satisfaction, which together approximate to the concept, developed by Diener^33 and others, of^ “subjective wellbeing” (SWB). Overall, the picture is somewhat unclear, with one review^34^ noting that reports are “inconsistent and conflicting,” with males faring better in some studies and females in others. One can even find mixed reports in the same study: in an analysis of 31 items pertaining to wellbeing in the GWP, although males fared better than females on 21 variables, including positive emotion, females scored higher on life evaluation, possibly because they do better on variables that matter more for that outcome, above all social relationships^35^. A similarly mixed picture was provided by Kahneman and Deaton^36^, also using GWP data, with females expressing greater life satisfaction and positive affect than males, but *also* greater stress and negative affect, implying that females may experience more intense emotions per se, good and bad, so in a sense are happier *and* unhappier. That said, we should also recall the point above that males do have the potential to be strongly emotional, but this is sometimes suppressed through gendered socialization processes, such as masculinity norms of toughness and stoicism^6^. Relatedly, similar gender norms may mean that even if males *do* experience strong emotions, they may also face pressure to conceal these from other people (i.e., to *appear* tough even if they do not actually feel tough), which could include not self-reporting high levels of emotionality in surveys designed to assess it^37^. While these considerations do not invalidate sex-based analyses of self-report scales, they do mean we must not necessarily take the data at face value but allow for the possibility that the picture may be more complex.

A related issue, and one that may also contribute to the “inconsistent and conflicting” results noted above^34,^ is the influence of socio-cultural dynamics. As noted above, sex differences in flourishing are intimately intertwined with gender considerations, and gender itself is heavily shaped by socio-cultural processes. How males and females are treated in various domains of life can vary considerably depending on the values and traditions in a given environment. Some trends are more universal, especially if linked to biological considerations. That females not males give birth, for instance, has implications that hold relatively true across cultures, such as in relation to employment, where child-bearing females are compelled to experience at least some disruption to their working lives, whereas males do not face nearly the same (or sometimes even any) kind of disruption in relation to having children. In other areas or aspects of life though, the cultural dynamics can be more variable, which can affect the relative SWB of the sexes. One study^38^ for example investigated “the social and cultural conditions that favor higher female relative to male happiness and life satisfaction” using data from more than 90 countries in the World Values Survey. It found various “conditions associated with a high level of female relative to male happiness and life satisfaction,” such as an “absence of communist history,” and—among indicators of gender equality—“a low rate of female non-agricultural employment.”

Such analyses and insights are valuable but also relatively rare, and call for more interdisciplinary research on the underlying mechanisms. It has become widely recognized that a majority of research in fields like psychology has been conducted by and on people in societies described—as introduced above—as relatively “WEIRD”^10^, from where most research in top journals continues to originate^39^. These biases apply to flourishing specifically^40^, even if this trend may be shifting as scholars increasingly appreciate the need to conduct more cross-cultural and global research^41^. A potent new instance of such research is the GFS, a five-year (minimum) panel study investigating the predictors of human flourishing across more than 200,000 participants from 22 geographically and culturally diverse countries. It involves a 109-item questionnaire^29^, which includes a comprehensive battery of items relating to all aspects of flourishing, together with a detailed demographic intake form. The present paper utilizes this unique dataset to explore sex differences across myriad aspects of flourishing.

## Methods

The description of the methods below has been adapted from VanderWeele and colleagues^42^. Further methodological detail is available elsewhere, including: an overview of the GFS as a whole^43^ and its general methodology^44^; an initial questionnaire development report^45^, as well as an updated account of the questionnaire development process^29^, of which one aspect was a process of piloting the items through cognitive interviewing,^46^; the Wave 1 codebook^47^; the survey sampling design for Wave 1^48^; the statistical analyses code^49^; and the methodology for demographic variation analyses for wave 1^50^. A major aim of the current paper is to describe the potential differences in flourishing between males and females without conditioning on control variables (e.g., age, employment, etc.). This allows for an initial descriptive investigation that aligns with the GFS’s existing methodological approach, which can then lead to more fine-grained conditional future explorations.

### Data

The GFS is a study involving over 200,000 participants (in Wave 1) from 22 geographically and culturally diverse countries: Argentina, Australia, Brazil, China (including the mainland, and also separately Hong Kong, a Special Administrative Region, meaning the GFS actually has *23* distinct populations), Egypt, Germany, India, Indonesia, Israel, Japan, Kenya, Mexico, Nigeria, the Philippines, Poland, South Africa, Spain, Sweden, Tanzania, Turkey, the United Kingdom, and the United States. The countries were selected to (1) maximize coverage of the world’s population, (2) ensure geographic, cultural, and religious diversity, and (3) prioritize feasibility in Gallup’s existing data collection infrastructure. Data for Wave 1 were collected from March 2022 to January 2024, except in mainland China (March/April of 2024). The present paper was written before the data for mainland China were available, so does not include that population—though it *does* include Hong Kong—and hence only includes data for 202,898 participants. The precise sampling design to ensure nationally representative samples varied by country and further details are available^44^. Survey items included aspects of flourishing such as SWB, health, meaning, character, relationships, and financial stability^28^, plus other demographic, social, economic, political, religious, personality, childhood, community, health, and wellbeing variables. These data are publicly available through the Center for Open Science (https://www.cos.io/gfs-access-data). During the translation process, Gallup adhered to the TRAPD model (translation, review, adjudication, pretesting, and documentation) for cross-cultural survey research; for additional details, see the questionnaire development process report^29^.

### Measures

#### Outcome variables

There are 73 specific outcomes, which can be organized into various dimensions of flourishing, elucidated below. Of these, most currently have an associated preregistration from one of the core GFS team (as indicated by the hyperlink below). These outcomes have been used as part of a coordinated set of global analyses across all 22 GFS countries, and many already have been published as peer reviewed papers.

#### Variables for demographic variation analyses

There are eight primary demographic variables: gender (sex); age marital status; employment; education; religious service attendance; race/ethnicity; education; and immigration status. Most relevantly here, the “gender” item is intended to record participants’ sex, though is referred to in the questionnaire and subsequent analyses as “gender,” as discussed above. The manner of assessing this varied. In face-to face interviews, interviewers recorded people’s sex (“gender”) without asking, with three options: male; female; and don’t know/unsure. In telephone interviews, after saying “I am required to ask this for quality assurance purposes,” interviewers asked, “Can you please tell me your gender?,” and left an open space for the participant to answer. After the person provided their answer, the interviewer coded this according to five options: male; female; other; don’t know; refused to answer. Finally, in web versions of the questionnaire, the question appeared as follows: “What is your gender?,” with the response options: male; female; other; prefer not to answer.

Continuous age was classified as 18–24, 25–29, 30–39, 40–49, 50–59, 60–69, 70–79, and 80 or older. Marital status was assessed as single/never married, married, separated, divorced, widowed, and domestic partner. Employment was assessed as employed, self-employed, retired, student, homemaker, unemployed and searching, and other. Education was assessed as up to 8 years, 9–15 years, and 16+ years. Religious service attendance was assessed as more than once/week, once/week, one-to-three times/month, a few times/year, or never. Immigration status was dichotomously assessed with: “Were you born in this country, or not?” Religious tradition/affiliation with categories of Christianity, Islam, Hinduism, Buddhism, Judaism, Sikhism, Baha’i, Jainism, Shinto, Taoism, Confucianism, Primal/Animist/Folk religion, Spiritism, African-Derived, some other religion, or no religion/atheist/agnostic; precise response categories varied by country^43^. Racial/ethnic identity was assessed in some, but not all, countries, with response categories varying by country. Immigration status was assessed with: “Were you born in this country, or not?” For additional details on the assessments see the COS GFS codebook^47^.

### Analyses

#### Outcome-wide analytic design

An outcome-wide analytic approach^51^ was employed to examine the associations of a single variable (gender) with a range of outcomes. Compared to traditional analytic strategies focused on a single outcome, this approach provides a more holistic assessment of a focal variable’s possibly differential relations to multiple outcomes. This design has strengths of reducing researcher subjectivity and degree-of-freedom in the analysis^52^ by ensuring a consistent analytic strategy across models for all outcomes, mitigating publication bias by reporting results for all examined outcomes simultaneously, and providing insights into positive, negative, and null associations with our focal variable of gender.

#### Statistical models

Analyses were aligned with those conducted globally on each outcome (see linked pre-registrations in the Supplementary File). Summary statistics of the observed data were estimated using the weighted sample with the full sample simultaneously—not separately by country. Next, analyses were conducted separately by country to estimate the overall mean on each outcome and the subgroup means across demographic characteristics. All of these country-specific analyses incorporated the within country complex sampling design to obtain adjusted standard errors. A global p-value from a significance test of differences in means or proportions across demographic categories is provided, and the reported p-values are a Wald-type tests for complex surveys^53^. These country-specific results are available in our online Supplemental Material. Primary results consist of a random effects meta-analysis of country-specific means/proportions by sex categories^54,55^ along with 95% confidence intervals of the meta-analysed means and proportions. Using random-effects meta-analyses to pool results across countries limits the influence any one country’s estimate has on the overall mean within sex category, so that countries with larger sample size are not more influential in the comparison. The full set of results for all outcomes described previously will be reported in at least the online supplement with focal results presented in text.

#### Inference criteria

We present exact p-values (not a crude > 0.05 or < 0.05), and 95% confidence intervals. P-values correspond to 2-tailed tests for each of our analyses. In our tables, and for ease of reviewing results, we present multiple p-value cutoffs (both with and without Bonferroni correction for multiple testing), because different investigators often use different threshold standards for interpreting evidence based on current norms in their specific discipline. Our Bonferroni correction is 0.05/73=0.00069.

#### Missing data and multiple imputation

All missing variables are imputed using multivariate imputation by chained equations, with five imputed datasets generated^56,57^. The imputation model incorporated the criterion/outcome variable, all demographic characteristics, including race/ethnicity and religious affiliation when available, and sampling weights. The sampling weights were included as a variable in the imputation models to allow for specific variable missingness to be related to probability of study inclusion. To account for variations in the assessment of certain variables across countries (e.g., race/ethnicity and religious affiliation), we conducted the imputation process separately for each country. The within-country imputation approach ensured that the imputation model accurately reflects country-specific contexts and assessment methods.

#### Accounting for complex sampling design

The GFS used different sampling schemes across countries based on availability of existing panels and recruitment needs^44^. All analyses accounted for the complex survey design components by including weights, primary sampling units, and strata. Additional methodological detail, including accounting for the complex sampling design is provided elsewhere^48^.

#### Ethics

This project was ruled exempt by the Baylor University Institutional Review Board (#1841317-2). Gallup Inc. IRB approved the study on November 16, 2021 (#2021-11-02). All data collection was performed in accordance with the ethical standards of Gallup and with the 1964 Helsinki Declaration and its later amendments. Informed consent is obtained during the respondent recruitment stage of fieldwork, at the start of the survey. The exact wording varies across countries depending on the local laws and regulations governing data protection. Subsequent surveys include a consent statement that reminds respondents that participation in the survey is optional and their personal information will not be shared by anyone outside of Gallup.

## Results

The results consist of four main Tables: (1) descriptive statistics of the complete sample; (2) outcome summary statistics for the complete sample; (3) outcome-wide results of demographic variation for gender; and (4) a summary of the outcome-wide results, both across the sample as a whole and in the 22 individual countries. Details of the outcome-wide results of demographic variation for gender for each individual country are included as Supplementary Files.

**Table 1:** Descriptive statistics of the complete sample.

Characteristic	*N* = 202,898^1^
Age group	
18–24	27,007 (13%)
25–29	20,700 (10%)
30–39	40,256 (20%)
40–49	34,464 (17%)
50–59	31,793 (16%)
60–69	27,763 (14%)
70–79	16,776 (8.3%)
80 or older	4,119 (2.0%)
(Missing)	20 (< 0.1%)
**Gender**	
Male	98,411 (49%)
Female	103,488 (51%)
Other	602 (0.3%)
(Missing)	397 (0.2%)
**Marital status**	
Married	107,354 (53%)
Separated	5,195 (2.6%)
Divorced	11,654 (5.7%)
Widowed	9,823 (4.8%)
Single, never married	52,115 (26%)
Domestic Partner	14,931 (7.4%)
(Missing)	1,826 (0.9%)
**Employment**	
Employed for an employer	78,815 (39%)
Self-employed	36,362 (18%)
Retired	29,303 (14%)
Student	10,726 (5.3%)
Homemaker	21,677 (11%)
Unemployed and looking for a job	16,790 (8.3%)
None of these/Other	8,431 (4.2%)
(Missing)	793 (0.4%)
**Religious service attendance**	
More than 1/week	26,537 (13%)
1/week	39,157 (19%)
1–3/month	19,749 (9.7%)
A few times a year	41,436 (20%)
Never	75,297 (37%)
(Missing)	722 (0.4%)
**Education**	
Up to 8 years	45,078 (22%)
9–15 years	115,097 (57%)
16 + years	42,578 (21%)
(Missing)	146 (< 0.1%)
**Immigration**	
Born in this country	190,998 (94%)
Born in another country	9,791 (4.8%)
(Missing)	2,110 (1.0%)
**Country**	
Argentina	6,724 (3.3%)
Australia	3,844 (1.9%)
Brazil	13,204 (6.5%)
Egypt	4,729 (2.3%)
Germany	9,506 (4.7%)
Hong Kong	3,012 (1.5%)
India	12,765 (6.3%)
Indonesia	6,992 (3.4%)
Israel	3,669 (1.8%)
Japan	20,543 (10%)
Kenya	11,389 (5.6%)
Mexico	5,776 (2.8%)
Nigeria	6,827 (3.4%)
Philippines	5,292 (2.6%)
Poland	10,389 (5.1%)
South Africa	2,651 (1.3%)
Spain	6,290 (3.1%)
Sweden	15,068 (7.4%)
Tanzania	9,075 (4.5%)
Turkey	1,473 (0.7%)
United Kingdom	5,368 (2.6%)
United States	38,312 (19%)

Note. This table is based on non-imputed data. Cumulative percentages for variables may not add up to 100% due to rounding. ^1^n (%);.

**Table 2:** Outcome summary statistics for the complete sample.

Characteristic	*N* = 202,898
**Flourish Index (10 items)**	7.31 (1.72)^1^
(Missing)	5,058 (2.5%)^2^
**Secure Flourish Index (12 items)**	7.07 (1.68)
(Missing)	5,622 (2.8%)
**Happiness & Life Satisfaction**	6.91 (2.26)
(Missing)	1,025 (0.5%)
**Social Relationship Quality**	7.40 (2.36)
(Missing)	1,465 (0.7%)
**Meaning and Purpose**	7.36 (2.21)
(Missing)	1,400 (0.7%)
**Character & Virtue**	7.59 (1.96)
(Missing)	1,445 (0.7%)
**Self-Rated Health**	7.31 (2.12)
(Missing)	679 (0.3%)
**Financial and Material Worry**	5.8 (3.2)
(Missing)	768 (0.4%)
**Happiness**	6.99 (2.38)
(Missing)	399 (0.2%)
**Life Satisfaction**	6.83 (2.60)
(Missing)	694 (0.3%)
**Present Life Evaluation**	6.42 (2.46)
(Missing)	516 (0.3%)
**Future Life Evaluation**	7.49 (2.30)
(Missing)	4,182 (2.1%)
**Optimism**	8.00 (2.32)
(Missing)	795 (0.4%)
**Freedom**	7.67 (2.45)
(Missing)	486 (0.2%)
**Peace**	
Always	44,831 (22%)
Often	100,921 (50%)
Rarely	48,417 (24%)
Never	7,958 (3.9%)
(Missing)	771 (0.4%)
**Balance in life**	
Always	34,422 (17%)
Often	105,791 (52%)
Rarely	53,603 (26%)
Never	8,162 (4.0%)
(Missing)	919 (0.5%)
**Mastery**	
Always	53,297 (26%)
Often	101,787 (50%)
Rarely	39,959 (20%)
Never	6,736 (3.3%)
(Missing)	1,118 (0.6%)
**Meaning**	7.30 (2.47)
(Missing)	625 (0.3%)
**Purpose**	7.41 (2.59)
(Missing)	838 (0.4%)
**Self-Rated Mental Health**	7.55 (2.42)
(Missing)	392 (0.2%)
**Content with My Relationships**	7.56 (2.48)
(Missing)	707 (0.3%)
**Satisfying Relationships**	7.24 (2.59)
(Missing)	891 (0.4%)
**Social Support**	7.39 (2.84)
(Missing)	557 (0.3%)
**Intimate friend**	
Yes	166,774 (82%)
No	35,189 (17%)
(Missing)	935 (0.5%)
**Government approval**	
Strongly approve	29,056 (14%)
Somewhat approve	46,802 (23%)
Neither approve nor disapprove	43,017 (21%)
Somewhat disapprove	35,702 (18%)
Strongly disapprove	42,300 (21%)
(Missing)	6,021 (3.0%)
**Political voice**	
Agree	71,668 (35%)
Disagree	80,182 (40%)
Unsure	50,137 (25%)
(Missing)	911 (0.4%)
**Belonging**	7.59 (2.62)
(Missing)	1,303 (0.6%)
**City satisfaction**	
Satisfied	151,321 (75%)
Dissatisfied	31,758 (16%)
Unsure	18,919 (9.3%)
(Missing)	900 (0.4%)
**Trust**	
All	6,162 (3.0%)
Most	39,489 (19%)
Some	83,777 (41%)
Not very many	61,197 (30%)
None	10,489 (5.2%)
(Missing)	1,785 (0.9%)
**Community participation**	
More than once a week	15,717 (7.7%)
Once a week	20,419 (10%)
One to three times a month	23,985 (12%)
A few times a year	45,699 (23%)
Never	96,419 (48%)
(Missing)	661 (0.3%)
**Traumatic distress**	
A lot	23,026 (11%)
Some	45,950 (23%)
Not very much	60,230 (30%)
None at all	72,840 (36%)
(Missing)	853 (0.4%)
**Suffering**	
A lot	21,244 (10%)
Some	66,329 (33%)
Not very much	67,197 (33%)
None at all	47,332 (23%)
(Missing)	796 (0.4%)
**Loneliness**	3.4 (3.1)
(Missing)	387 (0.2%)
**Discrimination**	
Always	12,958 (6.4%)
Often	32,247 (16%)
Rarely	78,243 (39%)
Never	78,507 (39%)
(Missing)	943 (0.5%)
**Promoting Good**	7.88 (2.10)
(Missing)	691 (0.3%)
**Delayed Gratification**	7.29 (2.47)
(Missing)	928 (0.5%)
**Hope**	7.93 (2.30)
(Missing)	2,668 (1.3%)
**Gratitude**	7.77 (2.43)
(Missing)	2,982 (1.5%)
**Love**	8.12 (2.25)
(Missing)	374 (0.2%)
**Forgiveness**	
Always	60,695 (30%)
Often	91,174 (45%)
Rarely	41,204 (20%)
Never	9,117 (4.5%)
(Missing)	708 (0.3%)
**Charitable giving**	
Yes	75,510 (37%)
No	126,827 (63%)
(Missing)	560 (0.3%)
**Helping**	
Yes	104,837 (52%)
No	97,094 (48%)
(Missing)	967 (0.5%)
**Volunteering**	
Yes	45,914 (23%)
No	156,368 (77%)
(Missing)	616 (0.3%)
**Self-Rated Physical Health**	7.06 (2.38)
(Missing)	350 (0.2%)
**Health limitations**	
Yes	43,267 (21%)
No	158,145 (78%)
(Missing)	1,486 (0.7%)
**Pain**	
A lot	24,960 (12%)
Some	65,692 (32%)
Not very much	64,273 (32%)
None at all	47,597 (23%)
(Missing)	377 (0.2%)
**Smoking**	2.0 (5.7)
(Missing)	3,296 (1.6%)
**Drinking**	2.1 (5.6)
(Missing)	2,932 (1.4%)
**Exercise**	
0 days	68,118 (34%)
1 day	22,472 (11%)
2 days	24,432 (12%)
3 days	24,414 (12%)
4 days	13,471 (6.6%)
5 days	12,947 (6.4%)
6 days	6,081 (3.0%)
7 days/Every day	27,503 (14%)
(Missing)	3,459 (1.7%)
**Financial Stability**	5.7 (3.4)
(Missing)	351 (0.2%)
**Material Stability**	6.0 (3.4)
(Missing)	484 (0.2%)
**Education**	
Up to 8 years	45,078 (22%)
9–15 years	115,097 (57%)
16 + years	42,578 (21%)
(Missing)	146 (< 0.1%)
**Employment**	
Employed for an employer	78,815 (39%)
Self-employed	36,362 (18%)
Retired	29,303 (14%)
Student	10,726 (5.3%)
Homemaker	21,677 (11%)
Unemployed and looking for a job	16,790 (8.3%)
None of these/Other	8,431 (4.2%)
(Missing)	793 (0.4%)
**Subjective financial well-being**	
Living comfortably on present income	49,702 (24%)
Getting by on present income	86,158 (42%)
Finding it difficult on present income	43,114 (21%)
Finding it very difficult on present income	21,670 (11%)
(Missing)	2,254 (1.1%)
**Housing**	
Someone in this household OWNS this home	114,023 (56%)
Someone in this household RENTS this home	41,636 (21%)
Both	6,516 (3.2%)
Neither	23,679 (12%)
Rent	4,224 (2.1%)
Own	10,600 (5.2%)
Something else	0 (0%)
(Missing)	2,221 (1.1%)
**Self-reported religion/spirituality**	
Always	64,434 (32%)
Often	52,203 (26%)
Rarely	50,914 (25%)
Never	34,758 (17%)
(Missing)	589 (0.3%)
**Religious service attendance**	
More than once a week	26,537 (13%)
Once a week	39,157 (19%)
One to three times a month	19,749 (9.7%)
A few times a year	41,436 (20%)
Never	75,297 (37%)
(Missing)	722 (0.4%)
**Life after death belief**	
Yes	103,185 (51%)
No	48,064 (24%)
Unsure	50,611 (25%)
(Missing)	1,038 (0.5%)
**Religious experience**	
Yes	74,166 (37%)
No	127,201 (63%)
(Missing)	1,531 (0.8%)
**Religious reading**	
More than once a day	22,822 (11%)
About once a day	31,784 (16%)
Sometimes	70,888 (35%)
Never	76,325 (38%)
(Missing)	1,077 (0.5%)
**Prayer-meditation**	
More than once a day	49,670 (24%)
About once a day	41,659 (21%)
Sometimes	57,806 (28%)
Never	53,072 (26%)
(Missing)	690 (0.3%)
**Belief in god**	
One God	119,461 (59%)
More than one god	10,985 (5.4%)
An impersonal spiritual force	17,793 (8.8%)
None of these	32,491 (16%)
Unsure	21,472 (11%)
(Missing)	695 (0.3%)
**Intrinsic religiosity**	
Agree	100,163 (49%)
Disagree	31,808 (16%)
Not relevant	47,873 (24%)
Unsure	21,957 (11%)
(Missing)	1,097 (0.5%)
**Religious comfort**	
Agree	115,788 (57%)
Disagree	25,075 (12%)
Not relevant	43,603 (21%)
Unsure	17,585 (8.7%)
(Missing)	847 (0.4%)
**Loved by god**	
Agree	118,740 (59%)
Disagree	22,520 (11%)
Not relevant	41,656 (21%)
Unsure	18,885 (9.3%)
(Missing)	1,097 (0.5%)
**Spiritual punishment**	
Agree	41,504 (20%)
Disagree	92,150 (45%)
Not relevant	44,322 (22%)
Unsure	23,723 (12%)
(Missing)	1,199 (0.6%)
**Religious criticism**	
Agree	38,858 (19%)
Disagree	74,494 (37%)
Not relevant	63,755 (31%)
Unsure	24,598 (12%)
(Missing)	1,194 (0.6%)
**Faith-sharing**	
Agree	85,708 (42%)
Disagree	49,112 (24%)
Not relevant	52,311 (26%)
Unsure	14,815 (7.3%)
(Missing)	952 (0.5%)
**Children**	1.03 (1.63)
(Missing)	1,418 (0.7%)

Note. This table is based on non-imputed data. Cumulative percentages for variables may not add up to 100% due to rounding. Means and standard deviations are based on complex survey adjusted (weighted) estimates of the observed cases only. ^1^weighted mean (SD), ^2^n (%);.

**Table 3:** Outcome-wide results of demographic variation for gender.

Outcome	Male	Female	Other	MFO Global *p*-value^1^	MF Global *p*-value^2^
Flourish index and domains					
Flourish Index	7.45 (7.20,7.69)	7.43 (7.20,7.66)	6.32 (5.83,6.82)	< 0.00001	0.00002
Secure Flourish Index	7.19 (6.99,7.40)	7.12 (6.92,7.32)	6.09 (5.69,6.50)	< 0.00001	0.00001
Happiness & Life Satisfaction^a^	6.90 (6.63,7.18)	6.96 (6.72,7.20)	5.92 (5.45,6.39)	< 0.00001	0.00002
Social Relationship Quality^b^	7.53 (7.28,7.79)	7.56 (7.33,7.80)	6.52 (6.12,6.91)	< 0.00001	0.00001
Meaning and Purpose^c^	7.50 (7.22,7.78)	7.55 (7.27,7.82)	6.15 (5.43,6.87)	< 0.00001	0.00005
Character & Virtue^d^	7.73 (7.46,7.99)	7.73 (7.46,8.00)	7.01 (6.43,7.59)	< 0.00001	0.02917
Self-Rated Health^e^	7.58 (7.28,7.87)	7.35 (7.05,7.66)	6.00 (5.36,6.65)	< 0.00001	< 0.00001
Financial and Material Worry^f^	5.93 (5.51,6.34)	5.56 (5.07,6.06)	4.94 (4.27,5.61)	< 0.00001	< 0.00001
Psychological well-being					
Happiness^a^	6.98 (6.73,7.24)	7.03 (6.80,7.26)	5.94 (5.45,6.44)	< 0.00001	0.00002
Life Satisfaction^a^	6.82 (6.49,7.16)	6.89 (6.60,7.18)	5.93 (5.48,6.38)	< 0.00001	0.00001
Present Life Evaluation	6.31 (5.95,6.67)	6.38 (6.07,6.69)	5.98 (5.72,6.23)	< 0.00001	0.00002
Future Life Evaluation	7.43 (7.15,7.72)	7.54 (7.27,7.82)	7.35 (6.80,7.91)	< 0.00001	0.00002
Optimism	8.04 (7.72,8.37)	8.19 (7.87,8.51)	6.88 (5.85,7.91)	< 0.00001	0.00002
Freedom	7.81 (7.54,8.09)	7.80 (7.56,8.04)	6.73 (6.00,7.45)	< 0.00001	0.00002
Peace	0.74 (0.69,0.78)	0.72 (0.67,0.77)	0.75 (0.29,0.96)	< 0.00001	< 0.00001
Balance in Life	0.71 (0.66,0.75)	0.69 (0.64,0.73)	0.63 (0.16,0.93)	< 0.00001	0.00001
Mastery	0.82 (0.77,0.85)	0.79 (0.75,0.83)	0.83 (0.40,0.97)	< 0.00001	0.00001
Meaning^c^	7.34 (7.06,7.62)	7.45 (7.18,7.71)	6.60 (6.03,7.18)	< 0.00001	0.00002
Purpose^c^	7.66 (7.33,7.99)	7.65 (7.32,7.98)	5.71 (4.80,6.61)	< 0.00001	0.09693
Self-Rated Mental Health^e^	7.81 (7.48,8.14)	7.62 (7.26,7.97)	5.75 (4.85,6.65)	< 0.00001	< 0.00001
Traumatic Distress (n)	0.34 (0.29,0.38)	0.38 (0.33,0.42)	0.26 (0.05,0.68)	< 0.00001	0.00001
Depression Symptoms (n)	0.29 (0.25,0.35)	0.32 (0.27,0.37)	0.53 (0.22,0.81)	< 0.00001	0.00002
Anxiety Symptoms (n)	0.26 (0.22,0.31)	0.31 (0.26,0.36)	0.38 (0.07,0.83)	< 0.00001	0.00001
Suffering (n)	0.41 (0.37,0.46)	0.46 (0.41,0.51)	0.44 (0.12,0.82)	< 0.00001	< 0.00001
Social well-being					
Content with my Relationships^b^	7.69 (7.43,7.94)	7.73 (7.50,7.97)	6.66 (6.25,7.06)	< 0.00001	0.00001
Satisfying Relationships^b^	7.38 (7.12,7.64)	7.39 (7.15,7.64)	6.46 (6.01,6.90)	< 0.00001	0.00001
Social Support	7.29 (6.90,7.69)	7.50 (7.14,7.87)	6.98 (6.39,7.57)	< 0.00001	< 0.00001
Intimate Friend	0.82 (0.80,0.85)	0.85 (0.83,0.87)	0.94 (0.77,0.98)	< 0.00001	0.00001
Government Approval	0.44 (0.35,0.53)	0.41 (0.32,0.52)	0.15 (0.03,0.52)	< 0.00001	0.00001
Political Voice	0.53 (0.43,0.62)	0.52 (0.41,0.62)	0.63 (0.16,0.94)	< 0.00001	0.00002
Belonging	7.71 (7.42,8.01)	7.77 (7.48,8.06)	5.86 (4.92,6.81)	< 0.00001	0.00002
City Satisfaction	0.83 (0.79,0.87)	0.85 (0.81,0.89)	0.76 (0.28,0.96)	< 0.00001	0.00158
Trust	0.25 (0.20,0.30)	0.21 (0.16,0.27)	0.02 (0.00,0.11)	< 0.00001	0.00001
Community Participation	0.21 (0.18,0.25)	0.17 (0.14,0.20)	0.07 (0.01,0.36)	< 0.00001	< 0.00001
Loneliness (n)	3.31 (3.08,3.54)	3.43 (3.21,3.65)	4.60 (4.05,5.14)	< 0.00001	0.00178
Discrimination (n)	0.24 (0.20,0.28)	0.22 (0.19,0.26)	0.49 (0.14,0.86)	< 0.00001	0.06092
*Character & Prosocial Behavior*					
Promoting Good^d^	7.99 (7.74,8.23)	8.03 (7.79,8.27)	7.41 (6.91,7.91)	< 0.00001	0.00359
Delayed Gratification^d^	7.47 (7.16,7.77)	7.43 (7.11,7.75)	6.63 (5.94,7.32)	< 0.00001	0.00002
Hope	8.11 (7.79,8.43)	8.15 (7.83,8.47)	6.76 (5.91,7.61)	< 0.00001	0.34088
Gratitude	7.72 (7.41,8.03)	7.96 (7.67,8.25)	7.03 (6.24,7.81)	< 0.00001	< 0.00001
Love	8.02 (7.71,8.34)	8.36 (8.07,8.65)	7.58 (6.92,8.23)	< 0.00001	< 0.00001
Forgiveness	0.76 (0.71,0.80)	0.77 (0.72,0.81)	0.73 (0.25,0.96)	< 0.00001	0.00015
Charitable Giving	0.38 (0.30,0.46)	0.35 (0.27,0.44)	0.08 (0.01,0.42)	< 0.00001	< 0.00001
Helping	0.58 (0.50,0.65)	0.54 (0.46,0.61)	0.55 (0.18,0.87)	< 0.00001	< 0.00001
Volunteering	0.23 (0.18,0.29)	0.20 (0.15,0.25)	0.22 (0.03,0.70)	< 0.00001	< 0.00001
Physical health & health behavior					
Self-Rated Physical Health^e^	7.34 (7.06,7.63)	7.09 (6.81,7.38)	6.29 (5.83,6.74)	< 0.00001	< 0.00001
Health Limitations (n)	0.19 (0.17,0.22)	0.22 (0.19,0.25)	0.06 (0.01,0.28)	< 0.00001	0.00001
Pain (n)	0.40 (0.36,0.44)	0.47 (0.43,0.52)	0.30 (0.06,0.73)	< 0.00001	< 0.00001
Smoking (n)	3.33 (2.15,4.51)	1.39 (0.81,1.97)	0.86 (0.41,1.31)	< 0.00001	< 0.00001
Drinking (n)	2.36 (1.68,3.04)	1.23 (0.79,1.67)	2.18 (1.44,2.91)	< 0.00001	< 0.00001
Exercise	2.69 (2.38,3.01)	2.21 (1.93,2.49)	2.13 (1.84,2.42)	< 0.00001	< 0.00001
Socioeconomic outcomes					
Financial Stability^f^	5.79 (5.39,6.19)	5.40 (4.93,5.86)	4.71 (4.08,5.35)	< 0.00001	< 0.00001
Material Stability^f^	6.06 (5.62,6.51)	5.73 (5.21,6.26)	5.18 (4.46,5.91)	< 0.00001	< 0.00001
Education	0.15 (0.10,0.22)	0.14 (0.08,0.22)	0.04 (0.00,0.24)	< 0.00001	0.00002
Employment	0.67 (0.64,0.70)	0.45 (0.39,0.52)	0.60 (0.18,0.91)	< 0.00001	< 0.00001
Subjective Financial Well-Being	0.69 (0.60,0.76)	0.64 (0.55,0.72)	0.47 (0.13,0.83)	< 0.00001	0.00001
Housing	0.66 (0.62,0.69)	0.64 (0.60,0.68)	0.51 (0.17,0.84)	< 0.00001	0.13022
Religion/spirituality					
Self-Reported Religion/Spirituality	0.61 (0.50,0.70)	0.66 (0.56,0.75)	0.63 (0.24,0.90)	< 0.00001	< 0.00001
Religious Service Attendance	0.33 (0.22,0.46)	0.33 (0.21,0.47)	0.15 (0.02,0.63)	< 0.00001	< 0.00001
Life after Death Belief	0.53 (0.44,0.63)	0.56 (0.48,0.64)	0.47 (0.11,0.86)	< 0.00001	< 0.00001
Religious Experience	0.36 (0.27,0.46)	0.37 (0.28,0.47)	0.25 (0.06,0.64)	< 0.00001	0.05631
Religious Reading	0.24 (0.16,0.34)	0.26 (0.18,0.38)	0.06 (0.00,0.46)	< 0.00001	0.00002
Prayer-Meditation	0.45 (0.33,0.58)	0.54 (0.39,0.67)	0.42 (0.06,0.89)	< 0.00001	< 0.00001
Belief in God	0.87 (0.73,0.94)	0.90 (0.80,0.96)	0.82 (0.41,0.97)	< 0.00001	< 0.00001
Intrinsic Religiosity	0.71 (0.57,0.82)	0.75 (0.62,0.85)	0.74 (0.26,0.96)	< 0.00001	< 0.00001
Religious Comfort	0.78 (0.63,0.87)	0.83 (0.71,0.90)	0.83 (0.38,0.97)	< 0.00001	< 0.00001
Loved by God	0.81 (0.66,0.90)	0.86 (0.73,0.93)	0.73 (0.28,0.95)	< 0.00001	< 0.00001
Spiritual Punishment (n)	0.31 (0.21,0.43)	0.28 (0.19,0.39)	0.30 (0.10,0.63)	< 0.00001	0.00002
Religious Criticism (n)	0.30 (0.22,0.40)	0.28 (0.20,0.38)	0.35 (0.12,0.68)	< 0.00001	0.00314
Faith-sharing	0.58 (0.48,0.68)	0.60 (0.49,0.70)	0.72 (0.28,0.94)	< 0.00001	0.00302
Family factors					
Ever Married	0.63 (0.58,0.68)	0.69 (0.62,0.75)	0.38 (0.10,0.77)	< 0.00001	< 0.00001
Divorced	0.03 (0.02,0.04)	0.04 (0.02,0.06)	0.00 (0.00,0.01)	< 0.00001	< 0.00001
Children	1.12 (0.83,1.41)	1.22 (0.90,1.54)	0.51 (0.36,0.66)	< 0.00001	< 0.00001

Notes. Est, meta-analytic estimated mean of outcome within group; 95% CI, confidence interval; ^1^MFO Global p-value, a global of whether there is evidence that the outcome differs among, Males, Females or Other groups in any country; ^2^MF Global p-value, a global of whether there is evidence that the outcome differs between Males and Females in any country; (a-f) Items from the SFI domains are presented individually for completeness and denoted with the super-script (a-f) to show which outcomes were combined for which domain. (n) indicates items which are negatively conceptualized and scored (i.e., higher scores are considered worse from a wellbeing perspective. p-value thresholds, *p* < 0.05; Bonferroni corrected thresholds, *p* < 0.05/73=0.00069.

**Table 4:** A summary of the outcome-wide results.

Row	Outcome	All	Arg	Aus	Bra	Egy	Ger	HK	India	Indo	Isr	Jap	Ken	Mex	Nig	Phi	Pol	SA	Spa	Swe	Tan	Tur	UK	US
1	Flourish Index and Domains/8	M 4.5^1^	M 5	=	M 8	F 5	M 6	M 8	F 6	F 6	M 8	F 8	M 7	M 6	F 8	F 5.5	=	M 8	M 8	M 5.5	F 4.5	M 4.5	M 8	M 5
2	Flourish Index	M 0.02	M 0.02	F 0.03	M 0.28	F 0.21	M 0.07	M 0.13	F 0.12	F 0.07	M 0.14	F 0.28	M 0.06	M 0.09	F 0.08	F 0.05	F 0.03	M 0.09	M 0.15	M 0.07	F 0.03	M 0.01	M 0.18	M 0.02
3	Secure Flourish Index	M 0.07	M 0.20	F 0.01	M 0.39	F 0.12	M 0.10	M 0.13	F 0.03	F 0.04	M 0.15	F 0.26	M 0.11	M 0.20	F 0.07	M 0.04	M 0.04	M 0.11	M 0.25	M 0.10	F 0.02	M 0.27	M 0.24	M 0.07
4	Happiness and Life Satisfaction	F 0.02	M 0.06	M 0.09	M 0.19	F 0.37	M 0.07	M 0.10	F 0.22	F 0.21	M 0.16	F 0.44	F 0.08	M 0.08	F 0.15	F 0.07	M 0.02	M 0.02	M 0.19	F 0.03	F 0.39	F 0.23	M 0.31	F 0.01
5	Social Relationship Quality	F 0.03	F 0.06	F 0.14	M 0.33	F 0.29	F 0.04	M 0.17	F 0.35	M 0.06	M 0.06	F 0.39	M 0.07	F 0.05	F 0.04	=	F 0.03	M 0.08	M 0.03	F 0.11	=	F 0.01	M 0.02	F 0.04
6	Meaning and Purpose	F 0.05	F 0.11	F 0.11	M 0.15	F 0.49	=	M 0.12	F 0.22	F 0.07	M 0.11	F 0.29	M 0.03	F 0.03	F 0.07	F 0.01	F 0.12	M 0.03	M 0.14	=	F 0.06	F 0.15	M 0.18	F 0.06
7	Character and Virtue	=	F 0.15	M 0.03	M 0.03	M 0.27	M 0.11	M 0.08	F 0.11	F 0.11	M 0.08	F 0.06	M 0.09	M 0.11	F 0.07	F 0.11	F 0.23	M 0.02	M 0.03	M 0.17	M 0.15	=	M 0.12	M 0.04
8	Self-Rated Health	M 0.23	M 0.36	M 0.15	M 0.72	M 0.34	M 0.20	M 0.21	M 0.28	F 0.03	M 0.30	F 0.25	M 0.15	M 0.33	F 0.06	F 0.06	M 0.15	M 0.31	M 0.36	M 0.32	M 0.22	M 0.48	M 0.29	M 0.17
9	Financial and Material Security	M 0.27	M 1.10	M 0.10	M 0.93	M 0.36	M 0.30	M 0.10	M 0.39	M 0.15	M 0.22	F 0.15	M 0.38	M 0.72	F 0.03	M 0.45	M 0.30	M 0.25	M 0.78	M 0.26	M 0.01	M 0.44	M 0.57	M 0.29
**10**	**Psychological Well-Being/16**	**M** **10**	**M** **10.5**	**M** **8.5**	**M** **14**	**F** **10**	**M** **12.5**	**M** **12**	**F** **9**	**F** **9**	**M** **16**	**F** **14**	**M** **11**	**M** **12**	**F** **13**	**=**	**M** **11**	**M** **13.5**	**M** **12.5**	**M** **10**	**F** **8.5**	**=**	**M** **14.5**	**=**
11	Happiness	F 0.05	M 0.02	F 0.07	M 0.16	F 0.30	M 0.04	M 0.13	F 0.19	F 0.23	M 0.16	F 0.44	F 0.20	M 0.03	F 0.16	F 0.06	M 0.03	M 0.01	M 0.17	F 0.05	F 0.16	F 0.24	M 0.34	=
12	Life Satisfaction	F 0.07	M 0.10	F 0.10	M 0.22	F 0.44	M 0.08	M 0.06	F 0.26	F 0.20	M 0.17	F 0.44	M 0.04	M 0.11	F 0.16	F 0.09	M 0.05	M 0.03	M 0.21	F 0.01	F 0.72	F 0.23	M 0.26	F 0.01
13	Present Life Evaluation	F 0.07	=	M 0.04	M 0.05	F 0.49	M 0.06	M 0.10	F 0.45	F 0.44	M 0.27	F 0.38	=	M 0.02	F 0.10	F 0.15	M 0.04	F 0.18	M 0.11	F 0.02	F 0.23	M 0.01	M 0.29	F 0.03
14	Future Life Evaluation	F 0.11	F 0.25	M 0.03	F 0.21	F 0.34	M 0.03	F 0.12	F 0.17	F 0.30	M 0.06	F 0.37	F 0.02	F 0.08	F 0.04	F 0.23	M 0.11	F 0.01	F 0.01	F 0.05	F 0.07	F 0.19	F 0.01	F 0.13
15	Optimism	F 0.15	F 0.39	F 0.28	F 0.17	F 0.16	F 0.09	F 0.08	F 0.20	F 0.13	F 0.08	F 0.38	F 0.09	F 0.17	F 0.15	F 0.17	F 0.11	M 0.16	F 0.03	M 0.12	M 0.02	F 0.61	M 0.12	F 0.21
16	Freedom	M 0.01	F 0.03	F 0.23	M 0.10	M 0.40	F 0.04	M 0.10	M 0.16	M 0.12	M 0.13	F 0.34	F 0.11	M 0.01	M 0.08	M 0.05	F 0.04	M 0.09	F 0.03	F 0.13	M 0.19	F 0.06	M 0.02	F 0.09
17	Peace	M 0.02	M 0.01	=	M 0.11	F 0.04	M 0.03	M 0.02	F 0.01	=	M 0.01	F 0.07	M 0.06	M 0.03	F 0.01	M 0.02	M 0.01	=	M 0.03	M 0.05	M 0.03	F 0.03	M 0.07	M 0.04
18	Balance in Life	M 0.02	M 0.03	=	M 0.08	F 0.02	M 0.02	M 0.01	=.	F 0.02	M 0.03	F 0.06	M 0.07	M 0.07	M 0.04	M 0.06	=	M 0.01	M 0.05	M 0.06	M 0.02	M 0.01	M 0.06	=
19	Mastery	M 0.03	M 0.03	M 0.02	M 0.09	F 0.01	M 0.04	M 0.02	M 0.02	M 0.03	M 0.02	F 0.01	M 0.05	M 0.02	M 0.01	M 0.03	=	M 0.03	=	M 0.04	M 0.03	F 0.01	M 0.03	M 0.01
20	Meaning	F 0.11	F 0.19	F 0.13	M 0.05	F 0.60	F 0.08	M 0.11	F 0.38	F 0.22	M 0.09	F 0.39	M 0.03	F 0.06	F 0.05	M 0.04	F 0.11	M 0.03	M 0.09	F 0.08	F 0.16	F 0.34	M 0.14	F 0.09
21	Purpose	M 0.01	F 0.03	F 0.09	M 0.24	F 0.37	M 0.08	M 0.12	F 0.06	M 0.09	M 0.12	F 0.19	M 0.02	F 0.01	F 0.10	F 0.04	F 0.12	M 0.04	M 0.19	M 0.07	M 0.05	M 0.04	M 0.21	F 0.04
22	Self-Rated Mental Health	M 0.19	M 0.30	M 0.09	M 0.93	M 0.07	M 0.28	M 0.20	M 0.17	F 0.04	M 0.24	F 0.25	M 0.10	M 0.25	F 0.16	F 0.06	M 0.10	M 0.21	M 0.42	M 0.36	M 0.04	M 0.34	M 0.37	M 0.31
23	Traumatic Distress (n)	M 0.04	M 0.10	M 0.06	M 0.11	M 0.06	M 0.06	F 0.01	M 0.02	=	M 0.08	M 0.01	M 0.02	M 0.05	F 0.04	=	M 0.03	M 0.02	M 0.03	M 0.09	F 0.2	M 0.06	M 0.03	M 0.07
24	Depression Symptoms (n)	M 0.03	M 0.07	=	M 0.9	M 0.04	=	M 0.01	=	M 0.01	M 0.06	F 0.02	=	M 0.05	F 0.03	M 0.05	M 0.02	M 0.03	M 0.04	M 0.04	F 0.2	M 0.03	=	M 0.01
25	Anxiety Symptoms (n)	M 0.05	M 0.10	M 0.04	M 0.12	M 0.13	M 0.02	M 0.01	M 0.05	M 0.02	M 0.04	M 0.01	M 0.01	M 0.04	F 0.01	M 0.07	M 0.01	M 0.03	M 0.06	M 0.05	=	M 0.02	M 0.05	M 0.08
26	Suffering (n)	M 0.05	M 0.08	M 0.07	M 0.13	M 0.09	M 0.06	F 0.02	M 0.06	M 0.01	M 0.07	F 0.01	M 0.02	M 0.02	F 0.01	=	M 0.05	M 0.06	M 0.11	M 0.09	F 0.04	M 0.05	M 0.05	M 0.08
**27**	**Social Well-Being/12**	**F** **7**	**F** **7**	**F** **8**	**F** **7.5**	**F** **8.5/11**	**F** **6.5**	**M** **10**	**F** **7.5**	**=**	**M** **7**	**F** **10.5**	**M** **9**	**=**	**F** **9**	**M** **6.5**	**F** **9**	**M** **6.5**	**M** **8**	**=**	**M** **7**	**F** **6.5**	**M** **6.5**	**=**
28	Content with My Relationships	F 0.04	F 0.06	F 0.14	M 0.25	F 0.39	F 0.01	M 0.14	F 0.36	M 0.08	M 0.04	F 0.40	M 0.16	F 0.08	F 0.07	M 0.01	=	M 0.03	F 0.01	F 0.13	M 0.01	F 0.07	F 0.02	F 0.04
29	Satisfying Relationships	F 0.01	F 0.05	F 0.15	M 0.40	F 0.19	F 0.06	M 0.20	F 0.34	M 0.05	M 0.08	F 0.38	M 0.11	F 0.01	F 0.01	=	F 0.05	M 0.13	M 0.05	F 0.09	M 0.02	M 0.06	M 0.05	F 0.03
30	Social Support	F 0.21	F 0.31	F 0.26	M 0.16	F 0.74	F 0.26	M 0.10	F 0.50	F 0.24	F 0.10	F 0.54	M 0.16	F 0.11	F 0.17	M 0.03	F 0.15	F 0.15	F 0.13	F 0.28	F 0.27	F 0.34	F 0.09	F 0.17
31	Intimate Friend	F 0.03	F 0.04	F 0.06	F 0.02	F 0.03	F 0.03	F 0.03	=	F 0.03	F 0.03	F 0.10	M 0.01	F 0.04	F 0.01	F 0.01	F 0.02	=	F 0.02	F 0.05	M 0.05	F 0.09	F 0.03	F 0.06
32	Government Approval	M 0.03	M 0.03	M 0.03	=	-	M 0.04	M 0.03	F 0.03	M 0.03	M 0.07	M 0.05	=	M 0.08	F 0.01	M 0.02	F 0.03	F 0.01	M 0.05	M 0.10	F 0.02	M 0.03	M 0.08	F 0.03
33	Political Voice	M 0.01	M 0.02	M 0.01	F 0.01	F 0.03	M 0.01	M 0.05	F 0.01	F 0.01	M 0.07	=	=	M 0.06	M 0.04	F 0.01	F 0.02	F 0.01	M 0.04	M 0.03	F 0.01	F 0.07	M 0.09	M 0.01
34	Belonging	F 0.06	F 0.03	F 0.24	F 0.19	F 0.13	F 0.06	M 0.13	M 0.20	M 0.12	F 0.10	F 0.14	M 0.02	M 0.01	F 0.01	M 0.08	F 0.09	F 0.09	M 0.04	M 0.39	M 0.04	=	F 0.22	F 0.09
35	City Satisfaction	F 0.02	F 0.03	F 0.04	M 0.01	F 0.03	=	M 0.02	M 0.01	F 0.01	F 0.02	F 0.05	F 0.01	F 0.01	M 0.03	F 0.02	F 0.02	F 0.04	M 0.01	F 0.02	F 0.07	F 0.10	=	M 0.02
36	Trust	M 0.04	M 0.05	M 0.06	M 0.04	=	M 0.03	M 0.02	M 0.01	M 0.03	M 0.03	F 0.03	M 0.02	M 0.05	F 0.01	F 0.01	F 0.03	M 0.02	M 0.03	M 0.03	M 0.01	M 0.05	M 0.11	M 0.04
37	Community Participation	M 0.04	M 0.05	F 0.02	M 0.09	M 0.06	M 0.03	M 0.04	M 0.07	M 0.08	M 0.04	F 0.02	M 0.11	M 0.08	M 0.08	M 0.07	=	M 0.04	M 0.02	M 0.03	M 0.07	M 0.11	M 0.06	M 0.02
38	Loneliness (n)	M 0.12	M 0.37	F 0.08	M 0.57	F 0.26	M 0.12	M 0.29	F 0.11	F 0.04	M 0.23	F 0.25	M 0.21	M 0.11	F 0.03	M 0.23	M 0.12	M 0.10	M 0.12	M 0.19	M 0.15	M 0.15	M 0.32	M 0.14
39	Discrimination (n)	F 0.02	F 0.02	M 0.03	F 0.02	M 0.03	F 0.02	F 0.04	F 0.01	F 0.04	F 0.01	F 0.02	F 0.03	F 0.04	F 0.05	F 0.04	M 0.02	M 0.01	F 0.04	F 0.01	F 0.02	F 0.02	F 0.05	M 0.01
**40**	**Character and Prosocial Behaviour/9**	**F** **5**	**F** **8**	**F** **8**	**=**	**F** **5.5**	**=**	**M** **7**	**F** **5.5**	**F** **6**	**M** **5.5**	**F** **5.5**	**M** **7**	**M** **6**	**F** **5.5**	**F** **5**	**F** **9**	**M** **7**	**M** **5.5**	**F** **8**	**M** **5**	**F** **5**	**=**	**F** **7**
41	Promoting Good	F 0.04	F 0.22	F 0.06	M 0.08	F 0.10	F 0.09	M 0.14	F 0.08	F 0.12	M 0.06	F 0.20	M 0.05	M 0.10	F 0.03	F 0.08	F 0.26	M 0.04	F 0.06	F 0.13	M 0.13	F 0.10	F 0.01	M 0.06
42	Delayed Gratification	M 0.04	F 0.09	M 0.12	F 0.03	F 0.42	M 0.31	M 0.02	F 0.15	F 0.10	M 0.08	M 0.09	M 0.13	M 0.12	F 0.12	F 0.15	F 0.19	=	M 0.11	F 0.53	M 0.19	M 0.08	M 0.25	M 0.02
43	Hope	F 0.04	F 0.29	F 0.16	=	F 0.47	=	M 0.16	M 0.03	M 0.03	M 0.12	F 0.13	M 0.02	F 0.10	F 0.06	F 0.11	F 0.13	M 0.21	M 0.09	M 0.03	M 0.03	F 0.11	M 0.04	F 0.10
44	Gratitude	F 0.24	F 0.42	F 0.58	F 0.25	F 0.56	F 0.16	F 0.09	F 0.17	F 0.03	F 0.02	F 0.50	F 0.07	F 0.18	F 0.13	F 0.35	F 0.26	=	F 0.06	F 0.51	F 0.06	F 0.26	F 0.11	F 0.38
45	Love	F 0.34	F 0.63	F 0.76	F 0.29	F 0.47	F 0.34	M 0.01	F 0.19	F 0.13	F 0.36	F 0.67	F 0.04	F 0.16	F 0.17	F 0.13	F 0.43	=	F 0.37	F 0.69	M 0.03	F 0.50	F 0.61	F 0.47
46	Forgiveness	F 0.01	F 0.06	F 0.04	M 0.01	=	F 0.01	M 0.03	F 0.02	F 0.02	F 0.04	F 0.05	M 0.02	M 0.01	=	M 0.02	F 0.10	=	=	F 0.01	M 0.02	M 0.05	F 0.03	F 0.01
47	Charitable Giving	M 0.03	F 0.01	F 0.09	M 0.01	M 0.06	M 0.15	M 0.01	=	F 0.03	M 0.03	M 0.01	M 0.04	M 0.03	M 0.10	M 0.02	F 0.03	M 0.03	M 0.12	F 0.08	F 0.13	M 0.05	=	F 0.04
48	Helping	M 0.04	=	F 0.06	M 0.03	M 0.07	M 0.17	F 0.04	M 0.07	M 0.06	M 0.04	=	M 0.06	M 0.07	M 0.04	M 0.03	F 0.02	M 0.10	M 0.16	F 0.03	F 0.07	M 0.04	M 0.01	F 0.02
49	Volunteering	M 0.03	=	F 0.02	F 0.01	M 0.03	M 0.10	M 0.02	M 0.06	M 0.08	=	0.02	M 0.03	M 0.06	M 0.15	M 0.05	F 0.03	M 0.04	M 0.10	F 0.01	F 0.05	M 0.03	M 0.04	F 0.02
**50**	**Physical Health and Health Behaviour/6**	**M** **4**	**M** **4**	**M** **4**	**M** **4**	**M** **4**	**M** **4**	**F** **4**	**M** **4**	**F** **3.5**	**M** **4**	**F** **4**	**M** **4**	**M** **4**	**=**	**F** **4.5**	**M** **4**	**M** **4**	**M** **4**	**M** **5**	**M** **4**	**M** **4**	**M** **4**	**M** **4**
51	Self-Rated Physical Health	M 0.25	M 0.40	M 0.21	M 0.52	M 0.61	M 0.13	M 0.20	M 0.39	F 0.02	M 0.37	F 0.25	M 0.19	M 0.39	M 0.03	F 0.04	M 0.21	M 0.40	M 0.30	M 0.27	M 0.38	M 0.62	M 0.20	M 0.04
52	Health Limitations (n)	M 0.03	M 0.05	M 0.09	M 0.01	M 0.09	M 0.01	F 0.02	M 0.05	=	M 0.02	F 0.01	M 0.05	M 0.06	=	F 0.03	M 0.01	M 0.06	M 0.01	M 0.09	M 0.07	M 0.04	M 0.05	M 0.05
53	Pain (n)	M 0.07	M 0.11	M 0.09	M 0.14	M 0.11	M 0.08	F 0.03	M 0.09	M 0.05	M 0.07	M 0.02	M 0.10	M 0.07	=	=	M 0.06	M 0.09	M 0.09	M 0.09	M 0.05	M 0.09	M 0.05	M 0.06
54	Smoking (n)	F 1.94	F 1.12	F 0.52	F 1.01	F 6.27	F 0.49	F 0.68	F 0.96	F 6.93	F 3.62	F 2.74	F 0.45	F 1.31	F 0.37	F 2.84	F 2.95	F 1.66	F 1.14	M 0.20	F 0.41	F 6.98	F 0.38	F 0.09
55	Drinking (n)	F 1.13	F 1.10	F 2.67	F 1.08	F 0.01	F 1.17	F 0.68	F 0.41	F 0.21	F 0.92	F 2.42	F 0.44	F 1.21	F 0.31	F 1.08	F 1.69	F 1.78	F 1.14	F 1.45	F 0.42	F 1.10	F 1.75	F 1.65
56	Exercise	M 0.48	M 0.51	M 0.33	M 0.54	M 0.57	M 0.15	M 0.42	M 0.85	M 0.52	M 0.16	M 0.26	M 1.14	M 0.63	M 0.84	M 0.48	M 0.21	M 0.61	M 0.36	M 0.01	M 1.35	M 0.09	M 0.28	M 0.29
**57**	**Socioeconomic Outcomes/6**	**M** **6**	**M** **6**	**M** **4**	**M** **5.5**	**M** **6**	**M** **6**	**M** **5**	**M** **5.5**	**M** **4.5**	**M** **5**	**F** **4.5**	**M** **6**	**M** **6**	**F** **3.5**	**M** **5**	**M** **5**	**M** **5**	**M** **5**	**M** **5**	**M** **5**	**M** **6**	**M** **6**	**M** **5**
58	Financial Stability	M 0.39	M 1.09	M 0.16	M 0.95	M 0.34	M 0.34	F 0.04	M 0.42	M 0.06	M 0.22	F 0.14	M 0.39	M 0.77	F 0.05	M 0.57	M 0.35	M 0.35	M 0.83	M 0.36	F 0.04	M 0.45	M 0.72	M 0.40
59	Material Stability	M 0.33	M 1.11	M 0.04	M 0.92	M 0.36	M 0.28	M 0.04	M 0.40	M 0.23	M 0.22	F 0.19	M 0.37	M 0.68	F 0.01	M 0.34	M 0.24	M 0.15	M 0.74	M 0.16	M 0.06	M 0.42	M 0.41	M 0.17
60	Education	M 0.01	M 0.01	F 0.08	=	M 0.01	M 0.05	M 0.05	=	=	F 0.03	F 0.03	M 0.04	M 0.01	=	F 0.01	F 0.06	M 0.02	F 0.01	F 0.03	M 0.01	M 0.03	M 0.04	F 0.02
61	Employment	M 0.22	M 0.22	M 0.08	M 0.18	M 0.59	M 0.03	M 0.02	M 0.41	M 0.43	M 0.07	M 0.18	M 0.20	M 0.37	M 0.05	M 0.32	M 0.14	M 0.18	M 0.07	M 0.08	M 0.29	M 0.34	M 0.07	M 0.11
62	Subjective Financial Well-Being	M 0.05	M 0.09	M 0.03	M 0.09	M 0.03	M 0.03	M 0.2	M 0.06	F 0.01	M 0.05	=	M 0.06	M 0.12	F 0.01	M 0.03	M 0.06	M 0.01	M 0.06	M 0.02	M 0.04	M 0.07	M 0.05	M 0.06
63	Housing	M 0.02	M 0.02	F 0.03	M 0.02	M 0.02	M 0.04	M 0.06	M 0.03	M 0.02	M 0.01	F 0.01	M 0.01	M 0.04	M 0.02	M 0.02	M 0.01	F 0.05	M 0.03	M 0.03	M 0.02	M 0.05	M 0.03	M 0.03
**64**	**FLOURISH TOTAL** **/57**	**M** **33.5**	**M** **31.5**	**F** **31.5**	**M** **42.5**	**F** **31**	**M** **40**	**M** **44**	**F** **32.5**	**F** **32**	**M** **45.5**	**F** **46.5**	**M** **44**	**M** **40**	**F** **42**	**F** **29.5**	**F** **30**	**M** **44**	**M** **43**	**M** **32.5**	**M** **30**	**M** **32**	**M** **43.5**	**M** **30**
**65**	**Religion/Spirituality** **/13**	**F** **12.5**	**F** **13**	**F** **13**	**F** **12**	**F** **8.5**	**F** **9.5**	**F** **8**	**F** **9.5**	**M** **7**	**F** **9**	**F** **8.5**	**F** **10/5**	**F** **10.5**	**F** **11.5**	**F** **11**	**F** **11.5**	**F** **11**	**M** **8**	**F** **11**	**F** **10.5**	**F** **10**	**F** **7**	**F** **12.5**
66	Self-Reported Religion/Spirituality	F 0.05	F 0.10	F 0.14	F 0.06	F 0.02	F 0.02	M 0.01	F 0.02	F 0.01	F 0.02	F 0.02	F 0.03	F 0.04	F 0.02	F 0.06	F 0.11	F 0.09	M 0.01	F 0.07	F 0.04	F 0.04	=	F 0.10
67	Religious Service Attendance	=	F 0.05	F 0.04	F 0.03	M 0.24	M 0.01	=	M 0.03	M 0.11	M 0.11	M 0.01	F 0.11	F 0.07	F 0.01	F 0.07	F 0.13	F 0.20	M 0.04	M 0.01	F 0.06	M 0.10	M 0.06	F 0.03
68	Life after Death Belief	F 0.03	F 0.08	F 0.17	M 0.02	M 0.10	F 0.03	F 0.06	F 0.02	=	M 0.02	F 0.05	F 0.02	=	M 0.06	F 0.03	F 0.11	M 0.01	F 0.02	F 0.13	M 0.06	F 0.01	F 0.04	F 0.09
69	Religious Experience	F 0.01	F 0.05	F 0.04	F 0.03	F 0.01	F 0.02	M 0.03	=	M 0.04	F 0.02	=	F 0.01	M 0.01	=	M 0.02	F 0.04	F 0.09	M 0.02	F 0.03	F 0.02	M 0.01	M 0.02	F 0.03
70	Religious Reading	F 0.02	F 0.04	F 0.03	F 0.05	F 0.06	M 0.02	M 0.01	F 0.01	F 0.05	M 0.03	M 0.01	F 0.09	F 0.02	F 0.02	F 0.03	F 0.04	F 0.08	M 0.01	M 0.01	F 0.01	F 0.11	M 0.05	F 0.06
71	Prayer-Meditation	F 0.11	F 0.13	F 0.09	F 0.10	F 0.08	=	M 0.01	F 0.06	M 0.07	M 0.04	=	F 0.06	F 0.07	F 0.03	F 0.11	F 0.14	F 0.12	=	F 0.03	F 0.04	F 0.14	=	F 0.11
72	Belief in God	F 0.03	F 0.08	F 0.15	F 0.04	=	F 0.01	F 0.02	F 0.03	=	F 0.02	=	F 0.01	F 0.04	F 0.01	F 0.02	F 0.06	F 0.03	F 0.03	F 0.12	=	F 0.05	F 0.04	F 0.10
73	Intrinsic Religiosity	F 0.04	F 0.06	F 0.09	F 0.06	F 0.01	=	F 0.02	F 0.03	=	F 0.03	F 0.04	F 0.02	F 0.05	F 0.01	F 0.01	F 0.12	F 0.04	M 0.01	F 0.04	F 0.02	F 0.07	M 0.01	F 0.08
74	Religious Comfort	F 0.05	F 0.09	F 0.14	F 0.07	=	F 0.02	F 0.04	F 0.02	M 0.01	F 0.03	F 0.04	F 0.01	F 0.07	F 0.01	F 0.01	F 0.14	F 0.05	=	F 0.07	F 0.01	F 0.08	F 0.05	F 0.09
75	Loved by God	F 0.05	F 0.09	F 0.14	F 0.05	=	F 0.02	F 0.03	F 0.03	M 0.01	F 0.02	F 0.08	=	F 0.06	F 0.01	F 0.01	F 0.12	F 0.03	M 0.01	F 0.07	F 0.01	F 0.07	F 0.03	F 0.10
76	Spiritual Punishment (n)	F 0.03	F 0.03	F 0.04	F 0.03	F 0.03	=	F 0.02	M 0.03	F 0.07	F 0.04	F 0.01	M 0.02	F 0.04	F 0.01	F 0.14	M 0.02	M 0.03	F 0.02	F 0.01	=	F 0.13	F 0.05	=
77	Religious Criticism (n)	F 0.02	F 0.01	F 0.02	F 0.01	F 0.01	F 0.01	F 0.04	M 0.02	F 0.06	F 0.01	F 0.01	M 0.02	F 0.01	F 0.01	F 0.05	=	F 0.02	F 0.04	F 0.01	=	F 0.10	F 0.05	F 0.01
78	Faith-sharing	F 0.02	F 0.02	F 0.03	F 0.04	M 0.02	F 0.01	=	F 0.02	=	F 0.04	M 0.01	F 0.02	M 0.01	F 0.03	M 0.01	F 0.06	F 0.05	M 0.02	F 0.03	F 0.02	M 0.01	M 0.01	F 0.03
**79**	**Family Factors/3**	**F** **2**	**F** **2**	**F** **2**	**M** **2**	**M** **2**	**M** **2**	**M** **2**	**F** **2.5**	**F** **2**	**F** **2**	**M** **2**	**F** **2.5**	**F** **2.5**	**=**	**F** **2.5**	**M** **2**	**=**	**M** **2**	**M** **2**	**F** **2**	**M** **2.5**	**M** **2**	**F** **2**
80	Ever Married	F 0.06	F 0.04	F 0.05	M 0.05	F 0.14	F 0.05	M 0.06	F 0.15	F 0.11	F 0.05	F 0.08	F 0.07	F 0.02	F 0.06	F 0.06	F 0.04	M 0.01	F 0.01	F 0.03	F 0.08	M 0.08	F 0.02	F 0.01
81	Divorced (n)	M 0.01	M 0.01	M 0.02	M 0.01	M 0.02	M 0.04	M 0.01	=	M 0.02	M 0.04	M 0.05	=	=.	=	=	M 0.02	=	M 0.02	M 0.03	M 0.01	=	M 0.04	M 0.03
82	Children	F 0.10	F 0.17	F 0.01	F 0.15	F 0.13	F 0.01	F 0.02	F 0.14	F 0.09	F 0.01	M 0.08	F 0.36	F 0.19	M 0.25	F 0.21	M 0.01	F 0.81	M 0.10	M 0.03	F 0.04	M 0.01	M 0.06	F 0.14
**83**	**TOTAL/73**	**F** **37.5**	**F** **40.5**	**F** **46.5**	**M** **43.5**	**F** **40.5/77**	**M** **44.5**	**M** **51**	**F** **49.5**	**F** **39**	**M** **50.5**	**F** **58**	**M** **46**	**F** **43**	**F** **52**	**F** **41**	**F** **42.5**	**M** **51.5**	**M** **53**	**=**	**M** **44.5**	**M** **44.5**	**M** **51.5**	**F** **42.5**
**84**	**Gender equality ranking (global and GFS)**		36/146 9/21^2^	26/146 7/21	57/146 12/21	134/146 21/21	5/146 1/21	**-**	127/146 18/21	87/146 16/21	83/146 15/21	125/146 17/21	76/146 14/21	32/146 8/21	130/146 20/21	16/146 4/21	60/146 13/21	20/146 6/21	17/146 5/21	6/146 2/21	47/146 11/21	129/146 19/21	15/146 3/21	43/146 10/21

Note. The M and F labels indicate which sex does better in terms of flourishing on a given item. Therefore, on items which are negatively conceptualized and scored, as indicated with (n), the sex who does better is the one who scores lower.

^1^ = A 0.5 score is awarded in cases where male and female are equal. In the case of overall ***Secure Flourish Index and Domains***, males do better on 4 out of 8, and females on 3 out of 8, with one item shared, so males are assessed as doing better on the category overall, with 4.5/8. ^2^ Hong Kong not included in equality rankings.

## Discussion

This analysis affords a unique insight into sex differences in flourishing, both overall and in its myriad dimensions, and moreover across the GFS as a whole while also in different countries. The first question is, who fares better in overall flourishing? While there are various ways of construing this, if appraised in terms of VanderWeele’s^28^ 12-item Secure Flourish Index (SFI)—featuring the five main domains of his flourishing framework, plus an additional sixth domain of financial and material security that helps people “secure” the main domains, with two items per domain—males report faring slightly better, albeit only marginally, being 0.07 higher. This finding can be seen in Row 3 (R3) of the first column in Table 4, to which we will refer repeatedly in this Discussion (using R to denote the relevant row). However, on the 10-item “Flourish Index” (i.e., minus financial and material security) in R2, males are only a negligible 0.02 higher. We should also emphasize that these figures only apply to the GFS overall, and there are many notable country-level variations, as we delve into below, including nine nations where *females* instead report higher overall flourishing (Australia, Egypt, India, Indonesia, Japan, Nigeria, Philippines, Poland, and Tanzania). Across the dataset collectively though, males report doing slightly better on both flourishing indices, and also cumulatively across all 57 items and indices pertaining to VanderWeele’s framework (see R64), on which males score higher on 33.5 of these. We note though that *females* report doing better if we expand our view of flourishing further to encompass domains that are not in VanderWeele’s framework per se but nevertheless have been included in the GFS as pertaining to flourishing, including religion/spirituality (R65-78) and family factors (R79-82). If these were to be interpreted as also constitutive of flourishing, females score higher than males on *all* but one of the 13 religion/spirituality items (with religious attendance being equal), so if all 73 items and indices are considered together, females report slightly higher flourishing overall (37.5).

In any case, beyond a gestalt look at sex differences in overall flourishing, a more meaningful analysis comes from examining its different domains and outcomes (and note that, in the text here, any figure with a percentage sign after it denotes a binary outcome, and any figure without it a continuous outcome). Of the six SFI domains, females report being higher on three (happiness and satisfaction, social relationship quality, and meaning and purpose), and males on two (self-rated health, and financial and material security), and with character/virtue being equal. However, the reason males report doing better on the SFI itself is that females only score marginally higher on the three in which they outperform males (0.06, 0.03, and 0.05 respectively), whereas the gaps on the two in which males outscore females are considerably bigger (0.23 and 0.27 respectively). These patterns play out across the items more generally. For example, in terms of social wellbeing (R27), females report doing better, but not overwhelmingly, scoring higher than males on 7/10 items. By contrast, for the socioeconomic outcomes (R57), males report doing better on all six. Nevertheless, these general patterns seem to reinforce general trends that are widely observed in the literature and indeed have become the basis for common sex-related stereotypes in public discourse more broadly. Crucially however, there is also considerable country-level variation on all these domains, with many countries subverting or complicating the general trends, suggesting that these broad sex-related patterns are far from universal or inevitable, but instead are contingent on the socio-cultural dynamics of particular places. With that in mind, let us briefly review all the flourishing domains, examining both the general trends and the national-level variation.

### Happiness and life satisfaction

The first domain is happiness and life satisfaction, which aligns with Diener’s (1984) influential and extensively researched notion of SWB. Here, SWB is directly captured by four items, on all of which females report doing slightly better, including happiness (by 0.05; shown in R14), life satisfaction (0.07; R15), present life evaluation (0.07; R16), and future life evaluation (0.11; R17). These trends align with prior literature; although one review^34^ found sex-related reports around SWB are “inconsistent and conflicting,” there are certainly many studies that have observed higher levels in females. As noted earlier, for instance, Kahneman and Deaton’s^36^ analysis of GWP data found females had higher life satisfaction and positive affect, but *also* greater stress and negative affect, which aligns with females faring worse in our data on anxiety and depression, as considered next. Additionally, this domain includes some items that are less directly related to SWB, with males scoring marginally higher on inner peace (0.02; R17) and life balance (0.02; R18), and females higher on optimism (0.15; R15). While there is much that can be unpacked in future work, a point we would especially emphasize is the cross-national variation. Taking the domain as a whole (R4), although females reported doing better overall, this only occurred in 11 individual places (Egypt, India, Indonesia, Japan, Kenya, Nigeria, Philippines, Sweden, Tanzania, Turkey, and US), while in the other 11 males did better (Argentina, Australia, Brazil, Germany, Hong Kong, Israel, Mexico, Poland, South Africa, Spain, and UK). While these lists are heterogenous, one tentative observation is that the places where females report doing better are arguably less WEIRD, excluding Sweden and the US (though in the US females were only 0.01 higher, so were essentially equal). Such patterns raise obvious questions about the problem of generalizing from relatively WEIRD populations to the rest of the world, which, despite efforts to make scholarship more global^41^, is still an issue in psychology^39^.

### Health

Closely related to the first domain is physical health, and especially mental health. Here, males generally reported faring better, including on self-rated mental health (0.19; R22), traumatic distress (0.03; R23), depression symptoms (3%; R24), anxiety symptoms (5%; R25), and suffering (0.05; R26). Such figures align with the consensus that higher rates of anxiety and depression are “one of the most widely documented findings in psychiatric epidemiology”^21^, though we must also bear in mind the argument, raised earlier, that males may experience and express such conditions in more “externalising” ways that miss detection by conventional diagnostic criteria. Additionally, females also mostly report doing worse on physical health, with males higher on self-rated physical health (0.25; R51), health limitations (0.03; R52), pain (0.07; R52), and exercise (0.48; R53), although females report doing far better on smoking (1.94; R55) and drinking (1.13; R56), which perhaps lends credence to the idea that males externalize their distress and engage in more health risk behaviours, to their detriment^25^. Again though, we must emphasize the national variation. While males scored higher on physical and mental health in most countries, there are exceptions (see R8), including Indonesia, Japan, Nigeria, and Philippines. Further investigation is needed to explore why females in these countries do better relative to males. One plausible explanation could be that these nations are particularly strong on gender equality. But although Philippines does very well in that regard (see R90), ranked 16th out of 146 nations by the World Economic Forum^58^, and hence 4th in the GFS (out of 21, with Hong Kong not ranked), Indonesia (87th/15th), Japan (125th/16th), and Nigeria (130th/19th) are much lower, and so the gender equality hypothesis does not seem viable, and other explanations must be sought.

### Meaning and purpose

The next two domains embrace concepts that have been influentially operationalized by Ryff^59^ as “psychological wellbeing,” which is in turn drawn from the classical Greek idea of “eudaimonia,” which in modern terms might be best translated as  “good conscience.” The first of these domains is meaning and purpose, on which females scored marginally higher overall (0.05; R6), though they fare especially well on meaningful activity (0.11; R20), while males instead report fractionally more understanding of their purpose (0.01; R21), which reinforces the idea that the constructs are not synonymous^60^. In trying to make sense of the overall lower scores for males on this domain, one wonders about the relevance of claims over recent years around an escalating “crisis of masculinity”^61^. There are many facets to this crisis, but key among them is the suggestion that two of the great sources of meaning are having/raising children and work, with females historically taking the lead in the former and males in the latter. In the modern era though, while females still take a larger role in childcare (especially given their biological role in bearing children), they are also increasingly finding parity in relation to work, leaving males unsure about their particular value or role.

Females do of course still face particular struggles compared to males in many areas of life (as we consider below in relation to financial and material security), including in the workplace (e.g., with respect to pay). But it is also notable that, in at least some work-related aspects of life, such as educational attainment, females are now surpassing males in many places^62^. Furthermore, exacerbating the potential crisis for males is that this emergent inequity is often celebrated as a *good*. An article in The Economist^63^ for example, discussing a recent study in Nature showing girls still falling behind in maths^64^, began “For decades a big story in education has been the ascent of girls. They now outperform boys in most subjects, leave school with better grades and are more likely to get a university degree. But one subject remains a problem: across much of the world, girls lag behind in mathematics.” By framing the one subject on which males still do better as the “problem,” the article thus explicitly suggests that the ideal is a system in which females do better on *all* subjects. One finds a similar bias in the World Economic Forum’s^65^ Global Gender Gap Report, where inequality is only defined as *females* lagging behind, whereas instances where males do worse are not considered inequality; as their methodology states, “the index rewards countries that reach the point where outcomes for women equal those for men, but it neither rewards nor penalizes cases in which women are outperforming men” (p.70). Taken to an extreme, this kind of discourse has led to the idea, as captured in the title of an article in TIME, that “Men Are Obsolete”^66^. As a result, the crisis of masculinity partly involves males overall becoming increasingly unsure of their purpose and role in the modern world. As such, the current study adds credence to the need for reframing men’s roles through a “positive” masculinity that includes an emphasis on meaning and purpose^67,68^. We must again note the regional variation though, with males nevertheless having higher combined meaning and purpose (R6) in seven countries out of 22 (Brazil, Hong Kong, Israel, Kenya, South Africa, Spain, UK), and equal in two (Germany and Sweden). So, even if lower meaning/purpose among males *is* linked to a potential masculinity crisis, this issue is not playing out identically in all places.

### Character and virtue

The fourth domain, which also falls under the ambit of psychological wellbeing^59^, is character and virtue, on which males and females were exactly equal. There are some interesting nuances though when differentiating this into nine aspects, with females scoring slightly higher on promoting good (0.04; R41), hope (0.04; R42), and forgiveness (0.01; R46), and substantially higher on gratitude (0.24; R44) and love (0.34; R45), while males reported doing marginally better on delaying gratification (0.04; R42), charitable giving (0.03; R47), helping strangers (0.04; R48), and volunteering (0.03; R49). Based on these trends, one observation that stands out is that females report doing better on items focused on emotions and attitudes, whereas males report faring slightly better on more action-oriented aspects, which perhaps maps onto the idea that females tend to exhibit more “internalizing” aspects of character and males more “externalizing” aspects. We should emphasize though that the differences in most items are relatively small, and that, apart from love and gratitude, males and females have fairly similar scores. One must thus be wary of over-extrapolating from these minor differences to create or reinforce sex-based stereotypes, as for instance found in the popular literature around “love languages,” in which males supposedly show their love primarily through “acts of service”^69^. This caution is then amplified when considering the country-level nuances. While females in general score far higher for love, for instance, males report doing better in Hong Kong and Tanzania, and conversely while males score higher overall on charitable giving, females report doing better in six countries (Argentina, Australia, Poland, Sweden, Tanzania, and US), and are equal in two (India and UK). As with all variables, any general sex-specific patterns are not universal but are dependent on local socio-cultural dynamics.

### Social relationships

The same point applies to the fifth category of social relationships. Overall, females reported doing marginally better (0.03; R5), which aligns with the general consensus on this topic, whereby males are generally regarded as faring worse, both in terms of quantity (i.e., fewer relationships) and quality (i.e., less intimacy in the ties people have)^70^. Yet the full picture is more complex. Females tend to report doing better on close relationships, scoring higher—albeit fairly marginally—on contentment with relationships (0.04; R28), relationship satisfaction (0.01; F29), social support (0.03; R30), and having an intimate friend (0.03; R31), as well as some broader forms of social capital^71^, namely belonging (0.06; R34), being satisfied with one’s city (0.02; R35), and not experiencing discrimination (0.02; R39). Conversely, males report doing slightly better on items related to broader social capital, which one could describe collectively as civic attitudes and participation, including government approval (0.03; R33), political voice (0.01; R34), and community participation (0.04; R37). Finally, notably, despite the aforementioned concern, and general stereotype, that males have poorer social relationships, here they report being less lonely overall (0.11; R38), possibly because, despite faring worse on *close* social relationships, they report having more relationships overall due to factors such as greater community participation. Despite such stereotypes, the findings represent a complex picture, especially when one further takes national variation into account. In terms of overall social relationship quality (R5), in contrast to females reporting doing better on this domain across the GFS, males scored higher in Brazil (by a large gap of 0.33), Hong Kong, Indonesia, Israel, Kenya, South Africa, Spain, and the UK, and are equal in Philippines and Tanzania. As with all domains, any general trends and conclusions need to be considered in light of the numerous country-level exceptions.

### Financial and material security

The final domain of VanderWeele’s SFI is financial and material security. This domain is treated somewhat differently, since VanderWeele does not regard such security as a universal “end” in itself, but as nevertheless important “in the preservation of those goods that are their own ends” (i.e., the five main domains), and hence a means of securing flourishing over time. Of all the domains, this had the largest sex-related gap, with males reporting doing better overall by 0.27 (R9), and on all items: financial stability (0.39; R58), material stability (0.33; R59), education (0.01; R60), employment (0.22; R61), subjective financial wellbeing (0.05; R62), and housing (0.02; R63). Here perhaps we see traces of the idea that many societies are still relatively patriarchal. While a complex and contested concept, it essentially reflects the notion that males have more power and control than females across different aspects of life, especially public or societal arenas like politics and the workplace^72^. Despite efforts in many countries towards more gender equality^73^, and indeed with females now surpassing males in areas like education in many places as noted above^62^, scholars still suggest the world remains relatively patriarchal. As one analysis^74,75^ summarizes it, “Women often face difficulties in accessing education, employment and political participation on an equal footing with men. In many countries, existing laws and policies do not always favour women, reinforcing gender inequality and limiting their opportunities.” Such views perhaps have manifestations reflected in our data, though we must also acknowledge the exceptions. While males report doing better overall across the domains (R9), females scored higher in Japan and Nigeria, which notably were two of the four places in which females did better on mental and physical health. Additionally, other countries also subverted the trend on specific items, including Hong Kong and Tanzania for financial stability, Australia, Israel, Philippines, South Africa, Spain and the US for education, Indonesia on subjective financial wellbeing, and Australia and South Africa on housing.

### Religion/spirituality and family factors

Finally, there are two other sets of items in the GFS that are not part of VanderWeele’s six-domain flourishing framework per se (though there is some overlap), and constitute additional domains: religion/spirituality, and family factors. With religion/spirituality, we already noted that females reported considerably higher flourishing, outscoring males on 12 of 13 factors (with attendance being equal). Note that these include two items which are negatively related to flourishing—as indicated in the Tables above by “(n)”—in that scoring higher on these is considered worse from a wellbeing perspective, namely “spiritual punishment” and “religious criticism,” in which females have lower scores than males, meaning that females can be considered as doing better. This stronger showing by females on religion/spirituality reflects well-established trends in the literature, as for example summarized in the book “Why are women more religious than men?”^75^. One must again note though that these trends are not universal, with males scoring higher on more items than females in Indonesia and Spain, and scoring higher on several items in certain countries (e.g., in the UK, males were higher on religious service attendance, religious experience, and religious reading). Notwithstanding the exceptions, the overall trends have naturally garnered much speculation, such as the possibility that females are more inclined (whether through nature or nurture) towards qualities associated with greater religiosity, such as compassion^76^. Lastly, to complete the entire set of items, females were also slightly more likely to report both having ever been married (0.06; R86) and divorced (0.01; R87), and having children (0.10; R88), though again there were exceptions (e.g., with males more likely to report having children in Japan, Nigeria, Poland, Spain, Sweden, Turkey and the UK).

### Gender “other”

One final point is regarding the 0.3% of participants identified as “other” gender. While the main focus in this paper has been comparing males and females, this is of course not because the “other” category is unimportant or uninteresting. Rather, it rests entirely on methodological concerns, namely that the “other” sample size is so minute, especially in some places (indeed with zero in Egypt, India, Israel, Nigeria, Tanzania, and Turkey). As a result, we deemed the resulting data too unreliable to draw definitive or meaningful conclusions. But, to reiterate, the flourishing—or more accurately and troublingly, the apparently comparatively lower levels of it—is of great concern. Consider the US, where although the “other” category is only 1%, it constitutes 394 people, which does allow some meaningful assessment. On nearly every metric, they reported substantively worse flourishing than males or females, as reflected in the overall SFI (with “other” gender participants having an average score of 6.03, vs. 7.16 for males and 7.09 for females), as well as on all six dimensions, including happiness and life satisfaction (5.82 vs. 6.94/6.95), social relationship quality (6.13 vs. 7.17/7.21), meaning and purpose (5.54 vs. 7.13/7.19), character and virtue (7.30 vs. 7.68/7/64), self-rated health (6.15 vs. 7.09/6.92) and financial and material security (5.23 vs. 6.92/6.63). Such findings align with an extensive literature showing that people who identify as LGBTQ+ tend to have lower wellbeing across the lifespan, from youth^77^ to older adults^78^. We should note that the picture was not all bleak, and on seven items “other” participants actually reported more favourably compared to males/females, including: intimate friend (0.90 vs 0.83/0.89), community participation (0.28 vs. 0.14/0.13), helping (0.62/vs. 0.58/0.60), smoking (0.48, vs. 1.29/1.20), drinking (1.41, vs. 3.49/1.84), exercise (2.84 vs. 2.73/2.44), and faith-sharing (0.54 vs. 0.50/0.53). But these favourable results do not detract from the overall point that respondents in this category generally report having far lower levels of flourishing, which is a cause for concern that needs significant attention.

## Conclusion

Although there is a vast empirical literature exploring how males and females are faring in life relative to each other, most of the research is constrained by two issues: a relatively limited and partial conceptualization and assessment of what it means to do well; and a relatively narrow coverage of the world’s population, with a bias towards people in places that are relatively “WEIRD.” To help address these issues, this paper analysed an expansive battery of 73 items relating to flourishing in the GFS, covering over 200,000 people in 22 countries. Overall, males report slightly higher flourishing than females, though only marginally, scoring higher by just 0.02 (on a scale from 0.00 to 10.00) on the 10-item Flourish Index (featuring the five main domains of happiness and life satisfaction, social relationship quality, meaning and purpose, character and virtue, and self-rated health), rising to 0.07 on the 12-item Secure Flourish Index (which adds a sixth domain of financial and material security). Males also report doing better overall across the 57 items and indices pertaining to VanderWeele’s framework, scoring higher on 33.5 variables. Throughout the Discussion, however, we pointed out exceptions to these trends. For a start, *females* do better if we expand our view of flourishing to encompass the additional domains not in VanderWeele’s framework, including family factors, and above all religion/spirituality (on which females report doing better on 12 out of 13 items). Moreover, when we break the SFI into its six domains, females score higher on three (happiness and satisfaction, social relationship quality, and meaning and purpose), and males on two (self-rated health, and financial and material security), and with character/virtue being equal. The reason males score slightly higher overall on the SFI though is that females are only marginally higher on their three domains, whereas the gaps on the two led by males are much bigger. Then, above all, there is considerable country-level variation, not only on all the individual items, but flourishing overall, with females scoring higher on the SFI in nine (Australia, Egypt, India, Indonesia, Japan, Nigeria, Philippines, Poland, and Tanzania) out of the 22 countries.

More research will be needed of course, both to further understand the general trends, but also to explain the exceptions. It was striking, for instance, that in terms of physical and mental health, while males report doing better in 18 countries, in the Philippines, Indonesia, Japan, and Nigeria, females score higher. Seeking to interpret this finding, one plausible idea that occurred to us was that these countries might do relatively well in terms of gender equality; but while Philippines does indeed score highly, the others are ranked relatively low on equality. So, that does not seem like a viable explanation for females in these countries bucking the overall trend in relation to health, and other interpretations must be explored through further research. Such scholarship can ideally also help to redress the limitations of the present study. One is that, despite the GFS itself being longitudinal, the analysis here is just a cross-sectional assessment of the Wave 1 data, and it will be interesting to explore trends and causal patterns over time (which indeed future waves of the GFS will allow). Another issue is that the GFS does not allow one to delve into the important nuances around gender identity, especially in terms of transgender and nonbinary people whose identity differs from their biological sex. It is increasingly appreciated that such people face particular barriers to wellbeing and have specific needs that demand further attention and study^79^. While Gallup is piloting ways to be more sensitive and inclusive towards such populations^7–9^, these considerations have not yet been incorporated into the GWP, and hence nor into the GFS, which uses the existing methodology from the GWP that identifies such people’s gender simply as “other.” While there were also good reasons for retaining this item wording/framing, finding better ways to incorporate nuances around gender identity into international surveys like this will be an important aspect of future research.

A final limitation and caveat pertains to the topic at the heart of the paper itself, namely potential gender-related differences in self-reporting. We noted above that there may be sex differences in the way people report wellbeing related outcomes, with concern in particular that males may be liable to downplay or conceal mental health issues—due in part to masculine norms such as toughness and stoicism—leading to underdiagnoses^6,27^. The potential dynamics here are complicated though, since other sex-related biases have also been observed in relation to self-reporting, such as suggestions that females are more prone towards social desirability responding^80^. As ever, more work is needed to better understand these methodological issues. But even as it stands, without knowing exactly how such biases may have influenced the results, we must nevertheless acknowledge that our data only reflect what people *report* their wellbeing to be, rather than what it actually “is” in some objective sense. There is of course still a presumption in survey research that self-reports do provide at least *some* index of what is really happening in people’s lives. Even so, it should always be borne in mind that some observed differences may reflect reporting tendencies rather than underlying wellbeing disparities. Such methodological issues aside though, exploring the impact of sex differences on wellbeing remains an important endeavour, and this paper will hopefully provide the impetus for further work on this key topic.

## Supplementary Information

Below is the link to the electronic supplementary material.

Supplementary Material 1

## Data Availability

Data that support the findings of this article (Wave 1 non-sensitive Global data) are openly available on the Open Science Framework ([https://www.cos.io/gfs-access-data]), being available from February 2024 - March 2026 via preregistration and publicly from then onwards. Subsequent waves of the GFS will similarly be made available.

## Code availability

Code in multiple software is openly available in an online repository^49^ for the demographic variation and childhood predictor analyses (10.17605/osf.io/vbype).
