# Supplementary material for: A cross-sectional analysis of male versus female flourishing among 202,898 participants across 22 countries on 73 variables in the global flourishing study
Source: Sci Rep. 2026 Feb 22;16:10166. doi: 10.1038/s41598-026-40963-z (PMC13021907; doi:10.1038/s41598-026-40963-z)
Supplement: Supplementary file 1 — Supplementary Material 1 [file 41598_2026_40963_MOESM1_ESM.pdf]

# GFS male versus female flourishing: Supplementary File

## Contents

Notes and caveats.....3
Primary Analysis Topics and Preregistrations.....3
Country-Specific Tables.....6
Table S1a. Nationally representative descriptive statistics for Argentina.....6
Table S1b. Descriptive statistics of outcome variables for Argentina.....8
Table S1c. Demographic variation across outcomes for Argentina.....13
Table S2a. Nationally representative descriptive statistics for Australia.....15
Table S2b. Descriptive statistics of outcome variables for Australia.....17
Table S2c. Demographic variation across outcomes for Australia.....22
Table S3a. Nationally representative descriptive statistics for Brazil.....24
Table S3b. Descriptive statistics of outcome variables for Brazil.....26
Table S3c. Demographic variation across outcomes for Brazil.....31
Table S4a. Nationally representative descriptive statistics for Egypt.....33
Table S4b. Descriptive statistics of outcome variables for Egypt.....35
Table S4c. Demographic variation across outcomes for Egypt.....40
Table S5a. Nationally representative descriptive statistics for Germany.....42
Table S5b. Descriptive statistics of outcome variables for Germany.....44
Table S5c. Demographic variation across outcomes for Germany.....49
Table S6a. Nationally representative descriptive statistics for Hong Kong.....51
Table S6b. Descriptive statistics of outcome variables for Hong Kong.....53
Table S6c. Demographic variation across outcomes for Hong Kong.....58
Table S7a. Nationally representative descriptive statistics for India.....60
Table S7b. Descriptive statistics of outcome variables for India.....62
Table S7c. Demographic variation across outcomes for India.....67
Table S8a. Nationally representative descriptive statistics for Indonesia.....69
Table S8b. Descriptive statistics of outcome variables for Indonesia.....71
Table S8c. Demographic variation across outcomes for Indonesia.....76
Table S9a. Nationally representative descriptive statistics for Israel.....78
Table S9b. Descriptive statistics of outcome variables for Israel.....80
Table S9c. Demographic variation across outcomes for Israel.....85
Table S10a. Nationally representative descriptive statistics for Japan.....87
Table S10b. Descriptive statistics of outcome variables for Japan.....89
Table S10c. Demographic variation across outcomes for Japan.....94
Table S11a. Nationally representative descriptive statistics for Kenya.....96
Table S11b. Descriptive statistics of outcome variables for Kenya.....98
Table S11c. Demographic variation across outcomes for Kenya.....103
Table S12a. Nationally representative descriptive statistics for Mexico.....105
Table S12b. Descriptive statistics of outcome variables for Mexico.....107
Table S12c. Demographic variation across outcomes for Mexico.....112
Table S13a. Nationally representative descriptive statistics for Nigeria.....114

Table S13b. Descriptive statistics of outcome variables for Nigeria 116
Table S13c. Demographic variation across outcomes for Nigeria 121
Table S14a. Nationally representative descriptive statistics for Philippines 123
Table S14b. Descriptive statistics of outcome variables for Philippines 125
Table S14c. Demographic variation across outcomes for Philippines 130
Table S15a. Nationally representative descriptive statistics for Poland 132
Table S15b. Descriptive statistics of outcome variables for Poland 134
Table S15c. Demographic variation across outcomes for Poland 139
Table S16a. Nationally representative descriptive statistics for South Africa 141
Table S16b. Descriptive statistics of outcome variables for South Africa 143
Table S16c. Demographic variation across outcomes for South Africa 148
Table S17a. Nationally representative descriptive statistics for Spain 150
Table S17b. Descriptive statistics of outcome variables for Spain 152
Table S17c. Demographic variation across outcomes for Spain 157
Table S18a. Nationally representative descriptive statistics for Sweden 159
Table S18b. Descriptive statistics of outcome variables for Sweden 161
Table S18c. Demographic variation across outcomes for Sweden 166
Table S19a. Nationally representative descriptive statistics for Tanzania 168
Table S19b. Descriptive statistics of outcome variables for Tanzania 170
Table S19c. Demographic variation across outcomes for Tanzania 175
Table S20a. Nationally representative descriptive statistics for Turkey 177
Table S20c. Demographic variation across outcomes for Turkey 184
Table S21a. Nationally representative descriptive statistics for United Kingdom 186
Table S21b. Descriptive statistics of outcome variables for United Kingdom 188
Table S21c. Demographic variation across outcomes for United Kingdom 193
Table S22a. Nationally representative descriptive statistics for United States 195
Table S22b. Descriptive statistics of outcome variables for United States 197
Table S22c. Demographic variation across outcomes for United States 202
Supplementary Text: Further Considerations of Sex and Gender 204

## Notes and caveats

This online supplement to the Global Flourishing Study paper reporting on country-specific estimates of the outcome wide results. The analyses have several important caveats to interpretation for the demographic variation analyses. For the demographic variation analyses, estimating the within country group means can be unstable when the group size is small (<1%) of the country sample size. In such cases, the uncertainty in the estimate leads to a multiple imputation adjusted degrees of freedom less than 1. This means there is not enough information to evaluate the uncertainty in the estimate. We flagged such cases with a "*". Interval estimates for the mean of continuous outcomes (ranged 0-10) were based on a Wald-type confidence interval where items were treated as continuous which could rarely lead to intervals exceeding the bounds of the observed range of values; in such cases we have truncate the limits to be within the range of observed values, and such cases are marked with a "ɫ". Comparing results across countries should be done with caution due to possible measurement non-invariance, reliability of scores, seasonality effects, differences in translation, differences in the quality of weights across countries.

## Primary Analysis Topics and Preregistrations

1. Multidimensional wellbeing
    - 1.1. Psychological wellbeing
        - 1.1.1. Happiness: https://osf.io/46vr3
        - 1.1.2. Life satisfaction: https://osf.io/b8z59
        - 1.1.3. Life evaluation today: https://osf.io/b8z59
        - 1.1.4. Life evaluation in five years: https://osf.io/b8z59
        - 1.1.5. Optimism: https://osf.io/wdsx2
        - 1.1.6. Freedom
        - 1.1.7. Peace: https://osf.io/yf6s3
        - 1.1.8. Balance in life: https://osf.io/zt84x
        - 1.1.9. Mastery: https://osf.io/wtxsk
        - 1.1.10. Meaning: https://osf.io/u3pfz
        - 1.1.11. Purpose: https://osf.io/u3pfz
        - 1.1.12. Self-rated mental health: https://osf.io/6wb2u
        - 1.1.13. Traumatic distress: https://osf.io/s7naf
        - 1.1.14. Depression: https://osf.io/rjyqm
        - 1.1.15. Anxiety: https://osf.io/rjyqm
        - 1.1.16. Suffering: https://osf.io/8rxpg
    - 1.2. Social wellbeing
        - 1.2.1. Subjective social connectedness: https://osf.io/wvzns
        - 1.2.2. Social support: https://osf.io/93rqp
        - 1.2.3. Intimate friendship: https://osf.io/93rqp
        - 1.2.4. Government approval: https://osf.io/nrc52
        - 1.2.5. Political voice: https://osf.io/kwa9n
        - 1.2.6. Belonging: https://osf.io/x6qgf
        - 1.2.7. City satisfaction: https://osf.io/7v64f
        - 1.2.8. Trust: https://osf.io/546q9

1.2.9. Community participation: https://osf.io/kx369
1.2.10. Loneliness: https://osf.io/4rcnu
1.2.11. Discrimination: https://osf.io/ua6gs
1.3. Character and prosocial behaviours
1.3.1. Promoting good: https://osf.io/cyqxh
1.3.2. Delayed Gratification: https://osf.io/927bn
1.3.3. Hope: https://osf.io/wc98k
1.3.4. Gratitude: https://osf.io/mnx4s
1.3.5. Love: https://osf.io/r7dha
1.3.6. Forgiveness: https://osf.io/zugyn
1.3.7. Charitable giving: https://osf.io/e9urj
1.3.8. Helping strangers: https://osf.io/e9urj
1.3.9. Volunteering: https://osf.io/6nds5
1.4. Physical health and health behaviours
1.4.1. Self-rated physical health: https://osf.io/2z356
1.4.2. Health limitations: https://osf.io/uk9ay
1.4.3. Pain: https://osf.io/ewyr5
1.4.4. Smoking: https://osf.io/9d85z
1.4.5. Drinking: https://osf.io/hkb92
1.4.6. Exercise: https://osf.io/zvx2q
1.5. Socioeconomic factors
1.5.1. Financial and material worry: https://osf.io/g64qs
1.5.2. Education: https://osf.io/p479h
1.5.3. Employment
1.5.4. Subjective financial wellbeing: https://osf.io/9m4d6
1.5.5. Housing (owning vs. renting)
2. Religion/spirituality
2.1. Self-reported religion/spirituality: https://osf.io/taey8
2.2. Religious service attendance: https://osf.io/35xfv
2.3. Life after death belief: https://osf.io/6qyzh
2.4. Religious experience
2.5. Religious reading: https://osf.io/zf6hg
2.6. Prayer/meditation: https://osf.io/jdq35
2.7. Belief in God: https://osf.io/9486t
2.8. Intrinsic religiosity: https://osf.io/ck6uy
2.9. Religious comfort: https://osf.io/b3kg8
2.10. Loved by God: https://osf.io/djxyr
2.11. Spiritual punishment: https://osf.io/djxyr
2.12. Religious criticism: https://osf.io/rje6c
2.13. Evangelism: https://osf.io/8edcx
3. Personality traits
3.1. Extraversion
3.2. Openness to experience
3.3. Agreeableness

    3.4. Conscientiousness
    3.5. Neuroticism
4. Family factors
    4.1. Marital status: https://osf.io/wterx
    4.2. Ever divorced: https://osf.io/wterx
    4.3. Number of Children

## Country-Specific Tables

**Table S1a. Nationally representative descriptive statistics for Argentina**

| Characteristic | N = 6,724[1] |
|---|---|
| **Age group** | |
| 18-24 | 1,108 (16%) |
| 25-29 | 719 (11%) |
| 30-39 | 1,432 (21%) |
| 40-49 | 1,254 (19%) |
| 50-59 | 1,014 (15%) |
| 60-69 | 730 (11%) |
| 70-79 | 356 (5.3%) |
| 80 or older | 112 (1.7%) |
| (Missing) | 0 (0%) |
| **Gender** | |
| Male | 3,143 (47%) |
| Female | 3,542 (53%) |
| Other | 21 (0.3%) |
| (Missing) | 18 (0.3%) |
| **Marital status** | |
| Married | 1,565 (23%) |
| Separated | 455 (6.8%) |
| Divorced | 321 (4.8%) |
| Widowed | 401 (6.0%) |
| Single, never married | 2,381 (35%) |
| Domestic Partner | 1,514 (23%) |
| (Missing) | 88 (1.3%) |
| **Employment** | |
| Employed for an employer | 2,440 (36%) |
| Self-employed | 1,748 (26%) |
| Retired | 773 (11%) |
| Student | 354 (5.3%) |
| Homemaker | 639 (9.5%) |
| Unemployed and looking for a job | 569 (8.5%) |
| None of these/Other | 179 (2.7%) |
| (Missing) | 22 (0.3%) |
| **Religious service attendance** | |
| More than 1/week | 532 (7.9%) |
| 1/week | 773 (12%) |
| 1-3/month | 461 (6.8%) |
| A few times a year | 1,949 (29%) |
| Never | 2,982 (44%) |
| (Missing) | 27 (0.4%) |
| **Education** | |
| Up to 8 years | 2,263 (34%) |
| 9-15 years | 3,823 (57%) |
| 16+ years | 635 (9.4%) |
| (Missing) | 3 (<0.1%) |
| **Immigration** | |
| Born in this country | 6,346 (94%) |
| Born in another country | 348 (5.2%) |
| (Missing) | 29 (0.4%) |
| **Religious affiliation** | |
| Christianity | 4,992 (74%) |
| Islam | 9 (0.1%) |
| Hinduism | 6 (<0.1%) |
| Buddhism | 35 (0.5%) |
| Judaism | 40 (0.6%) |
| Sikhism | 0 (<0.1%) |
| Baha'i | 0 (0%) |
| Jainism | 0 (0%) |
| Shinto | 0 (0%) |
| Taoism | 2 (<0.1%) |
| Confucianism | 0 (<0.1%) |
| Primal, Animist, or Folk religion | 19 (0.3%) |
| Spiritism | 0 (0%) |
| Umbanda, Candomble, and other African-derived religions | 0 (0%) |
| Chinese folk/traditional religion | 0 (0%) |
| Some other religion | 156 (2.3%) |
| No religion/Atheist/Agnostic | 1,352 (20%) |
| (Missing) | 111 (1.7%) |

| **Characteristic** | **N = 6,724**[1] |
| --- | --- |
| **Race/Ethnicity** | |
| (Missing) | 1,070 (16%) |
| Asian | 43 (0.6%) |
| Black | 95 (1.4%) |
| Indigenous | 129 (1.9%) |
| Mestizo(a) | 1,801 (27%) |
| Mullato(a) | 75 (1.1%) |
| Other | 104 (1.5%) |
| White | 3,406 (51%) |

[1]n (%)

**Table S1b. Descriptive statistics of outcome variables for Argentina**

| Characteristic | N = 6,724[1] |
|---|---|
| **Flourishing Index** | 7.79 (1.49) |
| (Missing) | 193 |
| **Secure Flourishing Index** | 7.13 (1.47) |
| (Missing) | 209 |
| **Happiness & Life Satisfaction** | 7.29 (2.10) |
| (Missing) | 34 |
| **Social Relationship Quality** | 7.81 (2.19) |
| (Missing) | 39 |
| **Meaning and Purpose** | 7.92 (1.97) |
| (Missing) | 42 |
| **Character & Virtue** | 8.37 (1.56) |
| (Missing) | 70 |
| **Self-Rated Health** | 7.56 (1.86) |
| (Missing) | 23 |
| **Financial and Material Worry** | 3.9 (3.5) |
| (Missing) | 22 |
| **Happiness** | 7.36 (2.14) |
| (Missing) | 17 |
| **Life Satisfaction** | 7.22 (2.38) |
| (Missing) | 18 |
| **Present Life Evaluation** | 6.75 (2.19) |
| (Missing) | 7 |
| **Future Life Evaluation** | 8.03 (2.14) |
| (Missing) | 145 |
| **Optimism** | 8.86 (1.82) |
| (Missing) | 20 |
| **Freedom** | 8.29 (2.23) |
| (Missing) | 22 |
| **Peace** | |
| Always | 1,719 (26%) |
| Often | 2,808 (42%) |
| Rarely | 1,759 (26%) |
| Never | 426 (6.3%) |
| (Missing) | 13 (0.2%) |
| **Balance in Life** | |
| Always | 1,407 (21%) |
| Often | 3,441 (51%) |
| Rarely | 1,605 (24%) |
| Never | 247 (3.7%) |
| (Missing) | 24 (0.4%) |
| **Mastery** | |
| Always | 2,784 (41%) |
| Often | 3,100 (46%) |
| Rarely | 675 (10%) |
| Never | 117 (1.7%) |
| (Missing) | 48 (0.7%) |
| **Meaning** | 7.92 (2.22) |
| (Missing) | 15 |
| **Purpose** | 7.92 (2.32) |
| (Missing) | 28 |
| **Self-Rated Mental Health** | 7.87 (2.16) |
| (Missing) | 20 |
| **Content with My Relationships** | 8.05 (2.37) |
| (Missing) | 14 |
| **Satisfying Relationships** | 7.57 (2.45) |
| (Missing) | 30 |
| **Social Support** | 8.20 (2.54) |
| (Missing) | 14 |
| **Intimate Friend** | |
| Yes | 5,421 (81%) |
| No | 1,267 (19%) |
| (Missing) | 36 (0.5%) |
| **Government Approval** | |
| Strongly approve | 386 (5.7%) |
| Somewhat approve | 1,300 (19%) |
| Neither approve nor disapprove | 1,457 (22%) |
| Somewhat disapprove | 1,195 (18%) |
| Strongly disapprove | 2,311 (34%) |

| Characteristic | N = 6,724[1] |
|---|---|
| (Missing) | 74 (1.1%) |
| **Political Voice** | |
| Agree | 2,517 (37%) |
| Disagree | 2,183 (32%) |
| Unsure | 1,966 (29%) |
| (Missing) | 58 (0.9%) |
| **Belonging** | 8.08 (2.52) |
| (Missing) | 77 |
| **City Satisfaction** | |
| Satisfied | 4,770 (71%) |
| Dissatisfied | 1,200 (18%) |
| Unsure | 707 (11%) |
| (Missing) | 47 (0.7%) |
| **Trust** | |
| All | 133 (2.0%) |
| Most | 768 (11%) |
| Some | 2,783 (41%) |
| Not very many | 2,416 (36%) |
| None | 555 (8.3%) |
| (Missing) | 69 (1.0%) |
| **Community Participation** | |
| More than once a week | 734 (11%) |
| Once a week | 555 (8.3%) |
| One to three times a month | 489 (7.3%) |
| A few times a year | 1,581 (24%) |
| Never | 3,333 (50%) |
| (Missing) | 32 (0.5%) |
| **Traumatic Distress** | |
| A lot | 1,131 (17%) |
| Some | 1,434 (21%) |
| Not very much | 1,641 (24%) |
| None at all | 2,497 (37%) |
| (Missing) | 21 (0.3%) |
| **Suffering** | |
| A lot | 915 (14%) |
| Some | 2,051 (30%) |
| Not very much | 2,055 (31%) |
| None at all | 1,670 (25%) |
| (Missing) | 33 (0.5%) |
| **Loneliness** | 3.6 (3.3) |
| (Missing) | 3 |
| **Discrimination** | |
| Always | 530 (7.9%) |
| Often | 1,078 (16%) |
| Rarely | 2,238 (33%) |
| Never | 2,853 (42%) |
| (Missing) | 25 (0.4%) |
| **Promoting Good** | 8.63 (1.64) |
| (Missing) | 18 |
| **Delayed Gratification** | 8.11 (2.11) |
| (Missing) | 62 |
| **Hope** | 8.92 (1.67) |
| (Missing) | 17 |
| **Gratitude** | 8.53 (1.99) |
| (Missing) | 12 |
| **Love** | 8.50 (2.13) |
| (Missing) | 14 |
| **Forgiveness** | |
| Always | 2,446 (36%) |
| Often | 2,511 (37%) |
| Rarely | 1,329 (20%) |
| Never | 409 (6.1%) |
| (Missing) | 30 (0.4%) |
| **Charitable Giving** | |
| Yes | 1,352 (20%) |
| No | 5,357 (80%) |
| (Missing) | 15 (0.2%) |
| **Helping** | |
| Yes | 4,479 (67%) |

| Characteristic | N = 6,724[1] |
|---|---|
| No | 2,206 (33%) |
| (Missing) | 39 (0.6%) |
| **Volunteering** | |
| Yes | 1,405 (21%) |
| No | 5,310 (79%) |
| (Missing) | 9 (0.1%) |
| **Self-Rated Physical Health** | 7.26 (2.19) |
| (Missing) | 5 |
| **Health Limitations** | |
| Yes | 1,245 (19%) |
| No | 5,378 (80%) |
| (Missing) | 101 (1.5%) |
| **Pain** | |
| A lot | 1,018 (15%) |
| Some | 1,940 (29%) |
| Not very much | 2,135 (32%) |
| None at all | 1,617 (24%) |
| (Missing) | 14 (0.2%) |
| **Smoking** | 4 (8) |
| (Missing) | 109 |
| **Drinking** | 1.52 (3.55) |
| (Missing) | 102 |
| **Exercise** | |
| 0 days | 3,027 (45%) |
| 1 day | 644 (9.6%) |
| 2 days | 624 (9.3%) |
| 3 days | 876 (13%) |
| 4 days | 352 (5.2%) |
| 5 days | 425 (6.3%) |
| 6 days | 127 (1.9%) |
| 7 days/Every day | 613 (9.1%) |
| (Missing) | 34 (0.5%) |
| **Financial Stability** | 4.0 (3.7) |
| (Missing) | 15 |
| **Material Stability** | 3.8 (3.7) |
| (Missing) | 8 |
| **Education** | |
| Up to 8 years | 2,263 (34%) |
| 9-15 years | 3,823 (57%) |
| 16+ years | 635 (9.4%) |
| (Missing) | 3 (<0.1%) |
| **Employment** | |
| Employed for an employer | 2,440 (36%) |
| Self-employed | 1,748 (26%) |
| Retired | 773 (11%) |
| Student | 354 (5.3%) |
| Homemaker | 639 (9.5%) |
| Unemployed and looking for a job | 569 (8.5%) |
| None of these/Other | 179 (2.7%) |
| (Missing) | 22 (0.3%) |
| **Subjective Financial Well-Being** | |
| Living comfortably on present income | 804 (12%) |
| Getting by on present income | 2,354 (35%) |
| Finding it difficult on present income | 2,263 (34%) |
| Finding it very difficult on present income | 1,140 (17%) |
| (Missing) | 163 (2.4%) |
| **Housing** | |
| Someone in this household OWNS this home | 3,563 (53%) |
| Someone in this household RENTS this home | 1,121 (17%) |
| Both | 319 (4.7%) |
| Neither | 1,565 (23%) |
| Rent | 0 (0%) |
| Own | 0 (0%) |
| Something else | 0 (0%) |
| (Missing) | 157 (2.3%) |
| **Self-Reported Religion/Spirituality** | |
| Always | 2,224 (33%) |
| Often | 1,772 (26%) |
| Rarely | 1,725 (26%) |

| Characteristic | N = 6,724[1] |
|---|---|
| Never | 989 (15%) |
| (Missing) | 15 (0.2%) |
| **Religious Service Attendance** | |
| More than once a week | 532 (7.9%) |
| Once a week | 773 (12%) |
| One to three times a month | 461 (6.8%) |
| A few times a year | 1,949 (29%) |
| Never | 2,982 (44%) |
| (Missing) | 27 (0.4%) |
| **Life after Death Belief** | |
| Yes | 3,866 (58%) |
| No | 1,033 (15%) |
| Unsure | 1,766 (26%) |
| (Missing) | 59 (0.9%) |
| **Religious Experience** | |
| Yes | 2,682 (40%) |
| No | 4,013 (60%) |
| (Missing) | 29 (0.4%) |
| **Religious Reading** | |
| More than once a day | 631 (9.4%) |
| About once a day | 720 (11%) |
| Sometimes | 2,936 (44%) |
| Never | 2,389 (36%) |
| (Missing) | 47 (0.7%) |
| **Prayer-Meditation** | |
| More than once a day | 1,101 (16%) |
| About once a day | 1,408 (21%) |
| Sometimes | 2,649 (39%) |
| Never | 1,538 (23%) |
| (Missing) | 28 (0.4%) |
| **Belief in God** | |
| One God | 4,770 (71%) |
| More than one god | 159 (2.4%) |
| An impersonal spiritual force | 730 (11%) |
| None of these | 533 (7.9%) |
| Unsure | 509 (7.6%) |
| (Missing) | 23 (0.3%) |
| **Intrinsic Religiosity** | |
| Agree | 3,071 (46%) |
| Disagree | 737 (11%) |
| Not relevant | 1,569 (23%) |
| Unsure | 1,281 (19%) |
| (Missing) | 65 (1.0%) |
| **Religious Comfort** | |
| Agree | 3,884 (58%) |
| Disagree | 685 (10%) |
| Not relevant | 1,230 (18%) |
| Unsure | 887 (13%) |
| (Missing) | 38 (0.6%) |
| **Loved by God** | |
| Agree | 4,539 (68%) |
| Disagree | 477 (7.1%) |
| Not relevant | 966 (14%) |
| Unsure | 708 (11%) |
| (Missing) | 34 (0.5%) |
| **Spiritual Punishment** | |
| Agree | 922 (14%) |
| Disagree | 3,427 (51%) |
| Not relevant | 1,152 (17%) |
| Unsure | 1,176 (17%) |
| (Missing) | 46 (0.7%) |
| **Religious Criticism** | |
| Agree | 973 (14%) |
| Disagree | 2,132 (32%) |
| Not relevant | 1,893 (28%) |
| Unsure | 1,653 (25%) |
| (Missing) | 71 (1.1%) |
| **Evangelism** | |
| Agree | 3,323 (49%) |

| Characteristic | N = 6,724[1] |
|---|---|
| Disagree | 1,177 (18%) |
| Not relevant | 1,515 (23%) |
| Unsure | 670 (10.0%) |
| (Missing) | 39 (0.6%) |
| **Children** | 1.06 (1.48) |
| (Missing) | 97 |

[1]Mean (SD); n (%)

**Table S1c. Demographic variation across outcomes for Argentina**

| Outcome | Male | Female | Other | Male vs Female p-value | Global p-value |
|---|---|---|---|---|---|
| *Flourishing Index and Domains* | | | | | |
| Flourishing Index | 7.80 (7.73,7.88) | 7.78 (7.71,7.85) | 6.71 (6.20,7.22) | 2.62e-02 | 2.98e-05 |
| Secure Flourishing Index | 7.25 (7.17,7.32) | 7.05 (6.98,7.11) | 6.07 (5.70,6.44) | 2.56e-05 | 3.22e-12 |
| Happiness & Life Satisfaction | 7.32 (7.21,7.43) | 7.26 (7.16,7.36) | 6.46 (4.94,7.98) | 8.28e-03 | 3.63e-01 |
| Social Relationship Quality | 7.78 (7.67,7.89) | 7.84 (7.73,7.95) | 5.91 (4.74,7.07) | 9.78e-01 | 1.88e-03 |
| Meaning and Purpose | 7.86 (7.76,7.97) | 7.97 (7.88,8.06) | 6.91 (5.74,8.08) | 5.41e-01 | 5.42e-02 |
| Character & Virtue | 8.29 (8.21,8.37) | 8.44 (8.37,8.51) | 8.27 (7.41,9.13) | 4.72e-01 | 1.87e-02 |
| Self-Rated Health | 7.76 (7.67,7.85) | 7.40 (7.31,7.50) | 6.00 (5.39,6.61) | 5.03e-09 | 2.12e-13 |
| Financial and Material Worry | 4.46 (4.28,4.63) | 3.36 (3.20,3.51) | 2.88 (1.25,4.50) | 4.29e-09 | 1.6e-16 |
| *Psychological Well-Being* | | | | | |
| Happiness | 7.37 (7.26,7.49) | 7.35 (7.25,7.45) | 6.77 (5.00,8.54) | 3.04e-02 | 7.27e-01 |
| Life Satisfaction | 7.27 (7.15,7.39) | 7.17 (7.06,7.29) | 6.15 (4.75,7.56) | 7.77e-03 | 1.41e-01 |
| Present Life Evaluation | 6.75 (6.64,6.86) | 6.75 (6.64,6.86) | 5.78 (4.65,6.91) | 1.81e-01 | 1.86e-01 |
| Future Life Evaluation | 7.86 (7.74,7.98) | 8.11 (8.01,8.22) | 7.34 (5.79,8.88) | 4.03e-01 | 3.41e-03 |
| Optimism | 8.65 (8.55,8.75) | 9.04 (8.96,9.12) | 9.11 (8.23,9.99) | 1.16e-03 | 7.91e-09 |
| Freedom | 8.27 (8.16,8.37) | 8.30 (8.20,8.41) | 8.36 (7.33,9.39) | 3.28e-01 | 8.56e-01 |
| Peace | 0.68 (0.66,0.71) | 0.67 (0.65,0.69) | 0.30 (0.05,0.54) | 9.79e-01 | 2.04e-03 |
| Balance in Life | 0.74 (0.72,0.77) | 0.71 (0.69,0.73) | 0.78 (0.55,1.00) | 1.95e-03 | 4.53e-02 |
| Mastery | 0.90 (0.88,0.91) | 0.87 (0.85,0.88) | 0.71 (0.38,1.04) | 2.23e-03 | 3.31e-02 |
| Meaning | 7.82 (7.71,7.94) | 8.01 (7.90,8.11) | 7.17 (5.84,8.50) | 8.59e-01 | 3.12e-02 |
| Purpose | 7.91 (7.79,8.02) | 7.94 (7.83,8.04) | 6.65 (5.36,7.95) | 3.86e-01 | 1.05e-01 |
| Self-Rated Mental Health | 8.04 (7.94,8.15) | 7.74 (7.63,7.85) | 5.75 (4.61,6.89) | 6.28e-06 | 1.67e-07 |
| *Social Well-Being* | | | | | |
| Content with My Relationships | 8.02 (7.90,8.14) | 8.08 (7.97,8.20) | 5.37 (3.12,7.62) | 8.6e-01 | 2.99e-02 |
| Satisfying Relationships | 7.55 (7.42,7.67) | 7.60 (7.48,7.71) | 6.44 (5.69,7.19) | 8.41e-01 | 4.69e-03 |
| Social Support | 8.04 (7.90,8.18) | 8.35 (8.24,8.47) | 8.24 (6.81,9.67) | 2.83e-02 | 2.96e-03 |
| Intimate Friend | 0.79 (0.77,0.81) | 0.83 (0.81,0.85) | 0.57 (0.24,0.89) | 9.18e-02 | 3.39e-03 |
| Government Approval | 0.27 (0.25,0.29) | 0.24 (0.22,0.26) | 0.10 (0.00,0.25) | 9.17e-02 | 9.48e-03 |
| Political Voice | 0.53 (0.50,0.56) | 0.51 (0.49,0.53) | 0.50 (0.18,0.82) | 3.31e-01 | 4.65e-01 |
| Belonging | 8.08 (7.95,8.21) | 8.11 (7.99,8.23) | 5.42 (2.80,8.05) | 2.6e-01 | 8.35e-02 |
| City Satisfaction | 0.78 (0.76,0.80) | 0.81 (0.79,0.82) | 0.57 (0.14,1.00) | 1.46e-01 | 3.49e-02 |
| Trust | 0.16 (0.15,0.18) | 0.11 (0.10,0.12) | 0.05 (0.00,0.12) | 2.95e-05 | 4.45e-06 |
| Community Participation | 0.22 (0.20,0.24) | 0.17 (0.15,0.18) | 0.35 (0.04,0.66) | 9.26e-04 | 6.9e-05 |
| *Psychological Distress* | | | | | |
| Traumatic Distress | 0.33 (0.30,0.35) | 0.43 (0.41,0.45) | 0.30 (0.00,0.60) | 4.35e-07 | 3.03e-09 |
| Depression Symptoms | 0.32 (0.30,0.35) | 0.39 (0.37,0.41) | 0.57 (0.27,0.87) | 4.29e-04 | 8.01e-05 |
| Anxiety Symptoms | 0.35 (0.33,0.38) | 0.45 (0.43,0.48) | 0.29 (0.05,0.54) | 4.17e-05 | 2.3e-08 |
| Suffering | 0.40 (0.38,0.42) | 0.48 (0.46,0.51) | 0.50 (0.18,0.82) | 9.73e-05 | 1.05e-05 |
| *Social Distress* | | | | | |
| Loneliness | 3.36 (3.19,3.52) | 3.73 (3.57,3.88) | 5.33 (3.07,7.59) | 1.12e-02 | 1.23e-03 |
| Discrimination | 0.25 (0.22,0.27) | 0.23 (0.21,0.25) | 0.42 (0.11,0.74) | 5.22e-01 | 2.88e-01 |
| *Character & Prosocial Behavior* | | | | | |
| Promoting Good | 8.51 (8.42,8.60) | 8.73 (8.66,8.80) | 8.44 (7.60,9.29) | 1.13e-01 | 8.28e-04 |
| Delayed Gratification | 8.07 (7.97,8.17) | 8.16 (8.06,8.25) | 8.09 (7.19,9.00) | 8.6e-01 | 4.83e-01 |
| Hope | 8.77 (8.68,8.86) | 9.06 (8.98,9.13) | 8.37 (6.38,10.36) | 1.51e-01 | 7.63e-06 |
| Gratitude | 8.31 (8.20,8.42) | 8.73 (8.65,8.82) | 8.98 (8.02,9.94) | 2e-02 | 6.01e-09 |
| Love | 8.17 (8.05,8.29) | 8.80 (8.71,8.89) | 8.62 (7.64,9.60) | 5.9e-08 | 1.55e-15 |
| Forgiveness | 0.71 (0.69,0.74) | 0.77 (0.75,0.78) | 0.88 (0.71,1.04) | 6.49e-02 | 4.31e-04 |
| Charitable Giving | 0.20 (0.18,0.22) | 0.21 (0.19,0.22) | 0.08 (0.00,0.18) | 4.59e-01 | 2.17e-02 |
| Helping | 0.67 (0.64,0.69) | 0.67 (0.65,0.70) | 0.80 (0.57,1.03) | 8.1e-01 | 4.13e-01 |
| Volunteering | 0.21 (0.19,0.23) | 0.21 (0.19,0.23) | 0.20 (0.01,0.40) | 2.41e-01 | 9.62e-01 |
| *Physical Health & Health Behavior* | | | | | |
| Self-Rated Physical Health | 7.47 (7.37,7.57) | 7.07 (6.95,7.18) | 6.25 (5.41,7.08) | 1.16e-08 | 4.75e-08 |
| Health Limitations | 0.16 (0.14,0.18) | 0.21 (0.19,0.23) | 0.61 (0.34,0.89) | 4.28e-03 | 2.12e-05 |
| Pain | 0.38 (0.36,0.41) | 0.49 (0.47,0.52) | 0.49 (0.17,0.81) | 2.52e-07 | 1.87e-10 |
| Smoking | 4.37 (3.95,4.79) | 3.25 (2.92,3.57) | 2.27 (0.00,5.49) | 3.78e-03 | 9.36e-05 |
| Drinking | 2.07 (1.90,2.25) | 0.97 (0.86,1.08) | 2.37 (0.00,5.27) | 1.27e-12 | 1.6e-16 |
| Exercise | 2.23 (2.11,2.35) | 1.72 (1.62,1.82) | 1.87 (0.70,3.04) | 5.46e-05 | 1.25e-09 |
| *Socioeconomic Outcomes* | | | | | |
| Financial Stability | 4.54 (4.35,4.72) | 3.45 (3.28,3.61) | 2.81 (1.06,4.55) | 7.39e-08 | 1.6e-16 |
| Material Stability | 4.38 (4.19,4.57) | 3.27 (3.10,3.43) | 2.95 (1.40,4.50) | 1.31e-08 | 1.6e-16 |
| Education | 0.10 (0.09,0.11) | 0.09 (0.08,0.10) | 0.07 (0.00,0.15) | 4.04e-01 | 4.32e-01 |
| Employment | 0.74 (0.72,0.77) | 0.52 (0.50,0.54) | 0.29 (0.05,0.53) | 1.6e-16 | 1.6e-16 |
| Subjective Financial Well-Being | 0.53 (0.51,0.56) | 0.44 (0.41,0.46) | 0.31 (0.06,0.56) | 3.81e-06 | 3.13e-08 |
| Housing | 0.60 (0.57,0.62) | 0.58 (0.55,0.60) | 0.55 (0.22,0.88) | 3.56e-01 | 3.62e-01 |
| *Religion/Spirituality* | | | | | |
| Self-Reported Religion/Spirituality | 0.54 (0.52,0.57) | 0.64 (0.62,0.66) | 0.64 (0.36,0.91) | 3.86e-05 | 1.03e-08 |

| Outcome | Male | Female | Other | Male vs Female p-value | Global p-value |
|---|---|---|---|---|---|
| Religious Service Attendance | 0.17 (0.15,0.19) | 0.22 (0.20,0.24) | 0.03 (0.00,0.08) | 2.37e-01 | 2.24e-11 |
| Life after Death Belief | 0.54 (0.51,0.56) | 0.62 (0.59,0.64) | 0.51 (0.19,0.83) | 2.43e-04 | 2.85e-05 |
| Religious Experience | 0.37 (0.35,0.40) | 0.42 (0.40,0.45) | 0.46 (0.14,0.77) | 2.62e-02 | 1.15e-02 |
| Religious Reading | 0.18 (0.16,0.20) | 0.22 (0.20,0.24) | 0.01 (0.00,0.03) | 1.73e-01 | 1.6e-16 |
| Prayer-Meditation | 0.31 (0.28,0.33) | 0.44 (0.41,0.46) | 0.28 (0.00,0.58) | 2.79e-06 | 1.15e-14 |
| Belief in God | 0.80 (0.78,0.82) | 0.88 (0.87,0.90) | 0.70 (0.45,0.95) | 1.91e-05 | 6.39e-10 |
| Intrinsic Religiosity | 0.69 (0.66,0.71) | 0.75 (0.72,0.77) | 0.35 (0.05,0.66) | 4e-02 | 1.09e-05 |
| Religious Comfort | 0.72 (0.69,0.75) | 0.81 (0.78,0.83) | 0.62 (0.22,1.01) | 1.48e-05 | 8.75e-09 |
| Loved by God | 0.77 (0.74,0.79) | 0.86 (0.84,0.88) | 0.68 (0.39,0.97) | 3.87e-06 | 1.31e-11 |
| Spiritual Punishment | 0.24 (0.21,0.27) | 0.21 (0.18,0.23) | 0.23 (0.00,0.71) | 2.56e-01 | 5.52e-02 |
| Religious Criticism | 0.31 (0.28,0.34) | 0.30 (0.27,0.32) | 0.31 (0.00,0.65) | 7.18e-01 | 7.9e-01 |
| Evangelism | 0.68 (0.65,0.70) | 0.70 (0.68,0.72) | 0.47 (0.12,0.83) | 3.18e-01 | 9.61e-02 |
| *Family Factors* | | | | | |
| Ever Married | 0.39 (0.37,0.42) | 0.43 (0.40,0.45) | 0.06 (0.00,0.14) | 4.22e-01 | 1.6e-16 |
| Divorced | 0.04 (0.03,0.05) | 0.05 (0.04,0.06) | 0.00 * | 1.45e-02 | 1.6e-16 |
| Children | 0.98 (0.90,1.06) | 1.15 (1.08,1.22) | 0.65 (0.07,1.24) | 4.1e-01 | 2.38e-03 |

**Table S2a. Nationally representative descriptive statistics for Australia**

| Characteristic | N = 3,844[1] |
|---|---|
| **Age group** | |
| 18-24 | 345 (9.0%) |
| 25-29 | 282 (7.3%) |
| 30-39 | 641 (17%) |
| 40-49 | 618 (16%) |
| 50-59 | 691 (18%) |
| 60-69 | 589 (15%) |
| 70-79 | 498 (13%) |
| 80 or older | 178 (4.6%) |
| (Missing) | 2 (<0.1%) |
| **Gender** | |
| Male | 1,861 (48%) |
| Female | 1,941 (50%) |
| Other | 36 (0.9%) |
| (Missing) | 6 (0.2%) |
| **Marital status** | |
| Married | 1,797 (47%) |
| Separated | 158 (4.1%) |
| Divorced | 332 (8.6%) |
| Widowed | 215 (5.6%) |
| Single, never married | 855 (22%) |
| Domestic Partner | 450 (12%) |
| (Missing) | 38 (1.0%) |
| **Employment** | |
| Employed for an employer | 1,881 (49%) |
| Self-employed | 380 (9.9%) |
| Retired | 912 (24%) |
| Student | 190 (5.0%) |
| Homemaker | 137 (3.6%) |
| Unemployed and looking for a job | 134 (3.5%) |
| None of these/Other | 206 (5.4%) |
| (Missing) | 4 (0.1%) |
| **Religious service attendance** | |
| More than 1/week | 162 (4.2%) |
| 1/week | 299 (7.8%) |
| 1-3/month | 135 (3.5%) |
| A few times a year | 656 (17%) |
| Never | 2,584 (67%) |
| (Missing) | 7 (0.2%) |
| **Education** | |
| Up to 8 years | 70 (1.8%) |
| 9-15 years | 2,434 (63%) |
| 16+ years | 1,330 (35%) |
| (Missing) | 10 (0.3%) |
| **Immigration** | |
| Born in this country | 2,953 (77%) |
| Born in another country | 885 (23%) |
| (Missing) | 6 (0.2%) |
| **Religious affiliation** | |
| Christianity | 1,592 (41%) |
| Islam | 45 (1.2%) |
| Hinduism | 31 (0.8%) |
| Buddhism | 36 (0.9%) |
| Judaism | 26 (0.7%) |
| Sikhism | 8 (0.2%) |
| Baha'i | 7 (0.2%) |
| Jainism | 0 (0%) |
| Shinto | 0 (0%) |
| Taoism | 5 (0.1%) |
| Confucianism | 0 (0%) |
| Primal, Animist, or Folk religion | 23 (0.6%) |
| Spiritism | 0 (0%) |
| Umbanda, Candomble, and other African-derived religions | 0 (0%) |
| Chinese folk/traditional religion | 0 (0%) |
| Some other religion | 39 (1.0%) |
| No religion/Atheist/Agnostic | 2,020 (53%) |
| (Missing) | 15 (0.4%) |
| **Race/Ethnicity** | |

| Characteristic | N = 3,844[1] |
|---|---|
| (Missing) | 14 (0.4%) |
| Aboriginal | 53 (1.4%) |
| Australian | 1,946 (51%) |
| Australian British/European | 1,047 (27%) |
| Chinese | 75 (1.9%) |
| Indian | 58 (1.5%) |
| Japanese | 1 (<0.1%) |
| Malay | 11 (0.3%) |
| New Zealander | 91 (2.4%) |
| Other | 163 (4.2%) |
| Other European | 357 (9.3%) |
| Russian | 7 (0.2%) |
| Samoan | 4 (0.1%) |
| Sinhalese | 1 (<0.1%) |
| Spanish | 2 (<0.1%) |
| Sri Lankan Moor | 1 (<0.1%) |
| Sri Lankan Tamil | 7 (0.2%) |
| Vietnamese | 7 (0.2%) |

[1]n (%)

**Table S2b. Descriptive statistics of outcome variables for Australia**

| Characteristic | N = 3,844[1] |
|---|---|
| **Flourishing Index** | 7.01 (1.59) |
| (Missing) | 35 |
| **Secure Flourishing Index** | 7.01 (1.60) |
| (Missing) | 40 |
| **Happiness & Life Satisfaction** | 6.80 (1.87) |
| (Missing) | 11 |
| **Social Relationship Quality** | 7.05 (2.30) |
| (Missing) | 9 |
| **Meaning and Purpose** | 7.04 (2.11) |
| (Missing) | 5 |
| **Character & Virtue** | 7.56 (1.50) |
| (Missing) | 10 |
| **Self-Rated Health** | 6.65 (1.88) |
| (Missing) | 8 |
| **Financial and Material Worry** | 6.98 (2.72) |
| (Missing) | 5 |
| **Happiness** | 6.88 (1.82) |
| (Missing) | 5 |
| **Life Satisfaction** | 6.72 (2.10) |
| (Missing) | 8 |
| **Present Life Evaluation** | 6.78 (1.77) |
| (Missing) | 3 |
| **Future Life Evaluation** | 7.59 (1.86) |
| (Missing) | 6 |
| **Optimism** | 7.40 (2.28) |
| (Missing) | 54 |
| **Freedom** | 7.11 (2.53) |
| **Peace** | |
| Always | 450 (12%) |
| Often | 2,533 (66%) |
| Rarely | 792 (21%) |
| Never | 59 (1.5%) |
| (Missing) | 9 (0.2%) |
| **Balance in Life** | |
| Always | 239 (6.2%) |
| Often | 2,474 (64%) |
| Rarely | 1,044 (27%) |
| Never | 81 (2.1%) |
| (Missing) | 5 (0.1%) |
| **Mastery** | |
| Always | 545 (14%) |
| Often | 2,717 (71%) |
| Rarely | 523 (14%) |
| Never | 31 (0.8%) |
| (Missing) | 27 (0.7%) |
| **Meaning** | 7.22 (2.11) |
| (Missing) | 2 |
| **Purpose** | 6.85 (2.53) |
| (Missing) | 3 |
| **Self-Rated Mental Health** | 6.81 (2.27) |
| (Missing) | 4 |
| **Content with My Relationships** | 7.25 (2.29) |
| (Missing) | 6 |
| **Satisfying Relationships** | 6.84 (2.48) |
| (Missing) | 3 |
| **Social Support** | 8.12 (2.28) |
| (Missing) | 10 |
| **Intimate Friend** | |
| Yes | 3,205 (83%) |
| No | 550 (14%) |
| (Missing) | 89 (2.3%) |
| **Government Approval** | |
| Strongly approve | 357 (9.3%) |
| Somewhat approve | 1,292 (34%) |
| Neither approve nor disapprove | 963 (25%) |
| Somewhat disapprove | 805 (21%) |
| Strongly disapprove | 409 (11%) |
| (Missing) | 17 (0.5%) |

| Characteristic | N = 3,844[1] |
| --- | --- |
| **Political Voice** | |
| Agree | 1,458 (38%) |
| Disagree | 1,568 (41%) |
| Unsure | 806 (21%) |
| (Missing) | 12 (0.3%) |
| **Belonging** | 7.79 (2.27) |
| (Missing) | 31 |
| **City Satisfaction** | |
| Satisfied | 3,160 (82%) |
| Dissatisfied | 395 (10%) |
| Unsure | 202 (5.3%) |
| (Missing) | 88 (2.3%) |
| **Trust** | |
| All | 18 (0.5%) |
| Most | 1,455 (38%) |
| Some | 1,716 (45%) |
| Not very many | 621 (16%) |
| None | 6 (0.2%) |
| (Missing) | 28 (0.7%) |
| **Community Participation** | |
| More than once a week | 573 (15%) |
| Once a week | 507 (13%) |
| One to three times a month | 482 (13%) |
| A few times a year | 904 (24%) |
| Never | 1,372 (36%) |
| (Missing) | 6 (0.2%) |
| **Traumatic Distress** | |
| A lot | 323 (8.4%) |
| Some | 829 (22%) |
| Not very much | 1,189 (31%) |
| None at all | 1,500 (39%) |
| (Missing) | 3 (<0.1%) |
| **Suffering** | |
| A lot | 479 (12%) |
| Some | 1,453 (38%) |
| Not very much | 1,350 (35%) |
| None at all | 550 (14%) |
| (Missing) | 12 (0.3%) |
| **Loneliness** | 3.33 (2.80) |
| (Missing) | 2 |
| **Discrimination** | |
| Always | 115 (3.0%) |
| Often | 570 (15%) |
| Rarely | 2,015 (52%) |
| Never | 1,138 (30%) |
| (Missing) | 4 (0.1%) |
| **Promoting Good** | 7.86 (1.61) |
| (Missing) | 6 |
| **Delayed Gratification** | 7.25 (1.94) |
| (Missing) | 5 |
| **Hope** | 7.70 (2.02) |
| (Missing) | 160 |
| **Gratitude** | 7.83 (2.23) |
| (Missing) | 156 |
| **Love** | 8.31 (1.75) |
| (Missing) | 2 |
| **Forgiveness** | |
| Always | 623 (16%) |
| Often | 2,384 (62%) |
| Rarely | 741 (19%) |
| Never | 88 (2.3%) |
| (Missing) | 8 (0.2%) |
| **Charitable Giving** | |
| Yes | 2,046 (53%) |
| No | 1,789 (47%) |
| (Missing) | 8 (0.2%) |
| **Helping** | |
| Yes | 2,350 (61%) |
| No | 1,456 (38%) |

| Characteristic | N = 3,844[1] |
|---|---|
| (Missing) | 38 (1.0%) |
| **Volunteering** | |
| Yes | 1,295 (34%) |
| No | 2,546 (66%) |
| (Missing) | 3 (<0.1%) |
| **Self-Rated Physical Health** | 6.49 (2.06) |
| (Missing) | 5 |
| **Health Limitations** | |
| Yes | 1,053 (27%) |
| No | 2,752 (72%) |
| (Missing) | 40 (1.0%) |
| **Pain** | |
| A lot | 607 (16%) |
| Some | 1,533 (40%) |
| Not very much | 1,254 (33%) |
| None at all | 443 (12%) |
| (Missing) | 7 (0.2%) |
| **Smoking** | 1.3 (4.6) |
| (Missing) | 44 |
| **Drinking** | 5 (8) |
| (Missing) | 29 |
| **Exercise** | |
| 0 days | 958 (25%) |
| 1 day | 446 (12%) |
| 2 days | 570 (15%) |
| 3 days | 567 (15%) |
| 4 days | 361 (9.4%) |
| 5 days | 377 (9.8%) |
| 6 days | 244 (6.4%) |
| 7 days/Every day | 318 (8.3%) |
| (Missing) | 3 (<0.1%) |
| **Financial Stability** | 6.69 (2.99) |
| (Missing) | 2 |
| **Material Stability** | 7.28 (2.78) |
| (Missing) | 4 |
| **Education** | |
| Up to 8 years | 70 (1.8%) |
| 9-15 years | 2,434 (63%) |
| 16+ years | 1,330 (35%) |
| (Missing) | 10 (0.3%) |
| **Employment** | |
| Employed for an employer | 1,881 (49%) |
| Self-employed | 380 (9.9%) |
| Retired | 912 (24%) |
| Student | 190 (5.0%) |
| Homemaker | 137 (3.6%) |
| Unemployed and looking for a job | 134 (3.5%) |
| None of these/Other | 206 (5.4%) |
| (Missing) | 4 (0.1%) |
| **Subjective Financial Well-Being** | |
| Living comfortably on present income | 1,789 (47%) |
| Getting by on present income | 1,396 (36%) |
| Finding it difficult on present income | 454 (12%) |
| Finding it very difficult on present income | 157 (4.1%) |
| (Missing) | 48 (1.2%) |
| **Housing** | |
| Someone in this household OWNS this home | 2,461 (64%) |
| Someone in this household RENTS this home | 962 (25%) |
| Both | 172 (4.5%) |
| Neither | 209 (5.4%) |
| Rent | 0 (0%) |
| Own | 0 (0%) |
| Something else | 0 (0%) |
| (Missing) | 39 (1.0%) |
| **Self-Reported Religion/Spirituality** | |
| Always | 616 (16%) |
| Often | 807 (21%) |
| Rarely | 1,217 (32%) |
| Never | 1,200 (31%) |

| Characteristic | N = 3,844[1] |
|---|---|
| (Missing) | 3 (<0.1%) |
| **Religious Service Attendance** | |
| More than once a week | 162 (4.2%) |
| Once a week | 299 (7.8%) |
| One to three times a month | 135 (3.5%) |
| A few times a year | 656 (17%) |
| Never | 2,584 (67%) |
| (Missing) | 7 (0.2%) |
| **Life after Death Belief** | |
| Yes | 1,390 (36%) |
| No | 1,214 (32%) |
| Unsure | 1,218 (32%) |
| (Missing) | 22 (0.6%) |
| **Religious Experience** | |
| Yes | 961 (25%) |
| No | 2,869 (75%) |
| (Missing) | 15 (0.4%) |
| **Religious Reading** | |
| More than once a day | 103 (2.7%) |
| About once a day | 295 (7.7%) |
| Sometimes | 1,039 (27%) |
| Never | 2,374 (62%) |
| (Missing) | 33 (0.8%) |
| **Prayer-Meditation** | |
| More than once a day | 321 (8.3%) |
| About once a day | 494 (13%) |
| Sometimes | 1,380 (36%) |
| Never | 1,632 (42%) |
| (Missing) | 18 (0.5%) |
| **Belief in God** | |
| One God | 1,255 (33%) |
| More than one god | 65 (1.7%) |
| An impersonal spiritual force | 740 (19%) |
| None of these | 1,175 (31%) |
| Unsure | 598 (16%) |
| (Missing) | 12 (0.3%) |
| **Intrinsic Religiosity** | |
| Agree | 984 (26%) |
| Disagree | 716 (19%) |
| Not relevant | 1,794 (47%) |
| Unsure | 337 (8.8%) |
| (Missing) | 14 (0.4%) |
| **Religious Comfort** | |
| Agree | 1,264 (33%) |
| Disagree | 520 (14%) |
| Not relevant | 1,762 (46%) |
| Unsure | 288 (7.5%) |
| (Missing) | 10 (0.3%) |
| **Loved by God** | |
| Agree | 1,119 (29%) |
| Disagree | 542 (14%) |
| Not relevant | 1,750 (46%) |
| Unsure | 422 (11%) |
| (Missing) | 11 (0.3%) |
| **Spiritual Punishment** | |
| Agree | 152 (4.0%) |
| Disagree | 1,829 (48%) |
| Not relevant | 1,614 (42%) |
| Unsure | 237 (6.2%) |
| (Missing) | 12 (0.3%) |
| **Religious Criticism** | |
| Agree | 178 (4.6%) |
| Disagree | 981 (26%) |
| Not relevant | 2,421 (63%) |
| Unsure | 253 (6.6%) |
| (Missing) | 10 (0.3%) |
| **Evangelism** | |
| Agree | 883 (23%) |
| Disagree | 854 (22%) |

| Characteristic | N = 3,844[1] |
|---|---|
| Not relevant | 1,927 (50%) |
| Unsure | 175 (4.5%) |
| (Missing) | 5 (0.1%) |
| **Children** | |
| 0 | 2,780 (72%) |
| 1 | 397 (10%) |
| 2 | 447 (12%) |
| 3 | 165 (4.3%) |
| 4 | 38 (1.0%) |
| 5 | 7 (0.2%) |
| 6 | 4 (<0.1%) |
| (Missing) | 6 |

[1]Mean (SD); n (%)

**Table S2c. Demographic variation across outcomes for Australia**

| Outcome | Male | Female | Other | Male vs Female p-value | Global p-value |
|---|---|---|---|---|---|
| *Flourishing Index and Domains* | | | | | |
| Flourishing Index | 7.01 (6.92,7.11) | 7.04 (6.95,7.14) | 5.93 (5.31,6.55) | 5.88e-01 | 1.31e-03 |
| Secure Flourishing Index | 7.02 (6.92,7.12) | 7.03 (6.93,7.12) | 5.85 (5.15,6.54) | 7.09e-01 | 2.75e-03 |
| Happiness & Life Satisfaction | 6.77 (6.66,6.88) | 6.86 (6.75,6.97) | 5.48 (4.62,6.34) | 5.24e-01 | 4e-03 |
| Social Relationship Quality | 6.98 (6.84,7.12) | 7.12 (6.98,7.26) | 6.51 (5.73,7.29) | 2.39e-01 | 1.28e-01 |
| Meaning and Purpose | 7.00 (6.87,7.13) | 7.11 (6.98,7.23) | 5.24 (4.30,6.17) | 4.12e-01 | 1.95e-04 |
| Character & Virtue | 7.58 (7.50,7.67) | 7.55 (7.46,7.64) | 6.86 (6.18,7.54) | 4.88e-01 | 9.33e-02 |
| Self-Rated Health | 6.74 (6.63,6.85) | 6.59 (6.48,6.70) | 5.55 (4.72,6.39) | 1.98e-01 | 4.49e-03 |
| Financial and Material Worry | 7.05 (6.88,7.21) | 6.95 (6.79,7.11) | 5.43 (4.03,6.83) | 7.86e-01 | 5.19e-02 |
| *Psychological Well-Being* | | | | | |
| Happiness | 6.86 (6.75,6.97) | 6.93 (6.82,7.03) | 5.66 (4.87,6.45) | 6.89e-01 | 4.24e-03 |
| Life Satisfaction | 6.68 (6.56,6.81) | 6.78 (6.66,6.91) | 5.31 (4.28,6.34) | 4.26e-01 | 1.01e-02 |
| Present Life Evaluation | 6.81 (6.71,6.91) | 6.77 (6.67,6.88) | 6.20 (5.56,6.83) | 9.01e-01 | 1.43e-01 |
| Future Life Evaluation | 7.61 (7.50,7.72) | 7.58 (7.48,7.69) | 7.16 (6.29,8.03) | 9.14e-01 | 5.66e-01 |
| Optimism | 7.29 (7.15,7.43) | 7.57 (7.44,7.70) | 5.36 (4.09,6.62) | 1.07e-02 | 5.81e-05 |
| Freedom | 7.01 (6.85,7.16) | 7.24 (7.09,7.38) | 4.90 (3.42,6.38) | 8.26e-02 | 9.54e-04 |
| Peace | 0.78 (0.75,0.80) | 0.78 (0.76,0.81) | 0.57 (0.32,0.82) | 5.05e-01 | 2.09e-01 |
| Balance in Life | 0.71 (0.68,0.73) | 0.71 (0.69,0.74) | 0.45 (0.21,0.69) | 5.04e-01 | 8.91e-02 |
| Mastery | 0.87 (0.85,0.89) | 0.85 (0.83,0.87) | 0.58 (0.33,0.83) | 5.96e-02 | 3.71e-02 |
| Meaning | 7.17 (7.04,7.30) | 7.30 (7.17,7.42) | 6.17 (5.23,7.10) | 3.53e-01 | 2.5e-02 |
| Purpose | 6.83 (6.67,6.98) | 6.92 (6.77,7.06) | 4.31 (2.76,5.86) | 5.56e-01 | 2.33e-03 |
| Self-Rated Mental Health | 6.88 (6.74,7.01) | 6.79 (6.65,6.92) | 5.12 (4.10,6.14) | 5.64e-01 | 1.88e-03 |
| *Social Well-Being* | | | | | |
| Content with My Relationships | 7.19 (7.05,7.32) | 7.33 (7.19,7.47) | 6.52 (5.68,7.35) | 2.14e-01 | 7.36e-02 |
| Satisfying Relationships | 6.77 (6.62,6.92) | 6.92 (6.77,7.07) | 6.50 (5.63,7.38) | 3.01e-01 | 2.71e-01 |
| Social Support | 7.99 (7.85,8.12) | 8.25 (8.12,8.39) | 7.70 (6.74,8.67) | 7.22e-02 | 1.67e-02 |
| Intimate Friend | 0.82 (0.80,0.85) | 0.88 (0.86,0.90) | 0.84 (0.66,1.02) | 1.31e-02 | 5.71e-04 |
| Government Approval | 0.45 (0.42,0.48) | 0.42 (0.39,0.45) | 0.10 (0.00,0.25) | 7.3e-01 | 1.93e-05 |
| Political Voice | 0.49 (0.46,0.52) | 0.48 (0.45,0.51) | 0.30 (0.07,0.53) | 6.81e-01 | 1.7e-01 |
| Belonging | 7.60 (7.47,7.74) | 8.02 (7.89,8.14) | 5.39 (4.11,6.66) | 1.16e-04 | 4.65e-08 |
| City Satisfaction | 0.87 (0.85,0.89) | 0.91 (0.89,0.92) | 0.63 (0.34,0.91) | 1.06e-02 | 3.12e-03 |
| Trust | 0.42 (0.39,0.45) | 0.36 (0.33,0.38) | 0.28 (0.05,0.51) | 4.42e-01 | 2.93e-03 |
| Community Participation | 0.27 (0.25,0.30) | 0.29 (0.27,0.32) | 0.16 (0.00,0.34) | 4.95e-01 | 2.52e-01 |
| *Psychological Distress* | | | | | |
| Traumatic Distress | 0.27 (0.24,0.29) | 0.33 (0.30,0.35) | 0.62 (0.39,0.85) | 8.01e-03 | 6.45e-05 |
| Depression Symptoms | 0.24 (0.22,0.27) | 0.24 (0.22,0.27) | 0.37 (0.13,0.60) | 2.23e-01 | 5.71e-01 |
| Anxiety Symptoms | 0.21 (0.18,0.23) | 0.25 (0.22,0.27) | 0.43 (0.19,0.68) | 1.5e-02 | 2.15e-02 |
| Suffering | 0.47 (0.44,0.50) | 0.54 (0.51,0.56) | 0.60 (0.36,0.84) | 5.39e-04 | 3.4e-03 |
| *Social Distress* | | | | | |
| Loneliness | 3.36 (3.19,3.53) | 3.28 (3.13,3.44) | 4.29 (2.93,5.65) | 2.88e-01 | 2.79e-01 |
| Discrimination | 0.16 (0.14,0.18) | 0.19 (0.17,0.21) | 0.50 (0.25,0.75) | 4.05e-01 | 6.76e-03 |
| *Character & Prosocial Behavior* | | | | | |
| Promoting Good | 7.84 (7.74,7.93) | 7.90 (7.80,8.00) | 7.31 (6.58,8.03) | 3.7e-01 | 1.97e-01 |
| Delayed Gratification | 7.32 (7.21,7.44) | 7.20 (7.08,7.31) | 6.42 (5.46,7.38) | 7.82e-01 | 6.16e-02 |
| Hope | 7.65 (7.53,7.78) | 7.81 (7.69,7.92) | 6.29 (4.93,7.64) | 1.76e-01 | 1.77e-02 |
| Gratitude | 7.55 (7.41,7.69) | 8.13 (8.01,8.26) | 7.78 (6.78,8.78) | 1.39e-05 | 1.19e-08 |
| Love | 7.92 (7.81,8.03) | 8.68 (8.59,8.76) | 8.51 (7.86,9.15) | 1.07e-13 | 1.6e-16 |
| Forgiveness | 0.76 (0.74,0.79) | 0.80 (0.78,0.83) | 0.96 (0.86,1.06) | 9.04e-02 | 7.05e-07 |
| Charitable Giving | 0.49 (0.46,0.52) | 0.58 (0.55,0.60) | 0.37 (0.15,0.59) | 1.16e-02 | 7.68e-05 |
| Helping | 0.59 (0.56,0.62) | 0.65 (0.62,0.67) | 0.63 (0.40,0.86) | 7.47e-02 | 1.4e-02 |
| Volunteering | 0.33 (0.30,0.35) | 0.35 (0.32,0.38) | 0.16 (0.02,0.31) | 3.06e-01 | 1.73e-02 |
| *Physical Health & Health Behavior* | | | | | |
| Self-Rated Physical Health | 6.60 (6.49,6.71) | 6.39 (6.27,6.52) | 5.99 (5.04,6.94) | 8.2e-02 | 2.9e-02 |
| Health Limitations | 0.23 (0.21,0.26) | 0.32 (0.29,0.34) | 0.55 (0.31,0.80) | 5.8e-05 | 1.48e-06 |
| Pain | 0.51 (0.48,0.54) | 0.60 (0.58,0.63) | 0.63 (0.39,0.87) | 4.7e-05 | 7.53e-06 |
| Smoking | 1.56 (1.24,1.88) | 1.04 (0.82,1.27) | 0.82 (0.00,1.93) | 1.5e-02 | 2.67e-02 |
| Drinking | 5.88 (5.33,6.44) | 3.21 (2.90,3.52) | 1.52 (0.08,2.97) | 1.15e-08 | 1.6e-16 |
| Exercise | 2.86 (2.73,2.99) | 2.53 (2.40,2.65) | 2.11 (1.25,2.98) | 1.79e-03 | 6.01e-04 |
| *Socioeconomic Outcomes* | | | | | |
| Financial Stability | 6.78 (6.60,6.96) | 6.62 (6.45,6.79) | 5.37 (3.73,7.02) | 5.14e-01 | 1.14e-01 |
| Material Stability | 7.31 (7.15,7.48) | 7.27 (7.11,7.44) | 5.49 (4.19,6.78) | 8.69e-01 | 1.63e-02 |
| Education | 0.31 (0.29,0.33) | 0.39 (0.36,0.41) | 0.26 (0.09,0.44) | 1.06e-03 | 6.63e-05 |
| Employment | 0.63 (0.60,0.66) | 0.55 (0.52,0.57) | 0.73 (0.49,0.96) | 5.27e-05 | 4.7e-05 |
| Subjective Financial Well-Being | 0.86 (0.83,0.88) | 0.83 (0.80,0.85) | 0.62 (0.38,0.86) | 8.17e-01 | 2.78e-02 |
| Housing | 0.68 (0.65,0.71) | 0.71 (0.68,0.74) | 0.27 (0.05,0.48) | 1.57e-01 | 9.53e-05 |
| *Religion/Spirituality* | | | | | |
| Self-Reported Religion/Spirituality | 0.30 (0.27,0.32) | 0.44 (0.41,0.47) | 0.24 (0.03,0.46) | 3.52e-07 | 6.62e-13 |

| Outcome | Male | Female | Other | Male vs Female p-value | Global p-value |
|---|---|---|---|---|---|
| Religious Service Attendance | 0.10 (0.09,0.12) | 0.14 (0.12,0.16) | 0.03 (0.00,0.08) | 2.32e-02 | 2.65e-05 |
| Life after Death Belief | 0.28 (0.25,0.30) | 0.45 (0.42,0.47) | 0.41 (0.16,0.65) | 1.3e-10 | 1.6e-16 |
| Religious Experience | 0.23 (0.21,0.26) | 0.27 (0.24,0.29) | 0.31 (0.09,0.54) | 6.79e-01 | 9.98e-02 |
| Religious Reading | 0.09 (0.07,0.10) | 0.12 (0.10,0.14) | 0.00 * | 2.19e-02 | 1.6e-16 |
| Prayer-Meditation | 0.17 (0.14,0.19) | 0.26 (0.24,0.29) | 0.08 (0.00,0.18) | 4.69e-06 | 1.45e-09 |
| Belief in God | 0.46 (0.43,0.49) | 0.61 (0.58,0.64) | 0.58 (0.34,0.83) | 4.19e-08 | 1.53e-12 |
| Intrinsic Religiosity | 0.38 (0.34,0.42) | 0.47 (0.43,0.50) | 0.22 (0.02,0.41) | 3.26e-03 | 1.22e-05 |
| Religious Comfort | 0.41 (0.38,0.44) | 0.55 (0.52,0.58) | 0.31 (0.03,0.59) | 2.36e-06 | 3.89e-11 |
| Loved by God | 0.36 (0.32,0.39) | 0.50 (0.47,0.54) | 0.25 (0.00,0.56) | 5.38e-08 | 1.64e-13 |
| Spiritual Punishment | 0.11 (0.08,0.14) | 0.07 (0.05,0.09) | 0.15 (0.00,0.38) | 2.42e-02 | 9.35e-03 |
| Religious Criticism | 0.19 (0.15,0.23) | 0.17 (0.15,0.20) | 0.38 (0.08,0.68) | 3.15e-01 | 1.15e-01 |
| Evangelism | 0.40 (0.37,0.44) | 0.43 (0.39,0.47) | 0.44 (0.10,0.77) | 9.88e-02 | 2.55e-01 |
| *Family Factors* | | | | | |
| Ever Married | 0.64 (0.61,0.67) | 0.69 (0.66,0.71) | 0.23 (0.03,0.43) | 1.47e-02 | 2.29e-06 |
| Divorced | 0.08 (0.06,0.09) | 0.10 (0.08,0.11) | 0.14 (0.00,0.31) | 3.77e-02 | 8.54e-02 |
| Children | 0.52 (0.47,0.58) | 0.53 (0.47,0.59) | 0.20 (0.00,0.50) | 4.92e-01 | 9.45e-02 |

**Table S3a. Nationally representative descriptive statistics for Brazil**

| Characteristic | N = 13,204[1] |
|---|---|
| **Age group** | |
| 18-24 | 1,986 (15%) |
| 25-29 | 1,468 (11%) |
| 30-39 | 2,908 (22%) |
| 40-49 | 2,638 (20%) |
| 50-59 | 2,131 (16%) |
| 60-69 | 1,435 (11%) |
| 70-79 | 510 (3.9%) |
| 80 or older | 126 (1.0%) |
| (Missing) | 0 (0%) |
| **Gender** | |
| Male | 6,320 (48%) |
| Female | 6,820 (52%) |
| Other | 35 (0.3%) |
| (Missing) | 30 (0.2%) |
| **Marital status** | |
| Married | 4,646 (35%) |
| Separated | 594 (4.5%) |
| Divorced | 865 (6.5%) |
| Widowed | 408 (3.1%) |
| Single, never married | 4,347 (33%) |
| Domestic Partner | 2,081 (16%) |
| (Missing) | 263 (2.0%) |
| **Employment** | |
| Employed for an employer | 3,756 (28%) |
| Self-employed | 2,918 (22%) |
| Retired | 1,536 (12%) |
| Student | 624 (4.7%) |
| Homemaker | 1,305 (9.9%) |
| Unemployed and looking for a job | 2,419 (18%) |
| None of these/Other | 448 (3.4%) |
| (Missing) | 199 (1.5%) |
| **Religious service attendance** | |
| More than 1/week | 2,386 (18%) |
| 1/week | 2,272 (17%) |
| 1-3/month | 1,398 (11%) |
| A few times a year | 3,978 (30%) |
| Never | 3,110 (24%) |
| (Missing) | 61 (0.5%) |
| **Education** | |
| Up to 8 years | 3,139 (24%) |
| 9-15 years | 7,665 (58%) |
| 16+ years | 2,390 (18%) |
| (Missing) | 10 (<0.1%) |
| **Immigration** | |
| Born in this country | 12,688 (96%) |
| Born in another country | 153 (1.2%) |
| (Missing) | 363 (2.7%) |
| **Religious affiliation** | |
| Christianity | 9,911 (75%) |
| Islam | 6 (<0.1%) |
| Hinduism | 1 (<0.1%) |
| Buddhism | 37 (0.3%) |
| Judaism | 31 (0.2%) |
| Sikhism | 0 (0%) |
| Baha'i | 2 (<0.1%) |
| Jainism | 2 (<0.1%) |
| Shinto | 1 (<0.1%) |
| Taoism | 1 (<0.1%) |
| Confucianism | 6 (<0.1%) |
| Primal, Animist, or Folk religion | 15 (0.1%) |
| Spiritism | 696 (5.3%) |
| Umbanda, Candomble, and other African-derived religions | 525 (4.0%) |
| Chinese folk/traditional religion | 0 (0%) |
| Some other religion | 144 (1.1%) |
| No religion/Atheist/Agnostic | 1,712 (13%) |
| (Missing) | 113 (0.9%) |
| **Race/Ethnicity** | |

| **Characteristic** | **N = 13,204**[1] |
|---|---|
| (Missing) | 681 (5.2%) |
| Amarela | 238 (1.8%) |
| Branca | 5,169 (39%) |
| Indígena | 131 (1.0%) |
| Other | 61 (0.5%) |
| Parda | 5,125 (39%) |
| Preta | 1,615 (12%) |
| (Missing) | 184 (1.4%) |

[1]n (%)

**Table S3b. Descriptive statistics of outcome variables for Brazil**

| Characteristic | N = 13,204[1] |
|---|---|
| **Flourishing Index** | 7.62 (1.72) |
| (Missing) | 286 |
| **Secure Flourishing Index** | 7.01 (1.67) |
| (Missing) | 313 |
| **Happiness & Life Satisfaction** | 7.24 (2.23) |
| (Missing) | 65 |
| **Social Relationship Quality** | 7.46 (2.58) |
| (Missing) | 84 |
| **Meaning and Purpose** | 7.83 (2.11) |
| (Missing) | 64 |
| **Character & Virtue** | 8.34 (1.74) |
| (Missing) | 84 |
| **Self-Rated Health** | 7.28 (2.13) |
| (Missing) | 50 |
| **Financial and Material Worry** | 4.0 (3.6) |
| (Missing) | 47 |
| **Happiness** | 7.33 (2.30) |
| (Missing) | 37 |
| **Life Satisfaction** | 7.15 (2.48) |
| (Missing) | 33 |
| **Present Life Evaluation** | 6.59 (2.28) |
| (Missing) | 18 |
| **Future Life Evaluation** | 8.66 (1.86) |
| (Missing) | 141 |
| **Optimism** | 9.22 (1.62) |
| (Missing) | 28 |
| **Freedom** | 8.27 (2.39) |
| (Missing) | 35 |
| **Peace** | |
| Always | 3,234 (24%) |
| Often | 6,133 (46%) |
| Rarely | 3,345 (25%) |
| Never | 446 (3.4%) |
| (Missing) | 46 (0.3%) |
| **Balance in Life** | |
| Always | 2,230 (17%) |
| Often | 6,615 (50%) |
| Rarely | 3,878 (29%) |
| Never | 448 (3.4%) |
| (Missing) | 32 (0.2%) |
| **Mastery** | |
| Always | 4,325 (33%) |
| Often | 6,283 (48%) |
| Rarely | 2,260 (17%) |
| Never | 246 (1.9%) |
| (Missing) | 91 (0.7%) |
| **Meaning** | 7.86 (2.28) |
| (Missing) | 24 |
| **Purpose** | 7.80 (2.50) |
| (Missing) | 44 |
| **Self-Rated Mental Health** | 7.39 (2.58) |
| (Missing) | 24 |
| **Content with My Relationships** | 7.71 (2.66) |
| (Missing) | 43 |
| **Satisfying Relationships** | 7.2 (2.9) |
| (Missing) | 55 |
| **Social Support** | 7.2 (3.0) |
| (Missing) | 52 |
| **Intimate Friend** | |
| Yes | 10,755 (81%) |
| No | 2,339 (18%) |
| (Missing) | 110 (0.8%) |
| **Government Approval** | |
| Strongly approve | 1,631 (12%) |
| Somewhat approve | 2,970 (22%) |
| Neither approve nor disapprove | 3,247 (25%) |
| Somewhat disapprove | 2,087 (16%) |
| Strongly disapprove | 3,187 (24%) |

| Characteristic | N = 13,204[1] |
|---|---|
| (Missing) | 82 (0.6%) |
| **Political Voice** | |
| Agree | 3,562 (27%) |
| Disagree | 5,962 (45%) |
| Unsure | 3,586 (27%) |
| (Missing) | 94 (0.7%) |
| **Belonging** | 7.79 (2.56) |
| (Missing) | 168 |
| **City Satisfaction** | |
| Satisfied | 8,161 (62%) |
| Dissatisfied | 3,469 (26%) |
| Unsure | 1,469 (11%) |
| (Missing) | 104 (0.8%) |
| **Trust** | |
| All | 165 (1.3%) |
| Most | 1,099 (8.3%) |
| Some | 4,795 (36%) |
| Not very many | 5,705 (43%) |
| None | 1,357 (10%) |
| (Missing) | 83 (0.6%) |
| **Community Participation** | |
| More than once a week | 1,191 (9.0%) |
| Once a week | 1,189 (9.0%) |
| One to three times a month | 949 (7.2%) |
| A few times a year | 3,272 (25%) |
| Never | 6,522 (49%) |
| (Missing) | 80 (0.6%) |
| **Traumatic Distress** | |
| A lot | 2,673 (20%) |
| Some | 4,056 (31%) |
| Not very much | 3,040 (23%) |
| None at all | 3,368 (26%) |
| (Missing) | 67 (0.5%) |
| **Suffering** | |
| A lot | 1,555 (12%) |
| Some | 5,754 (44%) |
| Not very much | 3,091 (23%) |
| None at all | 2,738 (21%) |
| (Missing) | 66 (0.5%) |
| **Loneliness** | 3.9 (3.4) |
| (Missing) | 22 |
| **Discrimination** | |
| Always | 1,407 (11%) |
| Often | 2,650 (20%) |
| Rarely | 5,062 (38%) |
| Never | 4,048 (31%) |
| (Missing) | 37 (0.3%) |
| **Promoting Good** | 8.59 (1.79) |
| (Missing) | 49 |
| **Delayed Gratification** | 8.10 (2.26) |
| (Missing) | 37 |
| **Hope** | 8.87 (1.88) |
| (Missing) | 29 |
| **Gratitude** | 8.61 (2.05) |
| (Missing) | 37 |
| **Love** | 8.47 (2.18) |
| (Missing) | 26 |
| **Forgiveness** | |
| Always | 4,761 (36%) |
| Often | 4,799 (36%) |
| Rarely | 2,948 (22%) |
| Never | 664 (5.0%) |
| (Missing) | 32 (0.2%) |
| **Charitable Giving** | |
| Yes | 4,101 (31%) |
| No | 9,072 (69%) |
| (Missing) | 32 (0.2%) |
| **Helping** | |
| Yes | 8,999 (68%) |

| Characteristic | N = 13,204[1] |
|---|---|
| No | 4,129 (31%) |
| (Missing) | 76 (0.6%) |
| **Volunteering** | |
| Yes | 2,445 (19%) |
| No | 10,718 (81%) |
| (Missing) | 41 (0.3%) |
| **Self-Rated Physical Health** | 7.16 (2.31) |
| (Missing) | 31 |
| **Health Limitations** | |
| Yes | 2,492 (19%) |
| No | 10,381 (79%) |
| (Missing) | 331 (2.5%) |
| **Pain** | |
| A lot | 2,387 (18%) |
| Some | 5,386 (41%) |
| Not very much | 3,051 (23%) |
| None at all | 2,355 (18%) |
| (Missing) | 25 (0.2%) |
| **Smoking** | 2.4 (6.4) |
| (Missing) | 239 |
| **Drinking** | 2.0 (5.7) |
| (Missing) | 219 |
| **Exercise** | |
| 0 days | 5,505 (42%) |
| 1 day | 1,337 (10%) |
| 2 days | 1,375 (10%) |
| 3 days | 1,454 (11%) |
| 4 days | 735 (5.6%) |
| 5 days | 936 (7.1%) |
| 6 days | 373 (2.8%) |
| 7 days/Every day | 1,440 (11%) |
| (Missing) | 47 (0.4%) |
| **Financial Stability** | 4.0 (3.8) |
| (Missing) | 23 |
| **Material Stability** | 3.9 (3.9) |
| (Missing) | 24 |
| **Education** | |
| Up to 8 years | 3,139 (24%) |
| 9-15 years | 7,665 (58%) |
| 16+ years | 2,390 (18%) |
| (Missing) | 10 (<0.1%) |
| **Employment** | |
| Employed for an employer | 3,756 (28%) |
| Self-employed | 2,918 (22%) |
| Retired | 1,536 (12%) |
| Student | 624 (4.7%) |
| Homemaker | 1,305 (9.9%) |
| Unemployed and looking for a job | 2,419 (18%) |
| None of these/Other | 448 (3.4%) |
| (Missing) | 199 (1.5%) |
| **Subjective Financial Well-Being** | |
| Living comfortably on present income | 2,003 (15%) |
| Getting by on present income | 5,744 (43%) |
| Finding it difficult on present income | 3,688 (28%) |
| Finding it very difficult on present income | 1,318 (10.0%) |
| (Missing) | 452 (3.4%) |
| **Housing** | |
| Someone in this household OWNS this home | 7,325 (55%) |
| Someone in this household RENTS this home | 3,345 (25%) |
| Both | 263 (2.0%) |
| Neither | 1,820 (14%) |
| Rent | 0 (0%) |
| Own | 0 (0%) |
| Something else | 0 (0%) |
| (Missing) | 451 (3.4%) |
| **Self-Reported Religion/Spirituality** | |
| Always | 5,929 (45%) |
| Often | 4,256 (32%) |
| Rarely | 2,328 (18%) |

| Characteristic | N = 13,204[1] |
|---|---|
| Never | 662 (5.0%) |
| (Missing) | 30 (0.2%) |
| **Religious Service Attendance** | |
| More than once a week | 2,386 (18%) |
| Once a week | 2,272 (17%) |
| One to three times a month | 1,398 (11%) |
| A few times a year | 3,978 (30%) |
| Never | 3,110 (24%) |
| (Missing) | 61 (0.5%) |
| **Life after Death Belief** | |
| Yes | 8,004 (61%) |
| No | 2,847 (22%) |
| Unsure | 2,291 (17%) |
| (Missing) | 62 (0.5%) |
| **Religious Experience** | |
| Yes | 7,808 (59%) |
| No | 5,307 (40%) |
| (Missing) | 89 (0.7%) |
| **Religious Reading** | |
| More than once a day | 2,265 (17%) |
| About once a day | 2,884 (22%) |
| Sometimes | 6,346 (48%) |
| Never | 1,577 (12%) |
| (Missing) | 131 (1.0%) |
| **Prayer-Meditation** | |
| More than once a day | 4,061 (31%) |
| About once a day | 4,398 (33%) |
| Sometimes | 3,744 (28%) |
| Never | 862 (6.5%) |
| (Missing) | 140 (1.1%) |
| **Belief in God** | |
| One God | 11,462 (87%) |
| More than one god | 323 (2.4%) |
| An impersonal spiritual force | 813 (6.2%) |
| None of these | 260 (2.0%) |
| Unsure | 264 (2.0%) |
| (Missing) | 82 (0.6%) |
| **Intrinsic Religiosity** | |
| Agree | 8,564 (65%) |
| Disagree | 1,381 (10%) |
| Not relevant | 1,665 (13%) |
| Unsure | 1,516 (11%) |
| (Missing) | 78 (0.6%) |
| **Religious Comfort** | |
| Agree | 10,386 (79%) |
| Disagree | 811 (6.1%) |
| Not relevant | 1,121 (8.5%) |
| Unsure | 841 (6.4%) |
| (Missing) | 45 (0.3%) |
| **Loved by God** | |
| Agree | 11,460 (87%) |
| Disagree | 451 (3.4%) |
| Not relevant | 681 (5.2%) |
| Unsure | 553 (4.2%) |
| (Missing) | 59 (0.4%) |
| **Spiritual Punishment** | |
| Agree | 2,393 (18%) |
| Disagree | 7,844 (59%) |
| Not relevant | 1,542 (12%) |
| Unsure | 1,357 (10%) |
| (Missing) | 68 (0.5%) |
| **Religious Criticism** | |
| Agree | 2,275 (17%) |
| Disagree | 6,394 (48%) |
| Not relevant | 2,540 (19%) |
| Unsure | 1,935 (15%) |
| (Missing) | 59 (0.5%) |
| **Evangelism** | |
| Agree | 8,196 (62%) |

| Characteristic | N = 13,204[1] |
|---|---|
| Disagree | 2,182 (17%) |
| Not relevant | 2,124 (16%) |
| Unsure | 647 (4.9%) |
| (Missing) | 55 (0.4%) |
| **Children** | 0.92 (1.34) |
| (Missing) | 318 |

[1]Mean (SD); n (%)

**Table S3c. Demographic variation across outcomes for Brazil**

| Outcome | Male | Female | Other | Male vs Female p-value | Global p-value |
|---|---|---|---|---|---|
| *Flourishing Index and Domains* | | | | | |
| Flourishing Index | 7.78 (7.72,7.84) | 7.50 (7.44,7.55) | 7.21 (6.29,8.14) | 5.48e-04 | 1.03e-11 |
| Secure Flourishing Index | 7.22 (7.17,7.28) | 6.83 (6.78,6.89) | 6.77 (5.86,7.67) | 4.31e-09 | 1.6e-16 |
| Happiness & Life Satisfaction | 7.35 (7.27,7.42) | 7.16 (7.09,7.22) | 6.82 (5.71,7.93) | 3.38e-01 | 9.66e-04 |
| Social Relationship Quality | 7.63 (7.55,7.71) | 7.30 (7.22,7.38) | 7.67 (6.70,8.65) | 5.99e-02 | 3.99e-07 |
| Meaning and Purpose | 7.91 (7.84,7.97) | 7.76 (7.70,7.83) | 7.34 (6.20,8.48) | 4.32e-01 | 8.95e-03 |
| Character & Virtue | 8.36 (8.30,8.42) | 8.33 (8.28,8.39) | 7.33 (6.25,8.41) | 8.59e-01 | 1.16e-01 |
| Self-Rated Health | 7.65 (7.59,7.71) | 6.93 (6.86,7.00) | 6.90 (5.62,8.18) | 1.6e-16 | 1.6e-16 |
| Financial and Material Worry | 4.45 (4.33,4.57) | 3.52 (3.41,3.63) | 4.53 (2.92,6.15) | 5.55e-16 | 1.6e-16 |
| *Psychological Well-Being* | | | | | |
| Happiness | 7.42 (7.34,7.49) | 7.26 (7.19,7.33) | 6.94 (5.85,8.02) | 5.75e-01 | 1.26e-02 |
| Life Satisfaction | 7.27 (7.19,7.36) | 7.05 (6.97,7.13) | 6.70 (5.51,7.89) | 2.31e-01 | 4.24e-04 |
| Present Life Evaluation | 6.62 (6.54,6.70) | 6.57 (6.50,6.64) | 6.01 (5.06,6.95) | 9.32e-01 | 2.8e-01 |
| Future Life Evaluation | 8.54 (8.47,8.60) | 8.75 (8.69,8.82) | 7.92 (6.61,9.23) | 5.38e-04 | 1.24e-05 |
| Optimism | 9.13 (9.07,9.18) | 9.30 (9.26,9.35) | 8.37 (7.17,9.57) | 2.58e-04 | 4.24e-06 |
| Freedom | 8.33 (8.25,8.40) | 8.23 (8.15,8.30) | 8.17 (7.00,9.33) | 7.08e-01 | 1.88e-01 |
| Peace | 0.77 (0.75,0.78) | 0.66 (0.65,0.68) | 0.40 (0.21,0.59) | 8.37e-10 | 1.6e-16 |
| Balance in Life | 0.71 (0.70,0.73) | 0.63 (0.62,0.65) | 0.37 (0.18,0.57) | 5.51e-03 | 6.22e-15 |
| Mastery | 0.86 (0.84,0.87) | 0.77 (0.75,0.78) | 0.74 (0.52,0.97) | 2.77e-09 | 1.6e-16 |
| Meaning | 7.89 (7.81,7.96) | 7.84 (7.77,7.91) | 7.44 (6.29,8.60) | 3.93e-01 | 5.49e-01 |
| Purpose | 7.93 (7.85,8.01) | 7.69 (7.61,7.76) | 7.23 (5.88,8.58) | 3.24e-02 | 7.86e-05 |
| Self-Rated Mental Health | 7.88 (7.80,7.95) | 6.95 (6.87,7.03) | 7.26 (5.95,8.56) | 1.6e-16 | 1.6e-16 |
| *Social Well-Being* | | | | | |
| Content with My Relationships | 7.84 (7.75,7.92) | 7.59 (7.50,7.67) | 7.44 (6.22,8.66) | 4.8e-01 | 2.32e-04 |
| Satisfying Relationships | 7.42 (7.33,7.51) | 7.02 (6.92,7.11) | 7.90 (6.87,8.93) | 5.45e-03 | 4.95e-09 |
| Social Support | 7.30 (7.20,7.39) | 7.14 (7.04,7.23) | 7.56 (6.25,8.87) | 4.55e-01 | 5.32e-02 |
| Intimate Friend | 0.81 (0.80,0.83) | 0.83 (0.82,0.84) | 0.78 (0.61,0.95) | 3.51e-03 | 1.23e-01 |
| Government Approval | 0.35 (0.33,0.36) | 0.35 (0.34,0.37) | 0.57 (0.37,0.77) | 6.21e-01 | 7.7e-02 |
| Political Voice | 0.36 (0.35,0.38) | 0.37 (0.36,0.39) | 0.37 (0.08,0.66) | 1.74e-01 | 4.13e-01 |
| Belonging | 7.69 (7.60,7.78) | 7.88 (7.80,7.95) | 7.31 (6.08,8.55) | 1.95e-03 | 5.36e-03 |
| City Satisfaction | 0.70 (0.68,0.72) | 0.69 (0.67,0.70) | 0.68 (0.48,0.88) | 7.85e-01 | 4.78e-01 |
| Trust | 0.12 (0.11,0.13) | 0.08 (0.07,0.08) | 0.03 (0.00,0.07) | 6.04e-07 | 6.86e-11 |
| Community Participation | 0.23 (0.21,0.24) | 0.14 (0.13,0.15) | 0.20 (0.00,0.42) | 1.34e-10 | 1.6e-16 |
| *Psychological Distress* | | | | | |
| Traumatic Distress | 0.47 (0.45,0.48) | 0.56 (0.54,0.57) | 0.54 (0.35,0.74) | 2.19e-05 | 2.91e-14 |
| Depression Symptoms | 0.36 (0.34,0.37) | 0.45 (0.44,0.47) | 0.52 (0.32,0.73) | 1.36e-07 | 1.6e-16 |
| Anxiety Symptoms | 0.40 (0.38,0.42) | 0.52 (0.51,0.54) | 0.41 (0.22,0.61) | 7.62e-12 | 1.6e-16 |
| Suffering | 0.49 (0.47,0.51) | 0.62 (0.60,0.63) | 0.45 (0.25,0.66) | 1.49e-10 | 1.6e-16 |
| *Social Distress* | | | | | |
| Loneliness | 3.62 (3.51,3.73) | 4.19 (4.08,4.30) | 2.82 (1.59,4.05) | 4.06e-04 | 1.38e-12 |
| Discrimination | 0.32 (0.30,0.33) | 0.30 (0.28,0.31) | 0.39 (0.20,0.59) | 3.92e-03 | 8.83e-02 |
| *Character & Prosocial Behavior* | | | | | |
| Promoting Good | 8.63 (8.57,8.69) | 8.55 (8.49,8.61) | 7.46 (6.33,8.59) | 3.96e-01 | 1.65e-02 |
| Delayed Gratification | 8.09 (8.02,8.17) | 8.12 (8.05,8.18) | 7.20 (5.94,8.47) | 6.81e-01 | 3.19e-01 |
| Hope | 8.87 (8.81,8.93) | 8.87 (8.81,8.93) | 8.24 (6.89,9.58) | 5.81e-01 | 6.26e-01 |
| Gratitude | 8.48 (8.41,8.55) | 8.73 (8.67,8.79) | 8.11 (7.01,9.20) | 1.09e-05 | 4.44e-07 |
| Love | 8.32 (8.24,8.40) | 8.61 (8.55,8.67) | 8.20 (6.98,9.42) | 3.71e-10 | 1.51e-07 |
| Forgiveness | 0.73 (0.72,0.75) | 0.72 (0.71,0.73) | 0.63 (0.42,0.84) | 4.32e-01 | 2.78e-01 |
| Charitable Giving | 0.32 (0.30,0.33) | 0.31 (0.29,0.32) | 0.21 (0.06,0.36) | 6.64e-01 | 2e-01 |
| Helping | 0.70 (0.69,0.72) | 0.67 (0.65,0.68) | 0.68 (0.46,0.90) | 1.49e-01 | 1.42e-02 |
| Volunteering | 0.18 (0.17,0.20) | 0.19 (0.18,0.20) | 0.17 (0.08,0.26) | 5.4e-01 | 7.19e-01 |
| *Physical Health & Health Behavior* | | | | | |
| Self-Rated Physical Health | 7.43 (7.36,7.50) | 6.91 (6.83,6.99) | 6.54 (5.09,7.99) | 2.21e-09 | 1.6e-16 |
| Health Limitations | 0.19 (0.18,0.21) | 0.20 (0.18,0.21) | 0.24 (0.07,0.41) | 6.89e-01 | 7.17e-01 |
| Pain | 0.52 (0.50,0.53) | 0.66 (0.64,0.67) | 0.60 (0.41,0.79) | 4.77e-15 | 1.6e-16 |
| Smoking | 2.87 (2.65,3.09) | 1.86 (1.69,2.03) | 1.74 (0.37,3.11) | 9.87e-09 | 1.28e-11 |
| Drinking | 2.44 (2.26,2.63) | 1.36 (1.26,1.45) | 2.66 (0.70,4.62) | 8.08e-13 | 1.6e-16 |
| Exercise | 2.44 (2.36,2.53) | 1.90 (1.83,1.97) | 2.12 (1.21,3.03) | 1.75e-11 | 1.6e-16 |
| *Socioeconomic Outcomes* | | | | | |
| Financial Stability | 4.52 (4.39,4.64) | 3.57 (3.46,3.68) | 4.45 (2.80,6.10) | 1.67e-13 | 1.6e-16 |
| Material Stability | 4.39 (4.26,4.52) | 3.47 (3.36,3.59) | 4.62 (2.84,6.39) | 9.99e-15 | 1.6e-16 |
| Education | 0.18 (0.17,0.19) | 0.18 (0.17,0.19) | 0.28 (0.12,0.43) | 8.72e-01 | 4.29e-01 |
| Employment | 0.61 (0.59,0.62) | 0.43 (0.41,0.44) | 0.44 (0.24,0.63) | 1.6e-16 | 1.6e-16 |
| Subjective Financial Well-Being | 0.65 (0.63,0.66) | 0.56 (0.55,0.58) | 0.56 (0.36,0.76) | 6.26e-06 | 3.45e-12 |
| Housing | 0.60 (0.58,0.62) | 0.58 (0.57,0.60) | 0.43 (0.20,0.65) | 5.53e-01 | 6.66e-02 |
| *Religion/Spirituality* | | | | | |
| Self-Reported Religion/Spirituality | 0.74 (0.73,0.76) | 0.80 (0.79,0.82) | 0.73 (0.54,0.91) | 1.13e-08 | 8.03e-10 |

| Outcome | Male | Female | Other | Male vs Female p-value | Global p-value |
|---|---|---|---|---|---|
| Religious Service Attendance | 0.34 (0.32,0.35) | 0.37 (0.36,0.39) | 0.20 (0.00,0.42) | 2.87e-02 | 2.28e-03 |
| Life after Death Belief | 0.62 (0.60,0.63) | 0.60 (0.59,0.62) | 0.47 (0.26,0.67) | 1.31e-01 | 1.47e-01 |
| Religious Experience | 0.58 (0.56,0.59) | 0.61 (0.60,0.63) | 0.58 (0.37,0.78) | 9.36e-03 | 5.69e-03 |
| Religious Reading | 0.37 (0.35,0.39) | 0.42 (0.40,0.43) | 0.32 (0.10,0.54) | 4.9e-03 | 2.29e-04 |
| Prayer-Meditation | 0.60 (0.58,0.61) | 0.70 (0.68,0.71) | 0.58 (0.39,0.78) | 2.8e-10 | 1.6e-16 |
| Belief in God | 0.94 (0.93,0.95) | 0.98 (0.97,0.98) | 0.96 (0.90,1.02) | 2.38e-07 | 2.8e-12 |
| Intrinsic Religiosity | 0.78 (0.76,0.79) | 0.84 (0.83,0.86) | 0.62 (0.40,0.84) | 1.28e-10 | 1.31e-13 |
| Religious Comfort | 0.85 (0.84,0.87) | 0.92 (0.91,0.93) | 0.83 (0.67,0.98) | 1.47e-13 | 1.6e-16 |
| Loved by God | 0.91 (0.90,0.92) | 0.96 (0.95,0.96) | 0.86 (0.74,0.98) | 6.22e-09 | 1.6e-16 |
| Spiritual Punishment | 0.25 (0.24,0.27) | 0.22 (0.20,0.24) | 0.20 (0.00,0.45) | 6.36e-03 | 2.31e-03 |
| Religious Criticism | 0.27 (0.25,0.29) | 0.26 (0.24,0.28) | 0.27 (0.07,0.47) | 3.28e-02 | 3.18e-01 |
| Evangelism | 0.75 (0.73,0.76) | 0.79 (0.77,0.81) | 0.74 (0.46,1.03) | 1.99e-04 | 1.94e-05 |
| *Family Factors* | | | | | |
| Ever Married | 0.53 (0.51,0.55) | 0.48 (0.47,0.50) | 0.34 (0.12,0.56) | 1.74e-02 | 9.21e-05 |
| Divorced | 0.06 (0.06,0.07) | 0.07 (0.06,0.08) | 0.06 (0.00,0.14) | 1.41e-01 | 8.29e-01 |
| Children | 0.84 (0.80,0.88) | 0.99 (0.96,1.03) | 0.55 (0.19,0.90) | 2.05e-07 | 2.04e-09 |

**Table S4a. Nationally representative descriptive statistics for Egypt**

| Characteristic | N = 4,729[1] |
|---|---|
| **Age group** | |
| 18-24 | 960 (20%) |
| 25-29 | 607 (13%) |
| 30-39 | 1,204 (25%) |
| 40-49 | 897 (19%) |
| 50-59 | 613 (13%) |
| 60-69 | 387 (8.2%) |
| 70-79 | 54 (1.1%) |
| 80 or older | 7 (0.2%) |
| (Missing) | 0 (0%) |
| **Gender** | |
| Male | 2,394 (51%) |
| Female | 2,334 (49%) |
| Other | 0 (0%) |
| (Missing) | 0 (<0.1%) |
| **Marital status** | |
| Married | 3,387 (72%) |
| Separated | 39 (0.8%) |
| Divorced | 101 (2.1%) |
| Widowed | 238 (5.0%) |
| Single, never married | 947 (20%) |
| Domestic Partner | 0 (0%) |
| (Missing) | 17 (0.4%) |
| **Employment** | |
| Employed for an employer | 1,267 (27%) |
| Self-employed | 892 (19%) |
| Retired | 253 (5.4%) |
| Student | 297 (6.3%) |
| Homemaker | 1,772 (37%) |
| Unemployed and looking for a job | 224 (4.7%) |
| None of these/Other | 21 (0.4%) |
| (Missing) | 3 (<0.1%) |
| **Religious service attendance** | |
| More than 1/week | 839 (18%) |
| 1/week | 960 (20%) |
| 1-3/month | 368 (7.8%) |
| A few times a year | 458 (9.7%) |
| Never | 2,091 (44%) |
| (Missing) | 12 (0.3%) |
| **Education** | |
| Up to 8 years | 2,486 (53%) |
| 9-15 years | 1,599 (34%) |
| 16+ years | 643 (14%) |
| (Missing) | 1 (<0.1%) |
| **Immigration** | |
| Born in this country | 4,713 (100%) |
| Born in another country | 16 (0.3%) |
| (Missing) | 1 (<0.1%) |
| **Religious affiliation** | |
| Christianity | 120 (2.5%) |
| Islam | 4,607 (97%) |
| Hinduism | 0 (0%) |
| Buddhism | 0 (0%) |
| Judaism | 0 (0%) |
| Sikhism | 0 (0%) |
| Baha'i | 0 (0%) |
| Jainism | 0 (0%) |
| Shinto | 0 (0%) |
| Taoism | 0 (<0.1%) |
| Confucianism | 0 (0%) |
| Primal, Animist, or Folk religion | 0 (0%) |
| Spiritism | 0 (0%) |
| Umbanda, Candomble, and other African-derived religions | 0 (0%) |
| Chinese folk/traditional religion | 0 (0%) |
| Some other religion | 0 (0%) |
| No religion/Atheist/Agnostic | 0 (0%) |
| (Missing) | 1 (<0.1%) |
| **Race/Ethnicity** | |

| Characteristic | N = 4,729[1] |
|---|---|
| (Missing) | 102 (2.2%) |
| Arab | 4,585 (97%) |
| Bedouin Arab | 4 (<0.1%) |
| Greek | 1 (<0.1%) |
| Nubian | 27 (0.6%) |
| Turkish | 9 (0.2%) |

[1]n (%)

**Table S4b. Descriptive statistics of outcome variables for Egypt**

| Characteristic | N = 4,729[1] |
|---|---|
| **Flourishing Index** | 7.64 (1.46) |
| (Missing) | 210 |
| **Secure Flourishing Index** | 7.32 (1.50) |
| (Missing) | 220 |
| **Happiness & Life Satisfaction** | 6.94 (2.44) |
| (Missing) | 45 |
| **Social Relationship Quality** | 7.88 (2.31) |
| (Missing) | 22 |
| **Meaning and Purpose** | 7.99 (1.98) |
| (Missing) | 51 |
| **Character & Virtue** | 7.65 (2.00) |
| (Missing) | 104 |
| **Self-Rated Health** | 7.71 (1.97) |
| (Missing) | 51 |
| **Financial and Material Worry** | 5.7 (3.1) |
| (Missing) | 21 |
| **Happiness** | 6.2 (2.9) |
| (Missing) | 19 |
| **Life Satisfaction** | 7.69 (2.83) |
| (Missing) | 28 |
| **Present Life Evaluation** | 5.0 (2.9) |
| (Missing) | 33 |
| **Future Life Evaluation** | 7.11 (2.76) |
| (Missing) | 838 |
| **Optimism** | 7.66 (2.57) |
| (Missing) | 73 |
| **Freedom** | 7.56 (2.70) |
| (Missing) | 14 |
| **Peace** | |
| Always | 1,745 (37%) |
| Often | 2,327 (49%) |
| Rarely | 480 (10%) |
| Never | 168 (3.6%) |
| (Missing) | 8 (0.2%) |
| **Balance in Life** | |
| Always | 1,207 (26%) |
| Often | 2,580 (55%) |
| Rarely | 707 (15%) |
| Never | 223 (4.7%) |
| (Missing) | 13 (0.3%) |
| **Mastery** | |
| Always | 2,260 (48%) |
| Often | 1,970 (42%) |
| Rarely | 368 (7.8%) |
| Never | 125 (2.6%) |
| (Missing) | 5 (0.1%) |
| **Meaning** | 8.01 (2.40) |
| (Missing) | 21 |
| **Purpose** | 7.96 (2.41) |
| (Missing) | 34 |
| **Self-Rated Mental Health** | 8.61 (2.09) |
| (Missing) | 37 |
| **Content with My Relationships** | 7.93 (2.69) |
| (Missing) | 11 |
| **Satisfying Relationships** | 7.84 (2.60) |
| (Missing) | 15 |
| **Social Support** | 6.9 (3.6) |
| (Missing) | 12 |
| **Intimate Friend** | |
| Yes | 4,133 (87%) |
| No | 583 (12%) |
| (Missing) | 13 (0.3%) |
| **Government Approval** | |
| Strongly approve | 0 (0%) |
| Somewhat approve | 0 (0%) |
| Neither approve nor disapprove | 0 (0%) |
| Somewhat disapprove | 0 (0%) |
| Strongly disapprove | 0 (0%) |

| Characteristic | N = 4,729[1] |
|---|---|
| (Missing) | 4,729 (100%) |
| **Political Voice** | |
| Agree | 2,259 (48%) |
| Disagree | 1,076 (23%) |
| Unsure | 1,280 (27%) |
| (Missing) | 113 (2.4%) |
| **Belonging** | 8.86 (2.30) |
| (Missing) | 16 |
| **City Satisfaction** | |
| Satisfied | 3,557 (75%) |
| Dissatisfied | 1,089 (23%) |
| Unsure | 81 (1.7%) |
| (Missing) | 2 (<0.1%) |
| **Trust** | |
| All | 284 (6.0%) |
| Most | 948 (20%) |
| Some | 1,299 (27%) |
| Not very many | 1,382 (29%) |
| None | 738 (16%) |
| (Missing) | 78 (1.7%) |
| **Community Participation** | |
| More than once a week | 199 (4.2%) |
| Once a week | 134 (2.8%) |
| One to three times a month | 160 (3.4%) |
| A few times a year | 294 (6.2%) |
| Never | 3,935 (83%) |
| (Missing) | 6 (0.1%) |
| **Traumatic Distress** | |
| A lot | 1,691 (36%) |
| Some | 812 (17%) |
| Not very much | 684 (14%) |
| None at all | 1,528 (32%) |
| (Missing) | 13 (0.3%) |
| **Suffering** | |
| A lot | 1,022 (22%) |
| Some | 1,360 (29%) |
| Not very much | 690 (15%) |
| None at all | 1,651 (35%) |
| (Missing) | 6 (0.1%) |
| **Loneliness** | 3.8 (3.7) |
| (Missing) | 3 |
| **Discrimination** | |
| Always | 723 (15%) |
| Often | 1,488 (31%) |
| Rarely | 781 (17%) |
| Never | 1,670 (35%) |
| (Missing) | 67 (1.4%) |
| **Promoting Good** | 8.35 (2.08) |
| (Missing) | 60 |
| **Delayed Gratification** | 6.9 (3.0) |
| (Missing) | 56 |
| **Hope** | 8.18 (2.44) |
| (Missing) | 30 |
| **Gratitude** | 7.74 (2.52) |
| (Missing) | 51 |
| **Love** | 8.27 (2.67) |
| (Missing) | 12 |
| **Forgiveness** | |
| Always | 2,731 (58%) |
| Often | 1,385 (29%) |
| Rarely | 419 (8.9%) |
| Never | 191 (4.0%) |
| (Missing) | 3 (<0.1%) |
| **Charitable Giving** | |
| Yes | 2,716 (57%) |
| No | 1,979 (42%) |
| (Missing) | 34 (0.7%) |
| **Helping** | |
| Yes | 3,396 (72%) |

| Characteristic | N = 4,729[1] |
|---|---|
| No | 1,289 (27%) |
| (Missing) | 44 (0.9%) |
| **Volunteering** | |
| Yes | 209 (4.4%) |
| No | 4,514 (95%) |
| (Missing) | 6 (0.1%) |
| **Self-Rated Physical Health** | 6.81 (2.81) |
| (Missing) | 14 |
| **Health Limitations** | |
| Yes | 1,193 (25%) |
| No | 3,522 (74%) |
| (Missing) | 14 (0.3%) |
| **Pain** | |
| A lot | 1,705 (36%) |
| Some | 1,146 (24%) |
| Not very much | 771 (16%) |
| None at all | 1,101 (23%) |
| (Missing) | 6 (0.1%) |
| **Smoking** | 3 (8) |
| (Missing) | 34 |
| **Drinking** | |
| 0 | 4,654 (99%) |
| 1 | 27 (0.6%) |
| 2 | 6 (0.1%) |
| 3 | 2 (<0.1%) |
| 6 | 2 (<0.1%) |
| 7 | 1 (<0.1%) |
| (Missing) | 37 |
| **Exercise** | |
| 0 days | 3,760 (80%) |
| 1 day | 195 (4.1%) |
| 2 days | 233 (4.9%) |
| 3 days | 188 (4.0%) |
| 4 days | 66 (1.4%) |
| 5 days | 46 (1.0%) |
| 6 days | 24 (0.5%) |
| 7 days/Every day | 206 (4.4%) |
| (Missing) | 11 (0.2%) |
| **Financial Stability** | 5.3 (3.4) |
| (Missing) | 11 |
| **Material Stability** | 6.2 (3.4) |
| (Missing) | 12 |
| **Education** | |
| Up to 8 years | 2,486 (53%) |
| 9-15 years | 1,599 (34%) |
| 16+ years | 643 (14%) |
| (Missing) | 1 (<0.1%) |
| **Employment** | |
| Employed for an employer | 1,267 (27%) |
| Self-employed | 892 (19%) |
| Retired | 253 (5.4%) |
| Student | 297 (6.3%) |
| Homemaker | 1,772 (37%) |
| Unemployed and looking for a job | 224 (4.7%) |
| None of these/Other | 21 (0.4%) |
| (Missing) | 3 (<0.1%) |
| **Subjective Financial Well-Being** | |
| Living comfortably on present income | 323 (6.8%) |
| Getting by on present income | 2,365 (50%) |
| Finding it difficult on present income | 1,031 (22%) |
| Finding it very difficult on present income | 948 (20%) |
| (Missing) | 62 (1.3%) |
| **Housing** | |
| Someone in this household OWNS this home | 3,442 (73%) |
| Someone in this household RENTS this home | 1,031 (22%) |
| Both | 21 (0.4%) |
| Neither | 212 (4.5%) |
| Rent | 0 (0%) |
| Own | 0 (0%) |

| Characteristic | N = 4,729[1] |
|---|---|
| Something else | 0 (0%) |
| (Missing) | 23 (0.5%) |
| **Self-Reported Religion/Spirituality** | |
| Always | 3,600 (76%) |
| Often | 700 (15%) |
| Rarely | 206 (4.4%) |
| Never | 214 (4.5%) |
| (Missing) | 10 (0.2%) |
| **Religious Service Attendance** | |
| More than once a week | 839 (18%) |
| Once a week | 960 (20%) |
| One to three times a month | 368 (7.8%) |
| A few times a year | 458 (9.7%) |
| Never | 2,091 (44%) |
| (Missing) | 12 (0.3%) |
| **Life after Death Belief** | |
| Yes | 3,022 (64%) |
| No | 1,054 (22%) |
| Unsure | 627 (13%) |
| (Missing) | 26 (0.6%) |
| **Religious Experience** | |
| Yes | 1,628 (34%) |
| No | 3,061 (65%) |
| (Missing) | 40 (0.9%) |
| **Religious Reading** | |
| More than once a day | 1,589 (34%) |
| About once a day | 1,138 (24%) |
| Sometimes | 1,608 (34%) |
| Never | 384 (8.1%) |
| (Missing) | 10 (0.2%) |
| **Prayer-Meditation** | |
| More than once a day | 3,566 (75%) |
| About once a day | 194 (4.1%) |
| Sometimes | 879 (19%) |
| Never | 79 (1.7%) |
| (Missing) | 11 (0.2%) |
| **Belief in God** | |
| One God | 4,719 (100%) |
| More than one god | 0 (0%) |
| An impersonal spiritual force | 0 (0%) |
| None of these | 2 (<0.1%) |
| Unsure | 0 (0%) |
| (Missing) | 8 (0.2%) |
| **Intrinsic Religiosity** | |
| Agree | 4,229 (89%) |
| Disagree | 233 (4.9%) |
| Not relevant | 51 (1.1%) |
| Unsure | 197 (4.2%) |
| (Missing) | 19 (0.4%) |
| **Religious Comfort** | |
| Agree | 4,597 (97%) |
| Disagree | 51 (1.1%) |
| Not relevant | 16 (0.3%) |
| Unsure | 45 (1.0%) |
| (Missing) | 19 (0.4%) |
| **Loved by God** | |
| Agree | 4,633 (98%) |
| Disagree | 16 (0.3%) |
| Not relevant | 3 (<0.1%) |
| Unsure | 65 (1.4%) |
| (Missing) | 12 (0.2%) |
| **Spiritual Punishment** | |
| Agree | 4,132 (87%) |
| Disagree | 370 (7.8%) |
| Not relevant | 54 (1.2%) |
| Unsure | 146 (3.1%) |
| (Missing) | 27 (0.6%) |
| **Religious Criticism** | |
| Agree | 1,335 (28%) |

| Characteristic | N = 4,729[1] |
|---|---|
| Disagree | 2,660 (56%) |
| Not relevant | 342 (7.2%) |
| Unsure | 374 (7.9%) |
| (Missing) | 18 (0.4%) |
| **Evangelism** | |
| Agree | 1,942 (41%) |
| Disagree | 2,289 (48%) |
| Not relevant | 343 (7.3%) |
| Unsure | 134 (2.8%) |
| (Missing) | 20 (0.4%) |
| **Children** | 1.77 (1.52) |
| (Missing) | 2 |

[1]Mean (SD); n (%)

**Table S4c. Demographic variation across outcomes for Egypt**

| Outcome | Male | Female | Other | Male vs Female p-value | Global p-value |
|---|---|---|---|---|---|
| *Flourishing Index and Domains* | | | | | |
| Flourishing Index | 7.53 (7.44,7.61) | 7.74 (7.67,7.80) | 7.63 (7.56,7.71) | 7.83e-03 | 5.57e-05 |
| Secure Flourishing Index | 7.26 (7.17,7.35) | 7.38 (7.30,7.45) | 7.30 (7.23,7.38) | 2.12e-01 | 1.86e-02 |
| Happiness & Life Satisfaction | 6.75 (6.61,6.89) | 7.12 (7.00,7.24) | 6.97 (6.85,7.09) | 8.95e-03 | 4.06e-05 |
| Social Relationship Quality | 7.74 (7.62,7.87) | 8.03 (7.94,8.13) | 7.96 (7.87,8.05) | 1e-02 | 2.56e-04 |
| Meaning and Purpose | 7.74 (7.63,7.86) | 8.23 (8.15,8.30) | 8.04 (7.94,8.13) | 8.85e-06 | 1.11e-10 |
| Character & Virtue | 7.51 (7.41,7.62) | 7.78 (7.69,7.87) | 7.60 (7.52,7.68) | 2.06e-02 | 3.89e-04 |
| Self-Rated Health | 7.88 (7.76,7.99) | 7.54 (7.45,7.63) | 7.60 (7.52,7.69) | 1.58e-03 | 3.6e-06 |
| Financial and Material Worry | 5.92 (5.75,6.09) | 5.56 (5.41,5.72) | 5.66 (5.51,5.81) | 1.13e-02 | 7.82e-04 |
| *Psychological Well-Being* | | | | | |
| Happiness | 6.03 (5.86,6.21) | 6.33 (6.19,6.47) | 6.28 (6.14,6.43) | 7.32e-02 | 4.59e-03 |
| Life Satisfaction | 7.47 (7.31,7.62) | 7.91 (7.77,8.04) | 7.66 (7.54,7.79) | 5.76e-03 | 3.23e-05 |
| Present Life Evaluation | 4.80 (4.63,4.96) | 5.29 (5.17,5.42) | 4.99 (4.87,5.12) | 2.06e-04 | 1.88e-06 |
| Future Life Evaluation | 6.88 (6.73,7.04) | 7.22 (7.09,7.35) | 6.92 (6.79,7.05) | 6.49e-03 | 1.23e-04 |
| Optimism | 7.59 (7.43,7.74) | 7.75 (7.63,7.88) | 7.67 (7.56,7.79) | 3.76e-01 | 8.23e-02 |
| Freedom | 7.76 (7.61,7.91) | 7.36 (7.23,7.48) | 7.49 (7.37,7.61) | 9.81e-05 | 5.99e-05 |
| Peace | 0.84 (0.82,0.86) | 0.88 (0.87,0.90) | 0.87 (0.86,0.88) | 7.68e-03 | 8.61e-04 |
| Balance in Life | 0.79 (0.77,0.81) | 0.81 (0.80,0.83) | 0.81 (0.80,0.83) | 1.68e-02 | 9.11e-02 |
| Mastery | 0.89 (0.88,0.91) | 0.90 (0.88,0.91) | 0.90 (0.88,0.91) | 4.18e-01 | 7.04e-01 |
| Meaning | 7.71 (7.58,7.84) | 8.31 (8.22,8.40) | 8.10 (7.99,8.21) | 8e-06 | 7.89e-12 |
| Purpose | 7.77 (7.63,7.92) | 8.14 (8.04,8.25) | 7.98 (7.87,8.09) | 5.21e-03 | 3.02e-05 |
| Self-Rated Mental Health | 8.64 (8.54,8.74) | 8.57 (8.46,8.68) | 8.57 (8.49,8.66) | 3.9e-01 | 3.38e-01 |
| *Social Well-Being* | | | | | |
| Content with My Relationships | 7.74 (7.59,7.88) | 8.13 (8.01,8.24) | 8.02 (7.91,8.13) | 9.1e-03 | 1.69e-05 |
| Satisfying Relationships | 7.75 (7.62,7.89) | 7.94 (7.83,8.05) | 7.89 (7.79,7.99) | 4.98e-02 | 3.6e-02 |
| Social Support | 6.56 (6.33,6.78) | 7.30 (7.14,7.47) | 6.91 (6.75,7.06) | 1.2e-04 | 8.79e-07 |
| Intimate Friend | 0.86 (0.84,0.88) | 0.89 (0.88,0.90) | 0.88 (0.87,0.90) | 4.32e-01 | 2.06e-02 |
| Political Voice | 0.66 (0.63,0.70) | 0.69 (0.67,0.72) | 0.68 (0.65,0.70) | 1.73e-01 | 6.85e-02 |
| Belonging | 8.80 (8.67,8.94) | 8.93 (8.83,9.03) | 8.97 (8.88,9.07) | 1.26e-01 | 1.61e-01 |
| City Satisfaction | 0.75 (0.73,0.77) | 0.78 (0.76,0.80) | 0.79 (0.77,0.81) | 5.79e-02 | 3.9e-02 |
| Trust | 0.27 (0.25,0.29) | 0.27 (0.24,0.29) | 0.28 (0.26,0.30) | 6.3e-01 | 8.15e-01 |
| Community Participation | 0.10 (0.08,0.12) | 0.04 (0.03,0.05) | 0.05 (0.04,0.06) | 3.7e-05 | 1.46e-09 |
| Psychological Distress | | | | | |
| *Traumatic Distress* | 0.50 (0.48,0.53) | 0.56 (0.53,0.59) | 0.54 (0.51,0.56) | 6e-02 | 1.36e-03 |
| Depression Symptoms | 0.34 (0.31,0.37) | 0.38 (0.36,0.41) | 0.35 (0.32,0.37) | 8.48e-02 | 1.99e-02 |
| Anxiety Symptoms | 0.37 (0.35,0.40) | 0.50 (0.47,0.53) | 0.44 (0.41,0.46) | 9.41e-06 | 1.26e-10 |
| Suffering | 0.46 (0.43,0.49) | 0.55 (0.52,0.57) | 0.52 (0.50,0.54) | 1.34e-03 | 1.52e-05 |
| Social Distress | | | | | |
| *Loneliness* | 3.94 (3.70,4.19) | 3.68 (3.49,3.87) | 3.52 (3.35,3.69) | 5.12e-01 | 9.52e-02 |
| Discrimination | 0.46 (0.43,0.49) | 0.49 (0.46,0.51) | 0.48 (0.45,0.50) | 6.12e-02 | 1.35e-01 |
| Character & Prosocial Behavior | | | | | |
| *Promoting Good* | 8.30 (8.20,8.40) | 8.40 (8.29,8.52) | 8.35 (8.26,8.43) | 3.4e-01 | 1.93e-01 |
| Delayed Gratification | 6.73 (6.57,6.89) | 7.15 (7.03,7.27) | 6.85 (6.74,6.97) | 1.55e-02 | 5.45e-05 |
| Hope | 7.95 (7.81,8.09) | 8.42 (8.33,8.51) | 8.21 (8.12,8.31) | 6.2e-02 | 4.24e-07 |
| Gratitude | 7.46 (7.31,7.61) | 8.02 (7.91,8.13) | 7.82 (7.71,7.93) | 8.19e-04 | 9.92e-09 |
| Love | 8.04 (7.89,8.19) | 8.51 (8.39,8.62) | 8.47 (8.37,8.57) | 2.4e-04 | 5.06e-06 |
| Forgiveness | 0.87 (0.86,0.89) | 0.87 (0.85,0.88) | 0.89 (0.88,0.90) | 3.06e-01 | 7e-01 |
| Charitable Giving | 0.61 (0.59,0.64) | 0.55 (0.52,0.57) | 0.60 (0.58,0.62) | 1.81e-04 | 2.51e-04 |
| Helping | 0.76 (0.74,0.78) | 0.69 (0.67,0.71) | 0.73 (0.71,0.75) | 1.42e-04 | 3.84e-05 |
| Volunteering | 0.06 (0.04,0.07) | 0.03 (0.02,0.04) | 0.04 (0.03,0.05) | 4.84e-02 | 7.35e-04 |
| Physical Health & Health Behavior | | | | | |
| *Self-Rated Physical Health* | 7.11 (6.95,7.27) | 6.50 (6.38,6.63) | 6.63 (6.51,6.76) | 1.98e-04 | 1.95e-08 |
| Health Limitations | 0.21 (0.19,0.23) | 0.30 (0.28,0.32) | 0.28 (0.26,0.30) | 6.63e-04 | 1.06e-08 |
| Pain | 0.55 (0.52,0.58) | 0.66 (0.63,0.68) | 0.62 (0.60,0.64) | 3.39e-05 | 1.41e-08 |
| Smoking | 6.33 (5.82,6.84) | 0.05 (0.01,0.09) | 3.09 (2.74,3.43) | 1.6e-16 | 1.6e-16 |
| Drinking | 0.02 (0.01,0.04) | 0.01 (0.00,0.02) | 0.01 (0.00,0.02) | 1.7e-03 | 4e-02 |
| Exercise | 0.98 (0.88,1.09) | 0.41 (0.35,0.46) | 0.50 (0.44,0.55) | 1.07e-07 | 1.6e-16 |
| Socioeconomic Outcomes | | | | | |
| *Financial Stability* | 5.43 (5.25,5.61) | 5.09 (4.92,5.26) | 5.21 (5.04,5.37) | 1.38e-02 | 4.4e-03 |
| Material Stability | 6.40 (6.22,6.59) | 6.04 (5.87,6.20) | 6.11 (5.96,6.26) | 3.21e-02 | 8.23e-04 |
| Education | 0.14 (0.12,0.16) | 0.13 (0.11,0.15) | 0.14 (0.12,0.15) | 8e-01 | 2.63e-01 |
| Employment | 0.75 (0.73,0.77) | 0.16 (0.14,0.17) | 0.47 (0.44,0.49) | 1.6e-16 | 1.6e-16 |
| Subjective Financial Well-Being | 0.59 (0.56,0.62) | 0.56 (0.54,0.59) | 0.57 (0.55,0.59) | 7.63e-01 | 1.56e-01 |
| Housing | 0.75 (0.72,0.78) | 0.73 (0.70,0.76) | 0.75 (0.72,0.78) | 5.03e-01 | 2.48e-01 |
| Religion/Spirituality | | | | | |
| *Self-Reported Religion/Spirituality* | 0.90 (0.88,0.92) | 0.92 (0.91,0.94) | 0.92 (0.91,0.93) | 2.49e-01 | 2.56e-02 |
| Religious Service Attendance | 0.50 (0.48,0.53) | 0.26 (0.23,0.28) | 0.38 (0.35,0.40) | 3.33e-16 | 1.6e-16 |
| Life after Death Belief | 0.69 (0.67,0.72) | 0.59 (0.57,0.61) | 0.64 (0.62,0.66) | 7.47e-05 | 8.34e-09 |

| Outcome | Male | Female | Other | Male vs Female p-value | Global p-value |
|---|---|---|---|---|---|
| Religious Experience | 0.34 (0.31,0.37) | 0.35 (0.33,0.38) | 0.33 (0.31,0.34) | 7.96e-01 | 4.82e-01 |
| Religious Reading | 0.55 (0.52,0.57) | 0.61 (0.59,0.63) | 0.58 (0.55,0.60) | 4.01e-02 | 2.26e-04 |
| Prayer-Meditation | 0.76 (0.74,0.78) | 0.84 (0.82,0.86) | 0.80 (0.78,0.82) | 1.65e-04 | 2.43e-09 |
| Belief in God | 1.00 (1.00,1.00) | 1.00 (1.00,1.00) | 1.00 (1.00,1.00) | 2.96e-01 | 3.19e-01 |
| Intrinsic Religiosity | 0.94 (0.93,0.95) | 0.95 (0.94,0.96) | 0.95 (0.94,0.96) | 3.66e-01 | 2.86e-01 |
| Religious Comfort | 0.99 (0.98,0.99) | 0.99 (0.99,1.00) | 0.99 (0.99,0.99) | 2.29e-01 | 2.84e-01 |
| Loved by God | 1.00 (0.99,1.00) | 1.00 (1.00,1.00) | 1.00 (1.00,1.00) | 3.63e-01 | 2.47e-01 |
| Spiritual Punishment | 0.93 (0.92,0.94) | 0.90 (0.89,0.92) | 0.92 (0.91,0.93) | 4.88e-03 | 2.1e-03 |
| Religious Criticism | 0.34 (0.32,0.37) | 0.33 (0.30,0.35) | 0.33 (0.31,0.35) | 2.56e-01 | 2.69e-01 |
| Evangelism | 0.47 (0.44,0.50) | 0.45 (0.42,0.47) | 0.43 (0.41,0.45) | 4.04e-01 | 2.07e-01 |
| Family Factors | | | | | |
| *Ever Married* | 0.73 (0.70,0.75) | 0.87 (0.86,0.89) | 1.00 (1.00,1.00) | 1.01e-09 | 1.6e-16 |
| Divorced | 0.01 (0.00,0.01) | 0.03 (0.03,0.04) | 0.02 (0.01,0.03) | 2.99e-04 | 4.51e-07 |
| Children | 1.71 (1.64,1.79) | 1.84 (1.77,1.92) | 2.02 (1.96,2.08) | 3.4e-01 | 5.26e-03 |

**Table S5a. Nationally representative descriptive statistics for Germany**

| Characteristic | N = 9,506[1] |
|---|---|
| **Age group** | |
| 18-24 | 829 (8.7%) |
| 25-29 | 774 (8.1%) |
| 30-39 | 1,438 (15%) |
| 40-49 | 1,494 (16%) |
| 50-59 | 1,729 (18%) |
| 60-69 | 1,915 (20%) |
| 70-79 | 1,137 (12%) |
| 80 or older | 190 (2.0%) |
| (Missing) | 0 (0%) |
| **Gender** | |
| Male | 4,641 (49%) |
| Female | 4,843 (51%) |
| Other | 11 (0.1%) |
| (Missing) | 11 (0.1%) |
| **Marital status** | |
| Married | 4,784 (50%) |
| Separated | 219 (2.3%) |
| Divorced | 767 (8.1%) |
| Widowed | 409 (4.3%) |
| Single, never married | 2,627 (28%) |
| Domestic Partner | 619 (6.5%) |
| (Missing) | 81 (0.9%) |
| **Employment** | |
| Employed for an employer | 4,950 (52%) |
| Self-employed | 712 (7.5%) |
| Retired | 2,480 (26%) |
| Student | 605 (6.4%) |
| Homemaker | 251 (2.6%) |
| Unemployed and looking for a job | 288 (3.0%) |
| None of these/Other | 204 (2.1%) |
| (Missing) | 14 (0.2%) |
| **Religious service attendance** | |
| More than 1/week | 285 (3.0%) |
| 1/week | 424 (4.5%) |
| 1-3/month | 550 (5.8%) |
| A few times a year | 2,362 (25%) |
| Never | 5,876 (62%) |
| (Missing) | 9 (<0.1%) |
| **Education** | |
| Up to 8 years | 235 (2.5%) |
| 9-15 years | 6,094 (64%) |
| 16+ years | 3,164 (33%) |
| (Missing) | 13 (0.1%) |
| **Immigration** | |
| Born in this country | 8,722 (92%) |
| Born in another country | 744 (7.8%) |
| (Missing) | 40 (0.4%) |
| **Religious affiliation** | |
| Christianity | 5,052 (53%) |
| Islam | 351 (3.7%) |
| Hinduism | 12 (0.1%) |
| Buddhism | 51 (0.5%) |
| Judaism | 19 (0.2%) |
| Sikhism | 5 (<0.1%) |
| Baha'i | 3 (<0.1%) |
| Jainism | 0 (0%) |
| Shinto | 2 (<0.1%) |
| Taoism | 0 (<0.1%) |
| Confucianism | 4 (<0.1%) |
| Primal, Animist, or Folk religion | 34 (0.4%) |
| Spiritism | 0 (0%) |
| Umbanda, Candomble, and other African-derived religions | 0 (0%) |
| Chinese folk/traditional religion | 0 (0%) |
| Some other religion | 60 (0.6%) |
| No religion/Atheist/Agnostic | 3,815 (40%) |
| (Missing) | 99 (1.0%) |

[1]n (%)

**Table S5b. Descriptive statistics of outcome variables for Germany**

| Characteristic | N = 9,506[1] |
|---|---|
| **Flourishing Index** | 7.10 (1.36) |
| (Missing) | 158 |
| **Secure Flourishing Index** | 7.00 (1.37) |
| (Missing) | 163 |
| **Happiness & Life Satisfaction** | 6.92 (1.91) |
| (Missing) | 44 |
| **Social Relationship Quality** | 7.27 (2.17) |
| (Missing) | 59 |
| **Meaning and Purpose** | 7.19 (1.83) |
| (Missing) | 33 |
| **Character & Virtue** | 7.31 (1.59) |
| (Missing) | 24 |
| **Self-Rated Health** | 6.83 (1.93) |
| (Missing) | 27 |
| **Financial and Material Worry** | 6.51 (2.68) |
| (Missing) | 11 |
| **Happiness** | 6.90 (1.91) |
| (Missing) | 14 |
| **Life Satisfaction** | 6.93 (2.08) |
| (Missing) | 32 |
| **Present Life Evaluation** | 6.74 (1.82) |
| (Missing) | 8 |
| **Future Life Evaluation** | 7.16 (1.93) |
| (Missing) | 17 |
| **Optimism** | 7.50 (2.11) |
| (Missing) | 22 |
| **Freedom** | 7.56 (2.12) |
| (Missing) | 7 |
| **Peace** | |
| Always | 1,226 (13%) |
| Often | 6,333 (67%) |
| Rarely | 1,785 (19%) |
| Never | 152 (1.6%) |
| (Missing) | 11 (0.1%) |
| **Balance in Life** | |
| Always | 779 (8.2%) |
| Often | 6,500 (68%) |
| Rarely | 2,026 (21%) |
| Never | 179 (1.9%) |
| (Missing) | 22 (0.2%) |
| **Mastery** | |
| Always | 707 (7.4%) |
| Often | 7,268 (76%) |
| Rarely | 1,334 (14%) |
| Never | 166 (1.7%) |
| (Missing) | 31 (0.3%) |
| **Meaning** | 7.30 (1.96) |
| (Missing) | 12 |
| **Purpose** | 7.08 (2.34) |
| (Missing) | 21 |
| **Self-Rated Mental Health** | 7.05 (2.24) |
| (Missing) | 18 |
| **Content with My Relationships** | 7.45 (2.20) |
| (Missing) | 37 |
| **Satisfying Relationships** | 7.09 (2.37) |
| (Missing) | 31 |
| **Social Support** | 8.09 (2.14) |
| (Missing) | 4 |
| **Intimate Friend** | |
| Yes | 8,095 (85%) |
| No | 1,377 (14%) |
| (Missing) | 33 (0.4%) |
| **Government Approval** | |
| Strongly approve | 513 (5.4%) |
| Somewhat approve | 2,710 (29%) |
| Neither approve nor disapprove | 2,881 (30%) |
| Somewhat disapprove | 2,114 (22%) |
| Strongly disapprove | 1,251 (13%) |

| Characteristic | N = 9,506[1] |
|---|---|
| (Missing) | 37 (0.4%) |
| **Political Voice** | |
| Agree | 4,008 (42%) |
| Disagree | 3,483 (37%) |
| Unsure | 2,010 (21%) |
| (Missing) | 6 (<0.1%) |
| **Belonging** | 6.98 (2.39) |
| (Missing) | 33 |
| **City Satisfaction** | |
| Satisfied | 7,649 (80%) |
| Dissatisfied | 1,072 (11%) |
| Unsure | 742 (7.8%) |
| (Missing) | 43 (0.5%) |
| **Trust** | |
| All | 70 (0.7%) |
| Most | 2,596 (27%) |
| Some | 4,136 (44%) |
| Not very many | 2,480 (26%) |
| None | 193 (2.0%) |
| (Missing) | 30 (0.3%) |
| **Community Participation** | |
| More than once a week | 966 (10%) |
| Once a week | 1,215 (13%) |
| One to three times a month | 1,182 (12%) |
| A few times a year | 2,020 (21%) |
| Never | 4,113 (43%) |
| (Missing) | 9 (<0.1%) |
| **Traumatic Distress** | |
| A lot | 694 (7.3%) |
| Some | 2,091 (22%) |
| Not very much | 2,440 (26%) |
| None at all | 4,246 (45%) |
| (Missing) | 35 (0.4%) |
| **Suffering** | |
| A lot | 844 (8.9%) |
| Some | 3,819 (40%) |
| Not very much | 3,287 (35%) |
| None at all | 1,528 (16%) |
| (Missing) | 27 (0.3%) |
| **Loneliness** | 2.99 (2.64) |
| (Missing) | 17 |
| **Discrimination** | |
| Always | 224 (2.4%) |
| Often | 1,162 (12%) |
| Rarely | 3,687 (39%) |
| Never | 4,413 (46%) |
| (Missing) | 20 (0.2%) |
| **Promoting Good** | 7.68 (1.83) |
| (Missing) | 14 |
| **Delayed Gratification** | 6.94 (2.15) |
| (Missing) | 14 |
| **Hope** | 7.65 (1.95) |
| (Missing) | 36 |
| **Gratitude** | 7.63 (2.16) |
| (Missing) | 53 |
| **Love** | 7.81 (2.02) |
| (Missing) | 7 |
| **Forgiveness** | |
| Always | 1,332 (14%) |
| Often | 5,741 (60%) |
| Rarely | 2,167 (23%) |
| Never | 247 (2.6%) |
| (Missing) | 20 (0.2%) |
| **Charitable Giving** | |
| Yes | 3,440 (36%) |
| No | 6,042 (64%) |
| (Missing) | 24 (0.2%) |
| **Helping** | |
| Yes | 4,860 (51%) |

| Characteristic | N = 9,506[1] |
|---|---|
| No | 4,606 (48%) |
| (Missing) | 40 (0.4%) |
| **Volunteering** | |
| Yes | 1,992 (21%) |
| No | 7,490 (79%) |
| (Missing) | 24 (0.3%) |
| **Self-Rated Physical Health** | 6.62 (2.10) |
| (Missing) | 12 |
| **Health Limitations** | |
| Yes | 2,790 (29%) |
| No | 6,646 (70%) |
| (Missing) | 70 (0.7%) |
| **Pain** | |
| A lot | 983 (10%) |
| Some | 2,920 (31%) |
| Not very much | 2,963 (31%) |
| None at all | 2,621 (28%) |
| (Missing) | 20 (0.2%) |
| **Smoking** | 4 (8) |
| (Missing) | 83 |
| **Drinking** | 3.9 (9.0) |
| (Missing) | 84 |
| **Exercise** | |
| 0 days | 2,573 (27%) |
| 1 day | 1,254 (13%) |
| 2 days | 1,652 (17%) |
| 3 days | 1,477 (16%) |
| 4 days | 862 (9.1%) |
| 5 days | 627 (6.6%) |
| 6 days | 322 (3.4%) |
| 7 days/Every day | 726 (7.6%) |
| (Missing) | 13 (0.1%) |
| **Financial Stability** | 6.2 (3.0) |
| (Missing) | 9 |
| **Material Stability** | 6.80 (2.78) |
| (Missing) | 8 |
| **Education** | |
| Up to 8 years | 235 (2.5%) |
| 9-15 years | 6,094 (64%) |
| 16+ years | 3,164 (33%) |
| (Missing) | 13 (0.1%) |
| **Employment** | |
| Employed for an employer | 4,950 (52%) |
| Self-employed | 712 (7.5%) |
| Retired | 2,480 (26%) |
| Student | 605 (6.4%) |
| Homemaker | 251 (2.6%) |
| Unemployed and looking for a job | 288 (3.0%) |
| None of these/Other | 204 (2.1%) |
| (Missing) | 14 (0.2%) |
| **Subjective Financial Well-Being** | |
| Living comfortably on present income | 2,974 (31%) |
| Getting by on present income | 4,438 (47%) |
| Finding it difficult on present income | 1,534 (16%) |
| Finding it very difficult on present income | 470 (4.9%) |
| (Missing) | 91 (1.0%) |
| **Housing** | |
| Someone in this household OWNS this home | 3,879 (41%) |
| Someone in this household RENTS this home | 3,762 (40%) |
| Both | 694 (7.3%) |
| Neither | 1,076 (11%) |
| Rent | 0 (0%) |
| Own | 0 (0%) |
| Something else | 0 (0%) |
| (Missing) | 94 (1.0%) |
| **Self-Reported Religion/Spirituality** | |
| Always | 1,139 (12%) |
| Often | 2,385 (25%) |
| Rarely | 3,288 (35%) |

| Characteristic | N = 9,506[1] |
|---|---|
| Never | 2,685 (28%) |
| (Missing) | 9 (<0.1%) |
| **Religious Service Attendance** | |
| More than once a week | 285 (3.0%) |
| Once a week | 424 (4.5%) |
| One to three times a month | 550 (5.8%) |
| A few times a year | 2,362 (25%) |
| Never | 5,876 (62%) |
| (Missing) | 9 (<0.1%) |
| **Life after Death Belief** | |
| Yes | 3,254 (34%) |
| No | 3,354 (35%) |
| Unsure | 2,863 (30%) |
| (Missing) | 34 (0.4%) |
| **Religious Experience** | |
| Yes | 2,014 (21%) |
| No | 7,469 (79%) |
| (Missing) | 22 (0.2%) |
| **Religious Reading** | |
| More than once a day | 191 (2.0%) |
| About once a day | 477 (5.0%) |
| Sometimes | 3,192 (34%) |
| Never | 5,618 (59%) |
| (Missing) | 29 (0.3%) |
| **Prayer-Meditation** | |
| More than once a day | 529 (5.6%) |
| About once a day | 1,077 (11%) |
| Sometimes | 3,575 (38%) |
| Never | 4,311 (45%) |
| (Missing) | 14 (0.2%) |
| **Belief in God** | |
| One God | 3,131 (33%) |
| More than one god | 302 (3.2%) |
| An impersonal spiritual force | 1,820 (19%) |
| None of these | 2,824 (30%) |
| Unsure | 1,409 (15%) |
| (Missing) | 20 (0.2%) |
| **Intrinsic Religiosity** | |
| Agree | 1,692 (18%) |
| Disagree | 2,021 (21%) |
| Not relevant | 4,788 (50%) |
| Unsure | 961 (10%) |
| (Missing) | 45 (0.5%) |
| **Religious Comfort** | |
| Agree | 2,514 (26%) |
| Disagree | 1,703 (18%) |
| Not relevant | 4,050 (43%) |
| Unsure | 1,190 (13%) |
| (Missing) | 50 (0.5%) |
| **Loved by God** | |
| Agree | 2,519 (26%) |
| Disagree | 1,533 (16%) |
| Not relevant | 4,171 (44%) |
| Unsure | 1,227 (13%) |
| (Missing) | 56 (0.6%) |
| **Spiritual Punishment** | |
| Agree | 621 (6.5%) |
| Disagree | 4,166 (44%) |
| Not relevant | 3,785 (40%) |
| Unsure | 897 (9.4%) |
| (Missing) | 38 (0.4%) |
| **Religious Criticism** | |
| Agree | 394 (4.1%) |
| Disagree | 3,834 (40%) |
| Not relevant | 4,781 (50%) |
| Unsure | 449 (4.7%) |
| (Missing) | 48 (0.5%) |
| **Evangelism** | |
| Agree | 1,969 (21%) |

| Characteristic | N = 9,506[1] |
|---|---|
| Disagree | 2,783 (29%) |
| Not relevant | 4,262 (45%) |
| Unsure | 453 (4.8%) |
| (Missing) | 39 (0.4%) |
| **Children** | 0.39 (0.95) |
| (Missing) | 47 |

[1]Mean (SD); n (%)

**Table S5c. Demographic variation across outcomes for Germany**

| Outcome | Male | Female | Other | Male vs Female p-value | Global p-value |
|---|---|---|---|---|---|
| *Flourishing Index and Domains* | | | | | |
| Flourishing Index | 7.14 (7.09,7.19) | 7.07 (7.02,7.12) | 5.69 (5.02,6.37) | 5.09e-01 | 2.64e-07 |
| Secure Flourishing Index | 7.06 (7.01,7.11) | 6.96 (6.91,7.01) | 5.45 (4.80,6.10) | 1.82e-01 | 3.38e-10 |
| Happiness & Life Satisfaction | 6.95 (6.88,7.02) | 6.88 (6.82,6.95) | 5.67 (4.45,6.88) | 6.72e-01 | 1.69e-02 |
| Social Relationship Quality | 7.25 (7.17,7.33) | 7.29 (7.21,7.37) | 6.33 (5.35,7.32) | 6.15e-02 | 5.07e-02 |
| Meaning and Purpose | 7.19 (7.12,7.26) | 7.19 (7.13,7.26) | 4.98 (3.24,6.72) | 3.87e-01 | 6.82e-03 |
| Character & Virtue | 7.37 (7.31,7.42) | 7.26 (7.21,7.32) | 6.51 (5.87,7.14) | 3.03e-02 | 2.82e-04 |
| Self-Rated Health | 6.94 (6.88,7.01) | 6.74 (6.67,6.81) | 4.98 (3.94,6.03) | 2.15e-03 | 1.82e-08 |
| Financial and Material Worry | 6.67 (6.58,6.77) | 6.37 (6.27,6.47) | 4.23 (3.03,5.42) | 1.48e-02 | 1e-09 |
| *Psychological Well-Being* | | | | | |
| Happiness | 6.92 (6.85,6.99) | 6.88 (6.81,6.95) | 5.39 (3.95,6.82) | 9.38e-01 | 2.27e-02 |
| Life Satisfaction | 6.97 (6.90,7.05) | 6.89 (6.81,6.96) | 5.95 (4.79,7.11) | 3.93e-01 | 3.03e-02 |
| Present Life Evaluation | 6.77 (6.71,6.84) | 6.71 (6.65,6.78) | 5.53 (4.19,6.87) | 6.6e-01 | 3.73e-02 |
| Future Life Evaluation | 7.17 (7.10,7.24) | 7.14 (7.07,7.21) | 7.16 (6.18,8.14) | 4.68e-01 | 8.61e-01 |
| Optimism | 7.46 (7.38,7.54) | 7.55 (7.48,7.62) | 4.66 (2.22,7.11) | 2.1e-02 | 4.09e-03 |
| Freedom | 7.54 (7.47,7.61) | 7.58 (7.50,7.66) | 6.47 (5.14,7.81) | 7.01e-01 | 1.06e-01 |
| Peace | 0.81 (0.80,0.83) | 0.78 (0.77,0.79) | 0.40 (0.00,0.82) | 6.76e-01 | 2.41e-04 |
| Balance in Life | 0.78 (0.77,0.80) | 0.76 (0.74,0.77) | 0.27 (0.00,0.62) | 2.8e-01 | 1.33e-04 |
| Mastery | 0.86 (0.85,0.88) | 0.82 (0.81,0.83) | 0.40 (0.00,0.81) | 1.62e-03 | 7.55e-07 |
| Meaning | 7.26 (7.19,7.33) | 7.34 (7.27,7.41) | 6.21 (4.56,7.87) | 6.2e-02 | 6.87e-02 |
| Purpose | 7.12 (7.03,7.20) | 7.04 (6.96,7.13) | 3.74 (1.61,5.87) | 8.51e-01 | 2.31e-04 |
| Self-Rated Mental Health | 7.20 (7.12,7.28) | 6.92 (6.84,7.00) | 4.35 (3.01,5.68) | 1.91e-03 | 2.79e-11 |
| *Social Well-Being* | | | | | |
| Content with My Relationships | 7.45 (7.37,7.53) | 7.46 (7.38,7.54) | 6.79 (5.76,7.83) | 1.19e-01 | 2.86e-01 |
| Satisfying Relationships | 7.06 (6.97,7.14) | 7.12 (7.03,7.20) | 5.87 (4.43,7.32) | 4.66e-02 | 6.76e-02 |
| Social Support | 7.96 (7.88,8.03) | 8.22 (8.14,8.29) | 5.47 (2.73,8.22) | 1.69e-04 | 6.38e-07 |
| Intimate Friend | 0.84 (0.82,0.85) | 0.87 (0.86,0.88) | 1.00 * | 6.82e-02 | 1.6e-16 |
| Government Approval | 0.36 (0.34,0.38) | 0.32 (0.31,0.34) | 0.35 (0.00,0.82) | 4.34e-03 | 2.16e-02 |
| Political Voice | 0.54 (0.52,0.56) | 0.53 (0.51,0.55) | 0.34 (0.00,0.84) | 9.59e-02 | 1.08e-01 |
| Belonging | 6.95 (6.86,7.04) | 7.01 (6.93,7.09) | 2.35 (0.51,4.19) | 4.07e-01 | 2.17e-09 |
| City Satisfaction | 0.87 (0.85,0.88) | 0.87 (0.86,0.89) | 0.67 (0.20,1.14) | 4.18e-01 | 4.41e-01 |
| Trust | 0.30 (0.28,0.31) | 0.27 (0.25,0.29) | 0.00 * | 5.65e-02 | 1.6e-16 |
| Community Participation | 0.24 (0.22,0.26) | 0.22 (0.20,0.23) | 0.66 (0.29,1.03) | 1.96e-01 | 2.38e-03 |
| *Psychological Distress* | | | | | |
| Traumatic Distress | 0.26 (0.25,0.28) | 0.32 (0.31,0.34) | 0.31 (0.00,0.68) | 2.07e-04 | 1.16e-06 |
| Depression Symptoms | 0.15 (0.14,0.17) | 0.15 (0.14,0.17) | 0.59 (0.17,1.00) | 6.95e-01 | 3.52e-02 |
| Anxiety Symptoms | 0.18 (0.16,0.19) | 0.20 (0.19,0.22) | 0.62 (0.21,1.03) | 3.43e-01 | 1.27e-03 |
| Suffering | 0.46 (0.45,0.48) | 0.52 (0.50,0.54) | 0.54 (0.08,0.99) | 4.53e-03 | 2.14e-04 |
| *Social Distress* | | | | | |
| Loneliness | 2.93 (2.83,3.02) | 3.05 (2.96,3.15) | 6.14 (4.60,7.68) | 7.42e-01 | 4.59e-07 |
| Discrimination | 0.16 (0.14,0.17) | 0.14 (0.12,0.15) | 0.54 (0.09,0.99) | 6.69e-02 | 9.62e-03 |
| *Character & Prosocial Behavior* | | | | | |
| Promoting Good | 7.64 (7.57,7.70) | 7.73 (7.67,7.80) | 7.55 (6.46,8.65) | 2.17e-01 | 1.19e-01 |
| Delayed Gratification | 7.10 (7.02,7.17) | 6.79 (6.71,6.87) | 5.46 (4.87,6.05) | 1.97e-05 | 1.33e-15 |
| Hope | 7.65 (7.57,7.72) | 7.65 (7.59,7.72) | 6.07 (4.50,7.64) | 1.72e-01 | 4.33e-02 |
| Gratitude | 7.50 (7.42,7.58) | 7.76 (7.69,7.84) | 5.08 (3.89,6.27) | 4.82e-05 | 1.83e-11 |
| Love | 7.64 (7.57,7.72) | 7.98 (7.91,8.05) | 5.27 (3.50,7.04) | 1.47e-08 | 8.45e-13 |
| Forgiveness | 0.74 (0.72,0.76) | 0.75 (0.74,0.77) | 0.66 (0.20,1.13) | 3.99e-01 | 5.74e-01 |
| Charitable Giving | 0.44 (0.42,0.46) | 0.29 (0.27,0.30) | 0.31 (0.00,0.79) | 1.55e-15 | 1.6e-16 |
| Helping | 0.60 (0.58,0.62) | 0.43 (0.41,0.45) | 0.89 (0.73,1.04) | 1.6e-16 | 1.6e-16 |
| Volunteering | 0.26 (0.25,0.28) | 0.16 (0.15,0.17) | 0.35 (0.00,0.82) | 5.54e-12 | 1.6e-16 |
| *Physical Health & Health Behavior* | | | | | |
| Self-Rated Physical Health | 6.69 (6.61,6.76) | 6.56 (6.48,6.64) | 5.62 (4.44,6.81) | 2.2e-02 | 8.42e-03 |
| Health Limitations | 0.29 (0.27,0.30) | 0.30 (0.29,0.32) | 0.27 (0.00,0.61) | 2.38e-01 | 3.59e-01 |
| Pain | 0.37 (0.35,0.38) | 0.45 (0.44,0.47) | 0.36 (0.00,0.74) | 2.67e-08 | 1.53e-10 |
| Smoking | 3.85 (3.57,4.14) | 3.36 (3.09,3.62) | 1.90 (0.00,4.79) | 4.05e-02 | 1.57e-02 |
| Drinking | 4.20 (3.96,4.43) | 3.03 (2.77,3.29) | 3.63 (0.00,7.93) | 5.1e-07 | 6.76e-10 |
| Exercise | 2.46 (2.38,2.53) | 2.31 (2.23,2.39) | 1.90 (0.93,2.87) | 6.89e-02 | 1.7e-02 |
| *Socioeconomic Outcomes* | | | | | |
| Financial Stability | 6.41 (6.30,6.51) | 6.07 (5.96,6.18) | 3.78 (2.79,4.77) | 3.91e-02 | 5.14e-13 |
| Material Stability | 6.94 (6.84,7.04) | 6.66 (6.56,6.77) | 4.67 (2.37,6.97) | 1.34e-02 | 7.13e-05 |
| Education | 0.36 (0.35,0.38) | 0.31 (0.29,0.32) | 0.02 (0.00,0.06) | 8.3e-04 | 1.6e-16 |
| Employment | 0.61 (0.60,0.63) | 0.58 (0.56,0.60) | 0.05 (0.00,0.15) | 9.33e-02 | 1.6e-16 |
| Subjective Financial Well-Being | 0.80 (0.79,0.82) | 0.77 (0.75,0.78) | 0.62 (0.16,1.09) | 5.42e-03 | 4.94e-03 |
| Housing | 0.51 (0.49,0.53) | 0.47 (0.45,0.49) | 0.44 (0.00,0.90) | 1.33e-01 | 1.01e-02 |
| *Religion/Spirituality* | | | | | |
| Self-Reported Religion/Spirituality | 0.36 (0.35,0.38) | 0.38 (0.36,0.40) | 0.39 (0.00,0.87) | 1.34e-01 | 5.83e-01 |

| Outcome | Male | Female | Other | Male vs Female p-value | Global p-value |
|---|---|---|---|---|---|
| Religious Service Attendance | 0.08 (0.07,0.09) | 0.07 (0.06,0.08) | 0.00 * | 4.67e-02 | 1.6e-16 |
| Life after Death Belief | 0.33 (0.31,0.35) | 0.36 (0.34,0.37) | 0.40 (0.00,0.87) | 4.98e-01 | 1.69e-01 |
| Religious Experience | 0.20 (0.19,0.22) | 0.22 (0.21,0.24) | 0.12 (0.00,0.41) | 4.3e-01 | 1.35e-01 |
| Religious Reading | 0.08 (0.07,0.09) | 0.06 (0.05,0.07) | 0.00 * | 4.73e-01 | 1.6e-16 |
| Prayer-Meditation | 0.17 (0.15,0.18) | 0.17 (0.16,0.19) | 0.15 (0.00,0.44) | 4.07e-01 | 8.85e-01 |
| Belief in God | 0.55 (0.53,0.57) | 0.56 (0.54,0.58) | 0.26 (0.00,0.61) | 7.34e-01 | 7.08e-02 |
| Intrinsic Religiosity | 0.33 (0.31,0.35) | 0.33 (0.31,0.35) | 0.24 (0.00,1.09) | 6.1e-01 | 4.34e-01 |
| Religious Comfort | 0.41 (0.38,0.44) | 0.43 (0.41,0.45) | 0.51 (0.00,1.09) | 4.39e-01 | 1.67e-01 |
| Loved by God | 0.42 (0.40,0.45) | 0.44 (0.42,0.46) | 0.26 (0.00,0.73) | 8.79e-02 | 1.08e-01 |
| Spiritual Punishment | 0.13 (0.11,0.14) | 0.13 (0.11,0.14) | 0.10 (0.00,0.38) | 6.21e-01 | 4.4e-01 |
| Religious Criticism | 0.10 (0.08,0.12) | 0.09 (0.08,0.11) | 0.22 (0.00,0.62) | 5.41e-01 | 3.4e-01 |
| Evangelism | 0.33 (0.31,0.36) | 0.34 (0.32,0.36) | 0.42 (0.00,2.55) | 5.82e-01 | 2.49e-01 |
| *Family Factors* | | | | | |
| Ever Married | 0.63 (0.61,0.65) | 0.68 (0.67,0.70) | 0.03 (0.00,0.09) | 5.91e-05 | 1.6e-16 |
| Divorced | 0.06 (0.05,0.07) | 0.10 (0.09,0.11) | 0.00 * | 1.23e-05 | 1.6e-16 |
| Children | 0.38 (0.34,0.41) | 0.39 (0.36,0.42) | 0.16 (0.00,0.54) | 6.09e-01 | 2.71e-01 |

**Table S6a. Nationally representative descriptive statistics for Hong Kong**

| Characteristic | N = 3,012[1] |
|---|---|
| **Age group** | |
| 18-24 | 217 (7.2%) |
| 25-29 | 198 (6.6%) |
| 30-39 | 507 (17%) |
| 40-49 | 580 (19%) |
| 50-59 | 711 (24%) |
| 60-69 | 620 (21%) |
| 70-79 | 164 (5.5%) |
| 80 or older | 15 (0.5%) |
| (Missing) | 0 (0%) |
| **Gender** | |
| Male | 1,390 (46%) |
| Female | 1,620 (54%) |
| Other | 2 (<0.1%) |
| (Missing) | 0 (0%) |
| **Marital status** | |
| Married | 2,080 (69%) |
| Separated | 21 (0.7%) |
| Divorced | 105 (3.5%) |
| Widowed | 45 (1.5%) |
| Single, never married | 723 (24%) |
| Domestic Partner | 37 (1.2%) |
| (Missing) | 1 (<0.1%) |
| **Employment** | |
| Employed for an employer | 2,056 (68%) |
| Self-employed | 245 (8.1%) |
| Retired | 423 (14%) |
| Student | 55 (1.8%) |
| Homemaker | 114 (3.8%) |
| Unemployed and looking for a job | 62 (2.0%) |
| None of these/Other | 39 (1.3%) |
| (Missing) | 18 (0.6%) |
| **Religious service attendance** | |
| More than 1/week | 237 (7.9%) |
| 1/week | 567 (19%) |
| 1-3/month | 332 (11%) |
| A few times a year | 543 (18%) |
| Never | 1,332 (44%) |
| (Missing) | 1 (<0.1%) |
| **Education** | |
| Up to 8 years | 433 (14%) |
| 9-15 years | 2,031 (67%) |
| 16+ years | 547 (18%) |
| (Missing) | 0 (0%) |
| **Immigration** | |
| Born in this country | 2,637 (88%) |
| Born in another country | 321 (11%) |
| (Missing) | 53 (1.8%) |
| **Religious affiliation** | |
| Christianity | 757 (25%) |
| Islam | 86 (2.8%) |
| Hinduism | 20 (0.7%) |
| Buddhism | 349 (12%) |
| Judaism | 10 (0.3%) |
| Sikhism | 2 (<0.1%) |
| Baha'i | 3 (<0.1%) |
| Jainism | 1 (<0.1%) |
| Shinto | 19 (0.6%) |
| Taoism | 97 (3.2%) |
| Confucianism | 11 (0.4%) |
| Primal, Animist, or Folk religion | 27 (0.9%) |
| Spiritism | 0 (0%) |
| Umbanda, Candomble, and other African-derived religions | 0 (0%) |
| Chinese folk/traditional religion | 106 (3.5%) |
| Some other religion | 4 (0.1%) |
| No religion/Atheist/Agnostic | 1,518 (50%) |
| (Missing) | 5 (0.2%) |
| **Race/Ethnicity** | |

| Characteristic | N = 3,012[1] |
|---|---|
| (Missing) | 184 (6.1%) |
| Chinese (Cantonese) | 1,930 (64%) |
| Chinese (Chaoshan) | 201 (6.7%) |
| Chinese (Fujianese) | 117 (3.9%) |
| Chinese (Hakka) | 121 (4.0%) |
| Chinese (Other ethnicity) | 264 (8.8%) |
| Chinese (Shanghainese) | 89 (2.9%) |
| East Asian (Korean, Japanese) | 10 (0.3%) |
| Other | 4 (0.1%) |
| South Asian (Indian, Nepalese, Pakistani) | 17 (0.6%) |
| Southeast Asian (Filipino, Indonesian, Thailand) | 46 (1.5%) |
| Taiwanese | 14 (0.4%) |
| White | 15 (0.5%) |

[1]n (%)

**Table S6b. Descriptive statistics of outcome variables for Hong Kong**

| Characteristic | N = 3,012[1] |
|---|---|
| **Flourishing Index** | 7.15 (1.77) |
| (Missing) | 145 |
| **Secure Flourishing Index** | 7.10 (1.77) |
| (Missing) | 183 |
| **Happiness & Life Satisfaction** | 7.09 (1.97) |
| (Missing) | 54 |
| **Social Relationship Quality** | 7.12 (2.01) |
| (Missing) | 65 |
| **Meaning and Purpose** | 7.25 (1.91) |
| (Missing) | 36 |
| **Character & Virtue** | 7.21 (1.80) |
| (Missing) | 12 |
| **Self-Rated Health** | 7.15 (1.93) |
| (Missing) | 19 |
| **Financial and Material Worry** | 6.85 (2.37) |
| (Missing) | 46 |
| **Happiness** | 7.16 (2.00) |
| (Missing) | 5 |
| **Life Satisfaction** | 7.03 (2.08) |
| (Missing) | 51 |
| **Present Life Evaluation** | 6.85 (2.02) |
| (Missing) | 1 |
| **Future Life Evaluation** | 7.10 (1.97) |
| (Missing) | 38 |
| **Optimism** | 7.23 (2.10) |
| (Missing) | 11 |
| **Freedom** | 7.29 (2.06) |
| (Missing) | 16 |
| **Peace** | |
| Always | 923 (31%) |
| Often | 1,741 (58%) |
| Rarely | 322 (11%) |
| Never | 23 (0.8%) |
| (Missing) | 2 (<0.1%) |
| **Balance in Life** | |
| Always | 723 (24%) |
| Often | 1,684 (56%) |
| Rarely | 565 (19%) |
| Never | 29 (1.0%) |
| (Missing) | 12 (0.4%) |
| **Mastery** | |
| Always | 814 (27%) |
| Often | 1,764 (59%) |
| Rarely | 397 (13%) |
| Never | 30 (1.0%) |
| (Missing) | 7 (0.2%) |
| **Meaning** | 7.27 (1.98) |
| (Missing) | 35 |
| **Purpose** | 7.24 (2.15) |
| (Missing) | 1 |
| **Self-Rated Mental Health** | 7.17 (2.14) |
| (Missing) | 19 |
| **Content with My Relationships** | 7.18 (2.05) |
| (Missing) | 27 |
| **Satisfying Relationships** | 7.07 (2.14) |
| (Missing) | 43 |
| **Social Support** | 6.39 (2.58) |
| (Missing) | 1 |
| **Intimate Friend** | |
| Yes | 2,520 (84%) |
| No | 487 (16%) |
| (Missing) | 5 (0.2%) |
| **Government Approval** | |
| Strongly approve | 605 (20%) |
| Somewhat approve | 991 (33%) |
| Neither approve nor disapprove | 716 (24%) |
| Somewhat disapprove | 385 (13%) |
| Strongly disapprove | 311 (10%) |

| Characteristic | N = 3,012[1] |
|---|---|
| (Missing) | 4 (0.1%) |
| **Political Voice** | |
| Agree | 1,413 (47%) |
| Disagree | 874 (29%) |
| Unsure | 718 (24%) |
| (Missing) | 6 (0.2%) |
| **Belonging** | 7.40 (2.19) |
| (Missing) | 6 |
| **City Satisfaction** | |
| Satisfied | 2,107 (70%) |
| Dissatisfied | 608 (20%) |
| Unsure | 295 (9.8%) |
| (Missing) | 2 (<0.1%) |
| **Trust** | |
| All | 274 (9.1%) |
| Most | 1,162 (39%) |
| Some | 1,010 (34%) |
| Not very many | 520 (17%) |
| None | 44 (1.5%) |
| (Missing) | 2 (<0.1%) |
| **Community Participation** | |
| More than once a week | 236 (7.8%) |
| Once a week | 400 (13%) |
| One to three times a month | 404 (13%) |
| A few times a year | 619 (21%) |
| Never | 1,347 (45%) |
| (Missing) | 6 (0.2%) |
| **Traumatic Distress** | |
| A lot | 319 (11%) |
| Some | 806 (27%) |
| Not very much | 968 (32%) |
| None at all | 915 (30%) |
| (Missing) | 4 (0.1%) |
| **Suffering** | |
| A lot | 366 (12%) |
| Some | 1,082 (36%) |
| Not very much | 1,139 (38%) |
| None at all | 424 (14%) |
| (Missing) | 1 (<0.1%) |
| **Loneliness** | 3.26 (2.55) |
| (Missing) | 20 |
| **Discrimination** | |
| Always | 265 (8.8%) |
| Often | 572 (19%) |
| Rarely | 1,253 (42%) |
| Never | 917 (30%) |
| (Missing) | 5 (0.2%) |
| **Promoting Good** | 7.32 (1.93) |
| **Delayed Gratification** | 7.11 (1.96) |
| (Missing) | 12 |
| **Hope** | 7.30 (2.07) |
| (Missing) | 9 |
| **Gratitude** | 6.96 (2.22) |
| (Missing) | 10 |
| **Love** | 6.52 (2.56) |
| (Missing) | 4 |
| **Forgiveness** | |
| Always | 503 (17%) |
| Often | 1,369 (45%) |
| Rarely | 986 (33%) |
| Never | 141 (4.7%) |
| (Missing) | 13 (0.4%) |
| **Charitable Giving** | |
| Yes | 1,519 (50%) |
| No | 1,482 (49%) |
| (Missing) | 11 (0.4%) |
| **Helping** | |
| Yes | 1,948 (65%) |
| No | 1,057 (35%) |

| Characteristic | N = 3,012[1] |
|---|---|
| (Missing) | 7 (0.2%) |
| **Volunteering** | |
| Yes | 1,045 (35%) |
| No | 1,955 (65%) |
| (Missing) | 12 (0.4%) |
| **Self-Rated Physical Health** | 7.12 (1.99) |
| **Health Limitations** | |
| Yes | 625 (21%) |
| No | 2,386 (79%) |
| (Missing) | 1 (<0.1%) |
| **Pain** | |
| A lot | 318 (11%) |
| Some | 1,037 (34%) |
| Not very much | 1,096 (36%) |
| None at all | 548 (18%) |
| (Missing) | 13 (0.4%) |
| **Smoking** | 2.0 (4.2) |
| (Missing) | 31 |
| **Drinking** | 1.66 (2.86) |
| (Missing) | 30 |
| **Exercise** | |
| 0 days | 864 (29%) |
| 1 day | 500 (17%) |
| 2 days | 504 (17%) |
| 3 days | 417 (14%) |
| 4 days | 223 (7.4%) |
| 5 days | 164 (5.5%) |
| 6 days | 84 (2.8%) |
| 7 days/Every day | 249 (8.3%) |
| (Missing) | 6 (0.2%) |
| **Financial Stability** | 6.79 (2.68) |
| (Missing) | 1 |
| **Material Stability** | 6.93 (2.38) |
| (Missing) | 44 |
| **Education** | |
| Up to 8 years | 433 (14%) |
| 9-15 years | 2,031 (67%) |
| 16+ years | 547 (18%) |
| (Missing) | 0 (0%) |
| **Employment** | |
| Employed for an employer | 2,056 (68%) |
| Self-employed | 245 (8.1%) |
| Retired | 423 (14%) |
| Student | 55 (1.8%) |
| Homemaker | 114 (3.8%) |
| Unemployed and looking for a job | 62 (2.0%) |
| None of these/Other | 39 (1.3%) |
| (Missing) | 18 (0.6%) |
| **Subjective Financial Well-Being** | |
| Living comfortably on present income | 854 (28%) |
| Getting by on present income | 1,608 (53%) |
| Finding it difficult on present income | 444 (15%) |
| Finding it very difficult on present income | 104 (3.5%) |
| (Missing) | 1 (<0.1%) |
| **Housing** | |
| Someone in this household OWNS this home | 1,918 (64%) |
| Someone in this household RENTS this home | 670 (22%) |
| Both | 73 (2.4%) |
| Neither | 324 (11%) |
| Rent | 0 (0%) |
| Own | 0 (0%) |
| Something else | 0 (0%) |
| (Missing) | 26 (0.9%) |
| **Self-Reported Religion/Spirituality** | |
| Always | 501 (17%) |
| Often | 888 (29%) |
| Rarely | 948 (31%) |
| Never | 674 (22%) |
| (Missing) | 1 (<0.1%) |

| Characteristic | N = 3,012[1] |
|---|---|
| **Religious Service Attendance** | |
| More than once a week | 237 (7.9%) |
| Once a week | 567 (19%) |
| One to three times a month | 332 (11%) |
| A few times a year | 543 (18%) |
| Never | 1,332 (44%) |
| (Missing) | 1 (<0.1%) |
| **Life after Death Belief** | |
| Yes | 1,366 (45%) |
| No | 762 (25%) |
| Unsure | 881 (29%) |
| (Missing) | 3 (0.1%) |
| **Religious Experience** | |
| Yes | 1,271 (42%) |
| No | 1,736 (58%) |
| (Missing) | 5 (0.2%) |
| **Religious Reading** | |
| More than once a day | 279 (9.3%) |
| About once a day | 516 (17%) |
| Sometimes | 946 (31%) |
| Never | 1,269 (42%) |
| (Missing) | 2 (<0.1%) |
| **Prayer-Meditation** | |
| More than once a day | 377 (13%) |
| About once a day | 529 (18%) |
| Sometimes | 985 (33%) |
| Never | 1,115 (37%) |
| (Missing) | 5 (0.2%) |
| **Belief in God** | |
| One God | 728 (24%) |
| More than one god | 424 (14%) |
| An impersonal spiritual force | 635 (21%) |
| None of these | 740 (25%) |
| Unsure | 481 (16%) |
| (Missing) | 4 (0.1%) |
| **Intrinsic Religiosity** | |
| Agree | 969 (32%) |
| Disagree | 593 (20%) |
| Not relevant | 1,043 (35%) |
| Unsure | 399 (13%) |
| (Missing) | 8 (0.3%) |
| **Religious Comfort** | |
| Agree | 1,071 (36%) |
| Disagree | 539 (18%) |
| Not relevant | 1,004 (33%) |
| Unsure | 380 (13%) |
| (Missing) | 18 (0.6%) |
| **Loved by God** | |
| Agree | 906 (30%) |
| Disagree | 685 (23%) |
| Not relevant | 956 (32%) |
| Unsure | 428 (14%) |
| (Missing) | 37 (1.2%) |
| **Spiritual Punishment** | |
| Agree | 656 (22%) |
| Disagree | 885 (29%) |
| Not relevant | 984 (33%) |
| Unsure | 455 (15%) |
| (Missing) | 32 (1.1%) |
| **Religious Criticism** | |
| Agree | 506 (17%) |
| Disagree | 909 (30%) |
| Not relevant | 1,161 (39%) |
| Unsure | 414 (14%) |
| (Missing) | 21 (0.7%) |
| **Evangelism** | |
| Agree | 977 (32%) |
| Disagree | 722 (24%) |
| Not relevant | 908 (30%) |

| Characteristic | N = 3,012[1] |
|---|---|
| Unsure | 399 (13%) |
| (Missing) | 7 (0.2%) |
| **Children** | 0.56 (1.00) |
| (Missing) | 40 |

[1]Mean (SD); n (%)

**Table S6c. Demographic variation across outcomes for Hong Kong**

| Outcome | Male | Female | Other | Male vs Female p-value | Global p-value |
|---|---|---|---|---|---|
| *Flourishing Index and Domains* | | | | | |
| Flourishing Index | 7.24 (7.13,7.35) | 7.11 (6.99,7.23) | 6.48 * | 1.34e-01 | 3.63e-06 |
| Secure Flourishing Index | 7.19 (7.07,7.30) | 7.06 (6.94,7.18) | 6.42 * | 1.36e-01 | 9.74e-03 |
| Happiness & Life Satisfaction | 7.15 (7.02,7.28) | 7.05 (6.93,7.18) | 5.68 * | 3.54e-01 | 1.65e-02 |
| Social Relationship Quality | 7.22 (7.09,7.35) | 7.05 (6.91,7.19) | 6.18 * | 1.68e-01 | 4.14e-02 |
| Meaning and Purpose | 7.32 (7.20,7.44) | 7.20 (7.07,7.33) | 5.95 * | 1.82e-01 | 4.67e-04 |
| Character & Virtue | 7.26 (7.14,7.37) | 7.18 (7.05,7.30) | 7.32 * | 3.35e-01 | 6.34e-01 |
| Self-Rated Health | 7.26 (7.12,7.39) | 7.05 (6.92,7.18) | 7.27 * | 4.42e-02 | 7.9e-02 |
| Financial and Material Worry | 6.92 (6.76,7.09) | 6.82 (6.67,6.98) | 6.13 * | 3.25e-01 | 4.82e-01 |
| *Psychological Well-Being* | | | | | |
| Happiness | 7.24 (7.11,7.36) | 7.11 (6.97,7.24) | 5.45 * | 3.08e-01 | 2.91e-06 |
| Life Satisfaction | 7.06 (6.91,7.20) | 7.00 (6.87,7.14) | 5.91 * | 4.44e-01 | 2.44e-01 |
| Present Life Evaluation | 6.90 (6.76,7.04) | 6.80 (6.67,6.93) | 5.45 * | 9.45e-02 | 2.5e-04 |
| Future Life Evaluation | 7.03 (6.88,7.17) | 7.15 (7.01,7.29) | 7.00 * | 8.69e-01 | 8.75e-02 |
| Optimism | 7.19 (7.05,7.34) | 7.27 (7.13,7.40) | 6.55 * | 6.17e-01 | 1.18e-01 |
| Freedom | 7.34 (7.20,7.49) | 7.24 (7.10,7.38) | 7.73 * | 4.35e-01 | 5.93e-01 |
| Peace | 0.90 (0.88,0.92) | 0.88 (0.85,0.90) | 1.00 * | 3.61e-01 | 1.6e-16 |
| Balance in Life | 0.81 (0.78,0.83) | 0.80 (0.77,0.82) | 0.45 * | 5e-01 | 5.43e-01 |
| Mastery | 0.87 (0.84,0.89) | 0.85 (0.82,0.87) | 0.55 * | 5.24e-01 | 3.63e-01 |
| Meaning | 7.33 (7.20,7.45) | 7.22 (7.09,7.35) | 5.45 * | 1.58e-01 | 9.56e-07 |
| Purpose | 7.31 (7.17,7.46) | 7.19 (7.05,7.33) | 6.45 * | 2.86e-01 | 3.98e-02 |
| Self-Rated Mental Health | 7.28 (7.14,7.42) | 7.08 (6.93,7.23) | 6.45 * | 7.17e-02 | 1.98e-02 |
| *Social Well-Being* | | | | | |
| Content with My Relationships | 7.25 (7.11,7.38) | 7.11 (6.97,7.25) | 6.45 * | 1.66e-01 | 4.63e-02 |
| Satisfying Relationships | 7.19 (7.05,7.33) | 6.99 (6.84,7.13) | 5.91 * | 2.17e-01 | 3.53e-02 |
| Social Support | 6.45 (6.27,6.63) | 6.35 (6.17,6.53) | 5.45 * | 9.34e-02 | 2.11e-02 |
| Intimate Friend | 0.82 (0.79,0.85) | 0.85 (0.83,0.88) | 0.45 * | 3.14e-01 | 1.06e-01 |
| Government Approval | 0.55 (0.51,0.58) | 0.52 (0.48,0.55) | 0.00 * | 7.12e-01 | 1.6e-16 |
| Political Voice | 0.61 (0.57,0.65) | 0.56 (0.53,0.60) | 0.53 * | 6.35e-01 | 1.6e-16 |
| Belonging | 7.47 (7.31,7.63) | 7.34 (7.20,7.48) | 7.55 * | 7.15e-02 | 4.52e-01 |
| City Satisfaction | 0.77 (0.74,0.80) | 0.75 (0.72,0.78) | 0.00 * | 3.28e-01 | 1.6e-16 |
| Trust | 0.49 (0.45,0.52) | 0.47 (0.44,0.50) | 0.00 * | 6.32e-01 | 1.6e-16 |
| Community Participation | 0.23 (0.20,0.26) | 0.19 (0.17,0.22) | 0.00 * | 4.71e-02 | 1.6e-16 |
| *Psychological Distress* | | | | | |
| Traumatic Distress | 0.38 (0.34,0.41) | 0.37 (0.34,0.40) | 0.55 * | 9.53e-01 | 8.72e-01 |
| Depression Symptoms | 0.45 (0.41,0.48) | 0.46 (0.42,0.49) | 0.55 * | 4.54e-01 | 9.28e-01 |
| Anxiety Symptoms | 0.28 (0.25,0.32) | 0.29 (0.26,0.32) | 0.00 * | 8.45e-01 | 1.6e-16 |
| Suffering | 0.49 (0.45,0.53) | 0.47 (0.44,0.51) | 0.55 * | 6.72e-01 | 8.15e-01 |
| *Social Distress* | | | | | |
| Loneliness | 3.10 (2.93,3.28) | 3.39 (3.21,3.58) | 5.64 * | 3.4e-02 | 5.87e-03 |
| Discrimination | 0.30 (0.27,0.33) | 0.26 (0.23,0.29) | 1.00 * | 4.79e-02 | 1.6e-16 |
| *Character & Prosocial Behavior* | | | | | |
| Promoting Good | 7.39 (7.27,7.52) | 7.25 (7.12,7.38) | 8.00 * | 1.45e-01 | 1.6e-16 |
| Delayed Gratification | 7.12 (6.99,7.25) | 7.10 (6.97,7.23) | 6.64 * | 7.11e-01 | 8.89e-01 |
| Hope | 7.37 (7.24,7.51) | 7.21 (7.07,7.35) | 6.45 * | 1.45e-01 | 1.43e-02 |
| Gratitude | 6.92 (6.76,7.07) | 7.01 (6.87,7.16) | 6.91 * | 9.46e-01 | 6.87e-01 |
| Love | 6.53 (6.35,6.70) | 6.52 (6.35,6.69) | 5.55 * | 5.88e-01 | 2.37e-02 |
| Forgiveness | 0.64 (0.60,0.67) | 0.61 (0.58,0.65) | 0.00 * | 5.42e-01 | 1.6e-16 |
| Charitable Giving | 0.51 (0.48,0.55) | 0.50 (0.47,0.54) | 0.00 * | 8.16e-01 | 1.6e-16 |
| Helping | 0.63 (0.59,0.66) | 0.67 (0.63,0.70) | 0.00 * | 2.16e-01 | 1.6e-16 |
| Volunteering | 0.36 (0.33,0.39) | 0.34 (0.31,0.37) | 0.00 * | 5.64e-01 | 1.6e-16 |
| *Physical Health & Health Behavior* | | | | | |
| Self-Rated Physical Health | 7.23 (7.09,7.38) | 7.03 (6.90,7.16) | 8.09 * | 7.13e-02 | 4.54e-02 |
| Health Limitations | 0.22 (0.19,0.25) | 0.20 (0.17,0.22) | 0.00 * | 6.63e-01 | 1.6e-16 |
| Pain | 0.47 (0.43,0.50) | 0.44 (0.41,0.48) | 0.00 * | 4.94e-01 | 1.6e-16 |
| Smoking | 2.37 (2.06,2.67) | 1.69 (1.39,2.00) | 0.00 * | 3.38e-02 | 1.6e-16 |
| Drinking | 2.03 (1.81,2.24) | 1.35 (1.19,1.51) | 1.64 * | 1.17e-03 | 3.6e-06 |
| Exercise | 2.47 (2.30,2.63) | 2.05 (1.90,2.20) | 1.64 * | 1.63e-02 | 7.77e-04 |
| *Socioeconomic Outcomes* | | | | | |
| Financial Stability | 6.88 (6.69,7.07) | 6.72 (6.54,6.90) | 5.91 * | 1.94e-01 | 2.19e-01 |
| Material Stability | 6.96 (6.80,7.13) | 6.92 (6.77,7.07) | 6.36 * | 6.34e-01 | 8.03e-01 |
| Education | 0.21 (0.18,0.23) | 0.16 (0.14,0.18) | 0.00 * | 2.42e-03 | 1.6e-16 |
| Employment | 0.78 (0.75,0.82) | 0.76 (0.72,0.79) | 1.00 * | 7.12e-02 | 1.6e-16 |
| Subjective Financial Well-Being | 0.83 (0.80,0.85) | 0.81 (0.79,0.84) | 0.45 * | 1.02e-01 | 4.27e-01 |
| Housing | 0.70 (0.66,0.73) | 0.64 (0.61,0.68) | 0.55 * | 3.13e-01 | 7.11e-02 |
| *Religion/Spirituality* | | | | | |
| Self-Reported Religion/Spirituality | 0.47 (0.43,0.50) | 0.46 (0.42,0.49) | 0.55 * | 9.4e-01 | 8.85e-01 |
| Religious Service Attendance | 0.27 (0.24,0.30) | 0.27 (0.23,0.30) | 0.00 * | 7.07e-01 | 1.6e-16 |

| Outcome | Male | Female | Other | Male vs Female p-value | Global p-value |
|---|---|---|---|---|---|
| Life after Death Belief | 0.42 (0.39,0.46) | 0.48 (0.45,0.52) | 0.55 * | 2.84e-01 | 5.69e-02 |
| Religious Experience | 0.44 (0.40,0.47) | 0.41 (0.37,0.44) | 0.55 * | 3.44e-01 | 4.39e-01 |
| Religious Reading | 0.27 (0.24,0.30) | 0.26 (0.23,0.29) | 0.00 * | 9.17e-01 | 1.6e-16 |
| Prayer-Meditation | 0.31 (0.27,0.34) | 0.30 (0.27,0.33) | 0.00 * | 7.39e-01 | 1.6e-16 |
| Belief in God | 0.58 (0.54,0.62) | 0.60 (0.57,0.64) | 0.55 * | 2.68e-01 | 6.35e-01 |
| Intrinsic Religiosity | 0.51 (0.46,0.56) | 0.53 (0.46,0.60) | 0.33 * | 2.7e-01 | 1.6e-16 |
| Religious Comfort | 0.52 (0.47,0.56) | 0.56 (0.51,0.62) | 0.55 * | 2.06e-01 | 1.97e-01 |
| Loved by God | 0.45 (0.40,0.51) | 0.48 (0.42,0.53) | 0.55 * | 4.72e-01 | 6.34e-01 |
| Spiritual Punishment | 0.37 (0.31,0.42) | 0.35 (0.30,0.40) | 0.09 * | 6e-01 | 1.6e-16 |
| Religious Criticism | 0.32 (0.29,0.36) | 0.28 (0.25,0.32) | 0.11 * | 1.63e-01 | 1.6e-16 |
| Evangelism | 0.47 (0.43,0.52) | 0.47 (0.42,0.51) | 0.82 * | 4.15e-01 | 1.6e-16 |
| *Family Factors* | | | | | |
| Ever Married | 0.78 (0.75,0.81) | 0.72 (0.69,0.75) | 0.00 * | 1.68e-01 | 1.6e-16 |
| Divorced | 0.03 (0.01,0.04) | 0.04 (0.03,0.06) | 0.00 * | 6.97e-01 | 1.5e-09 |
| Children | 0.55 (0.48,0.62) | 0.57 (0.49,0.64) | 0.00 * | 6.64e-01 | 1.6e-16 |

**Table S7a. Nationally representative descriptive statistics for India**

| Characteristic | N = 12,765[1] |
|---|---|
| **Age group** | |
| 18-24 | 2,543 (20%) |
| 25-29 | 1,640 (13%) |
| 30-39 | 3,109 (24%) |
| 40-49 | 2,275 (18%) |
| 50-59 | 1,574 (12%) |
| 60-69 | 1,188 (9.3%) |
| 70-79 | 370 (2.9%) |
| 80 or older | 67 (0.5%) |
| (Missing) | 0 (0%) |
| **Gender** | |
| Male | 6,473 (51%) |
| Female | 6,292 (49%) |
| Other | 0 (0%) |
| (Missing) | 0 (0%) |
| **Marital status** | |
| Married | 9,848 (77%) |
| Separated | 45 (0.4%) |
| Divorced | 25 (0.2%) |
| Widowed | 445 (3.5%) |
| Single, never married | 2,065 (16%) |
| Domestic Partner | 269 (2.1%) |
| (Missing) | 69 (0.5%) |
| **Employment** | |
| Employed for an employer | 2,660 (21%) |
| Self-employed | 3,401 (27%) |
| Retired | 286 (2.2%) |
| Student | 532 (4.2%) |
| Homemaker | 4,221 (33%) |
| Unemployed and looking for a job | 902 (7.1%) |
| None of these/Other | 715 (5.6%) |
| (Missing) | 48 (0.4%) |
| **Religious service attendance** | |
| More than 1/week | 2,875 (23%) |
| 1/week | 3,166 (25%) |
| 1-3/month | 2,740 (21%) |
| A few times a year | 2,090 (16%) |
| Never | 1,823 (14%) |
| (Missing) | 71 (0.6%) |
| **Education** | |
| Up to 8 years | 11,422 (89%) |
| 9-15 years | 1,194 (9.4%) |
| 16+ years | 145 (1.1%) |
| (Missing) | 4 (<0.1%) |
| **Immigration** | |
| Born in this country | 12,629 (99%) |
| Born in another country | 110 (0.9%) |
| (Missing) | 26 (0.2%) |
| **Religious affiliation** | |
| Christianity | 306 (2.4%) |
| Islam | 1,555 (12%) |
| Hinduism | 10,362 (81%) |
| Buddhism | 230 (1.8%) |
| Judaism | 0 (0%) |
| Sikhism | 127 (1.0%) |
| Baha'i | 0 (0%) |
| Jainism | 10 (<0.1%) |
| Shinto | 1 (<0.1%) |
| Taoism | 0 (0%) |
| Confucianism | 0 (0%) |
| Primal, Animist, or Folk religion | 30 (0.2%) |
| Spiritism | 0 (0%) |
| Umbanda, Candomble, and other African-derived religions | 0 (0%) |
| Chinese folk/traditional religion | 0 (0%) |
| Some other religion | 67 (0.5%) |
| No religion/Atheist/Agnostic | 13 (0.1%) |
| (Missing) | 62 (0.5%) |
| **Race/Ethnicity** | |

| Characteristic | N = 12,765[1] |
|---|---|
| (Missing) | 267 (2.1%) |
| General | 3,538 (28%) |
| Other backward caste | 4,177 (33%) |
| Schedule caste | 3,599 (28%) |
| Schedule tribe | 1,185 (9.3%) |

[1]n (%)

**Table S7b. Descriptive statistics of outcome variables for India**

| Characteristic | N = 12,765[1] |
|---|---|
| **Flourishing Index** | 7.45 (2.02) |
| (Missing) | 495 |
| **Secure Flourishing Index** | 6.89 (1.89) |
| (Missing) | 523 |
| **Happiness & Life Satisfaction** | 6.7 (3.1) |
| (Missing) | 85 |
| **Social Relationship Quality** | 7.94 (2.73) |
| (Missing) | 108 |
| **Meaning and Purpose** | 7.49 (2.60) |
| (Missing) | 241 |
| **Character & Virtue** | 7.78 (2.49) |
| (Missing) | 166 |
| **Self-Rated Health** | 7.22 (2.95) |
| (Missing) | 60 |
| **Financial and Material Worry** | 4.0 (3.7) |
| (Missing) | 43 |
| **Happiness** | 6.5 (3.6) |
| (Missing) | 38 |
| **Life Satisfaction** | 7.0 (3.5) |
| (Missing) | 59 |
| **Present Life Evaluation** | 5.6 (3.6) |
| (Missing) | 133 |
| **Future Life Evaluation** | 7.30 (3.07) |
| (Missing) | 1,205 |
| **Optimism** | 8.14 (2.90) |
| (Missing) | 65 |
| **Freedom** | 8.2 (3.0) |
| (Missing) | 64 |
| **Peace** | |
| Always | 5,527 (43%) |
| Often | 2,145 (17%) |
| Rarely | 3,772 (30%) |
| Never | 1,201 (9.4%) |
| (Missing) | 121 (0.9%) |
| **Balance in Life** | |
| Always | 4,058 (32%) |
| Often | 2,365 (19%) |
| Rarely | 4,912 (38%) |
| Never | 1,243 (9.7%) |
| (Missing) | 186 (1.5%) |
| **Mastery** | |
| Always | 6,906 (54%) |
| Often | 1,948 (15%) |
| Rarely | 2,902 (23%) |
| Never | 917 (7.2%) |
| (Missing) | 91 (0.7%) |
| **Meaning** | 6.9 (3.4) |
| (Missing) | 150 |
| **Purpose** | 8.04 (2.99) |
| (Missing) | 118 |
| **Self-Rated Mental Health** | 7.4 (3.3) |
| (Missing) | 37 |
| **Content with My Relationships** | 8.0 (3.1) |
| (Missing) | 34 |
| **Satisfying Relationships** | 7.9 (3.1) |
| (Missing) | 80 |
| **Social Support** | 6.5 (3.9) |
| (Missing) | 52 |
| **Intimate Friend** | |
| Yes | 10,416 (82%) |
| No | 2,327 (18%) |
| (Missing) | 22 (0.2%) |
| **Government Approval** | |
| Strongly approve | 7,014 (55%) |
| Somewhat approve | 3,518 (28%) |
| Neither approve nor disapprove | 519 (4.1%) |
| Somewhat disapprove | 648 (5.1%) |
| Strongly disapprove | 779 (6.1%) |

| Characteristic | N = 12,765[1] |
|---|---|
| (Missing) | 286 (2.2%) |
| **Political Voice** | |
| Agree | 9,861 (77%) |
| Disagree | 2,052 (16%) |
| Unsure | 778 (6.1%) |
| (Missing) | 74 (0.6%) |
| **Belonging** | 8.4 (2.9) |
| (Missing) | 119 |
| **City Satisfaction** | |
| Satisfied | 11,830 (93%) |
| Dissatisfied | 825 (6.5%) |
| Unsure | 108 (0.8%) |
| (Missing) | 2 (<0.1%) |
| **Trust** | |
| All | 2,062 (16%) |
| Most | 2,203 (17%) |
| Some | 5,910 (46%) |
| Not very many | 1,079 (8.5%) |
| None | 1,095 (8.6%) |
| (Missing) | 416 (3.3%) |
| **Community Participation** | |
| More than once a week | 585 (4.6%) |
| Once a week | 1,102 (8.6%) |
| One to three times a month | 1,528 (12%) |
| A few times a year | 1,915 (15%) |
| Never | 7,481 (59%) |
| (Missing) | 153 (1.2%) |
| **Traumatic Distress** | |
| A lot | 3,205 (25%) |
| Some | 2,872 (22%) |
| Not very much | 1,064 (8.3%) |
| None at all | 5,537 (43%) |
| (Missing) | 87 (0.7%) |
| **Suffering** | |
| A lot | 2,610 (20%) |
| Some | 3,884 (30%) |
| Not very much | 1,256 (9.8%) |
| None at all | 4,918 (39%) |
| (Missing) | 97 (0.8%) |
| **Loneliness** | 3.8 (4.0) |
| (Missing) | 32 |
| **Discrimination** | |
| Always | 1,646 (13%) |
| Often | 1,092 (8.6%) |
| Rarely | 2,670 (21%) |
| Never | 7,161 (56%) |
| (Missing) | 196 (1.5%) |
| **Promoting Good** | 8.35 (2.68) |
| (Missing) | 69 |
| **Delayed Gratification** | 7.2 (3.5) |
| (Missing) | 114 |
| **Hope** | 8.32 (2.77) |
| (Missing) | 88 |
| **Gratitude** | 7.7 (3.1) |
| (Missing) | 209 |
| **Love** | 8.2 (3.0) |
| (Missing) | 39 |
| **Forgiveness** | |
| Always | 7,751 (61%) |
| Often | 1,745 (14%) |
| Rarely | 2,140 (17%) |
| Never | 1,016 (8.0%) |
| (Missing) | 112 (0.9%) |
| **Charitable Giving** | |
| Yes | 4,930 (39%) |
| No | 7,784 (61%) |
| (Missing) | 51 (0.4%) |
| **Helping** | |
| Yes | 7,031 (55%) |

| Characteristic | N = 12,765[1] |
|---|---|
| No | 5,591 (44%) |
| (Missing) | 144 (1.1%) |
| **Volunteering** | |
| Yes | 3,705 (29%) |
| No | 8,981 (70%) |
| (Missing) | 78 (0.6%) |
| **Self-Rated Physical Health** | 7.0 (3.4) |
| (Missing) | 29 |
| **Health Limitations** | |
| Yes | 3,591 (28%) |
| No | 9,067 (71%) |
| (Missing) | 107 (0.8%) |
| **Pain** | |
| A lot | 2,548 (20%) |
| Some | 3,875 (30%) |
| Not very much | 1,147 (9.0%) |
| None at all | 5,162 (40%) |
| (Missing) | 34 (0.3%) |
| **Smoking** | 0.66 (4.72) |
| (Missing) | 513 |
| **Drinking** | 0.36 (4.27) |
| (Missing) | 530 |
| **Exercise** | |
| 0 days | 4,304 (34%) |
| 1 day | 519 (4.1%) |
| 2 days | 584 (4.6%) |
| 3 days | 367 (2.9%) |
| 4 days | 327 (2.6%) |
| 5 days | 192 (1.5%) |
| 6 days | 118 (0.9%) |
| 7 days/Every day | 3,701 (29%) |
| (Missing) | 2,653 (21%) |
| **Financial Stability** | 4.1 (4.1) |
| (Missing) | 31 |
| **Material Stability** | 4.0 (4.1) |
| (Missing) | 17 |
| **Education** | |
| Up to 8 years | 11,422 (89%) |
| 9-15 years | 1,194 (9.4%) |
| 16+ years | 145 (1.1%) |
| (Missing) | 4 (<0.1%) |
| **Employment** | |
| Employed for an employer | 2,660 (21%) |
| Self-employed | 3,401 (27%) |
| Retired | 286 (2.2%) |
| Student | 532 (4.2%) |
| Homemaker | 4,221 (33%) |
| Unemployed and looking for a job | 902 (7.1%) |
| None of these/Other | 715 (5.6%) |
| (Missing) | 48 (0.4%) |
| **Subjective Financial Well-Being** | |
| Living comfortably on present income | 2,818 (22%) |
| Getting by on present income | 3,545 (28%) |
| Finding it difficult on present income | 3,496 (27%) |
| Finding it very difficult on present income | 2,830 (22%) |
| (Missing) | 76 (0.6%) |
| **Housing** | |
| Someone in this household OWNS this home | 10,284 (81%) |
| Someone in this household RENTS this home | 1,750 (14%) |
| Both | 140 (1.1%) |
| Neither | 484 (3.8%) |
| Rent | 0 (0%) |
| Own | 0 (0%) |
| Something else | 0 (0%) |
| (Missing) | 108 (0.8%) |
| **Self-Reported Religion/Spirituality** | |
| Always | 7,741 (61%) |
| Often | 1,406 (11%) |
| Rarely | 2,309 (18%) |

| Characteristic | N = 12,765[1] |
|---|---|
| Never | 1,216 (9.5%) |
| (Missing) | 93 (0.7%) |
| **Religious Service Attendance** | |
| More than once a week | 2,875 (23%) |
| Once a week | 3,166 (25%) |
| One to three times a month | 2,740 (21%) |
| A few times a year | 2,090 (16%) |
| Never | 1,823 (14%) |
| (Missing) | 71 (0.6%) |
| **Life after Death Belief** | |
| Yes | 6,058 (47%) |
| No | 5,378 (42%) |
| Unsure | 1,216 (9.5%) |
| (Missing) | 113 (0.9%) |
| **Religious Experience** | |
| Yes | 7,187 (56%) |
| No | 5,193 (41%) |
| (Missing) | 385 (3.0%) |
| **Religious Reading** | |
| More than once a day | 1,721 (13%) |
| About once a day | 2,896 (23%) |
| Sometimes | 5,489 (43%) |
| Never | 2,589 (20%) |
| (Missing) | 70 (0.5%) |
| **Prayer-Meditation** | |
| More than once a day | 4,331 (34%) |
| About once a day | 3,936 (31%) |
| Sometimes | 3,198 (25%) |
| Never | 1,269 (9.9%) |
| (Missing) | 32 (0.2%) |
| **Belief in God** | |
| One God | 7,212 (57%) |
| More than one god | 4,678 (37%) |
| An impersonal spiritual force | 269 (2.1%) |
| None of these | 378 (3.0%) |
| Unsure | 198 (1.6%) |
| (Missing) | 29 (0.2%) |
| **Intrinsic Religiosity** | |
| Agree | 10,891 (85%) |
| Disagree | 1,209 (9.5%) |
| Not relevant | 210 (1.6%) |
| Unsure | 405 (3.2%) |
| (Missing) | 51 (0.4%) |
| **Religious Comfort** | |
| Agree | 11,290 (88%) |
| Disagree | 1,049 (8.2%) |
| Not relevant | 146 (1.1%) |
| Unsure | 252 (2.0%) |
| (Missing) | 28 (0.2%) |
| **Loved by God** | |
| Agree | 11,550 (90%) |
| Disagree | 905 (7.1%) |
| Not relevant | 128 (1.0%) |
| Unsure | 164 (1.3%) |
| (Missing) | 18 (0.1%) |
| **Spiritual Punishment** | |
| Agree | 8,084 (63%) |
| Disagree | 4,000 (31%) |
| Not relevant | 241 (1.9%) |
| Unsure | 406 (3.2%) |
| (Missing) | 34 (0.3%) |
| **Religious Criticism** | |
| Agree | 7,864 (62%) |
| Disagree | 4,003 (31%) |
| Not relevant | 266 (2.1%) |
| Unsure | 574 (4.5%) |
| (Missing) | 57 (0.4%) |
| **Evangelism** | |
| Agree | 8,913 (70%) |

| **Characteristic** | **N = 12,765**[1] |
| --- | --- |
| Disagree | 3,093 (24%) |
| Not relevant | 334 (2.6%) |
| Unsure | 368 (2.9%) |
| (Missing) | 57 (0.4%) |
| **Children** | 1.50 (1.77) |
| (Missing) | 19 |

[1]Mean (SD); n (%)

**Table S7c. Demographic variation across outcomes for India**

| Outcome | Male | Female | Other | Male vs Female p-value | Global p-value |
|---|---|---|---|---|---|
| *Flourishing Index and Domains* | | | | | |
| Flourishing Index | 7.37 (7.30,7.44) | 7.49 (7.42,7.56) | 7.41 (7.35,7.47) | 9.68e-04 | 4.49e-03 |
| Secure Flourishing Index | 6.85 (6.78,6.91) | 6.88 (6.82,6.94) | 6.83 (6.78,6.88) | 1.34e-01 | 3.88e-01 |
| Happiness & Life Satisfaction | 6.63 (6.53,6.73) | 6.85 (6.75,6.95) | 6.76 (6.67,6.84) | 2.29e-04 | 7.25e-04 |
| Social Relationship Quality | 7.76 (7.68,7.85) | 8.11 (8.03,8.20) | 7.98 (7.91,8.05) | 3.9e-09 | 2.32e-08 |
| Meaning and Purpose | 7.37 (7.28,7.46) | 7.59 (7.50,7.67) | 7.44 (7.37,7.51) | 2.29e-03 | 1.76e-04 |
| Character & Virtue | 7.72 (7.64,7.81) | 7.83 (7.75,7.92) | 7.74 (7.66,7.81) | 1.59e-01 | 4.12e-02 |
| Self-Rated Health | 7.36 (7.26,7.45) | 7.08 (6.98,7.17) | 7.13 (7.05,7.21) | 4.04e-02 | 1.65e-05 |
| Financial and Material Worry | 4.25 (4.12,4.38) | 3.84 (3.70,3.97) | 3.94 (3.82,4.05) | 2.97e-05 | 3.44e-07 |
| *Psychological Well-Being* | | | | | |
| Happiness | 6.38 (6.27,6.50) | 6.57 (6.45,6.69) | 6.48 (6.38,6.58) | 1.98e-02 | 1.19e-02 |
| Life Satisfaction | 6.87 (6.76,6.99) | 7.13 (7.02,7.24) | 7.03 (6.95,7.12) | 3.57e-05 | 6.03e-04 |
| Present Life Evaluation | 5.40 (5.27,5.52) | 5.85 (5.73,5.96) | 5.64 (5.54,5.74) | 1.06e-04 | 5.87e-08 |
| Future Life Evaluation | 7.18 (7.08,7.29) | 7.35 (7.25,7.45) | 7.18 (7.10,7.26) | 2.6e-01 | 1.25e-02 |
| Optimism | 8.04 (7.95,8.14) | 8.24 (8.14,8.33) | 8.14 (8.05,8.22) | 1.7e-01 | 1.79e-03 |
| Freedom | 8.23 (8.13,8.32) | 8.07 (7.97,8.17) | 8.14 (8.06,8.22) | 1.73e-01 | 1.69e-02 |
| Peace | 0.60 (0.59,0.62) | 0.61 (0.59,0.62) | 0.61 (0.59,0.62) | 3.75e-01 | 4.96e-01 |
| Balance in Life | 0.51 (0.49,0.53) | 0.51 (0.49,0.53) | 0.51 (0.49,0.52) | 5.23e-01 | 9.35e-01 |
| Mastery | 0.71 (0.69,0.72) | 0.69 (0.67,0.70) | 0.70 (0.69,0.71) | 7.7e-02 | 7.75e-02 |
| Meaning | 6.73 (6.62,6.85) | 7.11 (7.01,7.21) | 6.89 (6.80,6.98) | 3.75e-05 | 1.77e-07 |
| Purpose | 8.01 (7.90,8.11) | 8.07 (7.97,8.17) | 7.99 (7.91,8.08) | 5.37e-01 | 3.8e-01 |
| Self-Rated Mental Health | 7.52 (7.42,7.62) | 7.35 (7.24,7.46) | 7.37 (7.28,7.46) | 6.71e-02 | 1.33e-02 |
| *Social Well-Being* | | | | | |
| Content with My Relationships | 7.78 (7.68,7.87) | 8.14 (8.04,8.23) | 7.98 (7.90,8.05) | 3.55e-08 | 2.17e-07 |
| Satisfying Relationships | 7.75 (7.65,7.85) | 8.09 (7.99,8.19) | 7.99 (7.91,8.06) | 1.27e-06 | 1.87e-06 |
| Social Support | 6.21 (6.08,6.35) | 6.71 (6.59,6.83) | 6.46 (6.36,6.57) | 1.03e-09 | 1.92e-08 |
| Intimate Friend | 0.82 (0.80,0.83) | 0.82 (0.80,0.83) | 0.82 (0.80,0.83) | 8.14e-01 | 9.65e-01 |
| Government Approval | 0.83 (0.82,0.84) | 0.86 (0.85,0.87) | 0.85 (0.84,0.86) | 1.1e-02 | 3.22e-04 |
| Political Voice | 0.82 (0.81,0.83) | 0.83 (0.82,0.84) | 0.83 (0.82,0.84) | 6.77e-01 | 9.99e-02 |
| Belonging | 8.50 (8.42,8.59) | 8.30 (8.20,8.39) | 8.38 (8.31,8.46) | 1.1e-01 | 8.44e-04 |
| City Satisfaction | 0.94 (0.93,0.94) | 0.93 (0.92,0.95) | 0.93 (0.92,0.94) | 2.28e-01 | 8.38e-01 |
| Trust | 0.35 (0.34,0.37) | 0.34 (0.32,0.35) | 0.35 (0.34,0.36) | 1.58e-01 | 1.17e-01 |
| Community Participation | 0.17 (0.15,0.18) | 0.10 (0.09,0.11) | 0.12 (0.12,0.13) | 1.49e-07 | <2e-16 |
| *Psychological Distress* | | | | | |
| Traumatic Distress | 0.47 (0.45,0.49) | 0.49 (0.47,0.51) | 0.48 (0.47,0.50) | 4.8e-01 | 8.43e-02 |
| Depression Symptoms | 0.49 (0.47,0.50) | 0.49 (0.48,0.51) | 0.49 (0.47,0.50) | 5.37e-01 | 5.49e-01 |
| Anxiety Symptoms | 0.33 (0.32,0.35) | 0.38 (0.37,0.40) | 0.36 (0.35,0.38) | 9.39e-05 | 7.45e-07 |
| Suffering | 0.48 (0.46,0.50) | 0.54 (0.53,0.56) | 0.53 (0.51,0.54) | 1.16e-03 | 1.25e-08 |
| *Social Distress* | | | | | |
| Loneliness | 3.89 (3.76,4.02) | 3.78 (3.66,3.91) | 3.76 (3.66,3.86) | 2.58e-01 | 2.15e-01 |
| Discrimination | 0.22 (0.21,0.24) | 0.21 (0.20,0.23) | 0.22 (0.21,0.23) | 6.43e-01 | 2.68e-01 |
| *Character & Prosocial Behavior* | | | | | |
| Promoting Good | 8.31 (8.22,8.40) | 8.39 (8.31,8.48) | 8.32 (8.24,8.39) | 4.44e-02 | 1.63e-01 |
| Delayed Gratification | 7.13 (7.02,7.25) | 7.28 (7.16,7.40) | 7.16 (7.06,7.26) | 5.89e-01 | 5.83e-02 |
| Hope | 8.33 (8.24,8.42) | 8.30 (8.20,8.39) | 8.28 (8.20,8.36) | 9.36e-01 | 5.95e-01 |
| Gratitude | 7.60 (7.50,7.70) | 7.77 (7.66,7.88) | 7.69 (7.60,7.78) | 6.36e-02 | 1.23e-02 |
| Love | 8.11 (8.01,8.21) | 8.30 (8.21,8.39) | 8.28 (8.20,8.35) | 4.11e-04 | 3.58e-03 |
| Forgiveness | 0.74 (0.73,0.76) | 0.76 (0.75,0.77) | 0.76 (0.74,0.77) | 2.18e-02 | 4.22e-02 |
| Charitable Giving | 0.39 (0.37,0.41) | 0.39 (0.36,0.41) | 0.40 (0.37,0.42) | 3.35e-01 | 7.23e-01 |
| Helping | 0.59 (0.57,0.62) | 0.52 (0.50,0.54) | 0.55 (0.53,0.57) | 8.3e-11 | 3.16e-11 |
| Volunteering | 0.32 (0.30,0.34) | 0.26 (0.24,0.29) | 0.29 (0.27,0.31) | 1.17e-05 | 3.35e-07 |
| *Physical Health & Health Behavior* | | | | | |
| Self-Rated Physical Health | 7.19 (7.08,7.30) | 6.80 (6.69,6.92) | 6.89 (6.79,6.98) | 7.23e-02 | 5.94e-07 |
| Health Limitations | 0.26 (0.25,0.27) | 0.31 (0.29,0.32) | 0.29 (0.28,0.31) | 5.19e-05 | 3.63e-07 |
| Pain | 0.46 (0.45,0.48) | 0.55 (0.53,0.56) | 0.51 (0.50,0.53) | 1.28e-06 | 3.15e-13 |
| Smoking | 1.03 (0.89,1.17) | 0.07 (0.03,0.11) | 0.55 (0.48,0.63) | <2e-16 | 1.6e-16 |
| Drinking | 0.46 (0.38,0.54) | 0.05 (0.00,0.10) | 0.25 (0.20,0.30) | 2.52e-07 | 1.6e-16 |
| Exercise | 3.54 (3.40,3.67) | 2.69 (2.57,2.81) | 3.06 (2.96,3.16) | 1.6e-16 | 1.6e-16 |
| *Socioeconomic Outcomes* | | | | | |
| Financial Stability | 4.32 (4.18,4.46) | 3.90 (3.76,4.04) | 4.02 (3.89,4.14) | 6.08e-04 | 3.85e-06 |
| Material Stability | 4.18 (4.04,4.33) | 3.78 (3.63,3.92) | 3.86 (3.74,3.98) | 4.96e-05 | 5.06e-06 |
| Education | 0.01 (0.01,0.02) | 0.01 (0.01,0.01) | 0.01 (0.01,0.01) | 1.27e-02 | 4.46e-04 |
| Employment | 0.68 (0.65,0.70) | 0.27 (0.25,0.29) | 0.48 (0.46,0.50) | 1.6e-16 | 1.6e-16 |
| Subjective Financial Well-Being | 0.53 (0.51,0.55) | 0.47 (0.45,0.49) | 0.49 (0.48,0.51) | 2.43e-03 | 1.59e-07 |
| Housing | 0.84 (0.82,0.85) | 0.81 (0.79,0.82) | 0.83 (0.81,0.84) | 1.68e-01 | 4.28e-03 |
| *Religion/Spirituality* | | | | | |
| Self-Reported Religion/Spirituality | 0.71 (0.70,0.73) | 0.73 (0.71,0.74) | 0.73 (0.72,0.74) | 3.6e-01 | 1.63e-01 |

| Outcome | Male | Female | Other | Male vs Female p-value | Global p-value |
|---|---|---|---|---|---|
| Religious Service Attendance | 0.49 (0.47,0.51) | 0.46 (0.45,0.48) | 0.49 (0.47,0.50) | 3.4e-02 | 1.33e-02 |
| Life after Death Belief | 0.47 (0.45,0.49) | 0.49 (0.47,0.50) | 0.50 (0.49,0.51) | 1.28e-01 | 2.07e-01 |
| Religious Experience | 0.58 (0.57,0.60) | 0.58 (0.56,0.59) | 0.59 (0.57,0.60) | 2.6e-01 | 5.94e-01 |
| Religious Reading | 0.36 (0.34,0.38) | 0.37 (0.35,0.38) | 0.38 (0.36,0.39) | 9e-01 | 5.4e-01 |
| Prayer-Meditation | 0.62 (0.60,0.64) | 0.68 (0.66,0.70) | 0.66 (0.65,0.68) | 1.44e-05 | 9.11e-08 |
| Belief in God | 0.94 (0.93,0.95) | 0.97 (0.96,0.97) | 0.96 (0.96,0.97) | 1.38e-04 | 5.92e-08 |
| Intrinsic Religiosity | 0.88 (0.87,0.90) | 0.91 (0.90,0.92) | 0.91 (0.90,0.91) | 5.57e-01 | 7.66e-04 |
| Religious Comfort | 0.90 (0.89,0.91) | 0.92 (0.91,0.93) | 0.92 (0.91,0.93) | 2.86e-02 | 2.65e-04 |
| Loved by God | 0.91 (0.90,0.92) | 0.94 (0.93,0.95) | 0.93 (0.93,0.94) | 1.42e-03 | 7.08e-09 |
| Spiritual Punishment | 0.65 (0.64,0.67) | 0.68 (0.67,0.70) | 0.68 (0.67,0.70) | 1.32e-01 | 3.95e-03 |
| Religious Criticism | 0.65 (0.63,0.67) | 0.67 (0.66,0.69) | 0.67 (0.66,0.68) | 2.2e-01 | 2.75e-02 |
| Evangelism | 0.73 (0.71,0.74) | 0.75 (0.73,0.76) | 0.76 (0.74,0.77) | 1.59e-01 | 8.55e-02 |
| *Family Factors* | | | | | |
| Ever Married | 0.74 (0.73,0.76) | 0.89 (0.88,0.90) | 1.00 (1.00,1.00) | 1.6e-16 | 1.6e-16 |
| Divorced | 0.00 (0.00,0.00) | 0.00 (0.00,0.00) | 0.00 (0.00,0.01) | 2.34e-01 | 9.28e-01 |
| Children | 1.43 (1.36,1.49) | 1.57 (1.51,1.62) | 1.62 (1.57,1.67) | 4.38e-03 | 1.62e-04 |

**Table S8a. Nationally representative descriptive statistics for Indonesia**

| Characteristic | N = 6,992[1] |
|---|---|
| **Age group** | |
| 18-24 | 1,216 (17%) |
| 25-29 | 849 (12%) |
| 30-39 | 1,591 (23%) |
| 40-49 | 1,576 (23%) |
| 50-59 | 1,169 (17%) |
| 60-69 | 490 (7.0%) |
| 70-79 | 83 (1.2%) |
| 80 or older | 17 (0.2%) |
| (Missing) | 0 (0%) |
| **Gender** | |
| Male | 3,461 (50%) |
| Female | 3,513 (50%) |
| Other | 7 (<0.1%) |
| (Missing) | 11 (0.2%) |
| **Marital status** | |
| Married | 4,846 (69%) |
| Separated | 81 (1.2%) |
| Divorced | 196 (2.8%) |
| Widowed | 425 (6.1%) |
| Single, never married | 1,381 (20%) |
| Domestic Partner | 18 (0.3%) |
| (Missing) | 45 (0.6%) |
| **Employment** | |
| Employed for an employer | 1,323 (19%) |
| Self-employed | 2,187 (31%) |
| Retired | 78 (1.1%) |
| Student | 272 (3.9%) |
| Homemaker | 2,138 (31%) |
| Unemployed and looking for a job | 529 (7.6%) |
| None of these/Other | 448 (6.4%) |
| (Missing) | 18 (0.3%) |
| **Religious service attendance** | |
| More than 1/week | 2,667 (38%) |
| 1/week | 2,529 (36%) |
| 1-3/month | 786 (11%) |
| A few times a year | 659 (9.4%) |
| Never | 332 (4.8%) |
| (Missing) | 18 (0.3%) |
| **Education** | |
| Up to 8 years | 3,079 (44%) |
| 9-15 years | 3,491 (50%) |
| 16+ years | 419 (6.0%) |
| (Missing) | 2 (<0.1%) |
| **Immigration** | |
| Born in this country | 6,958 (100%) |
| Born in another country | 34 (0.5%) |
| (Missing) | 0 (0%) |
| **Religious affiliation** | |
| Christianity | 504 (7.2%) |
| Islam | 6,406 (92%) |
| Hinduism | 73 (1.0%) |
| Buddhism | 3 (<0.1%) |
| Judaism | 0 (0%) |
| Sikhism | 0 (0%) |
| Baha'i | 0 (0%) |
| Jainism | 0 (0%) |
| Shinto | 0 (0%) |
| Taoism | 1 (<0.1%) |
| Confucianism | 0 (0%) |
| Primal, Animist, or Folk religion | 0 (0%) |
| Spiritism | 0 (0%) |
| Umbanda, Candomble, and other African-derived religions | 0 (0%) |
| Chinese folk/traditional religion | 0 (0%) |
| Some other religion | 1 (<0.1%) |
| No religion/Atheist/Agnostic | 0 (0%) |
| (Missing) | 4 (<0.1%) |
| **Race/Ethnicity** | |

| Characteristic | N = 6,992[1] |
|---|---|
| (Missing) | 38 (0.5%) |
| Bali | 69 (1.0%) |
| Banjar/Melayu Banjar | 320 (4.6%) |
| Batak | 165 (2.4%) |
| Betawi | 251 (3.6%) |
| Bugis | 243 (3.5%) |
| Jawa | 2,846 (41%) |
| Madura | 262 (3.7%) |
| Makasar | 91 (1.3%) |
| Minangkabau | 273 (3.9%) |
| Other | 1,262 (18%) |
| Sunda/Parahyangan | 1,172 (17%) |

[1]n (%)

**Table S8b. Descriptive statistics of outcome variables for Indonesia**

| Characteristic | N = 6,992[1] |
|---|---|
| **Flourishing Index** | 8.47 (1.34) |
| (Missing) | 156 |
| **Secure Flourishing Index** | 8.10 (1.34) |
| (Missing) | 172 |
| **Happiness & Life Satisfaction** | 8.02 (1.99) |
| (Missing) | 34 |
| **Social Relationship Quality** | 8.68 (1.65) |
| (Missing) | 48 |
| **Meaning and Purpose** | 8.70 (1.51) |
| (Missing) | 47 |
| **Character & Virtue** | 8.49 (1.66) |
| (Missing) | 54 |
| **Self-Rated Health** | 8.43 (1.71) |
| (Missing) | 39 |
| **Financial and Material Worry** | 6.3 (3.1) |
| (Missing) | 28 |
| **Happiness** | 8.04 (2.20) |
| (Missing) | 23 |
| **Life Satisfaction** | 7.99 (2.29) |
| (Missing) | 19 |
| **Present Life Evaluation** | 6.97 (2.52) |
| (Missing) | 38 |
| **Future Life Evaluation** | 8.37 (2.05) |
| (Missing) | 82 |
| **Optimism** | 9.15 (1.69) |
| (Missing) | 22 |
| **Freedom** | 8.77 (1.81) |
| (Missing) | 21 |
| **Peace** | |
| Always | 2,484 (36%) |
| Often | 2,479 (35%) |
| Rarely | 1,450 (21%) |
| Never | 556 (8.0%) |
| (Missing) | 23 (0.3%) |
| **Balance in Life** | |
| Always | 2,113 (30%) |
| Often | 2,679 (38%) |
| Rarely | 1,705 (24%) |
| Never | 459 (6.6%) |
| (Missing) | 36 (0.5%) |
| **Mastery** | |
| Always | 2,525 (36%) |
| Often | 2,871 (41%) |
| Rarely | 1,307 (19%) |
| Never | 253 (3.6%) |
| (Missing) | 36 (0.5%) |
| **Meaning** | 8.53 (1.89) |
| (Missing) | 27 |
| **Purpose** | 8.88 (1.67) |
| (Missing) | 25 |
| **Self-Rated Mental Health** | 8.58 (1.89) |
| (Missing) | 29 |
| **Content with My Relationships** | 8.78 (1.80) |
| (Missing) | 30 |
| **Satisfying Relationships** | 8.59 (1.90) |
| (Missing) | 29 |
| **Social Support** | 7.47 (2.85) |
| (Missing) | 10 |
| **Intimate Friend** | |
| Yes | 5,399 (77%) |
| No | 1,583 (23%) |
| (Missing) | 11 (0.2%) |
| **Government Approval** | |
| Strongly approve | 1,767 (25%) |
| Somewhat approve | 1,120 (16%) |
| Neither approve nor disapprove | 3,597 (51%) |
| Somewhat disapprove | 291 (4.2%) |
| Strongly disapprove | 167 (2.4%) |

| Characteristic | N = 6,992[1] |
|---|---|
| (Missing) | 51 (0.7%) |
| **Political Voice** | |
| Agree | 5,560 (80%) |
| Disagree | 355 (5.1%) |
| Unsure | 1,027 (15%) |
| (Missing) | 50 (0.7%) |
| **Belonging** | 8.78 (1.80) |
| (Missing) | 45 |
| **City Satisfaction** | |
| Satisfied | 6,302 (90%) |
| Dissatisfied | 297 (4.2%) |
| Unsure | 388 (5.5%) |
| (Missing) | 5 (<0.1%) |
| **Trust** | |
| All | 668 (9.6%) |
| Most | 2,761 (39%) |
| Some | 1,980 (28%) |
| Not very many | 1,213 (17%) |
| None | 277 (4.0%) |
| (Missing) | 92 (1.3%) |
| **Community Participation** | |
| More than once a week | 882 (13%) |
| Once a week | 1,358 (19%) |
| One to three times a month | 1,002 (14%) |
| A few times a year | 1,245 (18%) |
| Never | 2,482 (35%) |
| (Missing) | 23 (0.3%) |
| **Traumatic Distress** | |
| A lot | 421 (6.0%) |
| Some | 1,138 (16%) |
| Not very much | 2,628 (38%) |
| None at all | 2,781 (40%) |
| (Missing) | 24 (0.3%) |
| **Suffering** | |
| A lot | 570 (8.1%) |
| Some | 1,229 (18%) |
| Not very much | 2,667 (38%) |
| None at all | 2,480 (35%) |
| (Missing) | 46 (0.7%) |
| **Loneliness** | 2.5 (3.0) |
| (Missing) | 17 |
| **Discrimination** | |
| Always | 600 (8.6%) |
| Often | 861 (12%) |
| Rarely | 2,046 (29%) |
| Never | 3,449 (49%) |
| (Missing) | 36 (0.5%) |
| **Promoting Good** | 8.63 (1.80) |
| (Missing) | 32 |
| **Delayed Gratification** | 8.35 (2.02) |
| (Missing) | 43 |
| **Hope** | 9.17 (1.44) |
| (Missing) | 18 |
| **Gratitude** | 8.93 (1.76) |
| (Missing) | 43 |
| **Love** | 8.85 (1.78) |
| (Missing) | 11 |
| **Forgiveness** | |
| Always | 3,573 (51%) |
| Often | 2,358 (34%) |
| Rarely | 668 (9.6%) |
| Never | 386 (5.5%) |
| (Missing) | 7 (0.1%) |
| **Charitable Giving** | |
| Yes | 5,475 (78%) |
| No | 1,490 (21%) |
| (Missing) | 27 (0.4%) |
| **Helping** | |
| Yes | 3,458 (49%) |

| Characteristic | N = 6,992[1] |
|---|---|
| No | 3,499 (50%) |
| (Missing) | 35 (0.5%) |
| **Volunteering** | |
| Yes | 3,207 (46%) |
| No | 3,768 (54%) |
| (Missing) | 17 (0.2%) |
| **Self-Rated Physical Health** | 8.29 (1.98) |
| (Missing) | 21 |
| **Health Limitations** | |
| Yes | 1,035 (15%) |
| No | 5,891 (84%) |
| (Missing) | 66 (0.9%) |
| **Pain** | |
| A lot | 680 (9.7%) |
| Some | 1,718 (25%) |
| Not very much | 2,892 (41%) |
| None at all | 1,682 (24%) |
| (Missing) | 19 (0.3%) |
| **Smoking** | 3.9 (6.6) |
| (Missing) | 428 |
| **Drinking** | 0.15 (1.66) |
| (Missing) | 450 |
| **Exercise** | |
| 0 days | 1,482 (21%) |
| 1 day | 1,727 (25%) |
| 2 days | 696 (10.0%) |
| 3 days | 712 (10%) |
| 4 days | 152 (2.2%) |
| 5 days | 151 (2.2%) |
| 6 days | 116 (1.7%) |
| 7 days/Every day | 1,876 (27%) |
| (Missing) | 79 (1.1%) |
| **Financial Stability** | 6.1 (3.4) |
| (Missing) | 16 |
| **Material Stability** | 6.5 (3.4) |
| (Missing) | 13 |
| **Education** | |
| Up to 8 years | 3,079 (44%) |
| 9-15 years | 3,491 (50%) |
| 16+ years | 419 (6.0%) |
| (Missing) | 2 (<0.1%) |
| **Employment** | |
| Employed for an employer | 1,323 (19%) |
| Self-employed | 2,187 (31%) |
| Retired | 78 (1.1%) |
| Student | 272 (3.9%) |
| Homemaker | 2,138 (31%) |
| Unemployed and looking for a job | 529 (7.6%) |
| None of these/Other | 448 (6.4%) |
| (Missing) | 18 (0.3%) |
| **Subjective Financial Well-Being** | |
| Living comfortably on present income | 2,265 (32%) |
| Getting by on present income | 3,200 (46%) |
| Finding it difficult on present income | 951 (14%) |
| Finding it very difficult on present income | 455 (6.5%) |
| (Missing) | 122 (1.8%) |
| **Housing** | |
| Someone in this household OWNS this home | 4,282 (61%) |
| Someone in this household RENTS this home | 628 (9.0%) |
| Both | 272 (3.9%) |
| Neither | 1,696 (24%) |
| Rent | 0 (0%) |
| Own | 0 (0%) |
| Something else | 0 (0%) |
| (Missing) | 114 (1.6%) |
| **Self-Reported Religion/Spirituality** | |
| Always | 4,125 (59%) |
| Often | 1,954 (28%) |
| Rarely | 565 (8.1%) |

| Characteristic | N = 6,992[1] |
|---|---|
| Never | 319 (4.6%) |
| (Missing) | 29 (0.4%) |
| **Religious Service Attendance** | |
| More than once a week | 2,667 (38%) |
| Once a week | 2,529 (36%) |
| One to three times a month | 786 (11%) |
| A few times a year | 659 (9.4%) |
| Never | 332 (4.8%) |
| (Missing) | 18 (0.3%) |
| **Life after Death Belief** | |
| Yes | 6,616 (95%) |
| No | 144 (2.1%) |
| Unsure | 203 (2.9%) |
| (Missing) | 29 (0.4%) |
| **Religious Experience** | |
| Yes | 3,798 (54%) |
| No | 3,126 (45%) |
| (Missing) | 68 (1.0%) |
| **Religious Reading** | |
| More than once a day | 2,311 (33%) |
| About once a day | 1,812 (26%) |
| Sometimes | 2,247 (32%) |
| Never | 591 (8.5%) |
| (Missing) | 32 (0.5%) |
| **Prayer-Meditation** | |
| More than once a day | 4,673 (67%) |
| About once a day | 1,212 (17%) |
| Sometimes | 902 (13%) |
| Never | 179 (2.6%) |
| (Missing) | 26 (0.4%) |
| **Belief in God** | |
| One God | 6,625 (95%) |
| More than one god | 72 (1.0%) |
| An impersonal spiritual force | 68 (1.0%) |
| None of these | 64 (0.9%) |
| Unsure | 139 (2.0%) |
| (Missing) | 24 (0.3%) |
| **Intrinsic Religiosity** | |
| Agree | 6,522 (93%) |
| Disagree | 123 (1.8%) |
| Not relevant | 93 (1.3%) |
| Unsure | 213 (3.1%) |
| (Missing) | 40 (0.6%) |
| **Religious Comfort** | |
| Agree | 6,699 (96%) |
| Disagree | 82 (1.2%) |
| Not relevant | 62 (0.9%) |
| Unsure | 130 (1.9%) |
| (Missing) | 20 (0.3%) |
| **Loved by God** | |
| Agree | 6,321 (90%) |
| Disagree | 204 (2.9%) |
| Not relevant | 167 (2.4%) |
| Unsure | 264 (3.8%) |
| (Missing) | 36 (0.5%) |
| **Spiritual Punishment** | |
| Agree | 1,928 (28%) |
| Disagree | 3,122 (45%) |
| Not relevant | 759 (11%) |
| Unsure | 1,139 (16%) |
| (Missing) | 44 (0.6%) |
| **Religious Criticism** | |
| Agree | 1,242 (18%) |
| Disagree | 3,510 (50%) |
| Not relevant | 1,172 (17%) |
| Unsure | 1,035 (15%) |
| (Missing) | 33 (0.5%) |
| **Evangelism** | |
| Agree | 3,795 (54%) |

| **Characteristic** | **N = 6,992**[1] |
|---|---|
| Disagree | 1,591 (23%) |
| Not relevant | 776 (11%) |
| Unsure | 783 (11%) |
| (Missing) | 47 (0.7%) |
| **Children** | 1.31 (1.54) |

[1]Mean (SD); n (%)

**Table S8c. Demographic variation across outcomes for Indonesia**

| Outcome | Male | Female | Other | Male vs Female p-value | Global p-value |
|---|---|---|---|---|---|
| *Flourishing Index and Domains* | | | | | |
| Flourishing Index | 8.43 (8.36,8.50) | 8.50 (8.45,8.56) | 6.26 (2.97,9.56) | 3.85e-02 | 8.2e-03 |
| Secure Flourishing Index | 8.08 (8.01,8.16) | 8.12 (8.06,8.18) | 5.92 (2.18,9.67) | 2.39e-01 | 5.55e-02 |
| Happiness & Life Satisfaction | 7.91 (7.81,8.01) | 8.12 (8.04,8.21) | 5.42 (0.65,10.18) | 7.2e-02 | 2.78e-04 |
| Social Relationship Quality | 8.72 (8.63,8.81) | 8.66 (8.58,8.73) | 6.61 (3.89,9.34) | 5.71e-01 | 7.13e-03 |
| Meaning and Purpose | 8.67 (8.59,8.75) | 8.74 (8.67,8.80) | 6.48 (3.30,9.66) | 6.34e-02 | 9.23e-03 |
| Character & Virtue | 8.44 (8.35,8.52) | 8.55 (8.48,8.62) | 6.36 (3.01,9.71) | 2.6e-03 | 5.38e-03 |
| Self-Rated Health | 8.42 (8.33,8.50) | 8.45 (8.38,8.52) | 6.46 (3.45,9.46) | 2.8e-01 | 3.17e-02 |
| Financial and Material Worry | 6.35 (6.19,6.50) | 6.20 (6.06,6.34) | 4.22 (0.00,10.47) | 1.75e-01 | 1.48e-01 |
| *Psychological Well-Being* | | | | | |
| Happiness | 7.93 (7.82,8.04) | 8.16 (8.07,8.25) | 4.75 (0.00,12.01) | 2.55e-02 | 1.11e-03 |
| Life Satisfaction | 7.89 (7.77,8.01) | 8.09 (8.00,8.18) | 6.08 (3.53,8.64) | 3.65e-01 | 3.22e-04 |
| Present Life Evaluation | 6.75 (6.62,6.89) | 7.19 (7.08,7.30) | 3.39 (0.00,8.28) | 2.94e-05 | 6.74e-08 |
| Future Life Evaluation | 8.23 (8.12,8.33) | 8.53 (8.44,8.61) | 4.44 (0.00,11.59) | 7.63e-04 | 2.35e-06 |
| Optimism | 9.09 (9.01,9.17) | 9.22 (9.15,9.29) | 7.29 (2.59,11.98) | 4.55e-04 | 9.16e-03 |
| Freedom | 8.83 (8.75,8.92) | 8.71 (8.63,8.79) | 5.80 (3.78,7.81) | 5.18e-01 | 1.58e-08 |
| Peace | 0.71 (0.69,0.74) | 0.71 (0.69,0.73) | 0.29 (0.00,1.06) | 5.67e-01 | 1.07e-01 |
| Balance in Life | 0.68 (0.65,0.70) | 0.70 (0.68,0.72) | 0.92 (0.61,1.24) | 1.22e-02 | 8.17e-03 |
| Mastery | 0.79 (0.77,0.81) | 0.76 (0.74,0.78) | 0.75 (0.02,1.47) | 5.34e-01 | 1.29e-01 |
| Meaning | 8.42 (8.32,8.52) | 8.64 (8.57,8.71) | 6.60 (3.29,9.91) | 1.27e-03 | 1.29e-04 |
| Purpose | 8.92 (8.85,9.00) | 8.83 (8.76,8.91) | 6.36 (3.10,9.62) | 6.49e-01 | 2.35e-03 |
| Self-Rated Mental Health | 8.56 (8.47,8.65) | 8.60 (8.52,8.67) | 6.59 (3.43,9.75) | 4.21e-01 | 4.13e-02 |
| *Social Well-Being* | | | | | |
| Content with My Relationships | 8.82 (8.72,8.91) | 8.74 (8.67,8.82) | 7.16 (4.60,9.72) | 3.85e-01 | 2.23e-02 |
| Satisfying Relationships | 8.62 (8.52,8.71) | 8.57 (8.49,8.65) | 6.07 (3.01,9.13) | 8.97e-01 | 4.6e-03 |
| Social Support | 7.35 (7.20,7.51) | 7.59 (7.48,7.70) | 6.43 (3.39,9.46) | 5.39e-02 | 1.51e-02 |
| Intimate Friend | 0.76 (0.73,0.78) | 0.79 (0.77,0.81) | 0.56 (0.00,1.63) | 4.65e-02 | 2.87e-02 |
| Government Approval | 0.43 (0.41,0.46) | 0.40 (0.38,0.42) | 0.63 (0.00,1.51) | 1.59e-01 | 5.85e-02 |
| Political Voice | 0.93 (0.92,0.95) | 0.94 (0.92,0.95) | 0.98 (0.12,1.84) | 2.13e-01 | 1.6e-16 |
| Belonging | 8.84 (8.75,8.93) | 8.72 (8.65,8.79) | 6.46 (3.29,9.63) | 7.52e-01 | 1.9e-03 |
| City Satisfaction | 0.95 (0.94,0.96) | 0.96 (0.95,0.97) | 1.00 * | 7.31e-01 | 1.6e-16 |
| Trust | 0.51 (0.48,0.53) | 0.48 (0.46,0.51) | 0.75 (0.02,1.47) | 6.13e-01 | 1.51e-01 |
| Community Participation | 0.36 (0.34,0.38) | 0.28 (0.26,0.30) | 0.63 (0.00,1.51) | 5.1e-04 | 2.18e-07 |
| *Psychological Distress* | | | | | |
| Traumatic Distress | 0.22 (0.20,0.24) | 0.22 (0.21,0.24) | 0.30 (0.00,1.06) | 5.31e-01 | 9.18e-01 |
| Depression Symptoms | 0.19 (0.17,0.20) | 0.20 (0.18,0.22) | 0.11 (0.00,0.55) | 2.4e-02 | 3.78e-01 |
| Anxiety Symptoms | 0.13 (0.12,0.15) | 0.15 (0.13,0.17) | 0.11 (0.00,0.55) | 6.18e-02 | 3.19e-01 |
| Suffering | 0.25 (0.23,0.27) | 0.26 (0.25,0.28) | 0.19 (0.00,0.74) | 1.85e-01 | 6.34e-01 |
| *Social Distress* | | | | | |
| Loneliness | 2.50 (2.36,2.65) | 2.46 (2.34,2.59) | 3.93 (0.78,7.08) | 7.66e-01 | 1.91e-01 |
| Discrimination | 0.23 (0.21,0.25) | 0.19 (0.18,0.21) | 0.29 (0.00,1.06) | 6.49e-02 | 2.2e-02 |
| *Character & Prosocial Behavior* | | | | | |
| Promoting Good | 8.57 (8.48,8.67) | 8.69 (8.63,8.76) | 6.40 (2.98,9.82) | 4.06e-03 | 4.79e-03 |
| Delayed Gratification | 8.30 (8.20,8.40) | 8.40 (8.32,8.49) | 6.32 (3.04,9.60) | 1.33e-02 | 1.4e-02 |
| Hope | 9.19 (9.11,9.27) | 9.16 (9.10,9.22) | 6.83 (3.04,10.62) | 7.01e-01 | 4.8e-02 |
| Gratitude | 8.92 (8.82,9.01) | 8.95 (8.86,9.03) | 6.83 (2.83,10.83) | 3.02e-02 | 1.07e-01 |
| Love | 8.79 (8.69,8.88) | 8.92 (8.85,8.99) | 6.70 (3.32,10.09) | 3.26e-02 | 2.76e-03 |
| Forgiveness | 0.84 (0.82,0.85) | 0.86 (0.84,0.87) | 0.40 (0.00,1.32) | 1.13e-01 | 2.53e-02 |
| Charitable Giving | 0.77 (0.74,0.79) | 0.80 (0.78,0.82) | 0.82 (0.17,1.48) | 6.37e-02 | 3.22e-02 |
| Helping | 0.53 (0.51,0.55) | 0.46 (0.44,0.49) | 0.37 (0.00,1.24) | 1.47e-04 | 1.03e-04 |
| Volunteering | 0.50 (0.47,0.52) | 0.42 (0.40,0.44) | 0.75 (0.02,1.47) | 1.48e-03 | 2.41e-06 |
| *Physical Health & Health Behavior* | | | | | |
| Self-Rated Physical Health | 8.28 (8.17,8.38) | 8.30 (8.22,8.38) | 6.32 (3.07,9.56) | 2.63e-01 | 5.79e-02 |
| Health Limitations | 0.15 (0.14,0.17) | 0.15 (0.13,0.16) | 0.08 (0.00,0.39) | 4.2e-01 | 5.48e-01 |
| Pain | 0.32 (0.30,0.34) | 0.37 (0.35,0.39) | 0.37 (0.00,1.21) | 9.18e-04 | 7.07e-03 |
| Smoking | 7.29 (6.92,7.65) | 0.36 (0.26,0.45) | 0.46 (0.00,2.34) | 1.6e-16 | 1.6e-16 |
| Drinking | 0.25 (0.17,0.32) | 0.04 (0.02,0.06) | 0.46 (0.00,2.34) | 5.29e-06 | 7.92e-08 |
| Exercise | 3.22 (3.08,3.37) | 2.70 (2.58,2.83) | 2.00 (0.00,4.55) | 1.58e-04 | 1.49e-08 |
| *Socioeconomic Outcomes* | | | | | |
| Financial Stability | 6.11 (5.95,6.28) | 6.05 (5.90,6.20) | 3.95 (0.00,11.64) | 6.11e-01 | 4.73e-01 |
| Material Stability | 6.58 (6.41,6.75) | 6.35 (6.20,6.51) | 4.48 (0.00,9.36) | 4.57e-02 | 3.04e-02 |
| Education | 0.06 (0.05,0.07) | 0.06 (0.05,0.07) | 0.00 * | 5.37e-01 | 1.6e-16 |
| Employment | 0.72 (0.70,0.75) | 0.29 (0.27,0.31) | 0.19 (0.00,0.77) | 1.6e-16 | 1.6e-16 |
| Subjective Financial Well-Being | 0.79 (0.77,0.81) | 0.80 (0.78,0.82) | 0.92 (0.61,1.24) | 8.88e-01 | 2.55e-01 |
| Housing | 0.67 (0.65,0.69) | 0.65 (0.63,0.68) | 0.74 (0.07,1.40) | 4.36e-02 | 4.18e-01 |
| *Religion/Spirituality* | | | | | |
| Self-Reported Religion/Spirituality | 0.87 (0.85,0.88) | 0.88 (0.86,0.89) | 0.74 (0.07,1.40) | 6.91e-01 | 4.94e-01 |

| Outcome | Male | Female | Other | Male vs Female p-value | Global p-value |
|---|---|---|---|---|---|
| Religious Service Attendance | 0.80 (0.78,0.82) | 0.69 (0.67,0.71) | 0.92 (0.61,1.24) | 1.41e-09 | 1.37e-13 |
| Life after Death Belief | 0.95 (0.94,0.96) | 0.95 (0.94,0.96) | 1.00 * | 2.23e-01 | 1.6e-16 |
| Religious Experience | 0.57 (0.54,0.59) | 0.53 (0.51,0.55) | 0.33 (0.00,1.14) | 2.35e-01 | 3.52e-02 |
| Religious Reading | 0.57 (0.55,0.59) | 0.62 (0.60,0.64) | 0.85 (0.38,1.31) | 3.25e-02 | 6.03e-04 |
| Prayer-Meditation | 0.81 (0.79,0.83) | 0.88 (0.87,0.89) | 0.92 (0.61,1.24) | 1.17e-06 | 7.57e-09 |
| Belief in God | 0.97 (0.96,0.98) | 0.97 (0.96,0.98) | 0.92 (0.61,1.24) | 9.64e-01 | 8.35e-01 |
| Intrinsic Religiosity | 0.98 (0.97,0.98) | 0.98 (0.97,0.98) | 1.00 * | 7.78e-01 | 1.6e-16 |
| Religious Comfort | 0.99 (0.98,0.99) | 0.98 (0.98,0.99) | 1.00 * | 6.52e-01 | 8.62e-13 |
| Loved by God | 0.97 (0.96,0.98) | 0.96 (0.96,0.97) | 0.81 (0.26,1.37) | 1.18e-01 | 4.82e-01 |
| Spiritual Punishment | 0.41 (0.38,0.44) | 0.34 (0.32,0.36) | 0.27 (0.00,1.23) | 4.23e-04 | 2.03e-05 |
| Religious Criticism | 0.29 (0.26,0.31) | 0.23 (0.21,0.25) | 0.19 (0.00,1.12) | 1.56e-04 | 8.38e-05 |
| Evangelism | 0.70 (0.67,0.72) | 0.70 (0.68,0.72) | 0.36 (0.00,1.24) | 4.99e-01 | 2.46e-01 |
| *Family Factors* | | | | | |
| Ever Married | 0.74 (0.72,0.76) | 0.85 (0.84,0.87) | 0.60 (0.00,1.51) | 3.39e-10 | 1.6e-16 |
| Divorced | 0.02 (0.01,0.03) | 0.04 (0.03,0.04) | 0.00 * | 1.75e-02 | 1.6e-16 |
| Children | 1.26 (1.19,1.33) | 1.35 (1.29,1.40) | 0.73 (0.00,2.29) | 4.94e-02 | 2.44e-02 |

**Table S9a. Nationally representative descriptive statistics for Israel**

| Characteristic | N = 3,669[1] |
|---|---|
| **Age group** | |
| 18-24 | 553 (15%) |
| 25-29 | 407 (11%) |
| 30-39 | 666 (18%) |
| 40-49 | 616 (17%) |
| 50-59 | 542 (15%) |
| 60-69 | 469 (13%) |
| 70-79 | 336 (9.2%) |
| 80 or older | 79 (2.2%) |
| (Missing) | 0 (0%) |
| **Gender** | |
| Male | 1,791 (49%) |
| Female | 1,872 (51%) |
| Other | 0 (<0.1%) |
| (Missing) | 6 (0.2%) |
| **Marital status** | |
| Married | 2,056 (56%) |
| Separated | 48 (1.3%) |
| Divorced | 258 (7.0%) |
| Widowed | 212 (5.8%) |
| Single, never married | 834 (23%) |
| Domestic Partner | 193 (5.3%) |
| (Missing) | 69 (1.9%) |
| **Employment** | |
| Employed for an employer | 1,793 (49%) |
| Self-employed | 424 (12%) |
| Retired | 576 (16%) |
| Student | 388 (11%) |
| Homemaker | 211 (5.7%) |
| Unemployed and looking for a job | 148 (4.0%) |
| None of these/Other | 118 (3.2%) |
| (Missing) | 10 (0.3%) |
| **Religious service attendance** | |
| More than 1/week | 649 (18%) |
| 1/week | 495 (14%) |
| 1-3/month | 374 (10%) |
| A few times a year | 1,014 (28%) |
| Never | 1,122 (31%) |
| (Missing) | 14 (0.4%) |
| **Education** | |
| Up to 8 years | 224 (6.1%) |
| 9-15 years | 1,517 (41%) |
| 16+ years | 1,926 (52%) |
| (Missing) | 2 (<0.1%) |
| **Immigration** | |
| Born in this country | 2,796 (76%) |
| Born in another country | 868 (24%) |
| (Missing) | 5 (0.1%) |
| **Religious affiliation** | |
| Christianity | 39 (1.1%) |
| Islam | 656 (18%) |
| Hinduism | 0 (0%) |
| Buddhism | 0 (0%) |
| Judaism | 2,897 (79%) |
| Sikhism | 0 (0%) |
| Baha'i | 2 (<0.1%) |
| Jainism | 0 (0%) |
| Shinto | 0 (0%) |
| Taoism | 1 (<0.1%) |
| Confucianism | 0 (0%) |
| Primal, Animist, or Folk religion | 1 (<0.1%) |
| Spiritism | 0 (0%) |
| Umbanda, Candomble, and other African-derived religions | 0 (0%) |
| Chinese folk/traditional religion | 0 (0%) |
| Some other religion | 5 (0.1%) |
| No religion/Atheist/Agnostic | 64 (1.7%) |
| (Missing) | 4 (0.1%) |
| **Race/Ethnicity** | |

| Characteristic | N = 3,669[1] |
|---|---|
| (Missing) | 30 (0.8%) |
| Arab | 674 (18%) |
| Jewish | 2,926 (80%) |
| Other | 39 (1.1%) |

[1]n (%)

**Table S9b. Descriptive statistics of outcome variables for Israel**

| Characteristic | N = 3,669[1] |
|---|---|
| **Flourishing Index** | 8.01 (1.33) |
| (Missing) | 83 |
| **Secure Flourishing Index** | 7.88 (1.36) |
| (Missing) | 100 |
| **Happiness & Life Satisfaction** | 7.62 (1.65) |
| (Missing) | 13 |
| **Social Relationship Quality** | 7.91 (1.97) |
| (Missing) | 17 |
| **Meaning and Purpose** | 8.27 (1.67) |
| (Missing) | 28 |
| **Character & Virtue** | 7.95 (1.63) |
| (Missing) | 29 |
| **Self-Rated Health** | 8.27 (1.65) |
| (Missing) | 4 |
| **Financial and Material Worry** | 7.20 (2.35) |
| (Missing) | 17 |
| **Happiness** | 7.76 (1.65) |
| (Missing) | 4 |
| **Life Satisfaction** | 7.47 (2.00) |
| (Missing) | 10 |
| **Present Life Evaluation** | 7.33 (1.81) |
| (Missing) | 3 |
| **Future Life Evaluation** | 8.10 (1.85) |
| (Missing) | 75 |
| **Optimism** | 8.39 (1.77) |
| (Missing) | 43 |
| **Freedom** | 8.00 (1.89) |
| (Missing) | 18 |
| **Peace** | |
| Always | 1,385 (38%) |
| Often | 1,769 (48%) |
| Rarely | 443 (12%) |
| Never | 45 (1.2%) |
| (Missing) | 26 (0.7%) |
| **Balance in Life** | |
| Always | 968 (26%) |
| Often | 2,026 (55%) |
| Rarely | 561 (15%) |
| Never | 69 (1.9%) |
| (Missing) | 45 (1.2%) |
| **Mastery** | |
| Always | 1,355 (37%) |
| Often | 1,823 (50%) |
| Rarely | 413 (11%) |
| Never | 56 (1.5%) |
| (Missing) | 22 (0.6%) |
| **Meaning** | 8.09 (1.91) |
| (Missing) | 8 |
| **Purpose** | 8.44 (1.81) |
| (Missing) | 22 |
| **Self-Rated Mental Health** | 8.47 (1.80) |
| (Missing) | 2 |
| **Content with My Relationships** | 8.08 (1.94) |
| (Missing) | 2 |
| **Satisfying Relationships** | 7.73 (2.16) |
| (Missing) | 15 |
| **Social Support** | 8.55 (2.06) |
| (Missing) | 15 |
| **Intimate Friend** | |
| Yes | 3,217 (88%) |
| No | 423 (12%) |
| (Missing) | 29 (0.8%) |
| **Government Approval** | |
| Strongly approve | 655 (18%) |
| Somewhat approve | 758 (21%) |
| Neither approve nor disapprove | 807 (22%) |
| Somewhat disapprove | 576 (16%) |
| Strongly disapprove | 772 (21%) |

| Characteristic | N = 3,669[1] |
|---|---|
| (Missing) | 101 (2.8%) |
| **Political Voice** | |
| Agree | 1,170 (32%) |
| Disagree | 1,622 (44%) |
| Unsure | 850 (23%) |
| (Missing) | 28 (0.8%) |
| **Belonging** | 7.73 (2.49) |
| (Missing) | 52 |
| **City Satisfaction** | |
| Satisfied | 3,186 (87%) |
| Dissatisfied | 390 (11%) |
| Unsure | 87 (2.4%) |
| (Missing) | 7 (0.2%) |
| **Trust** | |
| All | 100 (2.7%) |
| Most | 780 (21%) |
| Some | 1,386 (38%) |
| Not very many | 1,070 (29%) |
| None | 270 (7.4%) |
| (Missing) | 64 (1.7%) |
| **Community Participation** | |
| More than once a week | 277 (7.6%) |
| Once a week | 441 (12%) |
| One to three times a month | 425 (12%) |
| A few times a year | 703 (19%) |
| Never | 1,817 (50%) |
| (Missing) | 6 (0.2%) |
| **Traumatic Distress** | |
| A lot | 420 (11%) |
| Some | 736 (20%) |
| Not very much | 1,109 (30%) |
| None at all | 1,371 (37%) |
| (Missing) | 32 (0.9%) |
| **Suffering** | |
| A lot | 214 (5.8%) |
| Some | 785 (21%) |
| Not very much | 1,218 (33%) |
| None at all | 1,438 (39%) |
| (Missing) | 13 (0.4%) |
| **Loneliness** | 2.30 (2.39) |
| (Missing) | 12 |
| **Discrimination** | |
| Always | 183 (5.0%) |
| Often | 565 (15%) |
| Rarely | 1,270 (35%) |
| Never | 1,628 (44%) |
| (Missing) | 23 (0.6%) |
| **Promoting Good** | 8.38 (1.60) |
| (Missing) | 8 |
| **Delayed Gratification** | 7.53 (2.30) |
| (Missing) | 21 |
| **Hope** | 8.40 (1.73) |
| (Missing) | 23 |
| **Gratitude** | 7.91 (2.03) |
| (Missing) | 19 |
| **Love** | 8.60 (1.66) |
| (Missing) | 7 |
| **Forgiveness** | |
| Always | 1,139 (31%) |
| Often | 1,563 (43%) |
| Rarely | 682 (19%) |
| Never | 265 (7.2%) |
| (Missing) | 21 (0.6%) |
| **Charitable Giving** | |
| Yes | 2,025 (55%) |
| No | 1,639 (45%) |
| (Missing) | 5 (0.1%) |
| **Helping** | |
| Yes | 2,316 (63%) |

| Characteristic | N = 3,669[1] |
|---|---|
| No | 1,313 (36%) |
| (Missing) | 39 (1.1%) |
| **Volunteering** | |
| Yes | 754 (21%) |
| No | 2,906 (79%) |
| (Missing) | 8 (0.2%) |
| **Self-Rated Physical Health** | 8.08 (1.89) |
| (Missing) | 1 |
| **Health Limitations** | |
| Yes | 505 (14%) |
| No | 3,132 (85%) |
| (Missing) | 32 (0.9%) |
| **Pain** | |
| A lot | 231 (6.3%) |
| Some | 671 (18%) |
| Not very much | 1,149 (31%) |
| None at all | 1,612 (44%) |
| (Missing) | 6 (0.2%) |
| **Smoking** | 3 (7) |
| (Missing) | 28 |
| **Drinking** | 1.25 (3.95) |
| (Missing) | 53 |
| **Exercise** | |
| 0 days | 1,734 (47%) |
| 1 day | 585 (16%) |
| 2 days | 607 (17%) |
| 3 days | 375 (10%) |
| 4 days | 173 (4.7%) |
| 5 days | 81 (2.2%) |
| 6 days | 26 (0.7%) |
| 7 days/Every day | 76 (2.1%) |
| (Missing) | 13 (0.4%) |
| **Financial Stability** | 7.16 (2.49) |
| (Missing) | 15 |
| **Material Stability** | 7.24 (2.53) |
| (Missing) | 6 |
| **Education** | |
| Up to 8 years | 224 (6.1%) |
| 9-15 years | 1,517 (41%) |
| 16+ years | 1,926 (52%) |
| (Missing) | 2 (<0.1%) |
| **Employment** | |
| Employed for an employer | 1,793 (49%) |
| Self-employed | 424 (12%) |
| Retired | 576 (16%) |
| Student | 388 (11%) |
| Homemaker | 211 (5.7%) |
| Unemployed and looking for a job | 148 (4.0%) |
| None of these/Other | 118 (3.2%) |
| (Missing) | 10 (0.3%) |
| **Subjective Financial Well-Being** | |
| Living comfortably on present income | 745 (20%) |
| Getting by on present income | 1,969 (54%) |
| Finding it difficult on present income | 697 (19%) |
| Finding it very difficult on present income | 188 (5.1%) |
| (Missing) | 70 (1.9%) |
| **Housing** | |
| Someone in this household OWNS this home | 2,348 (64%) |
| Someone in this household RENTS this home | 815 (22%) |
| Both | 93 (2.5%) |
| Neither | 359 (9.8%) |
| Rent | 0 (0%) |
| Own | 0 (0%) |
| Something else | 0 (0%) |
| (Missing) | 54 (1.5%) |
| **Self-Reported Religion/Spirituality** | |
| Always | 1,280 (35%) |
| Often | 1,003 (27%) |
| Rarely | 814 (22%) |

| Characteristic | N = 3,669[1] |
|---|---|
| Never | 541 (15%) |
| (Missing) | 30 (0.8%) |
| **Religious Service Attendance** | |
| More than once a week | 649 (18%) |
| Once a week | 495 (14%) |
| One to three times a month | 374 (10%) |
| A few times a year | 1,014 (28%) |
| Never | 1,122 (31%) |
| (Missing) | 14 (0.4%) |
| **Life after Death Belief** | |
| Yes | 1,985 (54%) |
| No | 796 (22%) |
| Unsure | 848 (23%) |
| (Missing) | 41 (1.1%) |
| **Religious Experience** | |
| Yes | 840 (23%) |
| No | 2,771 (76%) |
| (Missing) | 58 (1.6%) |
| **Religious Reading** | |
| More than once a day | 563 (15%) |
| About once a day | 510 (14%) |
| Sometimes | 1,264 (34%) |
| Never | 1,318 (36%) |
| (Missing) | 15 (0.4%) |
| **Prayer-Meditation** | |
| More than once a day | 834 (23%) |
| About once a day | 513 (14%) |
| Sometimes | 1,008 (27%) |
| Never | 1,302 (35%) |
| (Missing) | 12 (0.3%) |
| **Belief in God** | |
| One God | 2,630 (72%) |
| More than one god | 0 (0%) |
| An impersonal spiritual force | 0 (0%) |
| None of these | 478 (13%) |
| Unsure | 544 (15%) |
| (Missing) | 16 (0.4%) |
| **Intrinsic Religiosity** | |
| Agree | 1,686 (46%) |
| Disagree | 748 (20%) |
| Not relevant | 836 (23%) |
| Unsure | 380 (10%) |
| (Missing) | 19 (0.5%) |
| **Religious Comfort** | |
| Agree | 2,186 (60%) |
| Disagree | 537 (15%) |
| Not relevant | 687 (19%) |
| Unsure | 253 (6.9%) |
| (Missing) | 6 (0.2%) |
| **Loved by God** | |
| Agree | 2,120 (58%) |
| Disagree | 385 (10%) |
| Not relevant | 658 (18%) |
| Unsure | 481 (13%) |
| (Missing) | 26 (0.7%) |
| **Spiritual Punishment** | |
| Agree | 730 (20%) |
| Disagree | 1,694 (46%) |
| Not relevant | 704 (19%) |
| Unsure | 515 (14%) |
| (Missing) | 25 (0.7%) |
| **Religious Criticism** | |
| Agree | 383 (10%) |
| Disagree | 1,806 (49%) |
| Not relevant | 1,138 (31%) |
| Unsure | 319 (8.7%) |
| (Missing) | 22 (0.6%) |
| **Evangelism** | |
| Agree | 1,722 (47%) |

| **Characteristic** | **N = 3,669**[1] |
|---|---|
| Disagree | 831 (23%) |
| Not relevant | 920 (25%) |
| Unsure | 188 (5.1%) |
| (Missing) | 9 (0.2%) |
| **Children** | |
| 0 | 2,069 (57%) |
| 1 | 478 (13%) |
| 2 | 533 (15%) |
| 3 | 302 (8.3%) |
| 4 | 145 (4.0%) |
| 5 | 65 (1.8%) |
| 6 | 45 (1.2%) |
| 7 | 14 (0.4%) |
| 8 | 6 (0.2%) |
| (Missing) | 13 |

[1]Mean (SD); n (%)

**Table S9c. Demographic variation across outcomes for Israel**

| Outcome | Male | Female | Other | Male vs Female p-value | Global p-value |
|---|---|---|---|---|---|
| *Flourishing Index and Domains* | | | | | |
| Flourishing Index | 8.07 (7.94,8.20) | 7.93 (7.80,8.07) | 7.20 * | 6.75e-03 | 1.6e-16 |
| Secure Flourishing Index | 7.94 (7.81,8.08) | 7.79 (7.66,7.93) | 7.00 * | 7.84e-03 | 1.6e-16 |
| Happiness & Life Satisfaction | 7.70 (7.56,7.84) | 7.54 (7.39,7.68) | 7.00 * | 6.67e-02 | 1.78e-15 |
| Social Relationship Quality | 7.93 (7.76,8.10) | 7.87 (7.66,8.08) | 8.00 * | 4.3e-01 | 4.96e-01 |
| Meaning and Purpose | 8.32 (8.16,8.48) | 8.21 (8.04,8.38) | 6.50 * | 2.39e-02 | 1.6e-16 |
| Character & Virtue | 7.98 (7.82,8.13) | 7.92 (7.78,8.06) | 6.50 * | 2.43e-01 | 1.6e-16 |
| Self-Rated Health | 8.43 (8.27,8.58) | 8.13 (7.99,8.26) | 8.00 * | 1.62e-06 | 5.26e-07 |
| Financial and Material Worry | 7.31 (7.10,7.52) | 7.09 (6.90,7.29) | 6.00 * | 1.37e-01 | 1.6e-16 |
| *Psychological Well-Being* | | | | | |
| Happiness | 7.84 (7.71,7.98) | 7.68 (7.55,7.81) | 7.00 * | 1.11e-01 | 1.6e-16 |
| Life Satisfaction | 7.56 (7.39,7.72) | 7.39 (7.20,7.59) | 7.00 * | 9.72e-02 | 4.59e-09 |
| Present Life Evaluation | 7.47 (7.31,7.62) | 7.20 (7.03,7.37) | 9.00 * | 3.91e-03 | 1.6e-16 |
| Future Life Evaluation | 8.10 (7.91,8.30) | 8.04 (7.87,8.22) | 9.00 * | 6.06e-01 | 1.6e-16 |
| Optimism | 8.34 (8.19,8.49) | 8.42 (8.27,8.57) | 8.00 * | 1.46e-01 | 1.66e-07 |
| Freedom | 8.06 (7.90,8.21) | 7.93 (7.77,8.10) | 7.00 * | 1.18e-01 | 1.6e-16 |
| Peace | 0.87 (0.85,0.90) | 0.86 (0.84,0.88) | 1.00 * | 2.88e-01 | 1.6e-16 |
| Balance in Life | 0.84 (0.82,0.87) | 0.81 (0.78,0.83) | 0.00 * | 2.4e-03 | 1.6e-16 |
| Mastery | 0.88 (0.86,0.91) | 0.86 (0.83,0.88) | 1.00 * | 2e-01 | 1.6e-16 |
| Meaning | 8.13 (7.95,8.31) | 8.04 (7.85,8.23) | 7.00 * | 1.61e-02 | 1.6e-16 |
| Purpose | 8.50 (8.33,8.67) | 8.38 (8.21,8.54) | 6.00 * | 1.06e-01 | 1.6e-16 |
| Self-Rated Mental Health | 8.59 (8.43,8.75) | 8.35 (8.20,8.50) | 9.00 * | 2.06e-03 | 2.35e-12 |
| *Social Well-Being* | | | | | |
| Content with My Relationships | 8.10 (7.93,8.26) | 8.06 (7.85,8.26) | 8.00 * | 6.94e-01 | 4.99e-01 |
| Satisfying Relationships | 7.77 (7.58,7.95) | 7.69 (7.47,7.91) | 8.00 * | 2.98e-01 | 1.28e-02 |
| Social Support | 8.49 (8.34,8.65) | 8.59 (8.44,8.74) | 10.00 * | 7.32e-01 | 1.6e-16 |
| Intimate Friend | 0.87 (0.85,0.89) | 0.90 (0.88,0.92) | 1.00 * | 1.16e-01 | 1.6e-16 |
| Government Approval | 0.43 (0.39,0.47) | 0.36 (0.32,0.39) | 0.00 * | 7.8e-03 | 1.6e-16 |
| Political Voice | 0.45 (0.41,0.48) | 0.38 (0.35,0.42) | 0.00 * | 7.93e-03 | 1.6e-16 |
| Belonging | 7.66 (7.43,7.90) | 7.76 (7.58,7.93) | 10.00 * | 6.62e-01 | 1.6e-16 |
| City Satisfaction | 0.88 (0.86,0.90) | 0.90 (0.88,0.92) | 1.00 * | 3.77e-01 | 1.6e-16 |
| Trust | 0.26 (0.22,0.29) | 0.23 (0.20,0.26) | 0.00 * | 1.03e-01 | 1.6e-16 |
| Community Participation | 0.22 (0.19,0.25) | 0.18 (0.15,0.21) | 0.00 * | 2.3e-02 | 1.6e-16 |
| *Psychological Distress* | | | | | |
| Traumatic Distress | 0.28 (0.24,0.31) | 0.36 (0.32,0.39) | 0.00 * | 3.78e-05 | 1.6e-16 |
| Depression Symptoms | 0.18 (0.15,0.21) | 0.24 (0.21,0.28) | 0.00 * | 1.62e-03 | 1.6e-16 |
| Anxiety Symptoms | 0.14 (0.11,0.16) | 0.18 (0.15,0.21) | 0.00 * | 7.28e-04 | 1.6e-16 |
| Suffering | 0.24 (0.20,0.27) | 0.31 (0.27,0.34) | 0.00 * | 8.44e-04 | 1.6e-16 |
| *Social Distress* | | | | | |
| Loneliness | 2.18 (1.97,2.38) | 2.41 (2.21,2.62) | 7.00 * | 1.95e-01 | 1.6e-16 |
| Discrimination | 0.21 (0.18,0.24) | 0.20 (0.17,0.23) | 0.00 * | 9.34e-01 | 1.6e-16 |
| *Character & Prosocial Behavior* | | | | | |
| Promoting Good | 8.40 (8.23,8.56) | 8.36 (8.23,8.50) | 8.00 * | 8.39e-01 | 8.4e-07 |
| Delayed Gratification | 7.56 (7.37,7.75) | 7.48 (7.30,7.67) | 5.00 * | 9.87e-02 | 1.6e-16 |
| Hope | 8.46 (8.30,8.62) | 8.34 (8.19,8.49) | 8.00 * | 1.2e-01 | 1.02e-07 |
| Gratitude | 7.89 (7.69,8.10) | 7.91 (7.68,8.14) | 6.00 * | 8.47e-01 | 1.6e-16 |
| Love | 8.41 (8.25,8.56) | 8.77 (8.65,8.89) | 7.00 * | 3.69e-03 | 1.6e-16 |
| Forgiveness | 0.72 (0.68,0.75) | 0.76 (0.73,0.79) | 0.00 * | 9.61e-03 | 1.6e-16 |
| Charitable Giving | 0.57 (0.53,0.61) | 0.54 (0.50,0.59) | 0.00 * | 4.21e-02 | 1.6e-16 |
| Helping | 0.66 (0.62,0.69) | 0.62 (0.58,0.66) | 0.60 * | 1.27e-02 | 1.6e-16 |
| Volunteering | 0.21 (0.18,0.23) | 0.21 (0.18,0.23) | 1.00 * | 3.35e-01 | 1.6e-16 |
| *Physical Health & Health Behavior* | | | | | |
| Self-Rated Physical Health | 8.27 (8.10,8.43) | 7.90 (7.75,8.05) | 7.00 * | 2e-07 | 1.6e-16 |
| Health Limitations | 0.13 (0.10,0.16) | 0.15 (0.12,0.18) | 0.00 * | 2.62e-02 | 1.6e-16 |
| Pain | 0.21 (0.18,0.24) | 0.28 (0.25,0.31) | 0.00 * | 4.59e-05 | 1.6e-16 |
| Smoking | 4.78 (4.18,5.39) | 1.26 (1.07,1.46) | 3.00 * | 1.62e-14 | 1.6e-16 |
| Drinking | 1.65 (1.42,1.87) | 0.73 (0.60,0.87) | 4.00 * | 1.51e-06 | 1.6e-16 |
| Exercise | 1.37 (1.23,1.51) | 1.21 (1.09,1.33) | 0.00 * | 3.94e-02 | 1.6e-16 |
| *Socioeconomic Outcomes* | | | | | |
| Financial Stability | 7.27 (7.06,7.48) | 7.05 (6.85,7.25) | 6.00 * | 1.27e-01 | 1.6e-16 |
| Material Stability | 7.35 (7.12,7.58) | 7.13 (6.92,7.35) | 6.00 * | 1.94e-01 | 1.6e-16 |
| Education | 0.51 (0.48,0.55) | 0.54 (0.51,0.57) | 1.00 * | 4.28e-01 | 1.6e-16 |
| Employment | 0.64 (0.61,0.67) | 0.57 (0.54,0.61) | 1.00 * | 2.94e-03 | 1.6e-16 |
| Subjective Financial Well-Being | 0.78 (0.75,0.81) | 0.73 (0.70,0.76) | 0.00 * | 1.82e-02 | 1.6e-16 |
| Housing | 0.68 (0.65,0.71) | 0.67 (0.63,0.70) | 1.00 * | 6.74e-01 | 1.6e-16 |
| *Religion/Spirituality* | | | | | |
| Self-Reported Religion/Spirituality | 0.62 (0.58,0.66) | 0.64 (0.60,0.68) | 0.00 * | 5.12e-01 | 1.6e-16 |
| Religious Service Attendance | 0.37 (0.33,0.41) | 0.26 (0.22,0.29) | 0.00 * | 4.7e-05 | 1.6e-16 |

| Outcome | Male | Female | Other | Male vs Female p-value | Global p-value |
|---|---|---|---|---|---|
| Life after Death Belief | 0.56 (0.52,0.59) | 0.54 (0.50,0.58) | 0.00 * | 7.25e-01 | 1.6e-16 |
| Religious Experience | 0.22 (0.19,0.25) | 0.24 (0.21,0.27) | 0.00 * | 3.52e-01 | 1.6e-16 |
| Religious Reading | 0.31 (0.27,0.35) | 0.28 (0.24,0.31) | 0.00 * | 1.99e-01 | 1.6e-16 |
| Prayer-Meditation | 0.39 (0.35,0.43) | 0.35 (0.31,0.39) | 0.00 * | 2.36e-01 | 1.6e-16 |
| Belief in God | 0.71 (0.67,0.74) | 0.73 (0.69,0.77) | 0.00 * | 1.96e-01 | 1.6e-16 |
| Intrinsic Religiosity | 0.55 (0.51,0.59) | 0.58 (0.54,0.62) | 0.00 * | 1.82e-01 | 1.6e-16 |
| Religious Comfort | 0.67 (0.63,0.71) | 0.70 (0.66,0.74) | 0.00 * | 2.27e-01 | 1.6e-16 |
| Loved by God | 0.69 (0.66,0.73) | 0.71 (0.67,0.75) | 0.00 * | 8.55e-01 | 1.6e-16 |
| Spiritual Punishment | 0.29 (0.25,0.34) | 0.25 (0.21,0.29) | 0.00 * | 2.68e-02 | 1.6e-16 |
| Religious Criticism | 0.19 (0.16,0.22) | 0.18 (0.16,0.21) | 0.00 * | 5.52e-01 | 1.6e-16 |
| Evangelism | 0.59 (0.55,0.63) | 0.63 (0.59,0.67) | 0.00 * | 9.47e-02 | 1.6e-16 |
| *Family Factors* | | | | | |
| Ever Married | 0.69 (0.66,0.72) | 0.74 (0.72,0.77) | 1.00 * | 7.51e-03 | 1.6e-16 |
| Divorced | 0.05 (0.04,0.07) | 0.09 (0.07,0.11) | 0.00 * | 1.5e-02 | 1.6e-16 |
| Children | 1.03 (0.92,1.13) | 1.04 (0.95,1.13) | 2.00 * | 8.81e-01 | 1.6e-16 |

**Table S10a. Nationally representative descriptive statistics for Japan**

| Characteristic | N = 20,543[1] |
|---|---|
| **Age group** | |
| 18-24 | 1,589 (7.7%) |
| 25-29 | 806 (3.9%) |
| 30-39 | 2,851 (14%) |
| 40-49 | 3,363 (16%) |
| 50-59 | 3,770 (18%) |
| 60-69 | 4,118 (20%) |
| 70-79 | 3,554 (17%) |
| 80 or older | 493 (2.4%) |
| (Missing) | 0 (0%) |
| **Gender** | |
| Male | 9,847 (48%) |
| Female | 10,602 (52%) |
| Other | 28 (0.1%) |
| (Missing) | 66 (0.3%) |
| **Marital status** | |
| Married | 11,837 (58%) |
| Separated | 190 (0.9%) |
| Divorced | 2,126 (10%) |
| Widowed | 1,179 (5.7%) |
| Single, never married | 5,004 (24%) |
| Domestic Partner | 144 (0.7%) |
| (Missing) | 64 (0.3%) |
| **Employment** | |
| Employed for an employer | 10,853 (53%) |
| Self-employed | 1,748 (8.5%) |
| Retired | 2,535 (12%) |
| Student | 491 (2.4%) |
| Homemaker | 1,276 (6.2%) |
| Unemployed and looking for a job | 622 (3.0%) |
| None of these/Other | 2,983 (15%) |
| (Missing) | 36 (0.2%) |
| **Religious service attendance** | |
| More than 1/week | 316 (1.5%) |
| 1/week | 348 (1.7%) |
| 1-3/month | 862 (4.2%) |
| A few times a year | 3,112 (15%) |
| Never | 15,788 (77%) |
| (Missing) | 117 (0.6%) |
| **Education** | |
| Up to 8 years | 567 (2.8%) |
| 9-15 years | 14,893 (72%) |
| 16+ years | 5,083 (25%) |
| (Missing) | 0 (0%) |
| **Immigration** | |
| Born in this country | 19,548 (95%) |
| Born in another country | 158 (0.8%) |
| (Missing) | 837 (4.1%) |
| **Religious affiliation** | |
| Christianity | 381 (1.9%) |
| Islam | 10 (<0.1%) |
| Hinduism | 5 (<0.1%) |
| Buddhism | 6,709 (33%) |
| Judaism | 10 (<0.1%) |
| Sikhism | 6 (<0.1%) |
| Baha'i | 2 (<0.1%) |
| Jainism | 11 (<0.1%) |
| Shinto | 469 (2.3%) |
| Taoism | 7 (<0.1%) |
| Confucianism | 17 (<0.1%) |
| Primal, Animist, or Folk religion | 19 (<0.1%) |
| Spiritism | 0 (0%) |
| Umbanda, Candomble, and other African-derived religions | 0 (0%) |
| Chinese folk/traditional religion | 0 (0%) |
| Some other religion | 46 (0.2%) |
| No religion/Atheist/Agnostic | 12,497 (61%) |
| (Missing) | 355 (1.7%) |

[1]n (%)

**Table S10b. Descriptive statistics of outcome variables for Japan**

| Characteristic | N = 20,543[1] |
|---|---|
| **Flourishing Index** | 5.93 (1.79) |
| (Missing) | 824 |
| **Secure Flourishing Index** | 5.89 (1.79) |
| (Missing) | 936 |
| **Happiness & Life Satisfaction** | 6.14 (2.15) |
| (Missing) | 189 |
| **Social Relationship Quality** | 5.96 (2.22) |
| (Missing) | 307 |
| **Meaning and Purpose** | 5.83 (2.10) |
| (Missing) | 185 |
| **Character & Virtue** | 5.64 (1.77) |
| (Missing) | 168 |
| **Self-Rated Health** | 6.09 (2.01) |
| (Missing) | 88 |
| **Financial and Material Worry** | 5.7 (2.7) |
| (Missing) | 145 |
| **Happiness** | 6.22 (2.13) |
| (Missing) | 76 |
| **Life Satisfaction** | 6.06 (2.31) |
| (Missing) | 120 |
| **Present Life Evaluation** | 5.92 (2.14) |
| (Missing) | 79 |
| **Future Life Evaluation** | 6.02 (2.25) |
| (Missing) | 81 |
| **Optimism** | 6.74 (2.27) |
| (Missing) | 47 |
| **Freedom** | 6.43 (2.14) |
| (Missing) | 70 |
| **Peace** | |
| Always | 2,362 (11%) |
| Often | 11,513 (56%) |
| Rarely | 5,560 (27%) |
| Never | 1,000 (4.9%) |
| (Missing) | 108 (0.5%) |
| **Balance in Life** | |
| Always | 2,775 (14%) |
| Often | 11,986 (58%) |
| Rarely | 4,704 (23%) |
| Never | 982 (4.8%) |
| (Missing) | 97 (0.5%) |
| **Mastery** | |
| Always | 596 (2.9%) |
| Often | 7,403 (36%) |
| Rarely | 9,887 (48%) |
| Never | 2,489 (12%) |
| (Missing) | 168 (0.8%) |
| **Meaning** | 5.99 (2.25) |
| (Missing) | 88 |
| **Purpose** | 5.67 (2.29) |
| (Missing) | 102 |
| **Self-Rated Mental Health** | 6.21 (2.23) |
| (Missing) | 33 |
| **Content with My Relationships** | 5.99 (2.29) |
| (Missing) | 142 |
| **Satisfying Relationships** | 5.94 (2.26) |
| (Missing) | 182 |
| **Social Support** | 5.29 (2.37) |
| (Missing) | 81 |
| **Intimate Friend** | |
| Yes | 13,722 (67%) |
| No | 6,713 (33%) |
| (Missing) | 108 (0.5%) |
| **Government Approval** | |
| Strongly approve | 246 (1.2%) |
| Somewhat approve | 3,730 (18%) |
| Neither approve nor disapprove | 8,401 (41%) |
| Somewhat disapprove | 5,431 (26%) |
| Strongly disapprove | 2,638 (13%) |

| Characteristic | N = 20,543[1] |
|---|---|
| (Missing) | 97 (0.5%) |
| **Political Voice** | |
| Agree | 4,460 (22%) |
| Disagree | 8,088 (39%) |
| Unsure | 7,913 (39%) |
| (Missing) | 81 (0.4%) |
| **Belonging** | 6.03 (2.30) |
| (Missing) | 116 |
| **City Satisfaction** | |
| Satisfied | 11,464 (56%) |
| Dissatisfied | 3,651 (18%) |
| Unsure | 5,267 (26%) |
| (Missing) | 161 (0.8%) |
| **Trust** | |
| All | 128 (0.6%) |
| Most | 5,994 (29%) |
| Some | 8,826 (43%) |
| Not very many | 4,667 (23%) |
| None | 819 (4.0%) |
| (Missing) | 109 (0.5%) |
| **Community Participation** | |
| More than once a week | 872 (4.2%) |
| Once a week | 992 (4.8%) |
| One to three times a month | 1,520 (7.4%) |
| A few times a year | 2,237 (11%) |
| Never | 14,856 (72%) |
| (Missing) | 66 (0.3%) |
| **Traumatic Distress** | |
| A lot | 1,219 (5.9%) |
| Some | 4,624 (23%) |
| Not very much | 9,035 (44%) |
| None at all | 5,598 (27%) |
| (Missing) | 66 (0.3%) |
| **Suffering** | |
| A lot | 1,310 (6.4%) |
| Some | 6,296 (31%) |
| Not very much | 9,660 (47%) |
| None at all | 3,195 (16%) |
| (Missing) | 81 (0.4%) |
| **Loneliness** | 4.0 (2.7) |
| (Missing) | 39 |
| **Discrimination** | |
| Always | 426 (2.1%) |
| Often | 2,896 (14%) |
| Rarely | 9,286 (45%) |
| Never | 7,834 (38%) |
| (Missing) | 100 (0.5%) |
| **Promoting Good** | 6.12 (2.06) |
| (Missing) | 107 |
| **Delayed Gratification** | 5.16 (2.05) |
| (Missing) | 77 |
| **Hope** | 5.94 (2.23) |
| (Missing) | 113 |
| **Gratitude** | 5.81 (2.24) |
| (Missing) | 124 |
| **Love** | 5.96 (2.20) |
| (Missing) | 45 |
| **Forgiveness** | |
| Always | 1,603 (7.8%) |
| Often | 10,038 (49%) |
| Rarely | 7,075 (34%) |
| Never | 1,722 (8.4%) |
| (Missing) | 106 (0.5%) |
| **Charitable Giving** | |
| Yes | 1,954 (9.5%) |
| No | 18,460 (90%) |
| (Missing) | 128 (0.6%) |
| **Helping** | |
| Yes | 2,351 (11%) |

| Characteristic | N = 20,543[1] |
|---|---|
| No | 18,159 (88%) |
| (Missing) | 33 (0.2%) |
| **Volunteering** | |
| Yes | 1,810 (8.8%) |
| No | 18,618 (91%) |
| (Missing) | 115 (0.6%) |
| **Self-Rated Physical Health** | 5.97 (2.09) |
| (Missing) | 60 |
| **Health Limitations** | |
| Yes | 3,644 (18%) |
| No | 16,856 (82%) |
| (Missing) | 44 (0.2%) |
| **Pain** | |
| A lot | 644 (3.1%) |
| Some | 6,144 (30%) |
| Not very much | 9,077 (44%) |
| None at all | 4,615 (22%) |
| (Missing) | 63 (0.3%) |
| **Smoking** | 3 (7) |
| (Missing) | 252 |
| **Drinking** | 3.1 (7.2) |
| (Missing) | 153 |
| **Exercise** | |
| 0 days | 9,089 (44%) |
| 1 day | 2,311 (11%) |
| 2 days | 2,190 (11%) |
| 3 days | 1,845 (9.0%) |
| 4 days | 998 (4.9%) |
| 5 days | 1,177 (5.7%) |
| 6 days | 487 (2.4%) |
| 7 days/Every day | 2,408 (12%) |
| (Missing) | 38 (0.2%) |
| **Financial Stability** | 5.6 (3.0) |
| (Missing) | 71 |
| **Material Stability** | 5.9 (2.7) |
| (Missing) | 82 |
| **Education** | |
| Up to 8 years | 567 (2.8%) |
| 9-15 years | 14,893 (72%) |
| 16+ years | 5,083 (25%) |
| (Missing) | 0 (0%) |
| **Employment** | |
| Employed for an employer | 10,853 (53%) |
| Self-employed | 1,748 (8.5%) |
| Retired | 2,535 (12%) |
| Student | 491 (2.4%) |
| Homemaker | 1,276 (6.2%) |
| Unemployed and looking for a job | 622 (3.0%) |
| None of these/Other | 2,983 (15%) |
| (Missing) | 36 (0.2%) |
| **Subjective Financial Well-Being** | |
| Living comfortably on present income | 4,300 (21%) |
| Getting by on present income | 11,790 (57%) |
| Finding it difficult on present income | 2,924 (14%) |
| Finding it very difficult on present income | 1,432 (7.0%) |
| (Missing) | 98 (0.5%) |
| **Housing** | |
| Someone in this household OWNS this home | 11,084 (54%) |
| Someone in this household RENTS this home | 2,946 (14%) |
| Both | 382 (1.9%) |
| Neither | 6,003 (29%) |
| Rent | 0 (0%) |
| Own | 0 (0%) |
| Something else | 0 (0%) |
| (Missing) | 127 (0.6%) |
| **Self-Reported Religion/Spirituality** | |
| Always | 839 (4.1%) |
| Often | 3,830 (19%) |
| Rarely | 8,632 (42%) |

| Characteristic | N = 20,543[1] |
|---|---|
| Never | 7,201 (35%) |
| (Missing) | 41 (0.2%) |
| **Religious Service Attendance** | |
| More than once a week | 316 (1.5%) |
| Once a week | 348 (1.7%) |
| One to three times a month | 862 (4.2%) |
| A few times a year | 3,112 (15%) |
| Never | 15,788 (77%) |
| (Missing) | 117 (0.6%) |
| **Life after Death Belief** | |
| Yes | 4,212 (21%) |
| No | 6,693 (33%) |
| Unsure | 9,529 (46%) |
| (Missing) | 109 (0.5%) |
| **Religious Experience** | |
| Yes | 1,602 (7.8%) |
| No | 18,852 (92%) |
| (Missing) | 89 (0.4%) |
| **Religious Reading** | |
| More than once a day | 146 (0.7%) |
| About once a day | 574 (2.8%) |
| Sometimes | 2,889 (14%) |
| Never | 16,805 (82%) |
| (Missing) | 129 (0.6%) |
| **Prayer-Meditation** | |
| More than once a day | 527 (2.6%) |
| About once a day | 1,476 (7.2%) |
| Sometimes | 4,341 (21%) |
| Never | 14,138 (69%) |
| (Missing) | 61 (0.3%) |
| **Belief in God** | |
| One God | 570 (2.8%) |
| More than one god | 1,924 (9.4%) |
| An impersonal spiritual force | 1,614 (7.9%) |
| None of these | 10,129 (49%) |
| Unsure | 6,258 (30%) |
| (Missing) | 49 (0.2%) |
| **Intrinsic Religiosity** | |
| Agree | 1,532 (7.5%) |
| Disagree | 5,016 (24%) |
| Not relevant | 10,051 (49%) |
| Unsure | 3,858 (19%) |
| (Missing) | 86 (0.4%) |
| **Religious Comfort** | |
| Agree | 1,485 (7.2%) |
| Disagree | 5,744 (28%) |
| Not relevant | 9,755 (47%) |
| Unsure | 3,418 (17%) |
| (Missing) | 140 (0.7%) |
| **Loved by God** | |
| Agree | 2,150 (10%) |
| Disagree | 5,255 (26%) |
| Not relevant | 9,103 (44%) |
| Unsure | 3,832 (19%) |
| (Missing) | 203 (1.0%) |
| **Spiritual Punishment** | |
| Agree | 514 (2.5%) |
| Disagree | 7,219 (35%) |
| Not relevant | 9,281 (45%) |
| Unsure | 3,340 (16%) |
| (Missing) | 188 (0.9%) |
| **Religious Criticism** | |
| Agree | 262 (1.3%) |
| Disagree | 5,787 (28%) |
| Not relevant | 11,488 (56%) |
| Unsure | 2,839 (14%) |
| (Missing) | 167 (0.8%) |
| **Evangelism** | |
| Agree | 791 (3.8%) |

| Characteristic | N = 20,543[1] |
|---|---|
| Disagree | 5,890 (29%) |
| Not relevant | 10,895 (53%) |
| Unsure | 2,851 (14%) |
| (Missing) | 117 (0.6%) |
| **Children** | 0.41 (1.48) |
| (Missing) | 134 |

[1]Mean (SD); n (%)

**Table S10c. Demographic variation across outcomes for Japan**

| Outcome | Male | Female | Other | Male vs Female p-value | Global p-value |
|---|---|---|---|---|---|
| *Flourishing Index and Domains* | | | | | |
| Flourishing Index | 5.79 (5.75,5.83) | 6.07 (6.03,6.11) | 5.41 (4.78,6.04) | 3.94e-09 | 1.6e-16 |
| Secure Flourishing Index | 5.76 (5.72,5.80) | 6.02 (5.98,6.06) | 5.30 (4.69,5.91) | 5.78e-08 | 1.6e-16 |
| Happiness & Life Satisfaction | 5.91 (5.86,5.96) | 6.35 (6.30,6.40) | 5.76 (4.85,6.67) | 7.51e-14 | 1.6e-16 |
| Social Relationship Quality | 5.76 (5.71,5.81) | 6.15 (6.10,6.20) | 6.00 (5.12,6.88) | 1.77e-10 | 1.6e-16 |
| Meaning and Purpose | 5.68 (5.64,5.73) | 5.97 (5.93,6.02) | 5.25 (4.32,6.19) | 3.11e-06 | 1.6e-16 |
| Character & Virtue | 5.61 (5.58,5.65) | 5.67 (5.63,5.71) | 4.80 (4.09,5.52) | 3.67e-01 | 7.89e-03 |
| Self-Rated Health | 5.96 (5.92,6.01) | 6.21 (6.17,6.26) | 5.24 (4.41,6.06) | 5.94e-08 | 5.33e-15 |
| Financial and Material Worry | 5.62 (5.56,5.68) | 5.78 (5.72,5.85) | 4.73 (3.65,5.82) | 3.66e-02 | 1.3e-04 |
| *Psychological Well-Being* | | | | | |
| Happiness | 5.99 (5.94,6.04) | 6.43 (6.38,6.48) | 5.58 (4.55,6.60) | 2.95e-14 | 1.6e-16 |
| Life Satisfaction | 5.83 (5.78,5.88) | 6.27 (6.22,6.32) | 5.94 (4.97,6.91) | 4.84e-12 | 1.6e-16 |
| Present Life Evaluation | 5.73 (5.68,5.77) | 6.11 (6.06,6.16) | 5.63 (4.82,6.44) | 3.72e-11 | 1.6e-16 |
| Future Life Evaluation | 5.83 (5.78,5.88) | 6.20 (6.15,6.25) | 6.20 (5.20,7.20) | 4.15e-09 | 1.6e-16 |
| Optimism | 6.54 (6.49,6.60) | 6.92 (6.87,6.97) | 6.17 (5.16,7.18) | 1.86e-09 | 1.6e-16 |
| Freedom | 6.25 (6.21,6.30) | 6.59 (6.54,6.64) | 6.30 (5.42,7.18) | 3.91e-09 | 1.6e-16 |
| Peace | 0.64 (0.63,0.65) | 0.71 (0.70,0.72) | 0.46 (0.26,0.66) | 7.08e-08 | 1.6e-16 |
| Balance in Life | 0.69 (0.68,0.70) | 0.75 (0.74,0.76) | 0.43 (0.23,0.63) | 2.77e-07 | 1.6e-16 |
| Mastery | 0.39 (0.38,0.40) | 0.40 (0.39,0.41) | 0.21 (0.06,0.36) | 5.05e-01 | 2.35e-02 |
| Meaning | 5.79 (5.74,5.84) | 6.18 (6.13,6.23) | 5.78 (4.62,6.94) | 2.07e-10 | 1.6e-16 |
| Purpose | 5.58 (5.53,5.63) | 5.77 (5.72,5.82) | 4.73 (3.84,5.62) | 2.26e-02 | 1.15e-07 |
| Self-Rated Mental Health | 6.08 (6.03,6.13) | 6.33 (6.28,6.38) | 5.24 (4.36,6.12) | 8.06e-06 | 1.06e-12 |
| *Social Well-Being* | | | | | |
| Content with My Relationships | 5.78 (5.73,5.83) | 6.18 (6.12,6.23) | 5.86 (4.98,6.74) | 1.15e-10 | 1.6e-16 |
| Satisfying Relationships | 5.74 (5.69,5.79) | 6.12 (6.07,6.17) | 6.14 (5.12,7.16) | 2.57e-09 | 1.6e-16 |
| Social Support | 5.01 (4.96,5.07) | 5.55 (5.50,5.60) | 5.07 (4.11,6.03) | 1.6e-16 | 1.6e-16 |
| Intimate Friend | 0.62 (0.61,0.63) | 0.72 (0.71,0.73) | 0.54 (0.33,0.74) | 3.33e-16 | 1.6e-16 |
| Government Approval | 0.22 (0.21,0.23) | 0.17 (0.16,0.18) | 0.09 (0.00,0.18) | 1.85e-09 | 1.2e-12 |
| Political Voice | 0.34 (0.33,0.35) | 0.34 (0.33,0.35) | 0.29 (0.08,0.50) | 8.4e-01 | 7.21e-01 |
| Belonging | 5.96 (5.91,6.01) | 6.10 (6.05,6.15) | 5.13 (4.16,6.10) | 1.69e-01 | 1.6e-04 |
| City Satisfaction | 0.70 (0.69,0.72) | 0.75 (0.74,0.76) | 0.59 (0.38,0.80) | 5.28e-04 | 2.27e-11 |
| Trust | 0.28 (0.27,0.29) | 0.31 (0.30,0.33) | 0.23 (0.04,0.43) | 2.41e-02 | 2.08e-04 |
| Community Participation | 0.08 (0.08,0.09) | 0.10 (0.09,0.11) | 0.03 (0.00,0.07) | 1.14e-01 | 1.53e-05 |
| *Psychological Distress* | | | | | |
| Traumatic Distress | 0.28 (0.27,0.29) | 0.29 (0.28,0.30) | 0.36 (0.16,0.55) | 9.52e-01 | 4.57e-01 |
| Depression Symptoms | 0.23 (0.22,0.23) | 0.21 (0.20,0.22) | 0.42 (0.22,0.62) | 2.51e-03 | 1.82e-03 |
| Anxiety Symptoms | 0.18 (0.17,0.19) | 0.19 (0.18,0.20) | 0.34 (0.16,0.51) | 6.08e-01 | 1.42e-01 |
| Suffering | 0.38 (0.37,0.39) | 0.37 (0.36,0.38) | 0.47 (0.26,0.67) | 3.39e-01 | 3e-01 |
| *Social Distress* | | | | | |
| Loneliness | 4.15 (4.10,4.21) | 3.90 (3.84,3.96) | 4.28 (3.16,5.40) | 1.06e-04 | 1.69e-08 |
| Discrimination | 0.17 (0.16,0.18) | 0.15 (0.15,0.16) | 0.45 (0.25,0.65) | 2.54e-01 | 2.31e-04 |
| *Character & Prosocial Behavior* | | | | | |
| Promoting Good | 6.02 (5.98,6.07) | 6.22 (6.18,6.27) | 5.40 (4.47,6.33) | 2.73e-04 | 3.65e-09 |
| Delayed Gratification | 5.21 (5.16,5.25) | 5.12 (5.07,5.16) | 4.20 (3.42,4.99) | 3.64e-02 | 1.17e-03 |
| Hope | 5.88 (5.83,5.93) | 6.01 (5.96,6.06) | 5.25 (4.30,6.21) | 3.19e-01 | 2.52e-04 |
| Gratitude | 5.55 (5.50,5.60) | 6.05 (6.00,6.10) | 5.78 (4.89,6.67) | 1.6e-16 | 1.6e-16 |
| Love | 5.61 (5.56,5.66) | 6.28 (6.23,6.33) | 5.73 (4.97,6.48) | 1.6e-16 | 1.6e-16 |
| Forgiveness | 0.54 (0.53,0.55) | 0.59 (0.58,0.61) | 0.54 (0.34,0.74) | 1.16e-04 | 7.71e-10 |
| Charitable Giving | 0.10 (0.09,0.11) | 0.09 (0.09,0.10) | 0.14 (0.00,0.29) | 1.74e-01 | 3.86e-01 |
| Helping | 0.11 (0.11,0.12) | 0.11 (0.11,0.12) | 0.12 (0.00,0.25) | 1.85e-01 | 9.87e-01 |
| Volunteering | 0.10 (0.09,0.11) | 0.08 (0.07,0.08) | 0.03 (0.00,0.08) | 1.54e-07 | 1.42e-06 |
| *Physical Health & Health Behavior* | | | | | |
| Self-Rated Physical Health | 5.85 (5.80,5.89) | 6.10 (6.05,6.14) | 5.23 (4.36,6.11) | 1.36e-08 | 1.67e-13 |
| Health Limitations | 0.18 (0.18,0.19) | 0.17 (0.16,0.18) | 0.35 (0.17,0.53) | 4.2e-02 | 1.06e-02 |
| Pain | 0.32 (0.31,0.33) | 0.34 (0.33,0.35) | 0.46 (0.26,0.66) | 6.16e-01 | 1.21e-02 |
| Smoking | 4.39 (4.22,4.57) | 1.65 (1.54,1.77) | 1.00 (0.00,2.12) | 1.6e-16 | 1.6e-16 |
| Drinking | 4.23 (4.07,4.39) | 1.81 (1.71,1.92) | 1.47 (0.30,2.64) | 1.6e-16 | 1.6e-16 |
| Exercise | 2.18 (2.12,2.24) | 1.92 (1.86,1.97) | 1.90 (1.09,2.71) | 2.21e-08 | 3.69e-10 |
| *Socioeconomic Outcomes* | | | | | |
| Financial Stability | 5.48 (5.41,5.54) | 5.62 (5.55,5.69) | 4.79 (3.70,5.89) | 1.28e-01 | 4.38e-03 |
| Material Stability | 5.76 (5.70,5.82) | 5.95 (5.89,6.01) | 4.68 (3.47,5.88) | 1.34e-02 | 1.16e-05 |
| Education | 0.23 (0.22,0.24) | 0.26 (0.25,0.27) | 0.29 (0.11,0.47) | 1.7e-02 | 4.85e-06 |
| Employment | 0.71 (0.70,0.72) | 0.53 (0.52,0.54) | 0.59 (0.39,0.79) | 1.6e-16 | 1.6e-16 |
| Subjective Financial Well-Being | 0.79 (0.78,0.80) | 0.79 (0.78,0.80) | 0.69 (0.51,0.86) | 4.52e-01 | 4.75e-01 |
| Housing | 0.56 (0.55,0.57) | 0.57 (0.55,0.58) | 0.34 (0.14,0.54) | 5.13e-01 | 4.57e-02 |
| *Religion/Spirituality* | | | | | |
| Self-Reported Religion/Spirituality | 0.22 (0.21,0.23) | 0.24 (0.23,0.25) | 0.31 (0.13,0.49) | 4.39e-01 | 8.92e-03 |

| Outcome | Male | Female | Other | Male vs Female p-value | Global p-value |
|---|---|---|---|---|---|
| Religious Service Attendance | 0.04 (0.03,0.04) | 0.03 (0.02,0.03) | 0.04 (0.00,0.11) | 1.05e-03 | 2.72e-03 |
| Life after Death Belief | 0.18 (0.17,0.19) | 0.23 (0.22,0.24) | 0.27 (0.09,0.46) | 2.99e-03 | 3.56e-10 |
| Religious Experience | 0.08 (0.07,0.08) | 0.08 (0.07,0.09) | 0.16 (0.04,0.28) | 2.33e-01 | 3.86e-01 |
| Religious Reading | 0.04 (0.04,0.05) | 0.03 (0.03,0.03) | 0.05 (0.00,0.12) | 5.67e-04 | 4.64e-03 |
| Prayer-Meditation | 0.10 (0.09,0.10) | 0.10 (0.09,0.10) | 0.16 (0.03,0.28) | 3.47e-01 | 6e-01 |
| Belief in God | 0.20 (0.19,0.21) | 0.20 (0.19,0.21) | 0.43 (0.23,0.62) | 2.96e-02 | 5.1e-02 |
| Intrinsic Religiosity | 0.15 (0.14,0.16) | 0.19 (0.17,0.20) | 0.22 (0.00,0.45) | 9.81e-06 | 2.11e-10 |
| Religious Comfort | 0.13 (0.12,0.14) | 0.17 (0.16,0.18) | 0.23 (0.02,0.44) | 4.55e-05 | 7.18e-12 |
| Loved by God | 0.17 (0.16,0.18) | 0.25 (0.23,0.27) | 0.33 (0.05,0.60) | 1.46e-12 | 1.6e-16 |
| Spiritual Punishment | 0.06 (0.05,0.06) | 0.05 (0.05,0.06) | 0.15 (0.00,0.31) | 3.74e-01 | 1.94e-01 |
| Religious Criticism | 0.04 (0.03,0.04) | 0.03 (0.03,0.04) | 0.11 (0.00,0.33) | 6.09e-01 | 2.78e-01 |
| Evangelism | 0.09 (0.08,0.09) | 0.08 (0.08,0.09) | 0.12 (0.00,0.26) | 3.97e-01 | 5.68e-01 |
| *Family Factors* | | | | | |
| Ever Married | 0.71 (0.70,0.72) | 0.79 (0.78,0.79) | 0.39 (0.18,0.60) | 1.6e-16 | 1.6e-16 |
| Divorced | 0.08 (0.07,0.09) | 0.13 (0.12,0.13) | 0.10 (0.00,0.22) | 1.32e-08 | 6.66e-16 |
| Children | 0.44 (0.41,0.47) | 0.36 (0.34,0.38) | 0.61 (0.17,1.05) | 1.09e-03 | 7.3e-05 |

**Table S11a. Nationally representative descriptive statistics for Kenya**

| Characteristic | N = 11,389[1] |
|---|---|
| **Age group** | |
| 18-24 | 2,868 (25%) |
| 25-29 | 2,035 (18%) |
| 30-39 | 2,564 (23%) |
| 40-49 | 1,708 (15%) |
| 50-59 | 1,072 (9.4%) |
| 60-69 | 710 (6.2%) |
| 70-79 | 360 (3.2%) |
| 80 or older | 67 (0.6%) |
| (Missing) | 5 (<0.1%) |
| **Gender** | |
| Male | 5,567 (49%) |
| Female | 5,813 (51%) |
| Other | 2 (<0.1%) |
| (Missing) | 7 (<0.1%) |
| **Marital status** | |
| Married | 6,626 (58%) |
| Separated | 467 (4.1%) |
| Divorced | 111 (1.0%) |
| Widowed | 464 (4.1%) |
| Single, never married | 3,531 (31%) |
| Domestic Partner | 146 (1.3%) |
| (Missing) | 43 (0.4%) |
| **Employment** | |
| Employed for an employer | 1,467 (13%) |
| Self-employed | 3,630 (32%) |
| Retired | 319 (2.8%) |
| Student | 1,136 (10.0%) |
| Homemaker | 1,537 (13%) |
| Unemployed and looking for a job | 3,153 (28%) |
| None of these/Other | 138 (1.2%) |
| (Missing) | 9 (<0.1%) |
| **Religious service attendance** | |
| More than 1/week | 2,774 (24%) |
| 1/week | 6,063 (53%) |
| 1-3/month | 1,219 (11%) |
| A few times a year | 855 (7.5%) |
| Never | 465 (4.1%) |
| (Missing) | 13 (0.1%) |
| **Education** | |
| Up to 8 years | 4,485 (39%) |
| 9-15 years | 6,115 (54%) |
| 16+ years | 783 (6.9%) |
| (Missing) | 6 (<0.1%) |
| **Immigration** | |
| Born in this country | 11,270 (99%) |
| Born in another country | 117 (1.0%) |
| (Missing) | 2 (<0.1%) |
| **Religious affiliation** | |
| Christianity | 10,334 (91%) |
| Islam | 918 (8.1%) |
| Hinduism | 0 (0%) |
| Buddhism | 1 (<0.1%) |
| Judaism | 3 (<0.1%) |
| Sikhism | 0 (0%) |
| Baha'i | 1 (<0.1%) |
| Jainism | 1 (<0.1%) |
| Shinto | 0 (0%) |
| Taoism | 0 (0%) |
| Confucianism | 3 (<0.1%) |
| Primal, Animist, or Folk religion | 7 (<0.1%) |
| Spiritism | 0 (0%) |
| Umbanda, Candomble, and other African-derived religions | 0 (0%) |
| Chinese folk/traditional religion | 0 (0%) |
| Some other religion | 5 (<0.1%) |
| No religion/Atheist/Agnostic | 108 (0.9%) |
| (Missing) | 9 (<0.1%) |
| **Race/Ethnicity** | |

| **Characteristic** | **N = 11,389**[1] |
|---|---|
| (Missing) | 27 (0.2%) |
| Embu | 197 (1.7%) |
| Kalenjin | 1,377 (12%) |
| Kamba | 1,299 (11%) |
| Kenyan Somali/Somali | 396 (3.5%) |
| Kikuyu | 2,119 (19%) |
| Kisii | 789 (6.9%) |
| Luhya | 1,943 (17%) |
| Luo | 1,120 (9.8%) |
| Maasai | 237 (2.1%) |
| Meru | 630 (5.5%) |
| Miji Kenda tribes | 708 (6.2%) |
| Other | 548 (4.8%) |

[1] n (%)

**Table S11b. Descriptive statistics of outcome variables for Kenya**

| Characteristic | N = 11,389[1] |
|---|---|
| **Flourishing Index** | 7.77 (1.62) |
| (Missing) | 148 |
| **Secure Flourishing Index** | 7.29 (1.61) |
| (Missing) | 167 |
| **Happiness & Life Satisfaction** | 6.62 (2.67) |
| (Missing) | 22 |
| **Social Relationship Quality** | 7.88 (2.41) |
| (Missing) | 46 |
| **Meaning and Purpose** | 7.86 (2.20) |
| (Missing) | 47 |
| **Character & Virtue** | 8.10 (2.24) |
| (Missing) | 20 |
| **Self-Rated Health** | 8.40 (2.06) |
| (Missing) | 26 |
| **Financial and Material Worry** | 4.8 (3.4) |
| (Missing) | 21 |
| **Happiness** | 7.3 (3.0) |
| (Missing) | 12 |
| **Life Satisfaction** | 6.0 (3.5) |
| (Missing) | 14 |
| **Present Life Evaluation** | 5.5 (3.3) |
| (Missing) | 18 |
| **Future Life Evaluation** | 7.47 (2.86) |
| (Missing) | 124 |
| **Optimism** | 8.92 (2.20) |
| (Missing) | 9 |
| **Freedom** | 8.42 (2.57) |
| (Missing) | 11 |
| **Peace** | |
| Always | 2,953 (26%) |
| Often | 3,957 (35%) |
| Rarely | 3,827 (34%) |
| Never | 629 (5.5%) |
| (Missing) | 23 (0.2%) |
| **Balance in Life** | |
| Always | 2,431 (21%) |
| Often | 3,674 (32%) |
| Rarely | 4,599 (40%) |
| Never | 672 (5.9%) |
| (Missing) | 14 (0.1%) |
| **Mastery** | |
| Always | 4,065 (36%) |
| Often | 3,814 (33%) |
| Rarely | 3,006 (26%) |
| Never | 479 (4.2%) |
| (Missing) | 26 (0.2%) |
| **Meaning** | 7.2 (3.1) |
| (Missing) | 18 |
| **Purpose** | 8.51 (2.51) |
| (Missing) | 30 |
| **Self-Rated Mental Health** | 8.73 (2.27) |
| (Missing) | 16 |
| **Content with My Relationships** | 8.09 (2.70) |
| (Missing) | 27 |
| **Satisfying Relationships** | 7.7 (2.9) |
| (Missing) | 19 |
| **Social Support** | 7.0 (3.4) |
| (Missing) | 15 |
| **Intimate Friend** | |
| Yes | 9,307 (82%) |
| No | 2,074 (18%) |
| (Missing) | 7 (<0.1%) |
| **Government Approval** | |
| Strongly approve | 2,530 (22%) |
| Somewhat approve | 3,367 (30%) |
| Neither approve nor disapprove | 1,648 (14%) |
| Somewhat disapprove | 1,329 (12%) |
| Strongly disapprove | 2,490 (22%) |

| Characteristic | N = 11,389[1] |
|---|---|
| (Missing) | 25 (0.2%) |
| **Political Voice** | |
| Agree | 4,317 (38%) |
| Disagree | 3,510 (31%) |
| Unsure | 3,546 (31%) |
| (Missing) | 16 (0.1%) |
| **Belonging** | 7.8 (3.1) |
| (Missing) | 30 |
| **City Satisfaction** | |
| Satisfied | 9,077 (80%) |
| Dissatisfied | 1,275 (11%) |
| Unsure | 1,031 (9.0%) |
| (Missing) | 7 (<0.1%) |
| **Trust** | |
| All | 300 (2.6%) |
| Most | 1,095 (9.6%) |
| Some | 4,526 (40%) |
| Not very many | 4,568 (40%) |
| None | 854 (7.5%) |
| (Missing) | 46 (0.4%) |
| **Community Participation** | |
| More than once a week | 901 (7.9%) |
| Once a week | 2,219 (19%) |
| One to three times a month | 1,906 (17%) |
| A few times a year | 2,514 (22%) |
| Never | 3,832 (34%) |
| (Missing) | 18 (0.2%) |
| **Traumatic Distress** | |
| A lot | 1,722 (15%) |
| Some | 3,620 (32%) |
| Not very much | 3,180 (28%) |
| None at all | 2,853 (25%) |
| (Missing) | 15 (0.1%) |
| **Suffering** | |
| A lot | 1,235 (11%) |
| Some | 4,010 (35%) |
| Not very much | 3,456 (30%) |
| None at all | 2,671 (23%) |
| (Missing) | 17 (0.2%) |
| **Loneliness** | 3.5 (3.5) |
| (Missing) | 21 |
| **Discrimination** | |
| Always | 1,156 (10%) |
| Often | 2,202 (19%) |
| Rarely | 4,689 (41%) |
| Never | 3,310 (29%) |
| (Missing) | 32 (0.3%) |
| **Promoting Good** | 8.16 (2.68) |
| (Missing) | 11 |
| **Delayed Gratification** | 8.04 (2.79) |
| (Missing) | 12 |
| **Hope** | 8.76 (2.33) |
| (Missing) | 24 |
| **Gratitude** | 8.07 (2.73) |
| (Missing) | 21 |
| **Love** | 8.57 (2.42) |
| (Missing) | 13 |
| **Forgiveness** | |
| Always | 5,319 (47%) |
| Often | 4,335 (38%) |
| Rarely | 1,456 (13%) |
| Never | 271 (2.4%) |
| (Missing) | 8 (<0.1%) |
| **Charitable Giving** | |
| Yes | 3,165 (28%) |
| No | 8,215 (72%) |
| (Missing) | 9 (<0.1%) |
| **Helping** | |
| Yes | 7,486 (66%) |

| **Characteristic** | **N = 11,389**[1] |
|---|---|
| No | 3,882 (34%) |
| (Missing) | 22 (0.2%) |
| **Volunteering** | |
| Yes | 4,501 (40%) |
| No | 6,868 (60%) |
| (Missing) | 20 (0.2%) |
| **Self-Rated Physical Health** | 8.06 (2.66) |
| (Missing) | 13 |
| **Health Limitations** | |
| Yes | 1,676 (15%) |
| No | 9,674 (85%) |
| (Missing) | 39 (0.3%) |
| **Pain** | |
| A lot | 1,173 (10%) |
| Some | 3,932 (35%) |
| Not very much | 2,957 (26%) |
| None at all | 3,321 (29%) |
| (Missing) | 6 (<0.1%) |
| **Smoking** | 0.2803 (1.9232) |
| (Missing) | 111 |
| **Drinking** | 0.42 (2.81) |
| (Missing) | 115 |
| **Exercise** | |
| 0 days | 3,913 (34%) |
| 1 day | 1,330 (12%) |
| 2 days | 1,285 (11%) |
| 3 days | 1,272 (11%) |
| 4 days | 525 (4.6%) |
| 5 days | 366 (3.2%) |
| 6 days | 336 (2.9%) |
| 7 days/Every day | 2,333 (20%) |
| (Missing) | 29 (0.3%) |
| **Financial Stability** | 4.8 (3.8) |
| (Missing) | 10 |
| **Material Stability** | 4.9 (3.8) |
| (Missing) | 11 |
| **Education** | |
| Up to 8 years | 4,485 (39%) |
| 9-15 years | 6,115 (54%) |
| 16+ years | 783 (6.9%) |
| (Missing) | 6 (<0.1%) |
| **Employment** | |
| Employed for an employer | 1,467 (13%) |
| Self-employed | 3,630 (32%) |
| Retired | 319 (2.8%) |
| Student | 1,136 (10.0%) |
| Homemaker | 1,537 (13%) |
| Unemployed and looking for a job | 3,153 (28%) |
| None of these/Other | 138 (1.2%) |
| (Missing) | 9 (<0.1%) |
| **Subjective Financial Well-Being** | |
| Living comfortably on present income | 677 (5.9%) |
| Getting by on present income | 2,305 (20%) |
| Finding it difficult on present income | 5,228 (46%) |
| Finding it very difficult on present income | 3,152 (28%) |
| (Missing) | 27 (0.2%) |
| **Housing** | |
| Someone in this household OWNS this home | 5,719 (50%) |
| Someone in this household RENTS this home | 4,021 (35%) |
| Both | 500 (4.4%) |
| Neither | 1,127 (9.9%) |
| Rent | 0 (0%) |
| Own | 0 (0%) |
| Something else | 0 (0%) |
| (Missing) | 23 (0.2%) |
| **Self-Reported Religion/Spirituality** | |
| Always | 6,103 (54%) |
| Often | 3,078 (27%) |
| Rarely | 1,759 (15%) |

| Characteristic | N = 11,389[1] |
|---|---|
| Never | 442 (3.9%) |
| (Missing) | 7 (<0.1%) |
| **Religious Service Attendance** | |
| More than once a week | 2,774 (24%) |
| Once a week | 6,063 (53%) |
| One to three times a month | 1,219 (11%) |
| A few times a year | 855 (7.5%) |
| Never | 465 (4.1%) |
| (Missing) | 13 (0.1%) |
| **Life after Death Belief** | |
| Yes | 8,184 (72%) |
| No | 1,487 (13%) |
| Unsure | 1,694 (15%) |
| (Missing) | 24 (0.2%) |
| **Religious Experience** | |
| Yes | 7,379 (65%) |
| No | 3,913 (34%) |
| (Missing) | 97 (0.8%) |
| **Religious Reading** | |
| More than once a day | 3,286 (29%) |
| About once a day | 4,055 (36%) |
| Sometimes | 3,534 (31%) |
| Never | 494 (4.3%) |
| (Missing) | 20 (0.2%) |
| **Prayer-Meditation** | |
| More than once a day | 6,124 (54%) |
| About once a day | 3,465 (30%) |
| Sometimes | 1,557 (14%) |
| Never | 226 (2.0%) |
| (Missing) | 17 (0.1%) |
| **Belief in God** | |
| One God | 10,905 (96%) |
| More than one god | 153 (1.3%) |
| An impersonal spiritual force | 145 (1.3%) |
| None of these | 57 (0.5%) |
| Unsure | 117 (1.0%) |
| (Missing) | 12 (0.1%) |
| **Intrinsic Religiosity** | |
| Agree | 9,250 (81%) |
| Disagree | 771 (6.8%) |
| Not relevant | 374 (3.3%) |
| Unsure | 986 (8.7%) |
| (Missing) | 8 (<0.1%) |
| **Religious Comfort** | |
| Agree | 10,003 (88%) |
| Disagree | 544 (4.8%) |
| Not relevant | 336 (2.9%) |
| Unsure | 499 (4.4%) |
| (Missing) | 6 (<0.1%) |
| **Loved by God** | |
| Agree | 10,539 (93%) |
| Disagree | 340 (3.0%) |
| Not relevant | 246 (2.2%) |
| Unsure | 256 (2.2%) |
| (Missing) | 7 (<0.1%) |
| **Spiritual Punishment** | |
| Agree | 4,844 (43%) |
| Disagree | 4,355 (38%) |
| Not relevant | 729 (6.4%) |
| Unsure | 1,440 (13%) |
| (Missing) | 21 (0.2%) |
| **Religious Criticism** | |
| Agree | 5,868 (52%) |
| Disagree | 3,109 (27%) |
| Not relevant | 781 (6.9%) |
| Unsure | 1,616 (14%) |
| (Missing) | 15 (0.1%) |
| **Evangelism** | |
| Agree | 9,271 (81%) |

| Characteristic | N = 11,389[1] |
|---|---|
| Disagree | 1,029 (9.0%) |
| Not relevant | 577 (5.1%) |
| Unsure | 505 (4.4%) |
| (Missing) | 7 (<0.1%) |
| **Children** | 2.03 (1.85) |
| (Missing) | 3 |

[1]Mean (SD); n (%)

**Table S11c. Demographic variation across outcomes for Kenya**

| Outcome | Male | Female | Other | Male vs Female p-value | Global p-value |
|---|---|---|---|---|---|
| *Flourishing Index and Domains* | | | | | |
| Flourishing Index | 7.80 (7.73,7.88) | 7.74 (7.67,7.81) | 9.26 * | 3.22e-01 | 7.36e-02 |
| Secure Flourishing Index | 7.34 (7.28,7.41) | 7.23 (7.16,7.30) | 7.91 * | 7.07e-02 | 9.73e-03 |
| Happiness & Life Satisfaction | 6.58 (6.48,6.68) | 6.66 (6.56,6.76) | 7.99 * | 2.3e-01 | 3.81e-01 |
| Social Relationship Quality | 7.95 (7.86,8.04) | 7.82 (7.73,7.90) | 9.10 * | 8.03e-02 | 3.78e-02 |
| Meaning and Purpose | 7.87 (7.78,7.96) | 7.84 (7.76,7.93) | 9.40 * | 7.76e-01 | 7.63e-02 |
| Character & Virtue | 8.15 (8.05,8.24) | 8.06 (7.97,8.15) | 10.00 * | 4.98e-01 | 1.6e-16 |
| Self-Rated Health | 8.47 (8.38,8.56) | 8.32 (8.21,8.43) | 9.80 * | 4.84e-02 | 9.24e-09 |
| Financial and Material Worry | 5.04 (4.91,5.18) | 4.66 (4.52,4.81) | 1.21 * | 4.68e-03 | 7.1e-06 |
| *Psychological Well-Being* | | | | | |
| Happiness | 7.17 (7.07,7.27) | 7.37 (7.26,7.48) | 7.99 * | 3.13e-01 | 1.07e-02 |
| Life Satisfaction | 5.99 (5.87,6.12) | 5.95 (5.83,6.07) | 7.99 * | 3.3e-01 | 5.63e-01 |
| Present Life Evaluation | 5.51 (5.39,5.64) | 5.51 (5.38,5.64) | 9.00 * | 2.9e-01 | 9.95e-03 |
| Future Life Evaluation | 7.45 (7.34,7.56) | 7.47 (7.36,7.59) | 9.40 * | 5.86e-01 | 1.78e-02 |
| Optimism | 8.88 (8.78,8.98) | 8.97 (8.88,9.05) | 10.00 * | 3.26e-01 | 1.6e-16 |
| Freedom | 8.36 (8.26,8.47) | 8.47 (8.38,8.57) | 9.00 * | 3.06e-01 | 1.69e-01 |
| Peace | 0.64 (0.62,0.66) | 0.58 (0.56,0.59) | 0.00 * | 4.67e-07 | 1.6e-16 |
| Balance in Life | 0.57 (0.56,0.59) | 0.50 (0.48,0.52) | 0.00 * | 1.95e-05 | 1.6e-16 |
| Mastery | 0.72 (0.70,0.73) | 0.67 (0.66,0.69) | 0.00 * | 9.35e-03 | 1.6e-16 |
| Meaning | 7.22 (7.10,7.34) | 7.19 (7.07,7.30) | 8.79 * | 9.05e-01 | 4.78e-01 |
| Purpose | 8.52 (8.43,8.61) | 8.50 (8.40,8.60) | 10.00 * | 4.83e-01 | 1.6e-16 |
| Self-Rated Mental Health | 8.78 (8.68,8.88) | 8.68 (8.55,8.81) | 9.60 * | 1.1e-01 | 5e-02 |
| *Social Well-Being* | | | | | |
| Content with My Relationships | 8.17 (8.07,8.27) | 8.01 (7.90,8.11) | 9.00 * | 5.09e-02 | 4.18e-02 |
| Satisfying Relationships | 7.73 (7.63,7.83) | 7.62 (7.53,7.72) | 9.20 * | 2.57e-01 | 6.61e-02 |
| Social Support | 7.04 (6.91,7.17) | 6.88 (6.74,7.02) | 8.59 * | 2.67e-01 | 7.8e-02 |
| Intimate Friend | 0.82 (0.81,0.84) | 0.81 (0.80,0.83) | 0.80 * | 4.9e-01 | 4.66e-01 |
| Government Approval | 0.52 (0.50,0.54) | 0.52 (0.50,0.54) | 0.00 * | 7.31e-01 | 1.6e-16 |
| Political Voice | 0.54 (0.52,0.57) | 0.54 (0.52,0.56) | 1.00 * | 6e-01 | 1.6e-16 |
| Belonging | 7.78 (7.66,7.90) | 7.76 (7.64,7.89) | 9.00 * | 9.27e-01 | 5.47e-01 |
| City Satisfaction | 0.87 (0.86,0.89) | 0.88 (0.86,0.89) | 1.00 * | 6.86e-01 | 1.6e-16 |
| Trust | 0.13 (0.12,0.14) | 0.11 (0.10,0.12) | 0.00 * | 1.82e-01 | 1.6e-16 |
| Community Participation | 0.33 (0.31,0.34) | 0.22 (0.21,0.24) | 0.00 * | 2.53e-11 | 1.6e-16 |
| *Psychological Distress* | | | | | |
| Traumatic Distress | 0.46 (0.44,0.48) | 0.48 (0.46,0.49) | 1.00 * | 4.14e-01 | 1.6e-16 |
| Depression Symptoms | 0.40 (0.38,0.41) | 0.40 (0.38,0.42) | 0.80 * | 2.51e-01 | 2.06e-01 |
| Anxiety Symptoms | 0.35 (0.34,0.37) | 0.36 (0.35,0.38) | 1.00 * | 8.44e-02 | 1.6e-16 |
| Suffering | 0.45 (0.43,0.47) | 0.47 (0.46,0.49) | 0.00 * | 2.91e-02 | 1.6e-16 |
| *Social Distress* | | | | | |
| Loneliness | 3.43 (3.30,3.57) | 3.64 (3.51,3.77) | 1.00 * | 4.24e-02 | 1.09e-02 |
| Discrimination | 0.31 (0.29,0.33) | 0.28 (0.26,0.30) | 0.80 * | 3.03e-01 | 4.35e-03 |
| *Character & Prosocial Behavior* | | | | | |
| Promoting Good | 8.19 (8.08,8.29) | 8.14 (8.04,8.24) | 10.00 * | 6.19e-01 | 1.6e-16 |
| Delayed Gratification | 8.11 (8.00,8.22) | 7.98 (7.86,8.10) | 10.00 * | 5.85e-01 | 1.6e-16 |
| Hope | 8.77 (8.68,8.86) | 8.75 (8.66,8.84) | 10.00 * | 7.42e-01 | 1.6e-16 |
| Gratitude | 8.04 (7.94,8.14) | 8.11 (8.00,8.21) | 10.00 * | 5.7e-01 | 1.6e-16 |
| Love | 8.55 (8.45,8.64) | 8.59 (8.50,8.68) | 9.60 * | 9.24e-01 | 6.15e-02 |
| Forgiveness | 0.86 (0.84,0.87) | 0.84 (0.83,0.86) | 1.00 * | 9.17e-02 | 1.6e-16 |
| Charitable Giving | 0.30 (0.27,0.32) | 0.26 (0.24,0.28) | 0.00 * | 6.14e-04 | 1.6e-16 |
| Helping | 0.69 (0.67,0.71) | 0.63 (0.61,0.65) | 0.80 * | 9.23e-04 | 5.44e-08 |
| Volunteering | 0.41 (0.39,0.44) | 0.38 (0.36,0.40) | 0.20 * | 1.07e-02 | 2.04e-02 |
| *Physical Health & Health Behavior* | | | | | |
| Self-Rated Physical Health | 8.16 (8.06,8.26) | 7.97 (7.85,8.09) | 10.00 * | 7.57e-02 | 1.6e-16 |
| Health Limitations | 0.12 (0.11,0.14) | 0.17 (0.16,0.18) | 0.00 * | 7.38e-03 | 1.6e-16 |
| Pain | 0.40 (0.38,0.42) | 0.50 (0.48,0.51) | 1.00 * | 9.08e-10 | 1.6e-16 |
| Smoking | 0.50 (0.43,0.57) | 0.05 (0.02,0.08) | 0.00 * | 2.49e-13 | 1.6e-16 |
| Drinking | 0.67 (0.57,0.77) | 0.13 (0.09,0.17) | 0.00 * | 1.61e-14 | 1.6e-16 |
| Exercise | 3.22 (3.12,3.33) | 2.08 (1.95,2.21) | 1.60 * | 1.6e-16 | 1.6e-16 |
| *Socioeconomic Outcomes* | | | | | |
| Financial Stability | 4.96 (4.83,5.10) | 4.57 (4.42,4.72) | 1.21 * | 2.88e-02 | 1.07e-05 |
| Material Stability | 5.13 (4.98,5.28) | 4.76 (4.60,4.92) | 1.21 * | 4.38e-03 | 9.01e-05 |
| Education | 0.09 (0.08,0.10) | 0.05 (0.04,0.06) | 0.00 * | 3.32e-09 | 1.6e-16 |
| Employment | 0.55 (0.53,0.58) | 0.35 (0.33,0.36) | 0.00 * | 1.6e-16 | 1.6e-16 |
| Subjective Financial Well-Being | 0.29 (0.28,0.31) | 0.23 (0.22,0.25) | 0.20 * | 2.05e-05 | 8.31e-08 |
| Housing | 0.55 (0.53,0.58) | 0.54 (0.51,0.57) | 0.20 * | 3.49e-01 | 2.42e-01 |
| *Religion/Spirituality* | | | | | |
| Self-Reported Religion/Spirituality | 0.79 (0.77,0.80) | 0.82 (0.81,0.84) | 1.00 * | 7.78e-03 | 1.6e-16 |
| Religious Service Attendance | 0.72 (0.70,0.74) | 0.83 (0.81,0.84) | 1.00 * | 2.24e-14 | 1.6e-16 |

| Outcome | Male | Female | Other | Male vs Female p-value | Global p-value |
|---|---|---|---|---|---|
| Life after Death Belief | 0.71 (0.70,0.73) | 0.73 (0.71,0.74) | 1.00 * | 4.93e-01 | 1.6e-16 |
| Religious Experience | 0.65 (0.63,0.67) | 0.66 (0.64,0.67) | 0.20 * | 4.08e-01 | 1.29e-01 |
| Religious Reading | 0.60 (0.58,0.62) | 0.69 (0.67,0.71) | 1.00 * | 1.07e-09 | 1.6e-16 |
| Prayer-Meditation | 0.81 (0.80,0.83) | 0.87 (0.86,0.89) | 1.00 * | 4.12e-07 | 1.6e-16 |
| Belief in God | 0.98 (0.98,0.99) | 0.99 (0.98,0.99) | 1.00 * | 1.3e-02 | 1.6e-16 |
| Intrinsic Religiosity | 0.90 (0.89,0.92) | 0.92 (0.91,0.93) | 1.00 * | 7.29e-02 | 1.6e-16 |
| Religious Comfort | 0.93 (0.92,0.94) | 0.94 (0.93,0.96) | 1.00 * | 1.43e-01 | 1.6e-16 |
| Loved by God | 0.96 (0.95,0.97) | 0.96 (0.95,0.97) | 1.00 * | 7.49e-01 | 1.6e-16 |
| Spiritual Punishment | 0.51 (0.49,0.53) | 0.53 (0.50,0.55) | 0.80 * | 6.34e-02 | 1.87e-01 |
| Religious Criticism | 0.63 (0.61,0.66) | 0.65 (0.63,0.67) | 0.80 * | 1.61e-02 | 3.59e-01 |
| Evangelism | 0.88 (0.86,0.89) | 0.90 (0.89,0.91) | 1.00 * | 2.78e-02 | 1.6e-16 |
| *Family Factors* | | | | | |
| Ever Married | 0.64 (0.61,0.66) | 0.71 (0.70,0.73) | 0.80 * | 2.57e-07 | 1.53e-10 |
| Divorced | 0.01 (0.01,0.01) | 0.01 (0.01,0.01) | 0.00 * | 5.24e-01 | 6.27e-12 |
| Children | 1.85 (1.76,1.94) | 2.21 (2.13,2.29) | 0.00 * | 1.03e-08 | 1.6e-16 |

**Table S12a. Nationally representative descriptive statistics for Mexico**

| Characteristic | N = 5,776[1] |
|---|---|
| **Age group** | |
| 18-24 | 986 (17%) |
| 25-29 | 623 (11%) |
| 30-39 | 1,312 (23%) |
| 40-49 | 1,027 (18%) |
| 50-59 | 873 (15%) |
| 60-69 | 611 (11%) |
| 70-79 | 277 (4.8%) |
| 80 or older | 68 (1.2%) |
| (Missing) | 0 (0%) |
| **Gender** | |
| Male | 2,755 (48%) |
| Female | 2,997 (52%) |
| Other | 3 (<0.1%) |
| (Missing) | 21 (0.4%) |
| **Marital status** | |
| Married | 2,089 (36%) |
| Separated | 403 (7.0%) |
| Divorced | 230 (4.0%) |
| Widowed | 347 (6.0%) |
| Single, never married | 1,432 (25%) |
| Domestic Partner | 1,109 (19%) |
| (Missing) | 166 (2.9%) |
| **Employment** | |
| Employed for an employer | 1,921 (33%) |
| Self-employed | 1,091 (19%) |
| Retired | 386 (6.7%) |
| Student | 247 (4.3%) |
| Homemaker | 1,257 (22%) |
| Unemployed and looking for a job | 564 (9.8%) |
| None of these/Other | 169 (2.9%) |
| (Missing) | 141 (2.4%) |
| **Religious service attendance** | |
| More than 1/week | 609 (11%) |
| 1/week | 1,261 (22%) |
| 1-3/month | 676 (12%) |
| A few times a year | 2,054 (36%) |
| Never | 1,134 (20%) |
| (Missing) | 43 (0.7%) |
| **Education** | |
| Up to 8 years | 1,291 (22%) |
| 9-15 years | 3,180 (55%) |
| 16+ years | 1,304 (23%) |
| (Missing) | 1 (<0.1%) |
| **Immigration** | |
| Born in this country | 5,517 (96%) |
| Born in another country | 108 (1.9%) |
| (Missing) | 151 (2.6%) |
| **Religious affiliation** | |
| Christianity | 4,844 (84%) |
| Islam | 2 (<0.1%) |
| Hinduism | 3 (<0.1%) |
| Buddhism | 6 (0.1%) |
| Judaism | 7 (0.1%) |
| Sikhism | 0 (0%) |
| Baha'i | 1 (<0.1%) |
| Jainism | 1 (<0.1%) |
| Shinto | 2 (<0.1%) |
| Taoism | 4 (<0.1%) |
| Confucianism | 1 (<0.1%) |
| Primal, Animist, or Folk religion | 20 (0.3%) |
| Spiritism | 0 (0%) |
| Umbanda, Candomble, and other African-derived religions | 0 (0%) |
| Chinese folk/traditional religion | 0 (0%) |
| Some other religion | 41 (0.7%) |
| No religion/Atheist/Agnostic | 770 (13%) |
| (Missing) | 75 (1.3%) |
| **Race/Ethnicity** | |

| Characteristic | N = 5,776[1] |
|---|---|
| (Missing) | 671 (12%) |
| Black | 108 (1.9%) |
| Indigenous | 594 (10%) |
| Mestizo | 2,762 (48%) |
| Mulatto | 63 (1.1%) |
| Other | 339 (5.9%) |
| White | 1,116 (19%) |
| (Missing) | 123 (2.1%) |

[1]n (%)

**Table S12b. Descriptive statistics of outcome variables for Mexico**

| Characteristic | N = 5,776[1] |
|---|---|
| **Flourishing Index** | 8.19 (1.35) |
| (Missing) | 171 |
| **Secure Flourishing Index** | 7.64 (1.37) |
| (Missing) | 192 |
| **Happiness & Life Satisfaction** | 7.82 (1.93) |
| (Missing) | 31 |
| **Social Relationship Quality** | 8.20 (1.89) |
| (Missing) | 36 |
| **Meaning and Purpose** | 8.44 (1.67) |
| (Missing) | 32 |
| **Character & Virtue** | 8.44 (1.52) |
| (Missing) | 77 |
| **Self-Rated Health** | 8.07 (1.66) |
| (Missing) | 28 |
| **Financial and Material Worry** | 4.9 (3.5) |
| (Missing) | 27 |
| **Happiness** | 7.79 (2.03) |
| (Missing) | 19 |
| **Life Satisfaction** | 7.85 (2.14) |
| (Missing) | 15 |
| **Present Life Evaluation** | 7.10 (2.11) |
| (Missing) | 12 |
| **Future Life Evaluation** | 8.31 (2.05) |
| (Missing) | 171 |
| **Optimism** | 9.06 (1.66) |
| (Missing) | 19 |
| **Freedom** | 8.55 (1.96) |
| (Missing) | 15 |
| **Peace** | |
| Always | 1,610 (28%) |
| Often | 2,262 (39%) |
| Rarely | 1,434 (25%) |
| Never | 425 (7.4%) |
| (Missing) | 44 (0.8%) |
| **Balance in Life** | |
| Always | 1,459 (25%) |
| Often | 2,927 (51%) |
| Rarely | 1,116 (19%) |
| Never | 237 (4.1%) |
| (Missing) | 37 (0.6%) |
| **Mastery** | |
| Always | 2,524 (44%) |
| Often | 2,611 (45%) |
| Rarely | 494 (8.5%) |
| Never | 85 (1.5%) |
| (Missing) | 63 (1.1%) |
| **Meaning** | 8.43 (1.87) |
| (Missing) | 11 |
| **Purpose** | 8.44 (1.96) |
| (Missing) | 22 |
| **Self-Rated Mental Health** | 8.35 (1.84) |
| (Missing) | 15 |
| **Content with My Relationships** | 8.39 (2.00) |
| (Missing) | 21 |
| **Satisfying Relationships** | 8.00 (2.14) |
| (Missing) | 18 |
| **Social Support** | 8.27 (2.36) |
| (Missing) | 30 |
| **Intimate Friend** | |
| Yes | 4,539 (79%) |
| No | 1,188 (21%) |
| (Missing) | 49 (0.8%) |
| **Government Approval** | |
| Strongly approve | 1,295 (22%) |
| Somewhat approve | 1,888 (33%) |
| Neither approve nor disapprove | 1,360 (24%) |
| Somewhat disapprove | 659 (11%) |
| Strongly disapprove | 509 (8.8%) |

| Characteristic | N = 5,776[1] |
|---|---|
| (Missing) | 65 (1.1%) |
| **Political Voice** | |
| Agree | 2,485 (43%) |
| Disagree | 1,445 (25%) |
| Unsure | 1,760 (30%) |
| (Missing) | 86 (1.5%) |
| **Belonging** | 8.49 (2.01) |
| (Missing) | 92 |
| **City Satisfaction** | |
| Satisfied | 4,282 (74%) |
| Dissatisfied | 832 (14%) |
| Unsure | 612 (11%) |
| (Missing) | 51 (0.9%) |
| **Trust** | |
| All | 153 (2.7%) |
| Most | 978 (17%) |
| Some | 2,597 (45%) |
| Not very many | 1,701 (29%) |
| None | 300 (5.2%) |
| (Missing) | 47 (0.8%) |
| **Community Participation** | |
| More than once a week | 385 (6.7%) |
| Once a week | 525 (9.1%) |
| One to three times a month | 516 (8.9%) |
| A few times a year | 1,756 (30%) |
| Never | 2,565 (44%) |
| (Missing) | 28 (0.5%) |
| **Traumatic Distress** | |
| A lot | 766 (13%) |
| Some | 1,398 (24%) |
| Not very much | 1,713 (30%) |
| None at all | 1,874 (32%) |
| (Missing) | 26 (0.4%) |
| **Suffering** | |
| A lot | 485 (8.4%) |
| Some | 1,753 (30%) |
| Not very much | 1,842 (32%) |
| None at all | 1,671 (29%) |
| (Missing) | 25 (0.4%) |
| **Loneliness** | 3.3 (3.2) |
| (Missing) | 16 |
| **Discrimination** | |
| Always | 429 (7.4%) |
| Often | 884 (15%) |
| Rarely | 2,091 (36%) |
| Never | 2,331 (40%) |
| (Missing) | 41 (0.7%) |
| **Promoting Good** | 8.62 (1.59) |
| (Missing) | 33 |
| **Delayed Gratification** | 8.27 (2.04) |
| (Missing) | 51 |
| **Hope** | 9.00 (1.56) |
| (Missing) | 30 |
| **Gratitude** | 8.84 (1.75) |
| (Missing) | 21 |
| **Love** | 8.71 (1.85) |
| (Missing) | 21 |
| **Forgiveness** | |
| Always | 2,243 (39%) |
| Often | 2,197 (38%) |
| Rarely | 1,008 (17%) |
| Never | 297 (5.1%) |
| (Missing) | 30 (0.5%) |
| **Charitable Giving** | |
| Yes | 1,182 (20%) |
| No | 4,584 (79%) |
| (Missing) | 10 (0.2%) |
| **Helping** | |
| Yes | 3,620 (63%) |

| Characteristic | N = 5,776[1] |
|---|---|
| No | 2,127 (37%) |
| (Missing) | 29 (0.5%) |
| **Volunteering** | |
| Yes | 1,232 (21%) |
| No | 4,524 (78%) |
| (Missing) | 20 (0.4%) |
| **Self-Rated Physical Health** | 7.79 (1.96) |
| (Missing) | 16 |
| **Health Limitations** | |
| Yes | 983 (17%) |
| No | 4,586 (79%) |
| (Missing) | 206 (3.6%) |
| **Pain** | |
| A lot | 568 (9.8%) |
| Some | 1,603 (28%) |
| Not very much | 1,995 (35%) |
| None at all | 1,603 (28%) |
| (Missing) | 6 (0.1%) |
| **Smoking** | 1.23 (4.13) |
| (Missing) | 122 |
| **Drinking** | 1.38 (3.55) |
| (Missing) | 103 |
| **Exercise** | |
| 0 days | 2,062 (36%) |
| 1 day | 663 (11%) |
| 2 days | 593 (10%) |
| 3 days | 733 (13%) |
| 4 days | 279 (4.8%) |
| 5 days | 393 (6.8%) |
| 6 days | 172 (3.0%) |
| 7 days/Every day | 847 (15%) |
| (Missing) | 35 (0.6%) |
| **Financial Stability** | 4.9 (3.7) |
| (Missing) | 7 |
| **Material Stability** | 4.8 (3.8) |
| (Missing) | 22 |
| **Education** | |
| Up to 8 years | 1,291 (22%) |
| 9-15 years | 3,180 (55%) |
| 16+ years | 1,304 (23%) |
| (Missing) | 1 (<0.1%) |
| **Employment** | |
| Employed for an employer | 1,921 (33%) |
| Self-employed | 1,091 (19%) |
| Retired | 386 (6.7%) |
| Student | 247 (4.3%) |
| Homemaker | 1,257 (22%) |
| Unemployed and looking for a job | 564 (9.8%) |
| None of these/Other | 169 (2.9%) |
| (Missing) | 141 (2.4%) |
| **Subjective Financial Well-Being** | |
| Living comfortably on present income | 771 (13%) |
| Getting by on present income | 1,953 (34%) |
| Finding it difficult on present income | 2,054 (36%) |
| Finding it very difficult on present income | 683 (12%) |
| (Missing) | 315 (5.5%) |
| **Housing** | |
| Someone in this household OWNS this home | 2,994 (52%) |
| Someone in this household RENTS this home | 927 (16%) |
| Both | 368 (6.4%) |
| Neither | 1,195 (21%) |
| Rent | 0 (0%) |
| Own | 0 (0%) |
| Something else | 0 (0%) |
| (Missing) | 292 (5.1%) |
| **Self-Reported Religion/Spirituality** | |
| Always | 2,018 (35%) |
| Often | 1,845 (32%) |
| Rarely | 1,394 (24%) |

| Characteristic | N = 5,776[1] |
|---|---|
| Never | 490 (8.5%) |
| (Missing) | 29 (0.5%) |
| **Religious Service Attendance** | |
| More than once a week | 609 (11%) |
| Once a week | 1,261 (22%) |
| One to three times a month | 676 (12%) |
| A few times a year | 2,054 (36%) |
| Never | 1,134 (20%) |
| (Missing) | 43 (0.7%) |
| **Life after Death Belief** | |
| Yes | 3,437 (60%) |
| No | 851 (15%) |
| Unsure | 1,411 (24%) |
| (Missing) | 77 (1.3%) |
| **Religious Experience** | |
| Yes | 2,571 (45%) |
| No | 3,153 (55%) |
| (Missing) | 52 (0.9%) |
| **Religious Reading** | |
| More than once a day | 497 (8.6%) |
| About once a day | 816 (14%) |
| Sometimes | 3,125 (54%) |
| Never | 1,276 (22%) |
| (Missing) | 62 (1.1%) |
| **Prayer-Meditation** | |
| More than once a day | 1,010 (17%) |
| About once a day | 1,724 (30%) |
| Sometimes | 2,356 (41%) |
| Never | 639 (11%) |
| (Missing) | 47 (0.8%) |
| **Belief in God** | |
| One God | 4,539 (79%) |
| More than one god | 229 (4.0%) |
| An impersonal spiritual force | 467 (8.1%) |
| None of these | 209 (3.6%) |
| Unsure | 290 (5.0%) |
| (Missing) | 44 (0.8%) |
| **Intrinsic Religiosity** | |
| Agree | 3,102 (54%) |
| Disagree | 633 (11%) |
| Not relevant | 991 (17%) |
| Unsure | 958 (17%) |
| (Missing) | 93 (1.6%) |
| **Religious Comfort** | |
| Agree | 3,847 (67%) |
| Disagree | 394 (6.8%) |
| Not relevant | 804 (14%) |
| Unsure | 698 (12%) |
| (Missing) | 33 (0.6%) |
| **Loved by God** | |
| Agree | 4,486 (78%) |
| Disagree | 271 (4.7%) |
| Not relevant | 564 (9.8%) |
| Unsure | 415 (7.2%) |
| (Missing) | 40 (0.7%) |
| **Spiritual Punishment** | |
| Agree | 850 (15%) |
| Disagree | 3,054 (53%) |
| Not relevant | 961 (17%) |
| Unsure | 850 (15%) |
| (Missing) | 60 (1.0%) |
| **Religious Criticism** | |
| Agree | 1,319 (23%) |
| Disagree | 1,650 (29%) |
| Not relevant | 1,308 (23%) |
| Unsure | 1,428 (25%) |
| (Missing) | 71 (1.2%) |
| **Evangelism** | |
| Agree | 2,662 (46%) |

| Characteristic | N = 5,776[1] |
|---|---|
| Disagree | 1,216 (21%) |
| Not relevant | 1,212 (21%) |
| Unsure | 629 (11%) |
| (Missing) | 57 (1.0%) |
| **Children** | 1.16 (1.42) |
| (Missing) | 150 |

[1]Mean (SD); n (%)

**Table S12c. Demographic variation across outcomes for Mexico**

| Outcome | Male | Female | Other | Male vs Female p-value | Global p-value |
|---|---|---|---|---|---|
| *Flourishing Index and Domains* | | | | | |
| Flourishing Index | 8.24 (8.17,8.30) | 8.15 (8.09,8.21) | 7.47 * | 2.08e-01 | 9.52e-02 |
| Secure Flourishing Index | 7.74 (7.67,7.81) | 7.54 (7.48,7.60) | 7.57 * | 1.09e-02 | 3.01e-04 |
| Happiness & Life Satisfaction | 7.86 (7.76,7.95) | 7.78 (7.70,7.87) | 6.41 * | 5.67e-01 | 1.59e-01 |
| Social Relationship Quality | 8.17 (8.08,8.27) | 8.22 (8.14,8.30) | 7.67 * | 7.55e-01 | 6.3e-01 |
| Meaning and Purpose | 8.42 (8.33,8.50) | 8.45 (8.38,8.52) | 7.78 * | 6.54e-01 | 5.35e-01 |
| Character & Virtue | 8.50 (8.43,8.57) | 8.39 (8.32,8.46) | 8.45 * | 3.3e-02 | 7.27e-02 |
| Self-Rated Health | 8.24 (8.16,8.32) | 7.91 (7.84,7.99) | 7.02 * | 1.05e-02 | 3.09e-08 |
| Financial and Material Worry | 5.23 (5.06,5.41) | 4.51 (4.36,4.66) | 8.09 * | 2.48e-04 | 2.06e-13 |
| *Psychological Well-Being* | | | | | |
| Happiness | 7.80 (7.70,7.90) | 7.77 (7.68,7.86) | 6.56 * | 7.21e-01 | 2.55e-01 |
| Life Satisfaction | 7.91 (7.80,8.02) | 7.79 (7.70,7.89) | 6.26 * | 1.65e-01 | 8.86e-02 |
| Present Life Evaluation | 7.11 (7.01,7.22) | 7.09 (7.00,7.18) | 7.46 * | 7.37e-01 | 7.98e-01 |
| Future Life Evaluation | 8.23 (8.13,8.34) | 8.31 (8.21,8.41) | 7.40 * | 2.36e-01 | 2.52e-01 |
| Optimism | 8.97 (8.88,9.07) | 9.14 (9.07,9.21) | 8.06 * | 6.92e-02 | 6.02e-03 |
| Freedom | 8.56 (8.47,8.66) | 8.55 (8.46,8.63) | 8.84 * | 4.95e-01 | 8.74e-01 |
| Peace | 0.69 (0.67,0.71) | 0.66 (0.64,0.68) | 0.80 * | 1.61e-01 | 8.73e-02 |
| Balance in Life | 0.80 (0.78,0.82) | 0.73 (0.71,0.75) | 1.00 * | 1.85e-04 | 1.6e-16 |
| Mastery | 0.91 (0.90,0.93) | 0.89 (0.87,0.90) | 0.56 * | 1.05e-02 | 6.31e-03 |
| Meaning | 8.40 (8.30,8.49) | 8.46 (8.38,8.54) | 7.94 * | 5.05e-01 | 4.68e-01 |
| Purpose | 8.43 (8.33,8.53) | 8.44 (8.35,8.52) | 7.61 * | 8.83e-01 | 5.82e-01 |
| Self-Rated Mental Health | 8.48 (8.39,8.57) | 8.23 (8.15,8.31) | 7.79 * | 2.85e-01 | 4.23e-04 |
| *Social Well-Being* | | | | | |
| Content with My Relationships | 8.35 (8.25,8.45) | 8.43 (8.35,8.51) | 7.27 * | 6.62e-01 | 3.09e-01 |
| Satisfying Relationships | 7.99 (7.89,8.10) | 8.00 (7.91,8.10) | 8.07 * | 3.65e-01 | 9.8e-01 |
| Social Support | 8.21 (8.10,8.32) | 8.32 (8.22,8.42) | 8.19 * | 9.99e-02 | 3.51e-01 |
| Intimate Friend | 0.77 (0.75,0.80) | 0.81 (0.79,0.83) | 0.79 * | 6.83e-02 | 5.11e-02 |
| Government Approval | 0.60 (0.57,0.62) | 0.52 (0.50,0.54) | 0.77 * | 2.49e-03 | 4.76e-06 |
| Political Voice | 0.62 (0.60,0.65) | 0.56 (0.54,0.59) | 0.46 * | 7.27e-03 | 8.78e-04 |
| Belonging | 8.49 (8.39,8.59) | 8.48 (8.39,8.57) | 8.19 * | 7.8e-01 | 9.04e-01 |
| City Satisfaction | 0.82 (0.80,0.85) | 0.83 (0.82,0.85) | 0.71 * | 6.85e-01 | 4.73e-01 |
| Trust | 0.23 (0.21,0.25) | 0.17 (0.15,0.19) | 0.23 * | 2.91e-03 | 3.52e-04 |
| Community Participation | 0.20 (0.18,0.22) | 0.12 (0.11,0.14) | 0.33 * | 3.81e-07 | 1.29e-09 |
| *Psychological Distress* | | | | | |
| Traumatic Distress | 0.35 (0.33,0.38) | 0.40 (0.38,0.42) | 0.60 * | 5.56e-02 | 1.51e-02 |
| Depression Symptoms | 0.25 (0.23,0.27) | 0.30 (0.28,0.32) | 0.45 * | 1.18e-02 | 3.71e-03 |
| Anxiety Symptoms | 0.27 (0.24,0.29) | 0.31 (0.29,0.33) | 0.64 * | 1.33e-02 | 1.19e-03 |
| Suffering | 0.38 (0.35,0.40) | 0.40 (0.38,0.42) | 0.55 * | 2.61e-01 | 2.43e-01 |
| *Social Distress* | | | | | |
| Loneliness | 3.25 (3.09,3.42) | 3.36 (3.22,3.51) | 1.81 * | 1.11e-01 | 6.41e-02 |
| Discrimination | 0.25 (0.22,0.27) | 0.21 (0.20,0.23) | 0.48 * | 3.81e-02 | 2.9e-02 |
| *Character & Prosocial Behavior* | | | | | |
| Promoting Good | 8.67 (8.59,8.75) | 8.57 (8.51,8.64) | 8.24 * | 7.02e-02 | 1.59e-01 |
| Delayed Gratification | 8.33 (8.24,8.43) | 8.21 (8.11,8.30) | 8.66 * | 8.08e-02 | 1.25e-01 |
| Hope | 8.95 (8.86,9.04) | 9.05 (8.98,9.11) | 8.15 * | 3.99e-01 | 7.26e-02 |
| Gratitude | 8.70 (8.60,8.79) | 8.98 (8.91,9.05) | 7.94 * | 3.16e-03 | 3.3e-06 |
| Love | 8.63 (8.54,8.71) | 8.79 (8.71,8.88) | 7.18 * | 9.34e-02 | 6.98e-03 |
| Forgiveness | 0.78 (0.76,0.80) | 0.77 (0.75,0.79) | 0.68 * | 2.13e-01 | 6.97e-01 |
| Charitable Giving | 0.22 (0.20,0.24) | 0.19 (0.17,0.21) | 0.11 * | 4.62e-02 | 6.6e-02 |
| Helping | 0.67 (0.64,0.69) | 0.60 (0.57,0.62) | 0.79 * | 2.04e-04 | 2.27e-04 |
| Volunteering | 0.25 (0.22,0.27) | 0.19 (0.17,0.20) | 0.21 * | 6.04e-03 | 4.25e-05 |
| *Physical Health & Health Behavior* | | | | | |
| Self-Rated Physical Health | 7.99 (7.90,8.09) | 7.60 (7.51,7.69) | 6.25 * | 5.99e-04 | 7.05e-10 |
| Health Limitations | 0.15 (0.13,0.17) | 0.21 (0.19,0.23) | 0.30 * | 9.87e-02 | 1.24e-04 |
| Pain | 0.34 (0.32,0.37) | 0.41 (0.39,0.43) | 0.64 * | 5.21e-02 | 8.57e-05 |
| Smoking | 1.89 (1.62,2.17) | 0.58 (0.48,0.67) | 3.12 * | 7.39e-11 | 1.6e-16 |
| Drinking | 1.99 (1.78,2.19) | 0.78 (0.68,0.88) | 4.36 * | 2.48e-13 | 1.6e-16 |
| Exercise | 2.78 (2.64,2.91) | 2.15 (2.04,2.27) | 0.74 * | 6.22e-07 | 3.22e-15 |
| *Socioeconomic Outcomes* | | | | | |
| Financial Stability | 5.28 (5.09,5.47) | 4.51 (4.35,4.67) | 8.14 * | 5.27e-04 | 1.13e-11 |
| Material Stability | 5.19 (5.00,5.38) | 4.51 (4.34,4.67) | 8.03 * | 5.79e-04 | 4.87e-12 |
| Education | 0.23 (0.21,0.25) | 0.22 (0.20,0.24) | 0.00 * | 7.52e-01 | 1.6e-16 |
| Employment | 0.73 (0.70,0.75) | 0.36 (0.34,0.38) | 0.28 * | 1.6e-16 | 1.6e-16 |
| Subjective Financial Well-Being | 0.55 (0.53,0.58) | 0.43 (0.40,0.45) | 0.50 * | 3.77e-07 | 2.15e-13 |
| Housing | 0.61 (0.59,0.64) | 0.57 (0.55,0.60) | 0.58 * | 1.14e-01 | 5.92e-02 |
| *Religion/Spirituality* | | | | | |
| Self-Reported Religion/Spirituality | 0.65 (0.63,0.68) | 0.69 (0.67,0.71) | 0.57 * | 1.67e-01 | 7.37e-02 |
| Religious Service Attendance | 0.29 (0.26,0.31) | 0.36 (0.34,0.39) | 0.13 * | 5.66e-04 | 9.5e-06 |

| Outcome | Male | Female | Other | Male vs Female p-value | Global p-value |
|---|---|---|---|---|---|
| Life after Death Belief | 0.60 (0.58,0.63) | 0.60 (0.58,0.63) | 0.58 * | 6.28e-01 | 9.74e-01 |
| Religious Experience | 0.45 (0.43,0.48) | 0.44 (0.42,0.47) | 0.45 * | 1.15e-01 | 8.51e-01 |
| Religious Reading | 0.22 (0.20,0.24) | 0.24 (0.22,0.26) | 0.13 * | 6.21e-02 | 2.05e-01 |
| Prayer-Meditation | 0.44 (0.42,0.47) | 0.51 (0.49,0.53) | 0.59 * | 2.12e-03 | 7.49e-04 |
| Belief in God | 0.89 (0.88,0.91) | 0.93 (0.92,0.94) | 0.71 * | 4.52e-06 | 1.88e-05 |
| Intrinsic Religiosity | 0.72 (0.70,0.75) | 0.77 (0.75,0.79) | 0.45 * | 3.52e-02 | 7.21e-04 |
| Religious Comfort | 0.79 (0.76,0.81) | 0.86 (0.85,0.88) | 0.58 * | 7.45e-04 | 3.93e-08 |
| Loved by God | 0.85 (0.83,0.87) | 0.91 (0.89,0.92) | 0.46 * | 4.85e-05 | 8.48e-08 |
| Spiritual Punishment | 0.25 (0.23,0.28) | 0.21 (0.19,0.23) | 0.26 * | 6.74e-02 | 8.88e-03 |
| Religious Criticism | 0.43 (0.40,0.46) | 0.42 (0.38,0.46) | 0.40 * | 1.34e-01 | 6.18e-01 |
| Evangelism | 0.65 (0.62,0.67) | 0.64 (0.62,0.67) | 0.57 * | 1.99e-01 | 6.39e-01 |
| *Family Factors* | | | | | |
| Ever Married | 0.53 (0.51,0.56) | 0.55 (0.53,0.57) | 0.39 * | 1.37e-01 | 4.64e-01 |
| Divorced | 0.04 (0.03,0.05) | 0.04 (0.03,0.05) | 0.00 * | 8.64e-01 | 1.6e-16 |
| Children | 1.07 (0.98,1.15) | 1.26 (1.20,1.32) | 0.34 * | 9.83e-02 | 2.52e-10 |

**Table S13a. Nationally representative descriptive statistics for Nigeria**

| Characteristic | N = 6,827[1] |
|---|---|
| **Age group** | |
| 18-24 | 1,533 (22%) |
| 25-29 | 1,193 (17%) |
| 30-39 | 1,943 (28%) |
| 40-49 | 1,059 (16%) |
| 50-59 | 619 (9.1%) |
| 60-69 | 296 (4.3%) |
| 70-79 | 133 (2.0%) |
| 80 or older | 50 (0.7%) |
| (Missing) | 0 (0%) |
| **Gender** | |
| Male | 3,371 (49%) |
| Female | 3,456 (51%) |
| Other | 0 (<0.1%) |
| (Missing) | 0 (0%) |
| **Marital status** | |
| Married | 4,065 (60%) |
| Separated | 117 (1.7%) |
| Divorced | 71 (1.0%) |
| Widowed | 231 (3.4%) |
| Single, never married | 2,289 (34%) |
| Domestic Partner | 12 (0.2%) |
| (Missing) | 42 (0.6%) |
| **Employment** | |
| Employed for an employer | 699 (10%) |
| Self-employed | 3,898 (57%) |
| Retired | 178 (2.6%) |
| Student | 650 (9.5%) |
| Homemaker | 499 (7.3%) |
| Unemployed and looking for a job | 684 (10%) |
| None of these/Other | 211 (3.1%) |
| (Missing) | 8 (0.1%) |
| **Religious service attendance** | |
| More than 1/week | 4,049 (59%) |
| 1/week | 1,895 (28%) |
| 1-3/month | 531 (7.8%) |
| A few times a year | 254 (3.7%) |
| Never | 77 (1.1%) |
| (Missing) | 20 (0.3%) |
| **Education** | |
| Up to 8 years | 2,575 (38%) |
| 9-15 years | 4,120 (60%) |
| 16+ years | 130 (1.9%) |
| (Missing) | 2 (<0.1%) |
| **Immigration** | |
| Born in this country | 6,779 (99%) |
| Born in another country | 47 (0.7%) |
| (Missing) | 1 (<0.1%) |
| **Religious affiliation** | |
| Christianity | 3,476 (51%) |
| Islam | 3,302 (48%) |
| Hinduism | 0 (0%) |
| Buddhism | 0 (0%) |
| Judaism | 0 (0%) |
| Sikhism | 0 (0%) |
| Baha'i | 0 (0%) |
| Jainism | 0 (0%) |
| Shinto | 1 (<0.1%) |
| Taoism | 0 (0%) |
| Confucianism | 0 (<0.1%) |
| Primal, Animist, or Folk religion | 24 (0.3%) |
| Spiritism | 0 (0%) |
| Umbanda, Candomble, and other African-derived religions | 0 (0%) |
| Chinese folk/traditional religion | 0 (0%) |
| Some other religion | 1 (<0.1%) |
| No religion/Atheist/Agnostic | 15 (0.2%) |
| (Missing) | 9 (0.1%) |
| **Race/Ethnicity** | |

| Characteristic | N = 6,827[1] |
|---|---|
| (Missing) | 4 (<0.1%) |
| Edo | 116 (1.7%) |
| Efik | 48 (0.7%) |
| Fulani | 266 (3.9%) |
| Hausa | 2,342 (34%) |
| Ibibio | 180 (2.6%) |
| Idoma | 61 (0.9%) |
| Igala | 77 (1.1%) |
| Igbo (Ibo) | 1,111 (16%) |
| Ijaw | 110 (1.6%) |
| Kanuri | 31 (0.5%) |
| Other | 1,014 (15%) |
| Tiv | 198 (2.9%) |
| Urhobo | 38 (0.6%) |
| Yoruba | 1,230 (18%) |

[1]n (%)

**Table S13b. Descriptive statistics of outcome variables for Nigeria**

| Characteristic | N = 6,827[1] |
|---|---|
| **Flourishing Index** | 7.84 (1.45) |
| (Missing) | 163 |
| **Secure Flourishing Index** | 7.38 (1.39) |
| (Missing) | 179 |
| **Happiness & Life Satisfaction** | 6.78 (2.36) |
| (Missing) | 26 |
| **Social Relationship Quality** | 7.72 (2.16) |
| (Missing) | 29 |
| **Meaning and Purpose** | 7.90 (1.90) |
| (Missing) | 51 |
| **Character & Virtue** | 8.27 (1.83) |
| (Missing) | 57 |
| **Self-Rated Health** | 8.46 (1.85) |
| (Missing) | 25 |
| **Financial and Material Worry** | 5.1 (2.9) |
| (Missing) | 21 |
| **Happiness** | 7.06 (2.59) |
| (Missing) | 12 |
| **Life Satisfaction** | 6.5 (2.9) |
| (Missing) | 16 |
| **Present Life Evaluation** | 5.7 (2.8) |
| (Missing) | 19 |
| **Future Life Evaluation** | 8.25 (2.08) |
| (Missing) | 155 |
| **Optimism** | 8.90 (1.77) |
| (Missing) | 22 |
| **Freedom** | 8.16 (2.27) |
| (Missing) | 11 |
| **Peace** | |
| Always | 2,571 (38%) |
| Often | 2,773 (41%) |
| Rarely | 1,258 (18%) |
| Never | 197 (2.9%) |
| (Missing) | 28 (0.4%) |
| **Balance in Life** | |
| Always | 2,237 (33%) |
| Often | 2,634 (39%) |
| Rarely | 1,512 (22%) |
| Never | 413 (6.1%) |
| (Missing) | 30 (0.4%) |
| **Mastery** | |
| Always | 2,570 (38%) |
| Often | 2,918 (43%) |
| Rarely | 1,187 (17%) |
| Never | 129 (1.9%) |
| (Missing) | 23 (0.3%) |
| **Meaning** | 7.43 (2.54) |
| (Missing) | 23 |
| **Purpose** | 8.36 (2.11) |
| (Missing) | 29 |
| **Self-Rated Mental Health** | 8.66 (2.06) |
| (Missing) | 18 |
| **Content with My Relationships** | 7.83 (2.38) |
| (Missing) | 18 |
| **Satisfying Relationships** | 7.61 (2.45) |
| (Missing) | 12 |
| **Social Support** | 6.6 (2.9) |
| (Missing) | 28 |
| **Intimate Friend** | |
| Yes | 6,329 (93%) |
| No | 493 (7.2%) |
| (Missing) | 5 (<0.1%) |
| **Government Approval** | |
| Strongly approve | 1,178 (17%) |
| Somewhat approve | 1,494 (22%) |
| Neither approve nor disapprove | 894 (13%) |
| Somewhat disapprove | 1,433 (21%) |
| Strongly disapprove | 1,746 (26%) |

| Characteristic | N = 6,827[1] |
|---|---|
| (Missing) | 82 (1.2%) |
| **Political Voice** | |
| Agree | 2,783 (41%) |
| Disagree | 3,096 (45%) |
| Unsure | 933 (14%) |
| (Missing) | 15 (0.2%) |
| **Belonging** | 7.24 (2.81) |
| (Missing) | 78 |
| **City Satisfaction** | |
| Satisfied | 4,954 (73%) |
| Dissatisfied | 1,618 (24%) |
| Unsure | 250 (3.7%) |
| (Missing) | 5 (<0.1%) |
| **Trust** | |
| All | 166 (2.4%) |
| Most | 1,013 (15%) |
| Some | 2,268 (33%) |
| Not very many | 2,496 (37%) |
| None | 747 (11%) |
| (Missing) | 138 (2.0%) |
| **Community Participation** | |
| More than once a week | 1,044 (15%) |
| Once a week | 1,195 (18%) |
| One to three times a month | 1,240 (18%) |
| A few times a year | 1,666 (24%) |
| Never | 1,666 (24%) |
| (Missing) | 15 (0.2%) |
| **Traumatic Distress** | |
| A lot | 962 (14%) |
| Some | 1,707 (25%) |
| Not very much | 2,420 (35%) |
| None at all | 1,709 (25%) |
| (Missing) | 29 (0.4%) |
| **Suffering** | |
| A lot | 1,049 (15%) |
| Some | 1,764 (26%) |
| Not very much | 2,508 (37%) |
| None at all | 1,470 (22%) |
| (Missing) | 36 (0.5%) |
| **Loneliness** | 3.2 (2.9) |
| (Missing) | 24 |
| **Discrimination** | |
| Always | 902 (13%) |
| Often | 1,370 (20%) |
| Rarely | 2,466 (36%) |
| Never | 2,035 (30%) |
| (Missing) | 53 (0.8%) |
| **Promoting Good** | 8.21 (2.12) |
| (Missing) | 24 |
| **Delayed Gratification** | 8.32 (2.07) |
| (Missing) | 39 |
| **Hope** | 8.65 (1.97) |
| (Missing) | 10 |
| **Gratitude** | 8.42 (2.11) |
| (Missing) | 27 |
| **Love** | 8.38 (1.99) |
| (Missing) | 9 |
| **Forgiveness** | |
| Always | 4,276 (63%) |
| Often | 1,978 (29%) |
| Rarely | 476 (7.0%) |
| Never | 82 (1.2%) |
| (Missing) | 16 (0.2%) |
| **Charitable Giving** | |
| Yes | 3,259 (48%) |
| No | 3,555 (52%) |
| (Missing) | 13 (0.2%) |
| **Helping** | |
| Yes | 5,629 (82%) |

| Characteristic | N = 6,827[1] |
|---|---|
| No | 1,181 (17%) |
| (Missing) | 17 (0.2%) |
| **Volunteering** | |
| Yes | 3,444 (50%) |
| No | 3,369 (49%) |
| (Missing) | 13 (0.2%) |
| **Self-Rated Physical Health** | 8.27 (2.19) |
| (Missing) | 9 |
| **Health Limitations** | |
| Yes | 944 (14%) |
| No | 5,868 (86%) |
| (Missing) | 15 (0.2%) |
| **Pain** | |
| A lot | 834 (12%) |
| Some | 1,818 (27%) |
| Not very much | 2,665 (39%) |
| None at all | 1,500 (22%) |
| (Missing) | 9 (0.1%) |
| **Smoking** | 0.2571 (2.5251) |
| (Missing) | 64 |
| **Drinking** | 0.55 (3.57) |
| (Missing) | 66 |
| **Exercise** | |
| 0 days | 1,717 (25%) |
| 1 day | 668 (9.8%) |
| 2 days | 944 (14%) |
| 3 days | 841 (12%) |
| 4 days | 499 (7.3%) |
| 5 days | 450 (6.6%) |
| 6 days | 220 (3.2%) |
| 7 days/Every day | 1,353 (20%) |
| (Missing) | 136 (2.0%) |
| **Financial Stability** | 5.0 (3.1) |
| (Missing) | 12 |
| **Material Stability** | 5.2 (3.2) |
| (Missing) | 9 |
| **Education** | |
| Up to 8 years | 2,575 (38%) |
| 9-15 years | 4,120 (60%) |
| 16+ years | 130 (1.9%) |
| (Missing) | 2 (<0.1%) |
| **Employment** | |
| Employed for an employer | 699 (10%) |
| Self-employed | 3,898 (57%) |
| Retired | 178 (2.6%) |
| Student | 650 (9.5%) |
| Homemaker | 499 (7.3%) |
| Unemployed and looking for a job | 684 (10%) |
| None of these/Other | 211 (3.1%) |
| (Missing) | 8 (0.1%) |
| **Subjective Financial Well-Being** | |
| Living comfortably on present income | 553 (8.1%) |
| Getting by on present income | 2,094 (31%) |
| Finding it difficult on present income | 2,450 (36%) |
| Finding it very difficult on present income | 1,645 (24%) |
| (Missing) | 84 (1.2%) |
| **Housing** | |
| Someone in this household OWNS this home | 3,004 (44%) |
| Someone in this household RENTS this home | 3,093 (45%) |
| Both | 342 (5.0%) |
| Neither | 353 (5.2%) |
| Rent | 0 (0%) |
| Own | 0 (0%) |
| Something else | 0 (0%) |
| (Missing) | 35 (0.5%) |
| **Self-Reported Religion/Spirituality** | |
| Always | 4,276 (63%) |
| Often | 1,793 (26%) |
| Rarely | 568 (8.3%) |

| Characteristic | N = 6,827[1] |
|---|---|
| Never | 175 (2.6%) |
| (Missing) | 15 (0.2%) |
| **Religious Service Attendance** | |
| More than once a week | 4,049 (59%) |
| Once a week | 1,895 (28%) |
| One to three times a month | 531 (7.8%) |
| A few times a year | 254 (3.7%) |
| Never | 77 (1.1%) |
| (Missing) | 20 (0.3%) |
| **Life after Death Belief** | |
| Yes | 4,971 (73%) |
| No | 1,443 (21%) |
| Unsure | 403 (5.9%) |
| (Missing) | 11 (0.2%) |
| **Religious Experience** | |
| Yes | 4,036 (59%) |
| No | 2,693 (39%) |
| (Missing) | 98 (1.4%) |
| **Religious Reading** | |
| More than once a day | 2,327 (34%) |
| About once a day | 1,971 (29%) |
| Sometimes | 2,164 (32%) |
| Never | 338 (4.9%) |
| (Missing) | 26 (0.4%) |
| **Prayer-Meditation** | |
| More than once a day | 4,947 (72%) |
| About once a day | 1,298 (19%) |
| Sometimes | 521 (7.6%) |
| Never | 46 (0.7%) |
| (Missing) | 15 (0.2%) |
| **Belief in God** | |
| One God | 6,652 (97%) |
| More than one god | 100 (1.5%) |
| An impersonal spiritual force | 28 (0.4%) |
| None of these | 22 (0.3%) |
| Unsure | 15 (0.2%) |
| (Missing) | 10 (0.1%) |
| **Intrinsic Religiosity** | |
| Agree | 6,077 (89%) |
| Disagree | 484 (7.1%) |
| Not relevant | 119 (1.7%) |
| Unsure | 142 (2.1%) |
| (Missing) | 5 (<0.1%) |
| **Religious Comfort** | |
| Agree | 6,387 (94%) |
| Disagree | 301 (4.4%) |
| Not relevant | 63 (0.9%) |
| Unsure | 72 (1.0%) |
| (Missing) | 4 (<0.1%) |
| **Loved by God** | |
| Agree | 6,452 (95%) |
| Disagree | 214 (3.1%) |
| Not relevant | 79 (1.2%) |
| Unsure | 72 (1.1%) |
| (Missing) | 10 (0.1%) |
| **Spiritual Punishment** | |
| Agree | 1,748 (26%) |
| Disagree | 4,000 (59%) |
| Not relevant | 540 (7.9%) |
| Unsure | 508 (7.4%) |
| (Missing) | 30 (0.4%) |
| **Religious Criticism** | |
| Agree | 3,345 (49%) |
| Disagree | 2,339 (34%) |
| Not relevant | 558 (8.2%) |
| Unsure | 566 (8.3%) |
| (Missing) | 19 (0.3%) |
| **Evangelism** | |
| Agree | 5,217 (76%) |

| Characteristic | N = 6,827[1] |
|---|---|
| Disagree | 1,114 (16%) |
| Not relevant | 341 (5.0%) |
| Unsure | 148 (2.2%) |
| (Missing) | 6 (<0.1%) |
| **Children** | 2.84 (2.61) |
| (Missing) | 27 |

[1]Mean (SD); n (%)

**Table S13c. Demographic variation across outcomes for Nigeria**

| Outcome | Male | Female | Other | Male vs Female p-value | Global p-value |
|---|---|---|---|---|---|
| *Flourishing Index and Domains* | | | | | |
| Flourishing Index | 7.78 (7.70,7.87) | 7.86 (7.79,7.93) | 8.30 * | 4.48e-01 | 1.6e-16 |
| Secure Flourishing Index | 7.34 (7.26,7.42) | 7.41 (7.34,7.48) | 8.17 * | 6.81e-01 | 1.6e-16 |
| Happiness & Life Satisfaction | 6.71 (6.57,6.84) | 6.86 (6.74,6.99) | 6.50 * | 3.81e-01 | 7.23e-08 |
| Social Relationship Quality | 7.70 (7.59,7.81) | 7.74 (7.64,7.84) | 10.00 * | 7.7e-01 | 1.6e-16 |
| Meaning and Purpose | 7.86 (7.75,7.97) | 7.93 (7.83,8.03) | 7.00 * | 5.42e-01 | 1.6e-16 |
| Character & Virtue | 8.23 (8.11,8.34) | 8.30 (8.21,8.39) | 8.50 * | 8.98e-01 | 1.09e-07 |
| Self-Rated Health | 8.43 (8.32,8.53) | 8.49 (8.40,8.58) | 9.50 * | 1.28e-01 | 1.6e-16 |
| Financial and Material Worry | 5.10 (4.96,5.24) | 5.13 (4.99,5.28) | 7.50 * | 5.03e-01 | 1.6e-16 |
| *Psychological Well-Being* | | | | | |
| Happiness | 6.98 (6.85,7.12) | 7.14 (7.01,7.28) | 6.00 * | 3.68e-01 | 1.6e-16 |
| Life Satisfaction | 6.43 (6.27,6.59) | 6.59 (6.44,6.74) | 7.00 * | 5.32e-01 | 5.96e-12 |
| Present Life Evaluation | 5.68 (5.52,5.84) | 5.78 (5.62,5.94) | 5.00 * | 4.26e-01 | 1.6e-16 |
| Future Life Evaluation | 8.22 (8.10,8.34) | 8.26 (8.14,8.37) | 8.00 * | 3.22e-01 | 2.05e-06 |
| Optimism | 8.82 (8.72,8.93) | 8.97 (8.88,9.06) | 9.00 * | 8.5e-02 | 3.82e-03 |
| Freedom | 8.20 (8.09,8.32) | 8.12 (8.00,8.25) | 7.00 * | 8.56e-01 | 1.6e-16 |
| Peace | 0.78 (0.76,0.80) | 0.79 (0.76,0.81) | 1.00 * | 5.36e-01 | 1.6e-16 |
| Balance in Life | 0.74 (0.71,0.76) | 0.70 (0.67,0.73) | 1.00 * | 1.49e-01 | 1.6e-16 |
| Mastery | 0.81 (0.79,0.83) | 0.80 (0.78,0.82) | 1.00 * | 1.71e-01 | 1.6e-16 |
| Meaning | 7.40 (7.27,7.54) | 7.45 (7.31,7.58) | 8.00 * | 9.4e-01 | 1.6e-16 |
| Purpose | 8.31 (8.19,8.43) | 8.41 (8.31,8.51) | 6.00 * | 2.94e-01 | 1.6e-16 |
| Self-Rated Mental Health | 8.57 (8.45,8.68) | 8.73 (8.65,8.82) | 10.00 * | 5.46e-02 | 1.6e-16 |
| *Social Well-Being* | | | | | |
| Content with My Relationships | 7.79 (7.66,7.91) | 7.86 (7.74,7.97) | 10.00 * | 6.08e-01 | 1.6e-16 |
| Satisfying Relationships | 7.61 (7.49,7.73) | 7.62 (7.50,7.73) | 10.00 * | 3.19e-01 | 1.6e-16 |
| Social Support | 6.50 (6.33,6.67) | 6.77 (6.64,6.91) | 7.00 * | 2.1e-01 | 5.45e-09 |
| Intimate Friend | 0.92 (0.91,0.94) | 0.93 (0.92,0.95) | 1.00 * | 2.78e-01 | 1.6e-16 |
| Government Approval | 0.39 (0.36,0.42) | 0.40 (0.37,0.43) | 1.00 * | 9.5e-01 | 1.6e-16 |
| Political Voice | 0.48 (0.46,0.51) | 0.44 (0.41,0.47) | 1.00 * | 3.49e-02 | 1.6e-16 |
| Belonging | 7.23 (7.08,7.39) | 7.24 (7.09,7.40) | 8.00 * | 8.94e-01 | 1.6e-16 |
| City Satisfaction | 0.77 (0.74,0.79) | 0.74 (0.71,0.77) | 0.00 * | 2.63e-01 | 1.6e-16 |
| Trust | 0.17 (0.15,0.19) | 0.18 (0.15,0.20) | 0.00 * | 4.57e-01 | 1.6e-16 |
| Community Participation | 0.37 (0.35,0.39) | 0.29 (0.27,0.31) | 0.00 * | 8.13e-05 | 1.6e-16 |
| *Psychological Distress* | | | | | |
| Traumatic Distress | 0.41 (0.38,0.44) | 0.37 (0.35,0.40) | 0.00 * | 1.68e-01 | 1.6e-16 |
| Depression Symptoms | 0.46 (0.43,0.49) | 0.43 (0.40,0.46) | 1.00 * | 1.85e-01 | 1.6e-16 |
| Anxiety Symptoms | 0.37 (0.35,0.40) | 0.36 (0.33,0.39) | 1.00 * | 2.01e-01 | 1.6e-16 |
| Suffering | 0.42 (0.39,0.44) | 0.41 (0.39,0.44) | 1.00 * | 5.57e-01 | 1.6e-16 |
| *Social Distress* | | | | | |
| Loneliness | 3.24 (3.08,3.39) | 3.21 (3.05,3.36) | 7.00 * | 7.15e-01 | 1.6e-16 |
| Discrimination | 0.36 (0.33,0.38) | 0.31 (0.29,0.34) | 1.00 * | 3.85e-02 | 1.6e-16 |
| *Character & Prosocial Behavior* | | | | | |
| Promoting Good | 8.19 (8.07,8.32) | 8.22 (8.11,8.34) | 8.00 * | 8.39e-01 | 5.33e-05 |
| Delayed Gratification | 8.26 (8.14,8.38) | 8.38 (8.29,8.47) | 9.00 * | 6.85e-01 | 1.6e-16 |
| Hope | 8.62 (8.50,8.73) | 8.68 (8.57,8.78) | 9.00 * | 2.56e-01 | 1.6e-14 |
| Gratitude | 8.35 (8.22,8.48) | 8.48 (8.38,8.58) | 9.00 * | 2.78e-01 | 1.6e-16 |
| Love | 8.29 (8.19,8.38) | 8.46 (8.37,8.56) | 8.00 * | 4.14e-02 | 1.6e-16 |
| Forgiveness | 0.92 (0.90,0.93) | 0.92 (0.91,0.93) | 1.00 * | 1.85e-01 | 1.6e-16 |
| Charitable Giving | 0.53 (0.49,0.56) | 0.43 (0.40,0.46) | 1.00 * | 4.77e-04 | 1.6e-16 |
| Helping | 0.85 (0.83,0.87) | 0.81 (0.78,0.83) | 1.00 * | 2.9e-02 | 1.6e-16 |
| Volunteering | 0.58 (0.55,0.61) | 0.43 (0.40,0.47) | 1.00 * | 1.63e-06 | 1.6e-16 |
| *Physical Health & Health Behavior* | | | | | |
| Self-Rated Physical Health | 8.28 (8.17,8.40) | 8.25 (8.12,8.38) | 9.00 * | 4.65e-01 | 1.6e-16 |
| Health Limitations | 0.14 (0.12,0.16) | 0.14 (0.12,0.16) | 0.00 * | 2.45e-01 | 1.6e-16 |
| Pain | 0.39 (0.36,0.41) | 0.39 (0.37,0.42) | 0.00 * | 7.85e-01 | 1.6e-16 |
| Smoking | 0.42 (0.31,0.52) | 0.05 (0.02,0.09) | 0.00 * | 2.39e-10 | 1.86e-13 |
| Drinking | 0.67 (0.58,0.76) | 0.36 (0.18,0.53) | 0.00 * | 9.37e-10 | 1.6e-16 |
| Exercise | 3.43 (3.29,3.57) | 2.59 (2.43,2.74) | 3.00 * | 3.48e-11 | 3.33e-16 |
| *Socioeconomic Outcomes* | | | | | |
| Financial Stability | 4.97 (4.82,5.12) | 5.02 (4.86,5.19) | 7.00 * | 7.42e-01 | 1.6e-16 |
| Material Stability | 5.23 (5.07,5.40) | 5.24 (5.08,5.40) | 8.00 * | 3.61e-01 | 1.6e-16 |
| Education | 0.02 (0.02,0.03) | 0.02 (0.01,0.02) | 0.00 * | 1.36e-02 | 1.6e-16 |
| Employment | 0.72 (0.70,0.75) | 0.63 (0.60,0.66) | 1.00 * | 1.85e-05 | 1.6e-16 |
| Subjective Financial Well-Being | 0.39 (0.36,0.41) | 0.40 (0.37,0.43) | 0.00 * | 6.69e-01 | 1.6e-16 |
| Housing | 0.50 (0.48,0.53) | 0.48 (0.45,0.51) | 0.00 * | 1.63e-01 | 1.6e-16 |
| *Religion/Spirituality* | | | | | |
| Self-Reported Religion/Spirituality | 0.88 (0.86,0.90) | 0.90 (0.88,0.92) | 1.00 * | 6.06e-02 | 1.6e-16 |
| Religious Service Attendance | 0.87 (0.85,0.89) | 0.88 (0.86,0.89) | 1.00 * | 4.53e-01 | 1.6e-16 |

| Outcome | Male | Female | Other | Male vs Female p-value | Global p-value |
|---|---|---|---|---|---|
| Life after Death Belief | 0.76 (0.73,0.78) | 0.70 (0.68,0.72) | 0.00 * | 1.28e-03 | 1.6e-16 |
| Religious Experience | 0.60 (0.57,0.62) | 0.60 (0.57,0.63) | 1.00 * | 7.89e-01 | 1.6e-16 |
| Religious Reading | 0.62 (0.60,0.65) | 0.64 (0.61,0.66) | 1.00 * | 5.13e-01 | 1.6e-16 |
| Prayer-Meditation | 0.90 (0.88,0.91) | 0.93 (0.92,0.94) | 1.00 * | 6.5e-02 | 1.6e-16 |
| Belief in God | 0.99 (0.99,1.00) | 1.00 (1.00,1.00) | 1.00 * | 1.94e-01 | 1.22e-03 |
| Intrinsic Religiosity | 0.92 (0.90,0.93) | 0.93 (0.91,0.94) | 1.00 * | 6.09e-01 | 1.6e-16 |
| Religious Comfort | 0.95 (0.93,0.96) | 0.96 (0.95,0.97) | 1.00 * | 1.55e-02 | 1.6e-16 |
| Loved by God | 0.96 (0.95,0.97) | 0.97 (0.96,0.98) | 1.00 * | 3.95e-04 | 1.6e-16 |
| Spiritual Punishment | 0.31 (0.28,0.33) | 0.30 (0.27,0.33) | 1.00 * | 4.95e-01 | 1.6e-16 |
| Religious Criticism | 0.58 (0.55,0.61) | 0.57 (0.54,0.60) | 1.00 * | 8.46e-01 | 1.6e-16 |
| Evangelism | 0.80 (0.78,0.83) | 0.83 (0.81,0.85) | 1.00 * | 8.08e-02 | 1.6e-16 |
| *Family Factors* | | | | | |
| Ever Married | 0.63 (0.60,0.66) | 0.69 (0.66,0.71) | 1.00 * | 1.6e-02 | 1.6e-16 |
| Divorced | 0.01 (0.01,0.02) | 0.01 (0.01,0.01) | 0.00 * | 1.18e-01 | 4.75e-08 |
| Children | 2.97 (2.78,3.15) | 2.72 (2.49,2.96) | 2.00 * | 2.21e-01 | 1.6e-16 |

**Table S14a. Nationally representative descriptive statistics for Philippines**

| Characteristic | N = 5,292[1] |
|---|---|
| **Age group** | |
| 18-24 | 1,073 (20%) |
| 25-29 | 695 (13%) |
| 30-39 | 1,160 (22%) |
| 40-49 | 972 (18%) |
| 50-59 | 732 (14%) |
| 60-69 | 495 (9.4%) |
| 70-79 | 143 (2.7%) |
| 80 or older | 23 (0.4%) |
| (Missing) | 0 (0%) |
| **Gender** | |
| Male | 2,625 (50%) |
| Female | 2,643 (50%) |
| Other | 13 (0.2%) |
| (Missing) | 11 (0.2%) |
| **Marital status** | |
| Married | 2,385 (45%) |
| Separated | 249 (4.7%) |
| Divorced | 9 (0.2%) |
| Widowed | 274 (5.2%) |
| Single, never married | 1,206 (23%) |
| Domestic Partner | 1,152 (22%) |
| (Missing) | 16 (0.3%) |
| **Employment** | |
| Employed for an employer | 1,350 (26%) |
| Self-employed | 1,379 (26%) |
| Retired | 158 (3.0%) |
| Student | 585 (11%) |
| Homemaker | 1,049 (20%) |
| Unemployed and looking for a job | 658 (12%) |
| None of these/Other | 113 (2.1%) |
| (Missing) | 0 (0%) |
| **Religious service attendance** | |
| More than 1/week | 844 (16%) |
| 1/week | 1,929 (36%) |
| 1-3/month | 1,374 (26%) |
| A few times a year | 929 (18%) |
| Never | 210 (4.0%) |
| (Missing) | 6 (0.1%) |
| **Education** | |
| Up to 8 years | 1,188 (22%) |
| 9-15 years | 3,722 (70%) |
| 16+ years | 381 (7.2%) |
| (Missing) | 1 (<0.1%) |
| **Immigration** | |
| Born in this country | 5,284 (100%) |
| Born in another country | 8 (0.1%) |
| (Missing) | 0 (0%) |
| **Religious affiliation** | |
| Christianity | 4,914 (93%) |
| Islam | 297 (5.6%) |
| Hinduism | 0 (0%) |
| Buddhism | 4 (<0.1%) |
| Judaism | 4 (<0.1%) |
| Sikhism | 0 (0%) |
| Baha'i | 1 (<0.1%) |
| Jainism | 0 (0%) |
| Shinto | 0 (0%) |
| Taoism | 0 (0%) |
| Confucianism | 0 (0%) |
| Primal, Animist, or Folk religion | 5 (<0.1%) |
| Spiritism | 0 (0%) |
| Umbanda, Candomble, and other African-derived religions | 0 (0%) |
| Chinese folk/traditional religion | 0 (0%) |
| Some other religion | 35 (0.7%) |
| No religion/Atheist/Agnostic | 23 (0.4%) |
| (Missing) | 9 (0.2%) |
| **Race/Ethnicity** | |

| **Characteristic** | **N = 5,292**[1] |
|---|---|
| (Missing) | 3 (<0.1%) |
| Aeta | 1 (<0.1%) |
| Badjao | 2 (<0.1%) |
| Bicolano/Bikolano | 300 (5.7%) |
| Cebuano | 656 (12%) |
| Chinese-Filipino | 3 (<0.1%) |
| Igorot | 42 (0.8%) |
| Ilocano/Ilokano | 429 (8.1%) |
| Ilonggo/Hiligaynon | 428 (8.1%) |
| Kapampangan | 107 (2.0%) |
| Maguindanaoan | 84 (1.6%) |
| Mangyan | 2 (<0.1%) |
| Maranao | 39 (0.7%) |
| Masbateno | 54 (1.0%) |
| Other | 244 (4.6%) |
| Pangasinense | 107 (2.0%) |
| Tagalog | 1,691 (32%) |
| Tausug | 94 (1.8%) |
| Visayan/Bisaya | 739 (14%) |
| Waray | 216 (4.1%) |
| Zamboangueno | 51 (1.0%) |

[1]n (%)

**Table S14b. Descriptive statistics of outcome variables for Philippines**

| Characteristic | N = 5,292[1] |
|---|---|
| **Flourishing Index** | 8.11 (1.44) |
| (Missing) | 42 |
| **Secure Flourishing Index** | 7.70 (1.42) |
| (Missing) | 51 |
| **Happiness & Life Satisfaction** | 7.41 (2.12) |
| (Missing) | 8 |
| **Social Relationship Quality** | 8.55 (1.83) |
| (Missing) | 18 |
| **Meaning and Purpose** | 8.23 (1.83) |
| (Missing) | 8 |
| **Character & Virtue** | 8.48 (1.75) |
| (Missing) | 11 |
| **Self-Rated Health** | 7.87 (1.83) |
| (Missing) | 7 |
| **Financial and Material Worry** | 5.7 (2.9) |
| (Missing) | 12 |
| **Happiness** | 7.33 (2.33) |
| (Missing) | 3 |
| **Life Satisfaction** | 7.50 (2.43) |
| (Missing) | 5 |
| **Present Life Evaluation** | 6.38 (2.40) |
| (Missing) | 4 |
| **Future Life Evaluation** | 7.55 (2.24) |
| (Missing) | 10 |
| **Optimism** | 8.80 (2.00) |
| (Missing) | 10 |
| **Freedom** | 8.59 (2.00) |
| (Missing) | 7 |
| **Peace** | |
| Always | 1,178 (22%) |
| Often | 1,481 (28%) |
| Rarely | 2,150 (41%) |
| Never | 480 (9.1%) |
| (Missing) | 3 (<0.1%) |
| **Balance in Life** | |
| Always | 1,055 (20%) |
| Often | 1,615 (31%) |
| Rarely | 2,171 (41%) |
| Never | 450 (8.5%) |
| (Missing) | 0 (<0.1%) |
| **Mastery** | |
| Always | 1,646 (31%) |
| Often | 1,661 (31%) |
| Rarely | 1,743 (33%) |
| Never | 239 (4.5%) |
| (Missing) | 2 (<0.1%) |
| **Meaning** | 8.02 (2.33) |
| (Missing) | 3 |
| **Purpose** | 8.45 (2.02) |
| (Missing) | 5 |
| **Self-Rated Mental Health** | 8.05 (2.02) |
| (Missing) | 5 |
| **Content with My Relationships** | 8.66 (2.01) |
| (Missing) | 8 |
| **Satisfying Relationships** | 8.46 (2.03) |
| (Missing) | 14 |
| **Social Support** | 8.12 (2.31) |
| (Missing) | 5 |
| **Intimate Friend** | |
| Yes | 4,763 (90%) |
| No | 522 (9.9%) |
| (Missing) | 8 (0.1%) |
| **Government Approval** | |
| Strongly approve | 1,540 (29%) |
| Somewhat approve | 2,145 (41%) |
| Neither approve nor disapprove | 762 (14%) |
| Somewhat disapprove | 503 (9.5%) |
| Strongly disapprove | 329 (6.2%) |

| Characteristic | N = 5,292[1] |
|---|---|
| (Missing) | 14 (0.3%) |
| **Political Voice** | |
| Agree | 2,418 (46%) |
| Disagree | 400 (7.5%) |
| Unsure | 2,458 (46%) |
| (Missing) | 16 (0.3%) |
| **Belonging** | 8.14 (2.20) |
| (Missing) | 20 |
| **City Satisfaction** | |
| Satisfied | 4,565 (86%) |
| Dissatisfied | 178 (3.4%) |
| Unsure | 548 (10%) |
| (Missing) | 1 (<0.1%) |
| **Trust** | |
| All | 489 (9.2%) |
| Most | 2,079 (39%) |
| Some | 1,924 (36%) |
| Not very many | 685 (13%) |
| None | 107 (2.0%) |
| (Missing) | 9 (0.2%) |
| **Community Participation** | |
| More than once a week | 289 (5.5%) |
| Once a week | 702 (13%) |
| One to three times a month | 887 (17%) |
| A few times a year | 1,495 (28%) |
| Never | 1,913 (36%) |
| (Missing) | 6 (0.1%) |
| **Traumatic Distress** | |
| A lot | 777 (15%) |
| Some | 1,659 (31%) |
| Not very much | 1,979 (37%) |
| None at all | 874 (17%) |
| (Missing) | 4 (<0.1%) |
| **Suffering** | |
| A lot | 694 (13%) |
| Some | 2,431 (46%) |
| Not very much | 1,906 (36%) |
| None at all | 254 (4.8%) |
| (Missing) | 8 (0.2%) |
| **Loneliness** | 3.5 (3.2) |
| (Missing) | 3 |
| **Discrimination** | |
| Always | 514 (9.7%) |
| Often | 868 (16%) |
| Rarely | 2,610 (49%) |
| Never | 1,293 (24%) |
| (Missing) | 7 (0.1%) |
| **Promoting Good** | 8.54 (1.96) |
| (Missing) | 2 |
| **Delayed Gratification** | 8.42 (1.97) |
| (Missing) | 9 |
| **Hope** | 8.92 (1.82) |
| (Missing) | 8 |
| **Gratitude** | 8.64 (2.00) |
| (Missing) | 8 |
| **Love** | 9.05 (1.71) |
| (Missing) | 7 |
| **Forgiveness** | |
| Always | 2,522 (48%) |
| Often | 1,412 (27%) |
| Rarely | 1,134 (21%) |
| Never | 223 (4.2%) |
| (Missing) | 2 (<0.1%) |
| **Charitable Giving** | |
| Yes | 734 (14%) |
| No | 4,553 (86%) |
| (Missing) | 4 (<0.1%) |
| **Helping** | |
| Yes | 3,346 (63%) |

| Characteristic | N = 5,292[1] |
|---|---|
| No | 1,926 (36%) |
| (Missing) | 20 (0.4%) |
| **Volunteering** | |
| Yes | 1,349 (25%) |
| No | 3,941 (74%) |
| (Missing) | 2 (<0.1%) |
| **Self-Rated Physical Health** | 7.69 (2.14) |
| (Missing) | 4 |
| **Health Limitations** | |
| Yes | 1,817 (34%) |
| No | 3,458 (65%) |
| (Missing) | 17 (0.3%) |
| **Pain** | |
| A lot | 581 (11%) |
| Some | 1,693 (32%) |
| Not very much | 2,262 (43%) |
| None at all | 752 (14%) |
| (Missing) | 4 (<0.1%) |
| **Smoking** | 1.7 (4.6) |
| (Missing) | 81 |
| **Drinking** | 0.96 (4.17) |
| (Missing) | 95 |
| **Exercise** | |
| 0 days | 755 (14%) |
| 1 day | 943 (18%) |
| 2 days | 581 (11%) |
| 3 days | 520 (9.8%) |
| 4 days | 175 (3.3%) |
| 5 days | 122 (2.3%) |
| 6 days | 83 (1.6%) |
| 7 days/Every day | 2,103 (40%) |
| (Missing) | 8 (0.1%) |
| **Financial Stability** | 5.4 (3.3) |
| (Missing) | 2 |
| **Material Stability** | 6.0 (3.3) |
| (Missing) | 10 |
| **Education** | |
| Up to 8 years | 1,188 (22%) |
| 9-15 years | 3,722 (70%) |
| 16+ years | 381 (7.2%) |
| (Missing) | 1 (<0.1%) |
| **Employment** | |
| Employed for an employer | 1,350 (26%) |
| Self-employed | 1,379 (26%) |
| Retired | 158 (3.0%) |
| Student | 585 (11%) |
| Homemaker | 1,049 (20%) |
| Unemployed and looking for a job | 658 (12%) |
| None of these/Other | 113 (2.1%) |
| (Missing) | 0 (0%) |
| **Subjective Financial Well-Being** | |
| Living comfortably on present income | 417 (7.9%) |
| Getting by on present income | 2,147 (41%) |
| Finding it difficult on present income | 1,952 (37%) |
| Finding it very difficult on present income | 758 (14%) |
| (Missing) | 19 (0.4%) |
| **Housing** | |
| Someone in this household OWNS this home | 2,846 (54%) |
| Someone in this household RENTS this home | 692 (13%) |
| Both | 419 (7.9%) |
| Neither | 1,324 (25%) |
| Rent | 0 (0%) |
| Own | 0 (0%) |
| Something else | 0 (0%) |
| (Missing) | 10 (0.2%) |
| **Self-Reported Religion/Spirituality** | |
| Always | 2,094 (40%) |
| Often | 1,294 (24%) |
| Rarely | 1,550 (29%) |

| Characteristic | N = 5,292[1] |
|---|---|
| Never | 353 (6.7%) |
| (Missing) | 2 (<0.1%) |
| **Religious Service Attendance** | |
| More than once a week | 844 (16%) |
| Once a week | 1,929 (36%) |
| One to three times a month | 1,374 (26%) |
| A few times a year | 929 (18%) |
| Never | 210 (4.0%) |
| (Missing) | 6 (0.1%) |
| **Life after Death Belief** | |
| Yes | 2,839 (54%) |
| No | 446 (8.4%) |
| Unsure | 1,993 (38%) |
| (Missing) | 14 (0.3%) |
| **Religious Experience** | |
| Yes | 3,269 (62%) |
| No | 2,014 (38%) |
| (Missing) | 10 (0.2%) |
| **Religious Reading** | |
| More than once a day | 583 (11%) |
| About once a day | 771 (15%) |
| Sometimes | 3,589 (68%) |
| Never | 332 (6.3%) |
| (Missing) | 18 (0.3%) |
| **Prayer-Meditation** | |
| More than once a day | 1,972 (37%) |
| About once a day | 1,781 (34%) |
| Sometimes | 1,426 (27%) |
| Never | 90 (1.7%) |
| (Missing) | 23 (0.4%) |
| **Belief in God** | |
| One God | 4,545 (86%) |
| More than one god | 271 (5.1%) |
| An impersonal spiritual force | 83 (1.6%) |
| None of these | 74 (1.4%) |
| Unsure | 311 (5.9%) |
| (Missing) | 7 (0.1%) |
| **Intrinsic Religiosity** | |
| Agree | 3,912 (74%) |
| Disagree | 283 (5.3%) |
| Not relevant | 184 (3.5%) |
| Unsure | 905 (17%) |
| (Missing) | 8 (0.2%) |
| **Religious Comfort** | |
| Agree | 4,234 (80%) |
| Disagree | 265 (5.0%) |
| Not relevant | 184 (3.5%) |
| Unsure | 604 (11%) |
| (Missing) | 5 (<0.1%) |
| **Loved by God** | |
| Agree | 4,793 (91%) |
| Disagree | 144 (2.7%) |
| Not relevant | 85 (1.6%) |
| Unsure | 264 (5.0%) |
| (Missing) | 7 (0.1%) |
| **Spiritual Punishment** | |
| Agree | 2,369 (45%) |
| Disagree | 1,310 (25%) |
| Not relevant | 356 (6.7%) |
| Unsure | 1,245 (24%) |
| (Missing) | 12 (0.2%) |
| **Religious Criticism** | |
| Agree | 1,791 (34%) |
| Disagree | 1,364 (26%) |
| Not relevant | 473 (8.9%) |
| Unsure | 1,656 (31%) |
| (Missing) | 9 (0.2%) |
| **Evangelism** | |
| Agree | 2,950 (56%) |

| **Characteristic** | **N = 5,292**[1] |
|---|---|
| Disagree | 777 (15%) |
| Not relevant | 432 (8.2%) |
| Unsure | 1,128 (21%) |
| (Missing) | 5 (<0.1%) |
| **Children** | 1.84 (1.62) |

[1]Mean (SD); n (%)

**Table S14c. Demographic variation across outcomes for Philippines**

| Outcome | Male | Female | Other | Male vs Female p-value | Global p-value |
|---|---|---|---|---|---|
| *Flourishing Index and Domains* | | | | | |
| Flourishing Index | 8.09 (8.01,8.17) | 8.14 (8.08,8.19) | 7.30 (6.41,8.20) | 7.32e-01 | 7.25e-02 |
| Secure Flourishing Index | 7.73 (7.65,7.81) | 7.69 (7.64,7.75) | 6.70 (6.01,7.39) | 3.05e-01 | 2.73e-03 |
| Happiness & Life Satisfaction | 7.38 (7.26,7.51) | 7.45 (7.37,7.53) | 6.48 (5.17,7.78) | 7.27e-01 | 1.51e-01 |
| Social Relationship Quality | 8.56 (8.46,8.67) | 8.56 (8.49,8.63) | 7.43 (6.08,8.77) | 5.09e-01 | 1.43e-01 |
| Meaning and Purpose | 8.23 (8.14,8.32) | 8.24 (8.16,8.31) | 7.51 (6.47,8.55) | 5.58e-01 | 2.62e-01 |
| Character & Virtue | 8.43 (8.33,8.53) | 8.54 (8.47,8.61) | 7.46 (6.40,8.52) | 2.71e-01 | 1.27e-02 |
| Self-Rated Health | 7.84 (7.74,7.94) | 7.90 (7.83,7.96) | 7.65 (6.37,8.93) | 6.74e-01 | 6.47e-01 |
| Financial and Material Worry | 5.92 (5.75,6.10) | 5.47 (5.35,5.59) | 3.68 (2.14,5.22) | 2.67e-02 | 8.54e-07 |
| *Psychological Well-Being* | | | | | |
| Happiness | 7.30 (7.16,7.44) | 7.36 (7.27,7.44) | 6.52 (5.14,7.89) | 9.15e-01 | 3.02e-01 |
| Life Satisfaction | 7.46 (7.32,7.60) | 7.55 (7.45,7.64) | 6.43 (5.17,7.70) | 6.19e-01 | 8.59e-02 |
| Present Life Evaluation | 6.31 (6.17,6.44) | 6.46 (6.37,6.55) | 6.21 (4.70,7.71) | 3.46e-01 | 1.91e-01 |
| Future Life Evaluation | 7.43 (7.31,7.55) | 7.66 (7.57,7.75) | 7.75 (6.52,8.99) | 1.36e-01 | 1.24e-02 |
| Optimism | 8.71 (8.59,8.82) | 8.88 (8.81,8.95) | 9.07 (8.19,9.95) | 7.25e-01 | 3.29e-02 |
| Freedom | 8.62 (8.52,8.73) | 8.57 (8.49,8.65) | 7.91 (6.88,8.94) | 6.94e-02 | 2.31e-01 |
| Peace | 0.51 (0.49,0.54) | 0.49 (0.47,0.51) | 0.50 (0.19,0.80) | 5.81e-01 | 3.98e-01 |
| Balance in Life | 0.53 (0.51,0.56) | 0.47 (0.46,0.49) | 0.58 (0.29,0.87) | 9.08e-02 | 4.08e-04 |
| Mastery | 0.64 (0.62,0.67) | 0.61 (0.59,0.63) | 0.62 (0.32,0.93) | 8.86e-01 | 1.17e-01 |
| Meaning | 8.04 (7.91,8.16) | 8.00 (7.91,8.10) | 7.34 (6.20,8.48) | 4.14e-01 | 3.52e-01 |
| Purpose | 8.43 (8.32,8.53) | 8.47 (8.39,8.55) | 7.68 (6.54,8.82) | 9.14e-01 | 2.18e-01 |
| Self-Rated Mental Health | 8.02 (7.91,8.13) | 8.08 (8.01,8.15) | 7.74 (6.34,9.13) | 7.35e-01 | 5.54e-01 |
| *Social Well-Being* | | | | | |
| Content with My Relationships | 8.66 (8.55,8.78) | 8.65 (8.58,8.73) | 7.75 (6.45,9.05) | 8.79e-01 | 2.6e-01 |
| Satisfying Relationships | 8.46 (8.34,8.57) | 8.46 (8.39,8.54) | 7.10 (5.59,8.62) | 2.82e-01 | 1.11e-01 |
| Social Support | 8.13 (8.01,8.26) | 8.10 (8.01,8.20) | 7.03 (5.36,8.70) | 1.59e-01 | 3.07e-01 |
| Intimate Friend | 0.90 (0.88,0.92) | 0.91 (0.90,0.92) | 0.78 (0.52,1.05) | 8.04e-01 | 3.68e-01 |
| Government Approval | 0.71 (0.68,0.73) | 0.69 (0.67,0.71) | 0.64 (0.35,0.93) | 3.92e-02 | 4.41e-01 |
| Political Voice | 0.84 (0.81,0.86) | 0.85 (0.83,0.87) | 0.78 (0.43,1.13) | 4.46e-01 | 3.49e-01 |
| Belonging | 8.18 (8.07,8.30) | 8.10 (8.02,8.19) | 7.88 (6.88,8.88) | 2.3e-01 | 4.47e-01 |
| City Satisfaction | 0.95 (0.94,0.96) | 0.97 (0.96,0.97) | 0.98 (0.81,1.14) | 1.5e-01 | 1.6e-16 |
| Trust | 0.48 (0.46,0.51) | 0.49 (0.47,0.51) | 0.26 (0.00,0.54) | 2.15e-01 | 1.56e-01 |
| Community Participation | 0.22 (0.20,0.25) | 0.15 (0.14,0.17) | 0.28 (0.02,0.55) | 4.28e-03 | 2.21e-05 |
| *Psychological Distress* | | | | | |
| Traumatic Distress | 0.46 (0.43,0.49) | 0.46 (0.44,0.48) | 0.60 (0.30,0.90) | 7.75e-01 | 5.16e-01 |
| Depression Symptoms | 0.48 (0.45,0.51) | 0.53 (0.51,0.55) | 0.73 (0.46,1.01) | 5.37e-01 | 4.27e-03 |
| Anxiety Symptoms | 0.45 (0.42,0.48) | 0.52 (0.50,0.54) | 0.59 (0.29,0.89) | 7.42e-03 | 7.16e-04 |
| Suffering | 0.59 (0.56,0.62) | 0.59 (0.57,0.61) | 0.78 (0.54,1.02) | 3.17e-01 | 1.74e-01 |
| *Social Distress* | | | | | |
| Loneliness | 3.40 (3.21,3.59) | 3.63 (3.50,3.75) | 5.47 (3.82,7.13) | 3.62e-01 | 2.61e-03 |
| Discrimination | 0.28 (0.26,0.30) | 0.24 (0.22,0.26) | 0.55 (0.24,0.86) | 5.21e-02 | 1.93e-03 |
| *Character & Prosocial Behavior* | | | | | |
| Promoting Good | 8.50 (8.40,8.61) | 8.58 (8.50,8.66) | 7.70 (6.73,8.67) | 1.83e-01 | 6.35e-02 |
| Delayed Gratification | 8.35 (8.25,8.46) | 8.50 (8.42,8.58) | 7.21 (5.60,8.82) | 5.17e-01 | 1.72e-02 |
| Hope | 8.88 (8.77,8.98) | 8.97 (8.89,9.04) | 8.41 (7.27,9.56) | 9.52e-01 | 1.82e-01 |
| Gratitude | 8.46 (8.35,8.58) | 8.81 (8.74,8.88) | 7.77 (6.05,9.48) | 2.46e-02 | 2.11e-06 |
| Love | 8.98 (8.88,9.08) | 9.11 (9.05,9.18) | 8.08 (7.08,9.08) | 5.51e-01 | 5.31e-03 |
| Forgiveness | 0.75 (0.73,0.78) | 0.73 (0.72,0.75) | 0.58 (0.27,0.88) | 6.47e-02 | 1.8e-01 |
| Charitable Giving | 0.15 (0.13,0.17) | 0.13 (0.11,0.14) | 0.28 (0.02,0.54) | 8.99e-02 | 3.71e-02 |
| Helping | 0.65 (0.62,0.68) | 0.62 (0.60,0.64) | 0.83 (0.61,1.05) | 1.5e-01 | 8.95e-03 |
| Volunteering | 0.28 (0.25,0.30) | 0.23 (0.21,0.25) | 0.50 (0.19,0.81) | 3.68e-02 | 3.21e-03 |
| *Physical Health & Health Behavior* | | | | | |
| Self-Rated Physical Health | 7.67 (7.55,7.79) | 7.71 (7.63,7.79) | 7.56 (6.31,8.81) | 6.76e-01 | 8.23e-01 |
| Health Limitations | 0.36 (0.33,0.38) | 0.33 (0.31,0.35) | 0.50 (0.19,0.81) | 1.27e-02 | 2.05e-01 |
| Pain | 0.43 (0.40,0.45) | 0.43 (0.42,0.45) | 0.49 (0.18,0.80) | 5.13e-01 | 7.77e-01 |
| Smoking | 3.14 (2.82,3.47) | 0.29 (0.24,0.34) | 0.28 (0.00,0.93) | 1.6e-16 | 1.6e-16 |
| Drinking | 1.42 (1.22,1.63) | 0.34 (0.29,0.39) | 4.40 (1.26,7.55) | 3.83e-13 | 1.6e-16 |
| Exercise | 4.07 (3.92,4.22) | 3.59 (3.48,3.70) | 1.76 (0.31,3.20) | 1.01e-03 | 1.27e-09 |
| *Socioeconomic Outcomes* | | | | | |
| Financial Stability | 5.67 (5.48,5.85) | 5.10 (4.97,5.22) | 3.16 (1.07,5.25) | 1.07e-02 | 2.29e-07 |
| Material Stability | 6.18 (5.99,6.37) | 5.84 (5.71,5.97) | 4.20 (2.73,5.67) | 1.47e-01 | 2.49e-04 |
| Education | 0.07 (0.05,0.08) | 0.08 (0.07,0.09) | 0.14 (0.00,0.35) | 1.9e-01 | 2.97e-01 |
| Employment | 0.68 (0.65,0.70) | 0.36 (0.34,0.38) | 0.35 (0.06,0.64) | 1.6e-16 | 1.6e-16 |
| Subjective Financial Well-Being | 0.50 (0.47,0.53) | 0.47 (0.46,0.49) | 0.41 (0.11,0.72) | 1.67e-01 | 3.06e-01 |
| Housing | 0.63 (0.60,0.65) | 0.61 (0.59,0.63) | 0.44 (0.14,0.73) | 3.76e-01 | 2.83e-01 |
| *Religion/Spirituality* | | | | | |
| Self-Reported Religion/Spirituality | 0.61 (0.59,0.64) | 0.67 (0.65,0.69) | 0.52 (0.21,0.82) | 3.48e-03 | 4.81e-03 |

| Outcome | Male | Female | Other | Male vs Female p-value | Global p-value |
|---|---|---|---|---|---|
| Religious Service Attendance | 0.49 (0.46,0.52) | 0.56 (0.54,0.58) | 0.36 (0.05,0.66) | 1.93e-01 | 7.22e-04 |
| Life after Death Belief | 0.52 (0.49,0.55) | 0.55 (0.54,0.57) | 0.81 (0.58,1.05) | 9.59e-02 | 7.26e-03 |
| Religious Experience | 0.63 (0.60,0.65) | 0.61 (0.59,0.63) | 0.70 (0.42,0.98) | 2.75e-01 | 5.83e-01 |
| Religious Reading | 0.24 (0.22,0.27) | 0.27 (0.25,0.29) | 0.40 (0.10,0.71) | 9.85e-02 | 1.41e-01 |
| Prayer-Meditation | 0.66 (0.63,0.68) | 0.77 (0.75,0.78) | 0.66 (0.36,0.96) | 2.65e-06 | 1.15e-12 |
| Belief in God | 0.92 (0.90,0.93) | 0.94 (0.93,0.95) | 0.85 (0.62,1.08) | 1.96e-01 | 6.15e-02 |
| Intrinsic Religiosity | 0.91 (0.89,0.93) | 0.92 (0.90,0.93) | 0.86 (0.68,1.05) | 6.02e-01 | 6.36e-01 |
| Religious Comfort | 0.92 (0.90,0.94) | 0.93 (0.92,0.94) | 0.79 (0.51,1.08) | 1.14e-01 | 1.03e-01 |
| Loved by God | 0.96 (0.95,0.97) | 0.97 (0.96,0.98) | 0.92 (0.76,1.09) | 4.13e-01 | 1.64e-01 |
| Spiritual Punishment | 0.71 (0.67,0.74) | 0.57 (0.55,0.59) | 0.57 (0.20,0.94) | 1.18e-10 | 2.86e-14 |
| Religious Criticism | 0.60 (0.57,0.63) | 0.55 (0.52,0.57) | 0.64 (0.22,1.07) | 4.44e-03 | 4.27e-03 |
| Evangelism | 0.78 (0.75,0.80) | 0.77 (0.75,0.79) | 0.77 (0.47,1.06) | 5.39e-01 | 5.92e-01 |
| *Family Factors* | | | | | |
| Ever Married | 0.52 (0.49,0.55) | 0.58 (0.56,0.61) | 0.15 (0.00,0.39) | 2e-02 | 1.32e-07 |
| Divorced | 0.00 (0.00,0.00) | 0.00 (0.00,0.00) | 0.00 * | 1.34e-01 | 9.02e-03 |
| Children | 1.73 (1.63,1.84) | 1.94 (1.87,2.01) | 1.01 (0.42,1.60) | 2.34e-04 | 1.85e-05 |

**Table S15a. Nationally representative descriptive statistics for Poland**

| Characteristic | N = 10,389[1] |
|---|---|
| **Age group** | |
| 18-24 | 955 (9.2%) |
| 25-29 | 761 (7.3%) |
| 30-39 | 2,159 (21%) |
| 40-49 | 1,956 (19%) |
| 50-59 | 1,670 (16%) |
| 60-69 | 1,909 (18%) |
| 70-79 | 833 (8.0%) |
| 80 or older | 145 (1.4%) |
| (Missing) | 1 (<0.1%) |
| **Gender** | |
| Male | 4,974 (48%) |
| Female | 5,387 (52%) |
| Other | 3 (<0.1%) |
| (Missing) | 26 (0.2%) |
| **Marital status** | |
| Married | 6,065 (58%) |
| Separated | 111 (1.1%) |
| Divorced | 529 (5.1%) |
| Widowed | 990 (9.5%) |
| Single, never married | 1,811 (17%) |
| Domestic Partner | 504 (4.8%) |
| (Missing) | 379 (3.6%) |
| **Employment** | |
| Employed for an employer | 5,837 (56%) |
| Self-employed | 686 (6.6%) |
| Retired | 2,434 (23%) |
| Student | 515 (5.0%) |
| Homemaker | 338 (3.3%) |
| Unemployed and looking for a job | 284 (2.7%) |
| None of these/Other | 169 (1.6%) |
| (Missing) | 126 (1.2%) |
| **Religious service attendance** | |
| More than 1/week | 305 (2.9%) |
| 1/week | 3,263 (31%) |
| 1-3/month | 2,081 (20%) |
| A few times a year | 3,064 (29%) |
| Never | 1,597 (15%) |
| (Missing) | 78 (0.8%) |
| **Education** | |
| Up to 8 years | 1,238 (12%) |
| 9-15 years | 6,130 (59%) |
| 16+ years | 3,020 (29%) |
| (Missing) | 1 (<0.1%) |
| **Immigration** | |
| Born in this country | 10,258 (99%) |
| Born in another country | 108 (1.0%) |
| (Missing) | 23 (0.2%) |
| **Religious affiliation** | |
| Christianity | 9,378 (90%) |
| Islam | 2 (<0.1%) |
| Hinduism | 0 (0%) |
| Buddhism | 2 (<0.1%) |
| Judaism | 0 (0%) |
| Sikhism | 1 (<0.1%) |
| Baha'i | 0 (0%) |
| Jainism | 3 (<0.1%) |
| Shinto | 1 (<0.1%) |
| Taoism | 0 (0%) |
| Confucianism | 0 (0%) |
| Primal, Animist, or Folk religion | 11 (0.1%) |
| Spiritism | 0 (0%) |
| Umbanda, Candomble, and other African-derived religions | 0 (0%) |
| Chinese folk/traditional religion | 0 (0%) |
| Some other religion | 0 (0%) |
| No religion/Atheist/Agnostic | 942 (9.1%) |
| (Missing) | 50 (0.5%) |
| **Race/Ethnicity** | |

| **Characteristic** | **N = 10,389**[1] |
|---|---|
| (Missing) | 14 (0.1%) |
| Belarussian | 2 (<0.1%) |
| German | 4 (<0.1%) |
| Kashubians | 3 (<0.1%) |
| Other | 4 (<0.1%) |
| Polish | 10,309 (99%) |
| Silesia | 14 (0.1%) |
| Ukrainian | 38 (0.4%) |

[1]n (%)

**Table S15b. Descriptive statistics of outcome variables for Poland**

| Characteristic | N = 10,389[1] |
|---|---|
| **Flourishing Index** | 7.64 (1.30) |
| (Missing) | 457 |
| **Secure Flourishing Index** | 7.56 (1.31) |
| (Missing) | 522 |
| **Happiness & Life Satisfaction** | 7.54 (1.56) |
| (Missing) | 124 |
| **Social Relationship Quality** | 7.66 (1.75) |
| (Missing) | 211 |
| **Meaning and Purpose** | 7.74 (1.67) |
| (Missing) | 88 |
| **Character & Virtue** | 7.36 (1.61) |
| (Missing) | 80 |
| **Self-Rated Health** | 7.87 (1.68) |
| (Missing) | 42 |
| **Financial and Material Worry** | 7.16 (2.26) |
| (Missing) | 98 |
| **Happiness** | 7.55 (1.60) |
| (Missing) | 15 |
| **Life Satisfaction** | 7.52 (1.73) |
| (Missing) | 110 |
| **Present Life Evaluation** | 7.12 (1.62) |
| (Missing) | 24 |
| **Future Life Evaluation** | 7.40 (1.77) |
| (Missing) | 145 |
| **Optimism** | 7.72 (1.83) |
| (Missing) | 32 |
| **Freedom** | 7.60 (1.87) |
| (Missing) | 60 |
| **Peace** | |
| Always | 1,654 (16%) |
| Often | 6,433 (62%) |
| Rarely | 1,953 (19%) |
| Never | 275 (2.6%) |
| (Missing) | 75 (0.7%) |
| **Balance in Life** | |
| Always | 1,640 (16%) |
| Often | 6,446 (62%) |
| Rarely | 1,962 (19%) |
| Never | 276 (2.7%) |
| (Missing) | 66 (0.6%) |
| **Mastery** | |
| Always | 2,896 (28%) |
| Often | 5,763 (55%) |
| Rarely | 1,367 (13%) |
| Never | 278 (2.7%) |
| (Missing) | 85 (0.8%) |
| **Meaning** | 7.71 (1.76) |
| (Missing) | 56 |
| **Purpose** | 7.77 (1.99) |
| (Missing) | 34 |
| **Self-Rated Mental Health** | 8.07 (1.81) |
| (Missing) | 19 |
| **Content with My Relationships** | 7.75 (1.83) |
| (Missing) | 116 |
| **Satisfying Relationships** | 7.56 (1.88) |
| (Missing) | 115 |
| **Social Support** | 8.00 (1.85) |
| (Missing) | 52 |
| **Intimate Friend** | |
| Yes | 8,861 (85%) |
| No | 1,350 (13%) |
| (Missing) | 178 (1.7%) |
| **Government Approval** | |
| Strongly approve | 1,378 (13%) |
| Somewhat approve | 2,009 (19%) |
| Neither approve nor disapprove | 2,126 (20%) |
| Somewhat disapprove | 1,944 (19%) |
| Strongly disapprove | 2,896 (28%) |

| Characteristic | N = 10,389[1] |
|---|---|
| (Missing) | 37 (0.4%) |
| **Political Voice** | |
| Agree | 2,709 (26%) |
| Disagree | 5,845 (56%) |
| Unsure | 1,780 (17%) |
| (Missing) | 55 (0.5%) |
| **Belonging** | 7.72 (2.17) |
| (Missing) | 41 |
| **City Satisfaction** | |
| Satisfied | 9,114 (88%) |
| Dissatisfied | 651 (6.3%) |
| Unsure | 614 (5.9%) |
| (Missing) | 11 (0.1%) |
| **Trust** | |
| All | 84 (0.8%) |
| Most | 2,259 (22%) |
| Some | 4,276 (41%) |
| Not very many | 3,310 (32%) |
| None | 346 (3.3%) |
| (Missing) | 113 (1.1%) |
| **Community Participation** | |
| More than once a week | 270 (2.6%) |
| Once a week | 771 (7.4%) |
| One to three times a month | 1,155 (11%) |
| A few times a year | 3,017 (29%) |
| Never | 5,113 (49%) |
| (Missing) | 62 (0.6%) |
| **Traumatic Distress** | |
| A lot | 326 (3.1%) |
| Some | 1,348 (13%) |
| Not very much | 3,052 (29%) |
| None at all | 5,614 (54%) |
| (Missing) | 49 (0.5%) |
| **Suffering** | |
| A lot | 236 (2.3%) |
| Some | 2,299 (22%) |
| Not very much | 3,744 (36%) |
| None at all | 4,067 (39%) |
| (Missing) | 43 (0.4%) |
| **Loneliness** | 2.44 (2.25) |
| (Missing) | 59 |
| **Discrimination** | |
| Always | 205 (2.0%) |
| Often | 1,138 (11%) |
| Rarely | 2,738 (26%) |
| Never | 6,288 (61%) |
| (Missing) | 21 (0.2%) |
| **Promoting Good** | 7.92 (1.64) |
| (Missing) | 47 |
| **Delayed Gratification** | 6.80 (2.31) |
| (Missing) | 47 |
| **Hope** | 7.94 (1.78) |
| (Missing) | 47 |
| **Gratitude** | 7.35 (1.81) |
| (Missing) | 107 |
| **Love** | 7.80 (1.98) |
| (Missing) | 35 |
| **Forgiveness** | |
| Always | 1,713 (16%) |
| Often | 5,014 (48%) |
| Rarely | 2,789 (27%) |
| Never | 810 (7.8%) |
| (Missing) | 64 (0.6%) |
| **Charitable Giving** | |
| Yes | 2,112 (20%) |
| No | 8,218 (79%) |
| (Missing) | 59 (0.6%) |
| **Helping** | |
| Yes | 2,628 (25%) |

| Characteristic | N = 10,389[1] |
|---|---|
| No | 7,550 (73%) |
| (Missing) | 211 (2.0%) |
| **Volunteering** | |
| Yes | 856 (8.2%) |
| No | 9,447 (91%) |
| (Missing) | 86 (0.8%) |
| **Self-Rated Physical Health** | 7.68 (1.90) |
| (Missing) | 24 |
| **Health Limitations** | |
| Yes | 1,381 (13%) |
| No | 8,938 (86%) |
| (Missing) | 70 (0.7%) |
| **Pain** | |
| A lot | 533 (5.1%) |
| Some | 2,834 (27%) |
| Not very much | 3,176 (31%) |
| None at all | 3,822 (37%) |
| (Missing) | 24 (0.2%) |
| **Smoking** | 4 (7) |
| (Missing) | 102 |
| **Drinking** | 2.01 (3.55) |
| (Missing) | 130 |
| **Exercise** | |
| 0 days | 5,469 (53%) |
| 1 day | 867 (8.3%) |
| 2 days | 1,398 (13%) |
| 3 days | 1,193 (11%) |
| 4 days | 549 (5.3%) |
| 5 days | 342 (3.3%) |
| 6 days | 110 (1.1%) |
| 7 days/Every day | 356 (3.4%) |
| (Missing) | 106 (1.0%) |
| **Financial Stability** | 6.98 (2.37) |
| (Missing) | 32 |
| **Material Stability** | 7.33 (2.38) |
| (Missing) | 80 |
| **Education** | |
| Up to 8 years | 1,238 (12%) |
| 9-15 years | 6,130 (59%) |
| 16+ years | 3,020 (29%) |
| (Missing) | 1 (<0.1%) |
| **Employment** | |
| Employed for an employer | 5,837 (56%) |
| Self-employed | 686 (6.6%) |
| Retired | 2,434 (23%) |
| Student | 515 (5.0%) |
| Homemaker | 338 (3.3%) |
| Unemployed and looking for a job | 284 (2.7%) |
| None of these/Other | 169 (1.6%) |
| (Missing) | 126 (1.2%) |
| **Subjective Financial Well-Being** | |
| Living comfortably on present income | 1,768 (17%) |
| Getting by on present income | 6,328 (61%) |
| Finding it difficult on present income | 1,789 (17%) |
| Finding it very difficult on present income | 381 (3.7%) |
| (Missing) | 123 (1.2%) |
| **Housing** | |
| Someone in this household OWNS this home | 7,517 (72%) |
| Someone in this household RENTS this home | 1,309 (13%) |
| Both | 259 (2.5%) |
| Neither | 1,220 (12%) |
| Rent | 0 (0%) |
| Own | 0 (0%) |
| Something else | 0 (0%) |
| (Missing) | 83 (0.8%) |
| **Self-Reported Religion/Spirituality** | |
| Always | 1,999 (19%) |
| Often | 3,756 (36%) |
| Rarely | 2,994 (29%) |

| Characteristic | N = 10,389[1] |
|---|---|
| Never | 1,571 (15%) |
| (Missing) | 69 (0.7%) |
| **Religious Service Attendance** | |
| More than once a week | 305 (2.9%) |
| Once a week | 3,263 (31%) |
| One to three times a month | 2,081 (20%) |
| A few times a year | 3,064 (29%) |
| Never | 1,597 (15%) |
| (Missing) | 78 (0.8%) |
| **Life after Death Belief** | |
| Yes | 5,920 (57%) |
| No | 2,205 (21%) |
| Unsure | 2,202 (21%) |
| (Missing) | 63 (0.6%) |
| **Religious Experience** | |
| Yes | 875 (8.4%) |
| No | 9,476 (91%) |
| (Missing) | 38 (0.4%) |
| **Religious Reading** | |
| More than once a day | 102 (1.0%) |
| About once a day | 734 (7.1%) |
| Sometimes | 5,124 (49%) |
| Never | 4,347 (42%) |
| (Missing) | 83 (0.8%) |
| **Prayer-Meditation** | |
| More than once a day | 710 (6.8%) |
| About once a day | 2,225 (21%) |
| Sometimes | 4,715 (45%) |
| Never | 2,682 (26%) |
| (Missing) | 57 (0.5%) |
| **Belief in God** | |
| One God | 7,972 (77%) |
| More than one god | 103 (1.0%) |
| An impersonal spiritual force | 454 (4.4%) |
| None of these | 885 (8.5%) |
| Unsure | 908 (8.7%) |
| (Missing) | 66 (0.6%) |
| **Intrinsic Religiosity** | |
| Agree | 4,497 (43%) |
| Disagree | 2,491 (24%) |
| Not relevant | 1,193 (11%) |
| Unsure | 2,094 (20%) |
| (Missing) | 114 (1.1%) |
| **Religious Comfort** | |
| Agree | 4,918 (47%) |
| Disagree | 2,059 (20%) |
| Not relevant | 1,147 (11%) |
| Unsure | 2,149 (21%) |
| (Missing) | 117 (1.1%) |
| **Loved by God** | |
| Agree | 4,930 (47%) |
| Disagree | 1,634 (16%) |
| Not relevant | 1,145 (11%) |
| Unsure | 2,535 (24%) |
| (Missing) | 145 (1.4%) |
| **Spiritual Punishment** | |
| Agree | 1,773 (17%) |
| Disagree | 4,666 (45%) |
| Not relevant | 1,223 (12%) |
| Unsure | 2,574 (25%) |
| (Missing) | 154 (1.5%) |
| **Religious Criticism** | |
| Agree | 1,307 (13%) |
| Disagree | 4,980 (48%) |
| Not relevant | 1,964 (19%) |
| Unsure | 2,001 (19%) |
| (Missing) | 136 (1.3%) |
| **Evangelism** | |
| Agree | 3,090 (30%) |

| Characteristic | N = 10,389[1] |
|---|---|
| Disagree | 3,795 (37%) |
| Not relevant | 1,609 (15%) |
| Unsure | 1,804 (17%) |
| (Missing) | 92 (0.9%) |
| **Children** | |
| 0 | 7,002 (68%) |
| 1 | 1,555 (15%) |
| 2 | 1,322 (13%) |
| 3 | 304 (3.0%) |
| 4 | 20 (0.2%) |
| 5 | 13 (0.1%) |
| 6 | 17 (0.2%) |
| 7 | 1 (<0.1%) |
| 11 | 1 (<0.1%) |
| (Missing) | 154 |

[1]Mean (SD); n (%)

**Table S15c. Demographic variation across outcomes for Poland**

| Outcome | Male | Female | Other | Male vs Female p-value | Global p-value |
|---|---|---|---|---|---|
| *Flourishing Index and Domains* | | | | | |
| Flourishing Index | 7.62 (7.51,7.72) | 7.65 (7.56,7.74) | 5.88 * | 9.56e-01 | 8.4e-02 |
| Secure Flourishing Index | 7.56 (7.46,7.67) | 7.54 (7.45,7.63) | 5.76 * | 4.07e-01 | 8.61e-02 |
| Happiness & Life Satisfaction | 7.56 (7.45,7.67) | 7.52 (7.42,7.62) | 5.95 * | 4.35e-01 | 3.64e-01 |
| Social Relationship Quality | 7.64 (7.52,7.77) | 7.67 (7.56,7.77) | 5.04 * | 9.73e-01 | 1.6e-03 |
| Meaning and Purpose | 7.68 (7.55,7.82) | 7.80 (7.68,7.91) | 6.07 * | 1.02e-01 | 8.31e-03 |
| Character & Virtue | 7.24 (7.13,7.36) | 7.47 (7.36,7.57) | 5.79 * | 4.49e-02 | 2.2e-06 |
| Self-Rated Health | 7.95 (7.83,8.08) | 7.80 (7.69,7.91) | 6.55 * | 1.55e-02 | 1.12e-02 |
| Financial and Material Worry | 7.31 (7.16,7.46) | 7.01 (6.85,7.17) | 5.18 * | 9.02e-04 | 7.76e-06 |
| *Psychological Well-Being* | | | | | |
| Happiness | 7.57 (7.46,7.68) | 7.54 (7.44,7.64) | 5.89 * | 5.09e-01 | 3.97e-01 |
| Life Satisfaction | 7.55 (7.43,7.66) | 7.50 (7.39,7.62) | 6.02 * | 4.28e-01 | 3.47e-01 |
| Present Life Evaluation | 7.14 (7.04,7.24) | 7.10 (7.00,7.20) | 6.18 * | 3.14e-01 | 5.67e-01 |
| Future Life Evaluation | 7.45 (7.34,7.56) | 7.34 (7.23,7.45) | 7.06 * | 3.1e-02 | 1.86e-01 |
| Optimism | 7.66 (7.54,7.79) | 7.77 (7.66,7.89) | 5.76 * | 7.57e-01 | 6.52e-02 |
| Freedom | 7.58 (7.44,7.72) | 7.62 (7.50,7.74) | 5.84 * | 4.62e-01 | 1.72e-02 |
| Peace | 0.79 (0.76,0.81) | 0.78 (0.76,0.81) | 0.44 * | 9.66e-01 | 5.35e-01 |
| Balance in Life | 0.78 (0.75,0.81) | 0.78 (0.76,0.81) | 0.43 * | 7.99e-01 | 8.61e-02 |
| Mastery | 0.84 (0.81,0.87) | 0.84 (0.82,0.87) | 0.43 * | 5.88e-01 | 3.76e-02 |
| Meaning | 7.65 (7.52,7.79) | 7.76 (7.65,7.87) | 6.01 * | 3.54e-01 | 2.96e-02 |
| Purpose | 7.71 (7.55,7.86) | 7.83 (7.69,7.97) | 6.13 * | 3.19e-02 | 6.85e-03 |
| Self-Rated Mental Health | 8.12 (7.99,8.25) | 8.02 (7.90,8.14) | 6.22 * | 1.14e-01 | 6.35e-02 |
| *Social Well-Being* | | | | | |
| Content with My Relationships | 7.75 (7.63,7.88) | 7.75 (7.63,7.86) | 4.14 * | 6.35e-01 | 7.07e-03 |
| Satisfying Relationships | 7.53 (7.40,7.66) | 7.58 (7.47,7.70) | 5.93 * | 6.06e-01 | 4.42e-03 |
| Social Support | 7.93 (7.79,8.07) | 8.08 (7.96,8.19) | 6.81 * | 6.87e-01 | 1.92e-02 |
| Intimate Friend | 0.86 (0.84,0.88) | 0.88 (0.86,0.90) | 0.57 * | 5.13e-01 | 5.31e-02 |
| Government Approval | 0.31 (0.28,0.34) | 0.34 (0.31,0.37) | 0.13 * | 3.96e-02 | 2.63e-02 |
| Political Voice | 0.31 (0.28,0.34) | 0.33 (0.30,0.35) | 0.69 * | 8.32e-01 | 1.46e-01 |
| Belonging | 7.68 (7.52,7.83) | 7.77 (7.62,7.92) | 5.88 * | 8.86e-01 | 5.88e-02 |
| City Satisfaction | 0.92 (0.90,0.93) | 0.94 (0.93,0.96) | 0.69 * | 1.73e-02 | 2.71e-03 |
| Trust | 0.21 (0.19,0.24) | 0.24 (0.22,0.27) | 0.13 * | 5.58e-02 | 4.36e-02 |
| Community Participation | 0.10 (0.08,0.12) | 0.10 (0.08,0.12) | 0.00 * | 4.55e-01 | 1.6e-16 |
| *Psychological Distress* | | | | | |
| Traumatic Distress | 0.15 (0.13,0.17) | 0.18 (0.15,0.20) | 0.30 * | 9.73e-02 | 4.84e-02 |
| Depression Symptoms | 0.13 (0.11,0.15) | 0.15 (0.13,0.17) | 0.69 * | 2.13e-03 | 2.74e-02 |
| Anxiety Symptoms | 0.12 (0.10,0.14) | 0.13 (0.11,0.15) | 0.30 * | 2.25e-02 | 1.63e-01 |
| Suffering | 0.22 (0.20,0.24) | 0.27 (0.24,0.29) | 0.13 * | 1.18e-05 | 6.35e-04 |
| *Social Distress* | | | | | |
| Loneliness | 2.38 (2.23,2.53) | 2.50 (2.37,2.62) | 4.83 * | 1.77e-01 | 1.29e-02 |
| Discrimination | 0.12 (0.10,0.14) | 0.14 (0.12,0.16) | 0.43 * | 3.36e-01 | 7.32e-02 |
| *Character & Prosocial Behavior* | | | | | |
| Promoting Good | 7.78 (7.65,7.91) | 8.04 (7.94,8.15) | 6.31 * | 1.83e-02 | 2.35e-08 |
| Delayed Gratification | 6.70 (6.54,6.87) | 6.89 (6.73,7.05) | 5.28 * | 2.71e-01 | 3.57e-03 |
| Hope | 7.87 (7.73,8.01) | 8.00 (7.89,8.12) | 6.32 * | 1.87e-01 | 2.39e-02 |
| Gratitude | 7.22 (7.09,7.34) | 7.48 (7.37,7.58) | 6.03 * | 3.11e-04 | 1.3e-06 |
| Love | 7.58 (7.43,7.73) | 8.01 (7.89,8.13) | 5.34 * | 2.82e-05 | 5.06e-11 |
| Forgiveness | 0.60 (0.56,0.63) | 0.70 (0.67,0.73) | 0.43 * | 7.19e-06 | 8.36e-12 |
| Charitable Giving | 0.19 (0.16,0.21) | 0.22 (0.20,0.24) | 0.00 * | 3.52e-01 | 1.6e-16 |
| Helping | 0.25 (0.22,0.27) | 0.27 (0.25,0.30) | 0.00 * | 1.77e-01 | 1.6e-16 |
| Volunteering | 0.07 (0.06,0.09) | 0.10 (0.08,0.11) | 0.00 * | 6.3e-02 | 1.6e-16 |
| *Physical Health & Health Behavior* | | | | | |
| Self-Rated Physical Health | 7.79 (7.65,7.92) | 7.58 (7.46,7.70) | 6.88 * | 7.98e-03 | 5.21e-03 |
| Health Limitations | 0.13 (0.11,0.14) | 0.14 (0.12,0.16) | 0.00 * | 1.02e-01 | 1.6e-16 |
| Pain | 0.29 (0.27,0.32) | 0.35 (0.33,0.38) | 0.43 * | 4.19e-03 | 4.26e-04 |
| Smoking | 5.18 (4.78,5.58) | 2.23 (1.97,2.48) | 6.14 * | 1.6e-16 | 1.6e-16 |
| Drinking | 2.87 (2.62,3.13) | 1.18 (1.07,1.29) | 1.87 * | 1.6e-16 | 1.6e-16 |
| Exercise | 1.50 (1.38,1.63) | 1.29 (1.20,1.39) | 1.18 * | 2.76e-03 | 6.52e-04 |
| *Socioeconomic Outcomes* | | | | | |
| Financial Stability | 7.16 (7.01,7.31) | 6.81 (6.65,6.98) | 4.84 * | 5.99e-04 | 2.23e-06 |
| Material Stability | 7.45 (7.30,7.61) | 7.21 (7.05,7.38) | 5.53 * | 3.15e-03 | 4.52e-05 |
| Education | 0.26 (0.24,0.28) | 0.32 (0.30,0.34) | 0.31 * | 2.63e-04 | 3.16e-06 |
| Employment | 0.71 (0.68,0.73) | 0.57 (0.54,0.60) | 0.60 * | 2.22e-12 | 1.6e-16 |
| Subjective Financial Well-Being | 0.82 (0.79,0.84) | 0.76 (0.73,0.79) | 1.00 * | 7.42e-05 | 1.6e-16 |
| Housing | 0.76 (0.73,0.79) | 0.75 (0.73,0.78) | 0.56 * | 9.19e-01 | 6.31e-01 |
| *Religion/Spirituality* | | | | | |
| Self-Reported Religion/Spirituality | 0.50 (0.47,0.53) | 0.61 (0.59,0.64) | 0.73 * | 5.74e-09 | 2.17e-12 |
| Religious Service Attendance | 0.28 (0.25,0.31) | 0.41 (0.38,0.43) | 0.13 * | 6.64e-14 | <2e-16 |

| Outcome | Male | Female | Other | Male vs Female p-value | Global p-value |
|---|---|---|---|---|---|
| Life after Death Belief | 0.52 (0.48,0.55) | 0.63 (0.60,0.65) | 0.44 * | 1.09e-09 | 1.13e-13 |
| Religious Experience | 0.06 (0.05,0.08) | 0.10 (0.09,0.12) | 0.00 * | 4.03e-03 | 1.6e-16 |
| Religious Reading | 0.06 (0.04,0.07) | 0.10 (0.09,0.12) | 0.00 * | 2.35e-06 | 1.6e-16 |
| Prayer-Meditation | 0.21 (0.18,0.24) | 0.35 (0.32,0.38) | 0.31 * | 1.6e-16 | 1.6e-16 |
| Belief in God | 0.78 (0.76,0.81) | 0.86 (0.85,0.88) | 0.57 * | 1.04e-08 | 1.26e-11 |
| Intrinsic Religiosity | 0.51 (0.48,0.54) | 0.63 (0.60,0.65) | 0.51 * | 1.16e-09 | <2e-16 |
| Religious Comfort | 0.54 (0.51,0.57) | 0.68 (0.66,0.70) | 0.54 * | <2e-16 | 1.6e-16 |
| Loved by God | 0.58 (0.56,0.61) | 0.70 (0.67,0.73) | 0.16 * | 1.52e-10 | <2e-16 |
| Spiritual Punishment | 0.24 (0.20,0.27) | 0.26 (0.24,0.29) | 0.41 * | 7e-02 | 5.44e-02 |
| Religious Criticism | 0.21 (0.18,0.23) | 0.21 (0.18,0.23) | 0.44 * | 4.21e-01 | 4.94e-01 |
| Evangelism | 0.40 (0.37,0.44) | 0.46 (0.42,0.49) | 0.24 * | 4.28e-04 | 8.29e-05 |
| *Family Factors* | | | | | |
| Ever Married | 0.75 (0.73,0.77) | 0.79 (0.77,0.81) | 0.13 * | 4.1e-03 | 3.51e-07 |
| Divorced | 0.04 (0.03,0.05) | 0.06 (0.05,0.07) | 0.00 * | 6.92e-02 | 1.6e-16 |
| Children | 0.53 (0.48,0.58) | 0.52 (0.48,0.56) | 0.00 * | 5.08e-01 | 1.6e-16 |

**Table S16a. Nationally representative descriptive statistics for South Africa**

| Characteristic | N = 2,651[1] |
|---|---|
| **Age group** | |
| 18-24 | 461 (17%) |
| 25-29 | 364 (14%) |
| 30-39 | 655 (25%) |
| 40-49 | 522 (20%) |
| 50-59 | 309 (12%) |
| 60-69 | 195 (7.4%) |
| 70-79 | 120 (4.5%) |
| 80 or older | 17 (0.6%) |
| (Missing) | 9 (0.3%) |
| **Gender** | |
| Male | 1,288 (49%) |
| Female | 1,356 (51%) |
| Other | 2 (<0.1%) |
| (Missing) | 4 (0.2%) |
| **Marital status** | |
| Married | 539 (20%) |
| Separated | 76 (2.9%) |
| Divorced | 51 (1.9%) |
| Widowed | 133 (5.0%) |
| Single, never married | 1,561 (59%) |
| Domestic Partner | 264 (10.0%) |
| (Missing) | 28 (1.0%) |
| **Employment** | |
| Employed for an employer | 569 (21%) |
| Self-employed | 412 (16%) |
| Retired | 243 (9.2%) |
| Student | 204 (7.7%) |
| Homemaker | 137 (5.2%) |
| Unemployed and looking for a job | 1,008 (38%) |
| None of these/Other | 74 (2.8%) |
| (Missing) | 3 (0.1%) |
| **Religious service attendance** | |
| More than 1/week | 414 (16%) |
| 1/week | 891 (34%) |
| 1-3/month | 574 (22%) |
| A few times a year | 431 (16%) |
| Never | 334 (13%) |
| (Missing) | 7 (0.3%) |
| **Education** | |
| Up to 8 years | 668 (25%) |
| 9-15 years | 1,796 (68%) |
| 16+ years | 183 (6.9%) |
| (Missing) | 4 (0.2%) |
| **Immigration** | |
| Born in this country | 2,511 (95%) |
| Born in another country | 139 (5.2%) |
| (Missing) | 1 (<0.1%) |
| **Religious affiliation** | |
| Christianity | 2,163 (82%) |
| Islam | 62 (2.3%) |
| Hinduism | 1 (<0.1%) |
| Buddhism | 12 (0.5%) |
| Judaism | 0 (0%) |
| Sikhism | 0 (0%) |
| Baha'i | 0 (0%) |
| Jainism | 2 (<0.1%) |
| Shinto | 2 (<0.1%) |
| Taoism | 1 (<0.1%) |
| Confucianism | 0 (0%) |
| Primal, Animist, or Folk religion | 127 (4.8%) |
| Spiritism | 0 (0%) |
| Umbanda, Candomble, and other African-derived religions | 0 (0%) |
| Chinese folk/traditional religion | 0 (0%) |
| Some other religion | 5 (0.2%) |
| No religion/Atheist/Agnostic | 253 (9.6%) |
| (Missing) | 23 (0.9%) |
| **Race/Ethnicity** | |

| Characteristic | N = 2,651[1] |
|---|---|
| (Missing) | 3 (0.1%) |
| Asian/Indian | 6 (0.2%) |
| Black | 2,381 (90%) |
| Colored | 252 (9.5%) |
| Other | 1 (<0.1%) |
| White | 8 (0.3%) |

[1]n (%)

**Table S16b. Descriptive statistics of outcome variables for South Africa**

| Characteristic | N = 2,651[1] |
|---|---|
| **Flourishing Index** | 7.42 (1.58) |
| (Missing) | 51 |
| **Secure Flourishing Index** | 7.07 (1.55) |
| (Missing) | 61 |
| **Happiness & Life Satisfaction** | 6.66 (2.38) |
| (Missing) | 10 |
| **Social Relationship Quality** | 7.40 (2.30) |
| (Missing) | 9 |
| **Meaning and Purpose** | 7.39 (2.08) |
| (Missing) | 16 |
| **Character & Virtue** | 7.75 (2.02) |
| (Missing) | 13 |
| **Self-Rated Health** | 7.86 (2.22) |
| (Missing) | 11 |
| **Financial and Material Worry** | 5.4 (3.0) |
| (Missing) | 13 |
| **Happiness** | 6.95 (2.65) |
| (Missing) | 1 |
| **Life Satisfaction** | 6.4 (2.8) |
| (Missing) | 10 |
| **Present Life Evaluation** | 6.11 (2.84) |
| (Missing) | 6 |
| **Future Life Evaluation** | 7.62 (2.45) |
| (Missing) | 68 |
| **Optimism** | 8.22 (2.22) |
| (Missing) | 9 |
| **Freedom** | 7.97 (2.39) |
| (Missing) | 5 |
| **Peace** | |
| Always | 1,158 (44%) |
| Often | 953 (36%) |
| Rarely | 340 (13%) |
| Never | 181 (6.8%) |
| (Missing) | 19 (0.7%) |
| **Balance in Life** | |
| Always | 841 (32%) |
| Often | 1,014 (38%) |
| Rarely | 493 (19%) |
| Never | 287 (11%) |
| (Missing) | 16 (0.6%) |
| **Mastery** | |
| Always | 1,434 (54%) |
| Often | 797 (30%) |
| Rarely | 283 (11%) |
| Never | 128 (4.8%) |
| (Missing) | 10 (0.4%) |
| **Meaning** | 6.72 (2.77) |
| (Missing) | 12 |
| **Purpose** | 8.07 (2.40) |
| (Missing) | 4 |
| **Self-Rated Mental Health** | 8.18 (2.46) |
| (Missing) | 9 |
| **Content with My Relationships** | 7.61 (2.51) |
| (Missing) | 6 |
| **Satisfying Relationships** | 7.18 (2.70) |
| (Missing) | 5 |
| **Social Support** | 7.19 (2.82) |
| (Missing) | 7 |
| **Intimate Friend** | |
| Yes | 2,206 (83%) |
| No | 444 (17%) |
| (Missing) | 2 (<0.1%) |
| **Government Approval** | |
| Strongly approve | 572 (22%) |
| Somewhat approve | 430 (16%) |
| Neither approve nor disapprove | 578 (22%) |
| Somewhat disapprove | 295 (11%) |
| Strongly disapprove | 746 (28%) |

| Characteristic | N = 2,651[1] |
|---|---|
| (Missing) | 30 (1.1%) |
| **Political Voice** | |
| Agree | 1,321 (50%) |
| Disagree | 819 (31%) |
| Unsure | 502 (19%) |
| (Missing) | 8 (0.3%) |
| **Belonging** | 7.65 (2.71) |
| (Missing) | 17 |
| **City Satisfaction** | |
| Satisfied | 1,463 (55%) |
| Dissatisfied | 879 (33%) |
| Unsure | 307 (12%) |
| (Missing) | 2 (<0.1%) |
| **Trust** | |
| All | 85 (3.2%) |
| Most | 196 (7.4%) |
| Some | 676 (26%) |
| Not very many | 910 (34%) |
| None | 743 (28%) |
| (Missing) | 41 (1.6%) |
| **Community Participation** | |
| More than once a week | 265 (10.0%) |
| Once a week | 377 (14%) |
| One to three times a month | 615 (23%) |
| A few times a year | 537 (20%) |
| Never | 847 (32%) |
| (Missing) | 10 (0.4%) |
| **Traumatic Distress** | |
| A lot | 432 (16%) |
| Some | 465 (18%) |
| Not very much | 653 (25%) |
| None at all | 1,015 (38%) |
| (Missing) | 86 (3.2%) |
| **Suffering** | |
| A lot | 314 (12%) |
| Some | 474 (18%) |
| Not very much | 706 (27%) |
| None at all | 1,139 (43%) |
| (Missing) | 19 (0.7%) |
| **Loneliness** | 3.7 (3.0) |
| (Missing) | 2 |
| **Discrimination** | |
| Always | 424 (16%) |
| Often | 548 (21%) |
| Rarely | 501 (19%) |
| Never | 1,127 (43%) |
| (Missing) | 50 (1.9%) |
| **Promoting Good** | 7.82 (2.36) |
| (Missing) | 3 |
| **Delayed Gratification** | 7.68 (2.44) |
| (Missing) | 10 |
| **Hope** | 8.59 (2.07) |
| (Missing) | 5 |
| **Gratitude** | 7.85 (2.42) |
| (Missing) | 8 |
| **Love** | 8.59 (2.02) |
| (Missing) | 6 |
| **Forgiveness** | |
| Always | 1,600 (60%) |
| Often | 631 (24%) |
| Rarely | 257 (9.7%) |
| Never | 162 (6.1%) |
| (Missing) | 1 (<0.1%) |
| **Charitable Giving** | |
| Yes | 513 (19%) |
| No | 2,137 (81%) |
| (Missing) | 1 (<0.1%) |
| **Helping** | |
| Yes | 1,530 (58%) |

| Characteristic | N = 2,651[1] |
|---|---|
| No | 1,109 (42%) |
| (Missing) | 12 (0.4%) |
| **Volunteering** | |
| Yes | 656 (25%) |
| No | 1,995 (75%) |
| (Missing) | 0 (0%) |
| **Self-Rated Physical Health** | 7.52 (2.72) |
| (Missing) | 3 |
| **Health Limitations** | |
| Yes | 575 (22%) |
| No | 2,045 (77%) |
| (Missing) | 30 (1.1%) |
| **Pain** | |
| A lot | 287 (11%) |
| Some | 468 (18%) |
| Not very much | 762 (29%) |
| None at all | 1,125 (42%) |
| (Missing) | 8 (0.3%) |
| **Smoking** | 1.69 (4.60) |
| (Missing) | 44 |
| **Drinking** | 2.4 (6.0) |
| (Missing) | 49 |
| **Exercise** | |
| 0 days | 1,022 (39%) |
| 1 day | 189 (7.1%) |
| 2 days | 296 (11%) |
| 3 days | 362 (14%) |
| 4 days | 174 (6.5%) |
| 5 days | 214 (8.1%) |
| 6 days | 29 (1.1%) |
| 7 days/Every day | 328 (12%) |
| (Missing) | 36 (1.4%) |
| **Financial Stability** | 5.2 (3.3) |
| (Missing) | 9 |
| **Material Stability** | 5.5 (3.4) |
| (Missing) | 5 |
| **Education** | |
| Up to 8 years | 668 (25%) |
| 9-15 years | 1,796 (68%) |
| 16+ years | 183 (6.9%) |
| (Missing) | 4 (0.2%) |
| **Employment** | |
| Employed for an employer | 569 (21%) |
| Self-employed | 412 (16%) |
| Retired | 243 (9.2%) |
| Student | 204 (7.7%) |
| Homemaker | 137 (5.2%) |
| Unemployed and looking for a job | 1,008 (38%) |
| None of these/Other | 74 (2.8%) |
| (Missing) | 3 (0.1%) |
| **Subjective Financial Well-Being** | |
| Living comfortably on present income | 336 (13%) |
| Getting by on present income | 935 (35%) |
| Finding it difficult on present income | 855 (32%) |
| Finding it very difficult on present income | 459 (17%) |
| (Missing) | 66 (2.5%) |
| **Housing** | |
| Someone in this household OWNS this home | 1,700 (64%) |
| Someone in this household RENTS this home | 383 (14%) |
| Both | 163 (6.1%) |
| Neither | 358 (13%) |
| Rent | 0 (0%) |
| Own | 0 (0%) |
| Something else | 0 (0%) |
| (Missing) | 48 (1.8%) |
| **Self-Reported Religion/Spirituality** | |
| Always | 1,657 (63%) |
| Often | 575 (22%) |
| Rarely | 246 (9.3%) |

| Characteristic | N = 2,651[1] |
|---|---|
| Never | 154 (5.8%) |
| (Missing) | 19 (0.7%) |
| **Religious Service Attendance** | |
| More than once a week | 414 (16%) |
| Once a week | 891 (34%) |
| One to three times a month | 574 (22%) |
| A few times a year | 431 (16%) |
| Never | 334 (13%) |
| (Missing) | 7 (0.3%) |
| **Life after Death Belief** | |
| Yes | 1,389 (52%) |
| No | 765 (29%) |
| Unsure | 482 (18%) |
| (Missing) | 16 (0.6%) |
| **Religious Experience** | |
| Yes | 1,552 (59%) |
| No | 1,025 (39%) |
| (Missing) | 73 (2.8%) |
| **Religious Reading** | |
| More than once a day | 449 (17%) |
| About once a day | 472 (18%) |
| Sometimes | 1,392 (52%) |
| Never | 329 (12%) |
| (Missing) | 10 (0.4%) |
| **Prayer-Meditation** | |
| More than once a day | 1,157 (44%) |
| About once a day | 727 (27%) |
| Sometimes | 683 (26%) |
| Never | 80 (3.0%) |
| (Missing) | 4 (0.1%) |
| **Belief in God** | |
| One God | 2,129 (80%) |
| More than one god | 267 (10%) |
| An impersonal spiritual force | 135 (5.1%) |
| None of these | 61 (2.3%) |
| Unsure | 54 (2.0%) |
| (Missing) | 5 (0.2%) |
| **Intrinsic Religiosity** | |
| Agree | 2,072 (78%) |
| Disagree | 254 (9.6%) |
| Not relevant | 134 (5.0%) |
| Unsure | 185 (7.0%) |
| (Missing) | 6 (0.2%) |
| **Religious Comfort** | |
| Agree | 2,312 (87%) |
| Disagree | 158 (5.9%) |
| Not relevant | 101 (3.8%) |
| Unsure | 73 (2.8%) |
| (Missing) | 8 (0.3%) |
| **Loved by God** | |
| Agree | 2,420 (91%) |
| Disagree | 89 (3.4%) |
| Not relevant | 76 (2.9%) |
| Unsure | 58 (2.2%) |
| (Missing) | 8 (0.3%) |
| **Spiritual Punishment** | |
| Agree | 1,086 (41%) |
| Disagree | 1,155 (44%) |
| Not relevant | 216 (8.2%) |
| Unsure | 184 (7.0%) |
| (Missing) | 9 (0.3%) |
| **Religious Criticism** | |
| Agree | 1,032 (39%) |
| Disagree | 901 (34%) |
| Not relevant | 335 (13%) |
| Unsure | 376 (14%) |
| (Missing) | 8 (0.3%) |
| **Evangelism** | |
| Agree | 1,904 (72%) |

| **Characteristic** | **N = 2,651**[1] |
|---|---|
| Disagree | 467 (18%) |
| Not relevant | 171 (6.5%) |
| Unsure | 96 (3.6%) |
| (Missing) | 12 (0.4%) |
| **Children** | 1.91 (1.94) |
| (Missing) | 3 |

[1]Mean (SD); n (%)

**Table S16c. Demographic variation across outcomes for South Africa**

| Outcome | Male | Female | Other | Male vs Female p-value | Global p-value |
|---|---|---|---|---|---|
| *Flourishing Index and Domains* | | | | | |
| Flourishing Index | 7.46 (7.34,7.57) | 7.37 (7.24,7.49) | 7.15 * | 6.42e-01 | 4.4e-01 |
| Secure Flourishing Index | 7.13 (7.02,7.24) | 7.02 (6.90,7.13) | 7.07 * | 3.59e-01 | 3.03e-01 |
| Happiness & Life Satisfaction | 6.67 (6.48,6.85) | 6.65 (6.47,6.83) | 6.30 * | 6.34e-01 | 7.1e-01 |
| Social Relationship Quality | 7.44 (7.27,7.61) | 7.36 (7.21,7.52) | 6.44 * | 7.98e-01 | 4.33e-02 |
| Meaning and Purpose | 7.41 (7.26,7.55) | 7.38 (7.22,7.53) | 7.51 * | 5.01e-01 | 9.34e-01 |
| Character & Virtue | 7.76 (7.60,7.92) | 7.74 (7.58,7.90) | 7.65 * | 6.86e-01 | 9.74e-01 |
| Self-Rated Health | 8.01 (7.83,8.18) | 7.70 (7.53,7.88) | 7.86 * | 8.45e-02 | 4.29e-02 |
| Financial and Material Worry | 5.51 (5.27,5.74) | 5.26 (5.07,5.46) | 6.68 * | 9.82e-02 | 8.41e-05 |
| *Psychological Well-Being* | | | | | |
| Happiness | 6.96 (6.75,7.16) | 6.95 (6.74,7.16) | 6.30 * | 4.99e-01 | 5.86e-01 |
| Life Satisfaction | 6.38 (6.15,6.61) | 6.35 (6.16,6.54) | 6.29 * | 8.67e-01 | 9.43e-01 |
| Present Life Evaluation | 6.02 (5.78,6.25) | 6.20 (5.98,6.41) | 6.58 * | 1.43e-01 | 3.23e-01 |
| Future Life Evaluation | 7.59 (7.40,7.78) | 7.60 (7.41,7.79) | 7.86 * | 6.33e-01 | 9.3e-01 |
| Optimism | 8.30 (8.13,8.47) | 8.14 (7.97,8.31) | 7.28 * | 1.51e-01 | 6.7e-04 |
| Freedom | 8.02 (7.83,8.21) | 7.93 (7.76,8.09) | 6.71 * | 6.44e-01 | 1.34e-05 |
| Peace | 0.80 (0.77,0.83) | 0.80 (0.77,0.83) | 1.00 * | 8.59e-01 | 1.6e-16 |
| Balance in Life | 0.71 (0.67,0.75) | 0.70 (0.67,0.73) | 1.00 * | 5.79e-01 | 1.6e-16 |
| Mastery | 0.86 (0.83,0.88) | 0.83 (0.81,0.86) | 1.00 * | 3.27e-01 | 1.6e-16 |
| Meaning | 6.73 (6.52,6.94) | 6.70 (6.49,6.91) | 8.01 * | 6.15e-01 | 6.99e-03 |
| Purpose | 8.09 (7.91,8.26) | 8.05 (7.90,8.21) | 7.01 * | 5.82e-01 | 5.96e-01 |
| Self-Rated Mental Health | 8.28 (8.10,8.47) | 8.07 (7.87,8.28) | 7.43 * | 6.85e-02 | 1.93e-01 |
| *Social Well-Being* | | | | | |
| Content with My Relationships | 7.63 (7.43,7.82) | 7.60 (7.41,7.79) | 6.58 * | 7.29e-01 | 1.43e-01 |
| Satisfying Relationships | 7.25 (7.07,7.44) | 7.12 (6.95,7.29) | 6.29 * | 9.07e-01 | 2.86e-03 |
| Social Support | 7.11 (6.89,7.32) | 7.26 (7.07,7.45) | 6.01 * | 7.82e-01 | 8.39e-03 |
| Intimate Friend | 0.83 (0.80,0.86) | 0.83 (0.81,0.86) | 1.00 * | 6.55e-01 | 1.6e-16 |
| Government Approval | 0.38 (0.34,0.41) | 0.39 (0.35,0.43) | 0.29 * | 5.44e-01 | 7.8e-01 |
| Political Voice | 0.60 (0.55,0.64) | 0.61 (0.57,0.65) | 1.00 * | 7.22e-01 | 1.6e-16 |
| Belonging | 7.60 (7.38,7.82) | 7.69 (7.52,7.86) | 7.58 * | 8.35e-01 | 7.97e-01 |
| City Satisfaction | 0.60 (0.56,0.64) | 0.64 (0.61,0.68) | 0.58 * | 1.29e-01 | 2.29e-01 |
| Trust | 0.12 (0.10,0.15) | 0.10 (0.08,0.12) | 0.00 * | 2.27e-01 | 1.6e-16 |
| Community Participation | 0.26 (0.23,0.29) | 0.22 (0.19,0.25) | 1.00 * | 8.03e-02 | 1.6e-16 |
| *Psychological Distress* | | | | | |
| Traumatic Distress | 0.34 (0.30,0.38) | 0.36 (0.32,0.39) | 0.00 * | 5.71e-01 | 1.6e-16 |
| Depression Symptoms | 0.31 (0.27,0.34) | 0.34 (0.31,0.37) | 0.72 * | 3.02e-01 | 6.1e-02 |
| Anxiety Symptoms | 0.28 (0.25,0.31) | 0.31 (0.27,0.34) | 0.00 * | 6.75e-01 | 1.6e-16 |
| Suffering | 0.27 (0.23,0.30) | 0.33 (0.30,0.36) | 0.42 * | 7.13e-02 | 2.53e-02 |
| *Social Distress* | | | | | |
| Loneliness | 3.63 (3.39,3.86) | 3.73 (3.53,3.93) | 1.86 * | 1.76e-01 | 1.09e-03 |
| Discrimination | 0.37 (0.33,0.41) | 0.38 (0.34,0.41) | 0.00 * | 8.39e-01 | 1.6e-16 |
| *Character & Prosocial Behavior* | | | | | |
| Promoting Good | 7.84 (7.65,8.02) | 7.80 (7.61,7.99) | 7.29 * | 5.39e-01 | 1.43e-01 |
| Delayed Gratification | 7.68 (7.49,7.86) | 7.68 (7.50,7.86) | 8.01 * | 1.86e-01 | 9.52e-01 |
| Hope | 8.69 (8.53,8.85) | 8.48 (8.33,8.64) | 8.01 * | 1.94e-02 | 5.72e-02 |
| Gratitude | 7.85 (7.66,8.04) | 7.85 (7.68,8.02) | 6.44 * | 7.75e-01 | 2.64e-01 |
| Love | 8.59 (8.43,8.76) | 8.59 (8.44,8.74) | 6.29 * | 3.85e-01 | 4.88e-15 |
| Forgiveness | 0.84 (0.81,0.87) | 0.84 (0.82,0.87) | 1.00 * | 3.55e-01 | 1.6e-16 |
| Charitable Giving | 0.21 (0.18,0.24) | 0.18 (0.15,0.21) | 0.28 * | 4.36e-01 | 3.31e-01 |
| Helping | 0.63 (0.59,0.67) | 0.53 (0.49,0.57) | 0.71 * | 3.88e-03 | 5.12e-05 |
| Volunteering | 0.27 (0.23,0.30) | 0.23 (0.20,0.26) | 0.42 * | 6.32e-01 | 2.34e-01 |
| *Physical Health & Health Behavior* | | | | | |
| Self-Rated Physical Health | 7.73 (7.52,7.93) | 7.33 (7.11,7.56) | 8.29 * | 2.8e-01 | 3.71e-04 |
| Health Limitations | 0.19 (0.16,0.22) | 0.25 (0.21,0.28) | 0.42 * | 5.69e-02 | 3.06e-02 |
| Pain | 0.24 (0.21,0.28) | 0.33 (0.30,0.36) | 0.42 * | 2.68e-03 | 7.58e-04 |
| Smoking | 2.51 (2.12,2.90) | 0.85 (0.47,1.23) | 2.97 * | 3.34e-05 | 1.06e-11 |
| Drinking | 3.24 (2.89,3.58) | 1.44 (1.18,1.70) | 4.81 * | 1.82e-06 | 5.55e-16 |
| Exercise | 2.65 (2.45,2.85) | 2.04 (1.87,2.20) | 3.55 * | 4.29e-04 | 2.86e-05 |
| *Socioeconomic Outcomes* | | | | | |
| Financial Stability | 5.40 (5.13,5.66) | 5.05 (4.84,5.26) | 6.29 * | 5.64e-02 | 5.84e-06 |
| Material Stability | 5.62 (5.37,5.87) | 5.47 (5.26,5.69) | 7.07 * | 2.7e-01 | 5.87e-04 |
| Education | 0.08 (0.06,0.09) | 0.06 (0.05,0.08) | 0.00 * | 8.41e-01 | 1.6e-16 |
| Employment | 0.46 (0.42,0.50) | 0.28 (0.25,0.32) | 0.28 * | 1.48e-10 | 5.1e-10 |
| Subjective Financial Well-Being | 0.50 (0.47,0.54) | 0.49 (0.45,0.52) | 0.29 * | 7.23e-01 | 5.88e-01 |
| Housing | 0.69 (0.65,0.72) | 0.74 (0.71,0.77) | 1.00 * | 2.3e-02 | 1.6e-16 |
| *Religion/Spirituality* | | | | | |
| Self-Reported Religion/Spirituality | 0.80 (0.78,0.83) | 0.89 (0.87,0.91) | 0.58 * | 1.85e-02 | 1.87e-05 |
| Religious Service Attendance | 0.39 (0.35,0.43) | 0.59 (0.56,0.62) | 0.71 * | 4.4e-08 | 2.21e-14 |

| Outcome | Male | Female | Other | Male vs Female p-value | Global p-value |
|---|---|---|---|---|---|
| Life after Death Belief | 0.53 (0.49,0.57) | 0.52 (0.48,0.56) | 0.58 * | 7.83e-01 | 8.94e-01 |
| Religious Experience | 0.55 (0.51,0.59) | 0.64 (0.61,0.68) | 0.42 * | 2e-02 | 1.66e-03 |
| Religious Reading | 0.31 (0.27,0.35) | 0.39 (0.35,0.42) | 0.71 * | 2.42e-02 | 7.08e-03 |
| Prayer-Meditation | 0.65 (0.62,0.69) | 0.77 (0.74,0.79) | 1.00 * | 2.35e-03 | 1.6e-16 |
| Belief in God | 0.94 (0.92,0.96) | 0.97 (0.96,0.98) | 1.00 * | 4.1e-02 | 4.22e-15 |
| Intrinsic Religiosity | 0.86 (0.83,0.89) | 0.90 (0.88,0.92) | 1.00 * | 1.83e-01 | 1.6e-16 |
| Religious Comfort | 0.90 (0.87,0.92) | 0.95 (0.93,0.96) | 1.00 * | 3.51e-02 | 1.6e-16 |
| Loved by God | 0.94 (0.92,0.96) | 0.97 (0.96,0.99) | 1.00 * | 1.51e-02 | 1.57e-13 |
| Spiritual Punishment | 0.46 (0.42,0.51) | 0.49 (0.45,0.53) | 0.63 * | 7.72e-01 | 1.6e-16 |
| Religious Criticism | 0.54 (0.50,0.57) | 0.52 (0.49,0.56) | 0.41 * | 6.24e-01 | 6.38e-01 |
| Evangelism | 0.77 (0.74,0.80) | 0.82 (0.78,0.85) | 1.00 * | 3.36e-01 | 1.6e-16 |
| *Family Factors* | | | | | |
| Ever Married | 0.31 (0.27,0.34) | 0.30 (0.27,0.34) | 0.28 * | 6.67e-01 | 9.87e-01 |
| Divorced | 0.02 (0.01,0.03) | 0.02 (0.01,0.03) | 0.28 * | 2.06e-01 | 5.33e-01 |
| Children | 1.45 (1.31,1.59) | 2.36 (2.20,2.51) | 1.14 * | 3.06e-06 | <2e-16 |

**Table S17a. Nationally representative descriptive statistics for Spain**

| Characteristic | N = 6,290[1] |
|---|---|
| **Age group** | |
| 18-24 | 594 (9.4%) |
| 25-29 | 450 (7.2%) |
| 30-39 | 1,111 (18%) |
| 40-49 | 1,396 (22%) |
| 50-59 | 1,252 (20%) |
| 60-69 | 977 (16%) |
| 70-79 | 467 (7.4%) |
| 80 or older | 43 (0.7%) |
| (Missing) | 0 (0%) |
| **Gender** | |
| Male | 3,142 (50%) |
| Female | 3,119 (50%) |
| Other | 6 (0.1%) |
| (Missing) | 22 (0.4%) |
| **Marital status** | |
| Married | 2,947 (47%) |
| Separated | 237 (3.8%) |
| Divorced | 518 (8.2%) |
| Widowed | 189 (3.0%) |
| Single, never married | 1,742 (28%) |
| Domestic Partner | 589 (9.4%) |
| (Missing) | 67 (1.1%) |
| **Employment** | |
| Employed for an employer | 2,862 (45%) |
| Self-employed | 576 (9.2%) |
| Retired | 1,278 (20%) |
| Student | 448 (7.1%) |
| Homemaker | 345 (5.5%) |
| Unemployed and looking for a job | 646 (10%) |
| None of these/Other | 123 (2.0%) |
| (Missing) | 11 (0.2%) |
| **Religious service attendance** | |
| More than 1/week | 317 (5.0%) |
| 1/week | 662 (11%) |
| 1-3/month | 437 (6.9%) |
| A few times a year | 1,972 (31%) |
| Never | 2,875 (46%) |
| (Missing) | 27 (0.4%) |
| **Education** | |
| Up to 8 years | 802 (13%) |
| 9-15 years | 4,145 (66%) |
| 16+ years | 1,341 (21%) |
| (Missing) | 2 (<0.1%) |
| **Immigration** | |
| Born in this country | 5,479 (87%) |
| Born in another country | 788 (13%) |
| (Missing) | 23 (0.4%) |
| **Religious affiliation** | |
| Christianity | 4,074 (65%) |
| Islam | 135 (2.1%) |
| Hinduism | 7 (0.1%) |
| Buddhism | 36 (0.6%) |
| Judaism | 4 (<0.1%) |
| Sikhism | 3 (<0.1%) |
| Baha'i | 2 (<0.1%) |
| Jainism | 1 (<0.1%) |
| Shinto | 0 (0%) |
| Taoism | 5 (<0.1%) |
| Confucianism | 3 (<0.1%) |
| Primal, Animist, or Folk religion | 7 (0.1%) |
| Spiritism | 0 (0%) |
| Umbanda, Candomble, and other African-derived religions | 0 (0%) |
| Chinese folk/traditional religion | 0 (0%) |
| Some other religion | 27 (0.4%) |
| No religion/Atheist/Agnostic | 1,932 (31%) |
| (Missing) | 55 (0.9%) |

[1]n (%)

**Table S17b. Descriptive statistics of outcome variables for Spain**

| Characteristic | N = 6,290[1] |
|---|---|
| **Flourishing Index** | 7.31 (1.42) |
| (Missing) | 139 |
| **Secure Flourishing Index** | 6.89 (1.39) |
| (Missing) | 149 |
| **Happiness & Life Satisfaction** | 6.86 (1.99) |
| (Missing) | 29 |
| **Social Relationship Quality** | 7.41 (2.04) |
| (Missing) | 37 |
| **Meaning and Purpose** | 7.32 (1.92) |
| (Missing) | 43 |
| **Character & Virtue** | 7.80 (1.53) |
| (Missing) | 24 |
| **Self-Rated Health** | 7.15 (1.88) |
| (Missing) | 27 |
| **Financial and Material Worry** | 4.84 (3.02) |
| (Missing) | 14 |
| **Happiness** | 6.92 (2.00) |
| (Missing) | 16 |
| **Life Satisfaction** | 6.79 (2.18) |
| (Missing) | 16 |
| **Present Life Evaluation** | 6.67 (1.93) |
| (Missing) | 7 |
| **Future Life Evaluation** | 7.61 (1.90) |
| (Missing) | 17 |
| **Optimism** | 8.24 (1.97) |
| (Missing) | 17 |
| **Freedom** | 7.45 (2.28) |
| (Missing) | 3 |
| **Peace** | |
| Always | 777 (12%) |
| Often | 2,698 (43%) |
| Rarely | 2,356 (37%) |
| Never | 437 (6.9%) |
| (Missing) | 22 (0.4%) |
| **Balance in Life** | |
| Always | 975 (16%) |
| Often | 3,850 (61%) |
| Rarely | 1,326 (21%) |
| Never | 131 (2.1%) |
| (Missing) | 8 (0.1%) |
| **Mastery** | |
| Always | 1,510 (24%) |
| Often | 3,858 (61%) |
| Rarely | 798 (13%) |
| Never | 87 (1.4%) |
| (Missing) | 37 (0.6%) |
| **Meaning** | 7.30 (2.17) |
| (Missing) | 15 |
| **Purpose** | 7.34 (2.26) |
| (Missing) | 28 |
| **Self-Rated Mental Health** | 7.46 (2.18) |
| (Missing) | 9 |
| **Content with My Relationships** | 7.72 (2.06) |
| (Missing) | 18 |
| **Satisfying Relationships** | 7.10 (2.33) |
| (Missing) | 22 |
| **Social Support** | 8.08 (2.23) |
| (Missing) | 32 |
| **Intimate Friend** | |
| Yes | 5,450 (87%) |
| No | 814 (13%) |
| (Missing) | 26 (0.4%) |
| **Government Approval** | |
| Strongly approve | 421 (6.7%) |
| Somewhat approve | 1,819 (29%) |
| Neither approve nor disapprove | 1,403 (22%) |
| Somewhat disapprove | 1,472 (23%) |
| Strongly disapprove | 1,140 (18%) |

| Characteristic | N = 6,290[1] |
|---|---|
| (Missing) | 34 (0.5%) |
| **Political Voice** | |
| Agree | 896 (14%) |
| Disagree | 3,818 (61%) |
| Unsure | 1,555 (25%) |
| (Missing) | 21 (0.3%) |
| **Belonging** | 7.50 (2.48) |
| (Missing) | 48 |
| **City Satisfaction** | |
| Satisfied | 5,070 (81%) |
| Dissatisfied | 758 (12%) |
| Unsure | 408 (6.5%) |
| (Missing) | 53 (0.9%) |
| **Trust** | |
| All | 101 (1.6%) |
| Most | 1,002 (16%) |
| Some | 2,740 (44%) |
| Not very many | 2,186 (35%) |
| None | 222 (3.5%) |
| (Missing) | 40 (0.6%) |
| **Community Participation** | |
| More than once a week | 733 (12%) |
| Once a week | 706 (11%) |
| One to three times a month | 630 (10%) |
| A few times a year | 1,593 (25%) |
| Never | 2,612 (42%) |
| (Missing) | 15 (0.2%) |
| **Traumatic Distress** | |
| A lot | 718 (11%) |
| Some | 1,590 (25%) |
| Not very much | 1,788 (28%) |
| None at all | 2,183 (35%) |
| (Missing) | 10 (0.2%) |
| **Suffering** | |
| A lot | 736 (12%) |
| Some | 2,285 (36%) |
| Not very much | 2,155 (34%) |
| None at all | 1,083 (17%) |
| (Missing) | 32 (0.5%) |
| **Loneliness** | 3.32 (2.71) |
| (Missing) | 8 |
| **Discrimination** | |
| Always | 275 (4.4%) |
| Often | 940 (15%) |
| Rarely | 2,227 (35%) |
| Never | 2,841 (45%) |
| (Missing) | 7 (0.1%) |
| **Promoting Good** | 8.10 (1.69) |
| (Missing) | 18 |
| **Delayed Gratification** | 7.51 (2.00) |
| (Missing) | 8 |
| **Hope** | 8.08 (1.87) |
| (Missing) | 23 |
| **Gratitude** | 7.79 (2.09) |
| (Missing) | 20 |
| **Love** | 8.28 (1.96) |
| (Missing) | 14 |
| **Forgiveness** | |
| Always | 1,389 (22%) |
| Often | 3,516 (56%) |
| Rarely | 1,209 (19%) |
| Never | 169 (2.7%) |
| (Missing) | 6 (0.1%) |
| **Charitable Giving** | |
| Yes | 2,266 (36%) |
| No | 4,005 (64%) |
| (Missing) | 19 (0.3%) |
| **Helping** | |
| Yes | 3,328 (53%) |

| Characteristic | N = 6,290[1] |
|---|---|
| No | 2,947 (47%) |
| (Missing) | 15 (0.2%) |
| **Volunteering** | |
| Yes | 1,090 (17%) |
| No | 5,178 (82%) |
| (Missing) | 21 (0.3%) |
| **Self-Rated Physical Health** | 6.83 (2.07) |
| (Missing) | 19 |
| **Health Limitations** | |
| Yes | 1,215 (19%) |
| No | 4,982 (79%) |
| (Missing) | 94 (1.5%) |
| **Pain** | |
| A lot | 803 (13%) |
| Some | 2,145 (34%) |
| Not very much | 2,096 (33%) |
| None at all | 1,242 (20%) |
| (Missing) | 4 (<0.1%) |
| **Smoking** | 4 (7) |
| (Missing) | 73 |
| **Drinking** | 3.5 (7.4) |
| (Missing) | 64 |
| **Exercise** | |
| 0 days | 1,811 (29%) |
| 1 day | 536 (8.5%) |
| 2 days | 828 (13%) |
| 3 days | 979 (16%) |
| 4 days | 582 (9.3%) |
| 5 days | 591 (9.4%) |
| 6 days | 251 (4.0%) |
| 7 days/Every day | 705 (11%) |
| (Missing) | 6 (<0.1%) |
| **Financial Stability** | 4.7 (3.3) |
| (Missing) | 8 |
| **Material Stability** | 5.0 (3.3) |
| (Missing) | 7 |
| **Education** | |
| Up to 8 years | 802 (13%) |
| 9-15 years | 4,145 (66%) |
| 16+ years | 1,341 (21%) |
| (Missing) | 2 (<0.1%) |
| **Employment** | |
| Employed for an employer | 2,862 (45%) |
| Self-employed | 576 (9.2%) |
| Retired | 1,278 (20%) |
| Student | 448 (7.1%) |
| Homemaker | 345 (5.5%) |
| Unemployed and looking for a job | 646 (10%) |
| None of these/Other | 123 (2.0%) |
| (Missing) | 11 (0.2%) |
| **Subjective Financial Well-Being** | |
| Living comfortably on present income | 1,176 (19%) |
| Getting by on present income | 2,717 (43%) |
| Finding it difficult on present income | 1,707 (27%) |
| Finding it very difficult on present income | 548 (8.7%) |
| (Missing) | 142 (2.3%) |
| **Housing** | |
| Someone in this household OWNS this home | 3,584 (57%) |
| Someone in this household RENTS this home | 1,161 (18%) |
| Both | 177 (2.8%) |
| Neither | 1,230 (20%) |
| Rent | 0 (0%) |
| Own | 0 (0%) |
| Something else | 0 (0%) |
| (Missing) | 139 (2.2%) |
| **Self-Reported Religion/Spirituality** | |
| Always | 1,070 (17%) |
| Often | 1,620 (26%) |
| Rarely | 1,994 (32%) |

| Characteristic | N = 6,290[1] |
|---|---|
| Never | 1,597 (25%) |
| (Missing) | 9 (0.1%) |
| **Religious Service Attendance** | |
| More than once a week | 317 (5.0%) |
| Once a week | 662 (11%) |
| One to three times a month | 437 (6.9%) |
| A few times a year | 1,972 (31%) |
| Never | 2,875 (46%) |
| (Missing) | 27 (0.4%) |
| **Life after Death Belief** | |
| Yes | 2,495 (40%) |
| No | 1,732 (28%) |
| Unsure | 2,017 (32%) |
| (Missing) | 46 (0.7%) |
| **Religious Experience** | |
| Yes | 1,318 (21%) |
| No | 4,941 (79%) |
| (Missing) | 31 (0.5%) |
| **Religious Reading** | |
| More than once a day | 264 (4.2%) |
| About once a day | 460 (7.3%) |
| Sometimes | 2,243 (36%) |
| Never | 3,281 (52%) |
| (Missing) | 43 (0.7%) |
| **Prayer-Meditation** | |
| More than once a day | 590 (9.4%) |
| About once a day | 1,051 (17%) |
| Sometimes | 2,423 (39%) |
| Never | 2,207 (35%) |
| (Missing) | 19 (0.3%) |
| **Belief in God** | |
| One God | 2,796 (44%) |
| More than one god | 125 (2.0%) |
| An impersonal spiritual force | 1,066 (17%) |
| None of these | 1,425 (23%) |
| Unsure | 853 (14%) |
| (Missing) | 25 (0.4%) |
| **Intrinsic Religiosity** | |
| Agree | 1,659 (26%) |
| Disagree | 1,292 (21%) |
| Not relevant | 2,471 (39%) |
| Unsure | 788 (13%) |
| (Missing) | 80 (1.3%) |
| **Religious Comfort** | |
| Agree | 2,288 (36%) |
| Disagree | 1,171 (19%) |
| Not relevant | 2,117 (34%) |
| Unsure | 676 (11%) |
| (Missing) | 37 (0.6%) |
| **Loved by God** | |
| Agree | 2,277 (36%) |
| Disagree | 1,185 (19%) |
| Not relevant | 2,031 (32%) |
| Unsure | 758 (12%) |
| (Missing) | 40 (0.6%) |
| **Spiritual Punishment** | |
| Agree | 586 (9.3%) |
| Disagree | 3,034 (48%) |
| Not relevant | 1,939 (31%) |
| Unsure | 691 (11%) |
| (Missing) | 41 (0.6%) |
| **Religious Criticism** | |
| Agree | 388 (6.2%) |
| Disagree | 2,707 (43%) |
| Not relevant | 2,411 (38%) |
| Unsure | 737 (12%) |
| (Missing) | 47 (0.8%) |
| **Evangelism** | |
| Agree | 1,633 (26%) |

| Characteristic | N = 6,290[1] |
| --- | --- |
| Disagree | 2,095 (33%) |
| Not relevant | 2,174 (35%) |
| Unsure | 352 (5.6%) |
| (Missing) | 36 (0.6%) |
| **Children** | 0.64 (1.25) |
| (Missing) | 55 |

[1]Mean (SD); n (%)

**Table S17c. Demographic variation across outcomes for Spain**

| Outcome | Male | Female | Other | Male vs Female p-value | Global p-value |
|---|---|---|---|---|---|
| *Flourishing Index and Domains* | | | | | |
| Flourishing Index | 7.38 (7.31,7.45) | 7.23 (7.17,7.30) | 7.70 (6.25,9.15) | 2.27e-01 | 2.87e-03 |
| Secure Flourishing Index | 7.02 (6.96,7.09) | 6.77 (6.70,6.83) | 7.47 (6.02,8.93) | 2.1e-03 | 4.48e-08 |
| Happiness & Life Satisfaction | 6.95 (6.85,7.04) | 6.76 (6.67,6.85) | 7.14 (5.37,8.91) | 2.18e-01 | 1.33e-02 |
| Social Relationship Quality | 7.42 (7.33,7.51) | 7.39 (7.30,7.49) | 7.32 (4.42,10.21) | 6.25e-01 | 9.25e-01 |
| Meaning and Purpose | 7.39 (7.30,7.48) | 7.25 (7.16,7.34) | 8.00 (6.86,9.13) | 5.73e-01 | 1.17e-03 |
| Character & Virtue | 7.82 (7.75,7.89) | 7.79 (7.72,7.86) | 8.10 (6.75,9.45) | 5.46e-01 | 4.56e-01 |
| Self-Rated Health | 7.33 (7.25,7.41) | 6.97 (6.88,7.06) | 7.94 (6.08,9.79) | 3.55e-04 | 6.48e-09 |
| Financial and Material Worry | 5.23 (5.09,5.37) | 4.45 (4.31,4.59) | 6.35 (2.99,9.70) | 4.46e-09 | 9.1e-15 |
| *Psychological Well-Being* | | | | | |
| Happiness | 7.00 (6.91,7.10) | 6.83 (6.74,6.92) | 7.10 (5.54,8.66) | 2.38e-01 | 3.64e-02 |
| Life Satisfaction | 6.89 (6.79,6.99) | 6.68 (6.58,6.78) | 7.19 (4.93,9.44) | 2.37e-01 | 1.1e-02 |
| Present Life Evaluation | 6.72 (6.63,6.81) | 6.61 (6.52,6.70) | 7.10 (3.74,10.45) | 5.43e-01 | 2.02e-01 |
| Future Life Evaluation | 7.60 (7.51,7.69) | 7.61 (7.52,7.70) | 9.23 (7.53,10.92) | 9.38e-01 | 1.04e-05 |
| Optimism | 8.23 (8.14,8.32) | 8.26 (8.17,8.34) | 8.70 (7.73,9.66) | 8.71e-01 | 6.11e-02 |
| Freedom | 7.43 (7.33,7.54) | 7.46 (7.36,7.57) | 8.12 (6.20,10.04) | 5.43e-01 | 2.02e-01 |
| Peace | 0.57 (0.55,0.59) | 0.54 (0.51,0.56) | 0.38 (0.00,1.22) | 4.77e-02 | 7.41e-02 |
| Balance in Life | 0.79 (0.77,0.81) | 0.74 (0.72,0.76) | 0.59 (0.00,1.53) | 1.32e-03 | 1.06e-03 |
| Mastery | 0.86 (0.84,0.88) | 0.86 (0.84,0.87) | 1.00 (1.00,1.00) | 3.29e-01 | 1.6e-16 |
| Meaning | 7.34 (7.24,7.45) | 7.25 (7.16,7.35) | 8.28 (6.85,9.72) | 9.7e-01 | 1.41e-03 |
| Purpose | 7.43 (7.33,7.53) | 7.24 (7.14,7.34) | 7.71 (6.10,9.32) | 3.27e-01 | 1.9e-02 |
| Self-Rated Mental Health | 7.67 (7.57,7.77) | 7.25 (7.14,7.35) | 8.10 (6.21,9.99) | 2.76e-03 | 9.88e-09 |
| *Social Well-Being* | | | | | |
| Content with My Relationships | 7.71 (7.62,7.81) | 7.72 (7.62,7.81) | 7.18 (4.11,10.25) | 2.78e-01 | 6.79e-01 |
| Satisfying Relationships | 7.12 (7.01,7.23) | 7.07 (6.96,7.18) | 7.45 (4.66,10.25) | 9.17e-01 | 6.53e-01 |
| Social Support | 8.01 (7.91,8.11) | 8.14 (8.04,8.24) | 7.72 (3.79,11.64) | 2.15e-02 | 1.75e-01 |
| Intimate Friend | 0.86 (0.84,0.87) | 0.88 (0.87,0.90) | 0.87 (0.42,1.33) | 1.95e-01 | 5.57e-02 |
| Government Approval | 0.38 (0.36,0.40) | 0.33 (0.31,0.36) | 0.52 (0.00,1.42) | 2.6e-01 | 1.1e-02 |
| Political Voice | 0.22 (0.19,0.24) | 0.18 (0.16,0.20) | 0.26 (0.00,1.27) | 3.65e-02 | 2.25e-02 |
| Belonging | 7.52 (7.39,7.64) | 7.48 (7.38,7.59) | 7.43 (4.29,10.57) | 2.74e-01 | 9.16e-01 |
| City Satisfaction | 0.87 (0.85,0.88) | 0.86 (0.85,0.88) | 0.87 (0.00,2.21) | 7.96e-01 | 8.08e-01 |
| Trust | 0.19 (0.17,0.21) | 0.16 (0.14,0.18) | 0.35 (0.00,1.21) | 1.83e-01 | 4.4e-02 |
| Community Participation | 0.24 (0.22,0.26) | 0.22 (0.20,0.23) | 0.41 (0.00,1.36) | 1.95e-02 | 8.47e-02 |
| *Psychological Distress* | | | | | |
| Traumatic Distress | 0.35 (0.33,0.38) | 0.38 (0.36,0.40) | 0.43 (0.00,1.27) | 1.49e-02 | 2.34e-01 |
| Depression Symptoms | 0.27 (0.25,0.29) | 0.31 (0.29,0.33) | 0.24 (0.00,0.97) | 9.92e-03 | 1.66e-02 |
| Anxiety Symptoms | 0.29 (0.27,0.31) | 0.35 (0.33,0.37) | 0.47 (0.00,1.36) | 3.83e-05 | 2.41e-04 |
| Suffering | 0.43 (0.40,0.45) | 0.54 (0.51,0.56) | 0.78 (0.04,1.51) | 1.82e-06 | 4.29e-11 |
| *Social Distress* | | | | | |
| Loneliness | 3.26 (3.13,3.39) | 3.38 (3.25,3.50) | 4.11 (0.91,7.32) | 3.89e-01 | 1.99e-01 |
| Discrimination | 0.21 (0.19,0.23) | 0.17 (0.16,0.19) | 0.53 (0.00,1.45) | 3.05e-02 | 9.87e-04 |
| *Character & Prosocial Behavior* | | | | | |
| Promoting Good | 8.07 (8.00,8.15) | 8.13 (8.05,8.20) | 8.21 (7.03,9.38) | 2.01e-02 | 5.55e-01 |
| Delayed Gratification | 7.56 (7.47,7.66) | 7.45 (7.36,7.54) | 8.00 (6.25,9.74) | 3.02e-01 | 8.45e-02 |
| Hope | 8.12 (8.03,8.20) | 8.03 (7.95,8.12) | 8.65 (7.55,9.74) | 9.22e-01 | 1.36e-02 |
| Gratitude | 7.76 (7.67,7.85) | 7.82 (7.72,7.92) | 8.42 (6.98,9.86) | 2.26e-01 | 5.89e-02 |
| Love | 8.10 (8.01,8.19) | 8.47 (8.38,8.56) | 7.81 (5.27,10.34) | 9.09e-07 | 4.5e-08 |
| Forgiveness | 0.78 (0.76,0.80) | 0.78 (0.77,0.80) | 0.54 (0.00,1.49) | 5.54e-01 | 3.78e-01 |
| Charitable Giving | 0.42 (0.40,0.45) | 0.30 (0.28,0.32) | 0.00 * | 9.99e-07 | 1.6e-16 |
| Helping | 0.61 (0.58,0.63) | 0.45 (0.43,0.48) | 0.86 (0.21,1.51) | 3.63e-07 | 1.6e-16 |
| Volunteering | 0.22 (0.20,0.24) | 0.12 (0.11,0.14) | 0.26 (0.00,1.01) | 5.83e-07 | 4.24e-14 |
| *Physical Health & Health Behavior* | | | | | |
| Self-Rated Physical Health | 6.98 (6.89,7.08) | 6.68 (6.58,6.79) | 7.78 (4.96,10.59) | 8.38e-04 | 2.97e-05 |
| Health Limitations | 0.19 (0.17,0.21) | 0.20 (0.18,0.22) | 0.32 (0.00,1.03) | 6.12e-01 | 6.4e-01 |
| Pain | 0.42 (0.40,0.45) | 0.51 (0.49,0.54) | 0.47 (0.00,1.36) | 4.98e-05 | 6.82e-07 |
| Smoking | 4.15 (3.80,4.50) | 3.01 (2.74,3.27) | 5.08 (0.00,14.91) | 1.34e-05 | 1.08e-06 |
| Drinking | 3.87 (3.60,4.15) | 2.73 (2.44,3.01) | 4.99 (0.00,20.41) | 2.32e-06 | 5.28e-08 |
| Exercise | 2.86 (2.75,2.98) | 2.50 (2.39,2.61) | 3.90 (0.00,8.74) | 4.16e-02 | 1.67e-05 |
| *Socioeconomic Outcomes* | | | | | |
| Financial Stability | 5.11 (4.95,5.26) | 4.28 (4.13,4.43) | 5.59 (2.60,8.58) | 1.29e-08 | 6.81e-14 |
| Material Stability | 5.35 (5.20,5.50) | 4.61 (4.46,4.76) | 7.10 (3.10,11.10) | 1.23e-07 | 2.44e-12 |
| Education | 0.21 (0.19,0.23) | 0.22 (0.20,0.23) | 0.43 (0.00,1.39) | 9.59e-01 | 4.51e-01 |
| Employment | 0.58 (0.56,0.61) | 0.51 (0.49,0.54) | 0.76 (0.04,1.48) | 1.15e-02 | 9.77e-05 |
| Subjective Financial Well-Being | 0.66 (0.64,0.69) | 0.60 (0.58,0.63) | 0.28 (0.00,1.05) | 1.12e-03 | 5.87e-05 |
| Housing | 0.63 (0.60,0.65) | 0.60 (0.58,0.63) | 0.17 (0.00,0.70) | 8.38e-01 | 5.06e-05 |
| *Religion/Spirituality* | | | | | |
| Self-Reported Religion/Spirituality | 0.43 (0.41,0.45) | 0.42 (0.40,0.45) | 0.67 (0.00,1.47) | 8.39e-01 | 2.8e-01 |

| Outcome | Male | Female | Other | Male vs Female p-value | Global p-value |
|---|---|---|---|---|---|
| Religious Service Attendance | 0.18 (0.16,0.19) | 0.14 (0.12,0.16) | 0.24 (0.00,0.97) | 6.77e-03 | 8.46e-03 |
| Life after Death Belief | 0.39 (0.37,0.42) | 0.41 (0.38,0.43) | 0.50 (0.00,1.41) | 5.94e-01 | 6.32e-01 |
| Religious Experience | 0.22 (0.20,0.24) | 0.20 (0.19,0.22) | 0.61 (0.00,1.44) | 3.8e-01 | 2.09e-02 |
| Religious Reading | 0.12 (0.11,0.14) | 0.11 (0.09,0.12) | 0.24 (0.00,0.97) | 4.35e-01 | 2.45e-01 |
| Prayer-Meditation | 0.26 (0.24,0.28) | 0.26 (0.24,0.28) | 0.60 (0.00,1.45) | 9.22e-01 | 1.44e-01 |
| Belief in God | 0.62 (0.60,0.64) | 0.65 (0.63,0.67) | 0.87 (0.42,1.33) | 1.7e-02 | 5.97e-03 |
| Intrinsic Religiosity | 0.45 (0.42,0.48) | 0.44 (0.41,0.46) | 0.72 * | 7.47e-01 | 1.68e-02 |
| Religious Comfort | 0.51 (0.48,0.55) | 0.51 (0.48,0.53) | 0.77 (0.05,1.48) | 6.86e-01 | 7.7e-02 |
| Loved by God | 0.51 (0.49,0.54) | 0.50 (0.48,0.53) | 0.75 (0.11,1.39) | 6.98e-01 | 1.26e-01 |
| Spiritual Punishment | 0.17 (0.15,0.19) | 0.15 (0.13,0.17) | 0.25 (0.00,1.14) | 3.28e-01 | 1.32e-01 |
| Religious Criticism | 0.16 (0.14,0.18) | 0.12 (0.10,0.14) | 0.31 (0.00,1.10) | 2.85e-02 | 7.44e-04 |
| Evangelism | 0.38 (0.36,0.41) | 0.36 (0.34,0.39) | 0.60 (0.00,1.67) | 4.87e-01 | 1.7e-01 |
| *Family Factors* | | | | | |
| Ever Married | 0.62 (0.59,0.64) | 0.63 (0.61,0.66) | 0.25 (0.00,0.90) | 3.2e-02 | 8.14e-03 |
| Divorced | 0.07 (0.06,0.09) | 0.09 (0.08,0.11) | 0.07 (0.00,0.43) | 5.84e-02 | 1.42e-01 |
| Children | 0.69 (0.60,0.77) | 0.59 (0.55,0.62) | 0.64 (0.00,1.96) | 3.45e-01 | 1.08e-01 |

**Table S18a. Nationally representative descriptive statistics for Sweden**

| Characteristic | N = 15,068[1] |
|---|---|
| **Age group** | |
| 18-24 | 1,515 (10%) |
| 25-29 | 1,399 (9.3%) |
| 30-39 | 2,398 (16%) |
| 40-49 | 2,221 (15%) |
| 50-59 | 2,493 (17%) |
| 60-69 | 2,168 (14%) |
| 70-79 | 2,253 (15%) |
| 80 or older | 621 (4.1%) |
| (Missing) | 0 (0%) |
| **Gender** | |
| Male | 7,536 (50%) |
| Female | 7,493 (50%) |
| Other | 27 (0.2%) |
| (Missing) | 12 (<0.1%) |
| **Marital status** | |
| Married | 6,408 (43%) |
| Separated | 426 (2.8%) |
| Divorced | 801 (5.3%) |
| Widowed | 433 (2.9%) |
| Single, never married | 3,854 (26%) |
| Domestic Partner | 3,073 (20%) |
| (Missing) | 72 (0.5%) |
| **Employment** | |
| Employed for an employer | 7,907 (52%) |
| Self-employed | 1,243 (8.3%) |
| Retired | 3,832 (25%) |
| Student | 1,332 (8.8%) |
| Homemaker | 75 (0.5%) |
| Unemployed and looking for a job | 324 (2.2%) |
| None of these/Other | 337 (2.2%) |
| (Missing) | 18 (0.1%) |
| **Religious service attendance** | |
| More than 1/week | 236 (1.6%) |
| 1/week | 434 (2.9%) |
| 1-3/month | 486 (3.2%) |
| A few times a year | 3,950 (26%) |
| Never | 9,918 (66%) |
| (Missing) | 45 (0.3%) |
| **Education** | |
| Up to 8 years | 252 (1.7%) |
| 9-15 years | 10,790 (72%) |
| 16+ years | 4,026 (27%) |
| (Missing) | 0 (0%) |
| **Immigration** | |
| Born in this country | 13,922 (92%) |
| Born in another country | 1,052 (7.0%) |
| (Missing) | 94 (0.6%) |
| **Religious affiliation** | |
| Christianity | 8,346 (55%) |
| Islam | 470 (3.1%) |
| Hinduism | 22 (0.1%) |
| Buddhism | 110 (0.7%) |
| Judaism | 54 (0.4%) |
| Sikhism | 4 (<0.1%) |
| Baha'i | 6 (<0.1%) |
| Jainism | 0 (0%) |
| Shinto | 0 (<0.1%) |
| Taoism | 4 (<0.1%) |
| Confucianism | 0 (0%) |
| Primal, Animist, or Folk religion | 83 (0.5%) |
| Spiritism | 0 (0%) |
| Umbanda, Candomble, and other African-derived religions | 0 (0%) |
| Chinese folk/traditional religion | 0 (0%) |
| Some other religion | 198 (1.3%) |
| No religion/Atheist/Agnostic | 5,697 (38%) |
| (Missing) | 74 (0.5%) |

[1]n (%)

**Table S18b. Descriptive statistics of outcome variables for Sweden**

| Characteristic | N = 15,068[1] |
|---|---|
| **Flourishing Index** | 7.04 (1.57) |
| (Missing) | 458 |
| **Secure Flourishing Index** | 7.09 (1.54) |
| (Missing) | 509 |
| **Happiness & Life Satisfaction** | 7.06 (1.95) |
| (Missing) | 62 |
| **Social Relationship Quality** | 7.23 (2.23) |
| (Missing) | 96 |
| **Meaning and Purpose** | 6.85 (2.16) |
| (Missing) | 165 |
| **Character & Virtue** | 7.28 (1.56) |
| (Missing) | 171 |
| **Self-Rated Health** | 6.80 (1.85) |
| (Missing) | 47 |
| **Financial and Material Worry** | 7.36 (2.49) |
| (Missing) | 64 |
| **Happiness** | 7.03 (1.96) |
| (Missing) | 38 |
| **Life Satisfaction** | 7.09 (2.07) |
| (Missing) | 34 |
| **Present Life Evaluation** | 7.20 (1.76) |
| (Missing) | 45 |
| **Future Life Evaluation** | 7.70 (1.76) |
| (Missing) | 43 |
| **Optimism** | 7.30 (2.32) |
| (Missing) | 52 |
| **Freedom** | 7.49 (2.24) |
| (Missing) | 39 |
| **Peace** | |
| Always | 1,723 (11%) |
| Often | 10,561 (70%) |
| Rarely | 2,587 (17%) |
| Never | 136 (0.9%) |
| (Missing) | 61 (0.4%) |
| **Balance in Life** | |
| Always | 1,284 (8.5%) |
| Often | 10,642 (71%) |
| Rarely | 2,883 (19%) |
| Never | 149 (1.0%) |
| (Missing) | 109 (0.7%) |
| **Mastery** | |
| Always | 2,336 (16%) |
| Often | 10,523 (70%) |
| Rarely | 1,993 (13%) |
| Never | 128 (0.9%) |
| (Missing) | 88 (0.6%) |
| **Meaning** | 7.06 (2.21) |
| (Missing) | 43 |
| **Purpose** | 6.65 (2.54) |
| (Missing) | 124 |
| **Self-Rated Mental Health** | 7.18 (2.25) |
| (Missing) | 19 |
| **Content with My Relationships** | 7.44 (2.19) |
| (Missing) | 43 |
| **Satisfying Relationships** | 7.01 (2.43) |
| (Missing) | 65 |
| **Social Support** | 8.16 (2.12) |
| (Missing) | 58 |
| **Intimate Friend** | |
| Yes | 13,019 (86%) |
| No | 2,012 (13%) |
| (Missing) | 37 (0.2%) |
| **Government Approval** | |
| Strongly approve | 761 (5.0%) |
| Somewhat approve | 2,850 (19%) |
| Neither approve nor disapprove | 4,244 (28%) |
| Somewhat disapprove | 3,986 (26%) |
| Strongly disapprove | 3,143 (21%) |

| Characteristic | N = 15,068[1] |
|---|---|
| (Missing) | 85 (0.6%) |
| **Political Voice** | |
| Agree | 3,298 (22%) |
| Disagree | 7,782 (52%) |
| Unsure | 3,913 (26%) |
| (Missing) | 75 (0.5%) |
| **Belonging** | 8.58 (2.00) |
| (Missing) | 56 |
| **City Satisfaction** | |
| Satisfied | 12,321 (82%) |
| Dissatisfied | 1,399 (9.3%) |
| Unsure | 1,285 (8.5%) |
| (Missing) | 63 (0.4%) |
| **Trust** | |
| All | 60 (0.4%) |
| Most | 5,726 (38%) |
| Some | 6,107 (41%) |
| Not very many | 3,018 (20%) |
| None | 73 (0.5%) |
| (Missing) | 85 (0.6%) |
| **Community Participation** | |
| More than once a week | 1,640 (11%) |
| Once a week | 1,290 (8.6%) |
| One to three times a month | 1,896 (13%) |
| A few times a year | 3,365 (22%) |
| Never | 6,853 (45%) |
| (Missing) | 24 (0.2%) |
| **Traumatic Distress** | |
| A lot | 700 (4.6%) |
| Some | 3,056 (20%) |
| Not very much | 5,731 (38%) |
| None at all | 5,413 (36%) |
| (Missing) | 169 (1.1%) |
| **Suffering** | |
| A lot | 773 (5.1%) |
| Some | 4,373 (29%) |
| Not very much | 6,359 (42%) |
| None at all | 3,494 (23%) |
| (Missing) | 69 (0.5%) |
| **Loneliness** | 3.03 (2.66) |
| (Missing) | 30 |
| **Discrimination** | |
| Always | 260 (1.7%) |
| Often | 1,251 (8.3%) |
| Rarely | 5,272 (35%) |
| Never | 8,220 (55%) |
| (Missing) | 66 (0.4%) |
| **Promoting Good** | 7.56 (1.71) |
| (Missing) | 90 |
| **Delayed Gratification** | 6.99 (2.04) |
| (Missing) | 113 |
| **Hope** | 7.26 (2.22) |
| (Missing) | 59 |
| **Gratitude** | 7.48 (2.24) |
| (Missing) | 48 |
| **Love** | 8.11 (1.87) |
| (Missing) | 44 |
| **Forgiveness** | |
| Always | 2,004 (13%) |
| Often | 9,445 (63%) |
| Rarely | 3,241 (22%) |
| Never | 276 (1.8%) |
| (Missing) | 102 (0.7%) |
| **Charitable Giving** | |
| Yes | 7,822 (52%) |
| No | 7,198 (48%) |
| (Missing) | 49 (0.3%) |
| **Helping** | |
| Yes | 6,548 (43%) |

| Characteristic | N = 15,068[1] |
|---|---|
| No | 8,478 (56%) |
| (Missing) | 42 (0.3%) |
| **Volunteering** | |
| Yes | 1,708 (11%) |
| No | 13,293 (88%) |
| (Missing) | 67 (0.4%) |
| **Self-Rated Physical Health** | 6.41 (2.07) |
| (Missing) | 34 |
| **Health Limitations** | |
| Yes | 3,859 (26%) |
| No | 11,159 (74%) |
| (Missing) | 50 (0.3%) |
| **Pain** | |
| A lot | 1,343 (8.9%) |
| Some | 4,608 (31%) |
| Not very much | 5,915 (39%) |
| None at all | 3,160 (21%) |
| (Missing) | 42 (0.3%) |
| **Smoking** | 0.84 (3.34) |
| (Missing) | 457 |
| **Drinking** | 2.9 (4.6) |
| (Missing) | 203 |
| **Exercise** | |
| 0 days | 3,339 (22%) |
| 1 day | 1,842 (12%) |
| 2 days | 2,359 (16%) |
| 3 days | 2,314 (15%) |
| 4 days | 1,523 (10%) |
| 5 days | 1,442 (9.6%) |
| 6 days | 706 (4.7%) |
| 7 days/Every day | 1,521 (10%) |
| (Missing) | 23 (0.2%) |
| **Financial Stability** | 7.23 (2.79) |
| (Missing) | 31 |
| **Material Stability** | 7.50 (2.57) |
| (Missing) | 48 |
| **Education** | |
| Up to 8 years | 252 (1.7%) |
| 9-15 years | 10,790 (72%) |
| 16+ years | 4,026 (27%) |
| (Missing) | 0 (0%) |
| **Employment** | |
| Employed for an employer | 7,907 (52%) |
| Self-employed | 1,243 (8.3%) |
| Retired | 3,832 (25%) |
| Student | 1,332 (8.8%) |
| Homemaker | 75 (0.5%) |
| Unemployed and looking for a job | 324 (2.2%) |
| None of these/Other | 337 (2.2%) |
| (Missing) | 18 (0.1%) |
| **Subjective Financial Well-Being** | |
| Living comfortably on present income | 6,820 (45%) |
| Getting by on present income | 7,367 (49%) |
| Finding it difficult on present income | 650 (4.3%) |
| Finding it very difficult on present income | 159 (1.1%) |
| (Missing) | 72 (0.5%) |
| **Housing** | |
| Someone in this household OWNS this home | 0 (0%) |
| Someone in this household RENTS this home | 0 (0%) |
| Both | 0 (0%) |
| Neither | 0 (0%) |
| Rent | 4,224 (28%) |
| Own | 10,600 (70%) |
| Something else | 0 (0%) |
| (Missing) | 244 (1.6%) |
| **Self-Reported Religion/Spirituality** | |
| Always | 1,030 (6.8%) |
| Often | 2,816 (19%) |
| Rarely | 5,935 (39%) |

| Characteristic | N = 15,068[1] |
|---|---|
| Never | 5,234 (35%) |
| (Missing) | 53 (0.4%) |
| **Religious Service Attendance** | |
| More than once a week | 236 (1.6%) |
| Once a week | 434 (2.9%) |
| One to three times a month | 486 (3.2%) |
| A few times a year | 3,950 (26%) |
| Never | 9,918 (66%) |
| (Missing) | 45 (0.3%) |
| **Life after Death Belief** | |
| Yes | 4,387 (29%) |
| No | 5,530 (37%) |
| Unsure | 5,072 (34%) |
| (Missing) | 78 (0.5%) |
| **Religious Experience** | |
| Yes | 1,998 (13%) |
| No | 12,996 (86%) |
| (Missing) | 74 (0.5%) |
| **Religious Reading** | |
| More than once a day | 200 (1.3%) |
| About once a day | 495 (3.3%) |
| Sometimes | 3,720 (25%) |
| Never | 10,586 (70%) |
| (Missing) | 67 (0.4%) |
| **Prayer-Meditation** | |
| More than once a day | 540 (3.6%) |
| About once a day | 1,185 (7.9%) |
| Sometimes | 4,471 (30%) |
| Never | 8,831 (59%) |
| (Missing) | 40 (0.3%) |
| **Belief in God** | |
| One God | 2,993 (20%) |
| More than one god | 411 (2.7%) |
| An impersonal spiritual force | 3,187 (21%) |
| None of these | 5,812 (39%) |
| Unsure | 2,593 (17%) |
| (Missing) | 72 (0.5%) |
| **Intrinsic Religiosity** | |
| Agree | 1,889 (13%) |
| Disagree | 4,775 (32%) |
| Not relevant | 6,925 (46%) |
| Unsure | 1,336 (8.9%) |
| (Missing) | 144 (1.0%) |
| **Religious Comfort** | |
| Agree | 3,260 (22%) |
| Disagree | 3,407 (23%) |
| Not relevant | 6,830 (45%) |
| Unsure | 1,431 (9.5%) |
| (Missing) | 140 (0.9%) |
| **Loved by God** | |
| Agree | 2,705 (18%) |
| Disagree | 3,560 (24%) |
| Not relevant | 6,966 (46%) |
| Unsure | 1,673 (11%) |
| (Missing) | 165 (1.1%) |
| **Spiritual Punishment** | |
| Agree | 606 (4.0%) |
| Disagree | 6,729 (45%) |
| Not relevant | 6,427 (43%) |
| Unsure | 1,162 (7.7%) |
| (Missing) | 144 (1.0%) |
| **Religious Criticism** | |
| Agree | 347 (2.3%) |
| Disagree | 4,555 (30%) |
| Not relevant | 9,287 (62%) |
| Unsure | 690 (4.6%) |
| (Missing) | 189 (1.3%) |
| **Evangelism** | |
| Agree | 2,844 (19%) |

| **Characteristic** | **N = 15,068**[1] |
|---|---|
| Disagree | 3,709 (25%) |
| Not relevant | 7,666 (51%) |
| Unsure | 698 (4.6%) |
| (Missing) | 150 (1.0%) |
| **Children** | 0.55 (1.03) |
| (Missing) | 98 |

[1]Mean (SD); n (%)

**Table S18c. Demographic variation across outcomes for Sweden**

| Outcome | Male | Female | Other | Male vs Female p-value | Global p-value |
|---|---|---|---|---|---|
| *Flourishing Index and Domains* | | | | | |
| Flourishing Index | 7.08 (7.04,7.12) | 7.01 (6.97,7.05) | 5.57 (4.80,6.33) | 4.64e-02 | 2.09e-05 |
| Secure Flourishing Index | 7.15 (7.11,7.19) | 7.05 (7.01,7.09) | 5.74 (5.06,6.43) | 4.25e-03 | 6.06e-07 |
| Happiness & Life Satisfaction | 7.05 (7.00,7.10) | 7.08 (7.03,7.13) | 4.97 (4.00,5.94) | 7.87e-01 | 2.93e-05 |
| Social Relationship Quality | 7.17 (7.12,7.23) | 7.28 (7.22,7.35) | 5.86 (4.58,7.13) | 1.35e-01 | 3.42e-03 |
| Meaning and Purpose | 6.86 (6.80,6.91) | 6.86 (6.80,6.92) | 4.95 (3.50,6.41) | 5.35e-01 | 2.44e-02 |
| Character & Virtue | 7.36 (7.32,7.40) | 7.19 (7.14,7.23) | 7.03 (6.16,7.89) | 2.91e-03 | 1.16e-07 |
| Self-Rated Health | 6.96 (6.91,7.00) | 6.64 (6.59,6.69) | 5.03 (4.46,5.59) | 9.82e-11 | 1.6e-16 |
| Financial and Material Worry | 7.49 (7.43,7.56) | 7.23 (7.17,7.30) | 6.64 (5.53,7.74) | 1.56e-05 | 1.28e-07 |
| *Psychological Well-Being* | | | | | |
| Happiness | 7.01 (6.96,7.06) | 7.06 (7.01,7.11) | 5.15 (4.38,5.91) | 9.53e-01 | 7.93e-07 |
| Life Satisfaction | 7.09 (7.04,7.15) | 7.10 (7.04,7.16) | 4.79 (3.52,6.06) | 6.39e-01 | 7.82e-04 |
| Present Life Evaluation | 7.19 (7.15,7.24) | 7.21 (7.16,7.26) | 5.67 (4.67,6.66) | 7.48e-01 | 5.19e-03 |
| Future Life Evaluation | 7.68 (7.63,7.72) | 7.73 (7.68,7.78) | 7.21 (6.39,8.03) | 1.28e-01 | 1.16e-01 |
| Optimism | 7.31 (7.25,7.37) | 7.29 (7.22,7.35) | 5.27 (4.30,6.24) | 9.15e-01 | 7.03e-05 |
| Freedom | 7.43 (7.37,7.49) | 7.56 (7.50,7.62) | 6.12 (4.89,7.36) | 5.89e-02 | 9.78e-04 |
| Peace | 0.84 (0.83,0.85) | 0.79 (0.78,0.80) | 0.53 (0.29,0.77) | 4.85e-08 | 2.15e-12 |
| Balance in Life | 0.83 (0.82,0.84) | 0.77 (0.75,0.78) | 0.49 (0.26,0.73) | 3.59e-12 | <2e-16 |
| Mastery | 0.88 (0.87,0.89) | 0.84 (0.83,0.85) | 0.58 (0.35,0.81) | 3.07e-07 | 1.3e-09 |
| Meaning | 7.02 (6.97,7.08) | 7.10 (7.04,7.16) | 5.17 (3.90,6.45) | 6.08e-01 | 1.9e-03 |
| Purpose | 6.69 (6.62,6.76) | 6.62 (6.55,6.69) | 4.73 (3.01,6.45) | 1.4e-01 | 2.45e-02 |
| Self-Rated Mental Health | 7.37 (7.31,7.43) | 7.01 (6.94,7.07) | 3.67 (2.72,4.61) | 4.17e-11 | 1.6e-16 |
| *Social Well-Being* | | | | | |
| Content with My Relationships | 7.38 (7.32,7.44) | 7.51 (7.45,7.57) | 5.99 (4.65,7.34) | 1.55e-01 | 1.11e-03 |
| Satisfying Relationships | 6.97 (6.90,7.03) | 7.06 (6.99,7.13) | 5.72 (4.47,6.97) | 1.47e-01 | 1.35e-02 |
| Social Support | 8.02 (7.97,8.08) | 8.30 (8.24,8.35) | 6.62 (5.15,8.08) | 4.39e-07 | 4.71e-11 |
| Intimate Friend | 0.84 (0.83,0.85) | 0.89 (0.88,0.90) | 0.84 (0.67,1.00) | 1.69e-07 | 3.32e-13 |
| Government Approval | 0.29 (0.28,0.30) | 0.19 (0.18,0.20) | 0.09 (0.00,0.21) | 1.16e-10 | 1.6e-16 |
| Political Voice | 0.32 (0.30,0.34) | 0.29 (0.28,0.31) | 0.49 (0.11,0.86) | 1.66e-01 | 2.07e-03 |
| Belonging | 8.39 (8.33,8.45) | 8.78 (8.73,8.83) | 6.65 (5.47,7.82) | 4.59e-14 | 1.6e-16 |
| City Satisfaction | 0.88 (0.87,0.89) | 0.90 (0.89,0.91) | 0.77 (0.56,0.98) | 3.11e-01 | 1.76e-02 |
| Trust | 0.40 (0.39,0.42) | 0.37 (0.36,0.38) | 0.30 (0.09,0.52) | 4.25e-03 | 1.6e-03 |
| Community Participation | 0.21 (0.20,0.22) | 0.18 (0.17,0.19) | 0.40 (0.16,0.64) | 1.17e-02 | 1.44e-05 |
| *Psychological Distress* | | | | | |
| Traumatic Distress | 0.21 (0.20,0.22) | 0.30 (0.28,0.31) | 0.46 (0.22,0.69) | 2.98e-11 | 1.6e-16 |
| Depression Symptoms | 0.16 (0.15,0.17) | 0.20 (0.19,0.21) | 0.56 (0.33,0.79) | 1.29e-03 | 5.04e-08 |
| Anxiety Symptoms | 0.13 (0.12,0.14) | 0.18 (0.17,0.19) | 0.57 (0.33,0.80) | 6.96e-09 | <2e-16 |
| Suffering | 0.30 (0.29,0.31) | 0.39 (0.37,0.40) | 0.84 (0.71,0.97) | 5.53e-09 | 1.6e-16 |
| *Social Distress* | | | | | |
| Loneliness | 2.93 (2.86,3.00) | 3.12 (3.04,3.19) | 4.74 (3.36,6.12) | 2.22e-03 | 6.63e-05 |
| Discrimination | 0.11 (0.10,0.11) | 0.10 (0.09,0.10) | 0.38 (0.15,0.61) | 1.38e-01 | 1.29e-02 |
| *Character & Prosocial Behavior* | | | | | |
| Promoting Good | 7.49 (7.45,7.54) | 7.62 (7.57,7.67) | 7.41 (6.59,8.22) | 4.47e-04 | 9.29e-04 |
| Delayed Gratification | 7.23 (7.18,7.28) | 6.76 (6.70,6.82) | 6.65 (5.60,7.70) | 6.28e-14 | 1.6e-16 |
| Hope | 7.28 (7.22,7.34) | 7.25 (7.19,7.31) | 4.92 (3.69,6.15) | 8.89e-01 | 2.99e-04 |
| Gratitude | 7.23 (7.17,7.29) | 7.74 (7.68,7.80) | 5.75 (4.50,6.99) | <2e-16 | 1.6e-16 |
| Love | 7.77 (7.72,7.82) | 8.46 (8.41,8.50) | 8.03 (6.93,9.13) | 1.6e-16 | 1.6e-16 |
| Forgiveness | 0.76 (0.75,0.77) | 0.77 (0.76,0.78) | 0.70 (0.48,0.92) | 2.38e-01 | 3.48e-01 |
| Charitable Giving | 0.48 (0.47,0.49) | 0.56 (0.55,0.58) | 0.48 (0.24,0.72) | 6.21e-05 | 6.66e-16 |
| Helping | 0.42 (0.41,0.44) | 0.45 (0.43,0.46) | 0.47 (0.24,0.71) | 5.55e-01 | 8.48e-02 |
| Volunteering | 0.11 (0.10,0.12) | 0.12 (0.11,0.13) | 0.25 (0.06,0.43) | 4.94e-01 | 1.34e-01 |
| *Physical Health & Health Behavior* | | | | | |
| Self-Rated Physical Health | 6.54 (6.49,6.60) | 6.27 (6.21,6.33) | 6.38 (5.46,7.31) | 9.39e-06 | 2.33e-10 |
| Health Limitations | 0.21 (0.20,0.23) | 0.30 (0.29,0.31) | 0.64 (0.43,0.85) | 2.37e-11 | 1.6e-16 |
| Pain | 0.35 (0.34,0.36) | 0.44 (0.43,0.45) | 0.49 (0.25,0.73) | 3.39e-07 | 1.6e-16 |
| Smoking | 0.73 (0.65,0.82) | 0.93 (0.84,1.02) | 0.85 (0.00,2.14) | 3.18e-02 | 9.77e-03 |
| Drinking | 3.57 (3.43,3.70) | 2.12 (2.03,2.21) | 1.44 (0.47,2.40) | 1.6e-16 | 1.6e-16 |
| Exercise | 2.78 (2.71,2.84) | 2.77 (2.70,2.83) | 2.29 (1.26,3.33) | 9.07e-01 | 6.23e-01 |
| *Socioeconomic Outcomes* | | | | | |
| Financial Stability | 7.41 (7.34,7.48) | 7.05 (6.97,7.12) | 6.28 (5.00,7.57) | 7.42e-07 | 3.47e-11 |
| Material Stability | 7.58 (7.51,7.65) | 7.42 (7.35,7.49) | 6.99 (5.92,8.06) | 3.41e-03 | 3.1e-03 |
| Education | 0.25 (0.24,0.27) | 0.28 (0.27,0.29) | 0.19 (0.05,0.33) | 1.63e-02 | 7.52e-03 |
| Employment | 0.65 (0.64,0.66) | 0.57 (0.55,0.58) | 0.31 (0.10,0.52) | 1.47e-08 | 1.6e-16 |
| Subjective Financial Well-Being | 0.96 (0.95,0.96) | 0.94 (0.93,0.94) | 0.90 (0.81,0.99) | 9.77e-06 | 6.21e-09 |
| Housing | 0.73 (0.72,0.75) | 0.70 (0.68,0.71) | 0.44 (0.21,0.68) | 7e-03 | 8.19e-06 |
| *Religion/Spirituality* | | | | | |
| Self-Reported Religion/Spirituality | 0.22 (0.21,0.24) | 0.29 (0.27,0.30) | 0.38 (0.13,0.63) | 8.36e-06 | 3.42e-11 |

| Outcome | Male | Female | Other | Male vs Female p-value | Global p-value |
|---|---|---|---|---|---|
| Religious Service Attendance | 0.05 (0.04,0.06) | 0.04 (0.03,0.05) | 0.08 (0.00,0.20) | 5.44e-03 | 1.2e-01 |
| Life after Death Belief | 0.23 (0.21,0.24) | 0.36 (0.35,0.38) | 0.17 (0.00,0.34) | 1.6e-16 | 1.6e-16 |
| Religious Experience | 0.12 (0.11,0.13) | 0.15 (0.14,0.16) | 0.15 (0.00,0.31) | 2.82e-02 | 3.61e-05 |
| Religious Reading | 0.05 (0.04,0.06) | 0.04 (0.04,0.05) | 0.00 * | 2.43e-02 | 1.6e-16 |
| Prayer-Meditation | 0.10 (0.09,0.11) | 0.13 (0.12,0.14) | 0.10 (0.00,0.22) | 3.98e-02 | 2.02e-03 |
| Belief in God | 0.38 (0.37,0.40) | 0.50 (0.48,0.51) | 0.65 (0.45,0.85) | 8.88e-16 | 1.6e-16 |
| Intrinsic Religiosity | 0.17 (0.16,0.18) | 0.21 (0.19,0.22) | 0.13 (0.00,0.32) | 1.13e-02 | 3.18e-05 |
| Religious Comfort | 0.27 (0.26,0.28) | 0.35 (0.34,0.37) | 0.52 (0.24,0.80) | 2.71e-10 | 1.6e-16 |
| Loved by God | 0.24 (0.23,0.26) | 0.31 (0.30,0.33) | 0.43 (0.16,0.70) | 3.61e-07 | 1.23e-13 |
| Spiritual Punishment | 0.08 (0.07,0.09) | 0.07 (0.06,0.08) | 0.25 (0.00,0.51) | 5.51e-01 | 1.09e-01 |
| Religious Criticism | 0.06 (0.05,0.08) | 0.05 (0.05,0.06) | 0.22 (0.00,0.45) | 1.34e-02 | 2.68e-02 |
| Evangelism | 0.33 (0.31,0.35) | 0.36 (0.34,0.38) | 0.51 (0.26,0.76) | 3.43e-02 | 1.69e-03 |
| *Family Factors* | | | | | |
| Ever Married | 0.52 (0.51,0.54) | 0.55 (0.54,0.57) | 0.19 (0.00,0.40) | 2.43e-01 | 3.85e-05 |
| Divorced | 0.04 (0.04,0.05) | 0.07 (0.06,0.07) | 0.00 * | 2.97e-08 | 1.6e-16 |
| Children | 0.56 (0.53,0.60) | 0.53 (0.51,0.56) | 0.68 (0.29,1.07) | 2.09e-02 | 2.71e-01 |

**Table S19a. Nationally representative descriptive statistics for Tanzania**

| Characteristic | N = 9,075[1] |
|---|---|
| **Age group** | |
| 18-24 | 2,284 (25%) |
| 25-29 | 1,349 (15%) |
| 30-39 | 2,060 (23%) |
| 40-49 | 1,503 (17%) |
| 50-59 | 912 (10%) |
| 60-69 | 575 (6.3%) |
| 70-79 | 297 (3.3%) |
| 80 or older | 93 (1.0%) |
| (Missing) | 2 (<0.1%) |
| **Gender** | |
| Male | 4,299 (47%) |
| Female | 4,776 (53%) |
| Other | 0 (0%) |
| (Missing) | 0 (0%) |
| **Marital status** | |
| Married | 5,577 (61%) |
| Separated | 404 (4.5%) |
| Divorced | 103 (1.1%) |
| Widowed | 450 (5.0%) |
| Single, never married | 2,260 (25%) |
| Domestic Partner | 275 (3.0%) |
| (Missing) | 7 (<0.1%) |
| **Employment** | |
| Employed for an employer | 513 (5.6%) |
| Self-employed | 4,625 (51%) |
| Retired | 139 (1.5%) |
| Student | 319 (3.5%) |
| Homemaker | 1,796 (20%) |
| Unemployed and looking for a job | 1,491 (16%) |
| None of these/Other | 186 (2.1%) |
| (Missing) | 6 (<0.1%) |
| **Religious service attendance** | |
| More than 1/week | 2,622 (29%) |
| 1/week | 4,268 (47%) |
| 1-3/month | 1,082 (12%) |
| A few times a year | 814 (9.0%) |
| Never | 288 (3.2%) |
| (Missing) | 1 (<0.1%) |
| **Education** | |
| Up to 8 years | 6,699 (74%) |
| 9-15 years | 2,252 (25%) |
| 16+ years | 122 (1.3%) |
| (Missing) | 2 (<0.1%) |
| **Immigration** | |
| Born in this country | 9,048 (100%) |
| Born in another country | 25 (0.3%) |
| (Missing) | 1 (<0.1%) |
| **Religious affiliation** | |
| Christianity | 5,647 (62%) |
| Islam | 3,189 (35%) |
| Hinduism | 0 (0%) |
| Buddhism | 0 (0%) |
| Judaism | 0 (0%) |
| Sikhism | 0 (0%) |
| Baha'i | 0 (0%) |
| Jainism | 0 (0%) |
| Shinto | 0 (0%) |
| Taoism | 1 (<0.1%) |
| Confucianism | 0 (0%) |
| Primal, Animist, or Folk religion | 12 (0.1%) |
| Spiritism | 0 (0%) |
| Umbanda, Candomble, and other African-derived religions | 0 (0%) |
| Chinese folk/traditional religion | 0 (0%) |
| Some other religion | 0 (0%) |
| No religion/Atheist/Agnostic | 216 (2.4%) |
| (Missing) | 10 (0.1%) |
| **Race/Ethnicity** | |

| Characteristic | N = 9,075[1] |
|---|---|
| (Missing) | 2 (<0.1%) |
| African | 9,060 (100%) |
| Arab | 11 (0.1%) |
| Indian | 3 (<0.1%) |

[1]n (%)

**Table S19b. Descriptive statistics of outcome variables for Tanzania**

| Characteristic | N = 9,075[1] |
|---|---|
| **Flourishing Index** | 7.49 (1.81) |
| (Missing) | 221 |
| **Secure Flourishing Index** | 7.20 (1.82) |
| (Missing) | 236 |
| **Happiness & Life Satisfaction** | 6.0 (3.0) |
| (Missing) | 22 |
| **Social Relationship Quality** | 7.56 (2.76) |
| (Missing) | 37 |
| **Meaning and Purpose** | 7.67 (2.52) |
| (Missing) | 114 |
| **Character & Virtue** | 7.84 (2.43) |
| (Missing) | 44 |
| **Self-Rated Health** | 8.39 (2.16) |
| (Missing) | 40 |
| **Financial and Material Worry** | 5.8 (3.3) |
| (Missing) | 21 |
| **Happiness** | 6.6 (3.3) |
| (Missing) | 13 |
| **Life Satisfaction** | 5.3 (3.8) |
| (Missing) | 10 |
| **Present Life Evaluation** | 4.4 (3.3) |
| (Missing) | 17 |
| **Future Life Evaluation** | 6.66 (3.04) |
| (Missing) | 601 |
| **Optimism** | 8.75 (2.36) |
| (Missing) | 16 |
| **Freedom** | 8.45 (2.58) |
| (Missing) | 10 |
| **Peace** | |
| Always | 2,879 (32%) |
| Often | 2,694 (30%) |
| Rarely | 3,291 (36%) |
| Never | 202 (2.2%) |
| (Missing) | 9 (0.1%) |
| **Balance in Life** | |
| Always | 1,759 (19%) |
| Often | 2,213 (24%) |
| Rarely | 4,678 (52%) |
| Never | 393 (4.3%) |
| (Missing) | 33 (0.4%) |
| **Mastery** | |
| Always | 3,267 (36%) |
| Often | 2,678 (30%) |
| Rarely | 2,936 (32%) |
| Never | 171 (1.9%) |
| (Missing) | 22 (0.2%) |
| **Meaning** | 7.3 (3.2) |
| (Missing) | 27 |
| **Purpose** | 8.0 (3.0) |
| (Missing) | 93 |
| **Self-Rated Mental Health** | 8.99 (2.08) |
| (Missing) | 30 |
| **Content with My Relationships** | 7.8 (3.0) |
| (Missing) | 17 |
| **Satisfying Relationships** | 7.3 (3.3) |
| (Missing) | 22 |
| **Social Support** | 7.4 (3.3) |
| (Missing) | 6 |
| **Intimate Friend** | |
| Yes | 6,809 (75%) |
| No | 2,256 (25%) |
| (Missing) | 10 (0.1%) |
| **Government Approval** | |
| Strongly approve | 4,557 (50%) |
| Somewhat approve | 3,419 (38%) |
| Neither approve nor disapprove | 400 (4.4%) |
| Somewhat disapprove | 283 (3.1%) |
| Strongly disapprove | 367 (4.0%) |

| Characteristic | N = 9,075[1] |
| --- | --- |
| (Missing) | 50 (0.5%) |
| **Political Voice** | |
| Agree | 3,791 (42%) |
| Disagree | 1,699 (19%) |
| Unsure | 3,561 (39%) |
| (Missing) | 23 (0.3%) |
| **Belonging** | 8.23 (2.76) |
| (Missing) | 41 |
| **City Satisfaction** | |
| Satisfied | 7,385 (81%) |
| Dissatisfied | 1,218 (13%) |
| Unsure | 471 (5.2%) |
| (Missing) | 2 (<0.1%) |
| **Trust** | |
| All | 596 (6.6%) |
| Most | 638 (7.0%) |
| Some | 5,380 (59%) |
| Not very many | 1,955 (22%) |
| None | 384 (4.2%) |
| (Missing) | 122 (1.3%) |
| **Community Participation** | |
| More than once a week | 493 (5.4%) |
| Once a week | 1,363 (15%) |
| One to three times a month | 1,298 (14%) |
| A few times a year | 2,259 (25%) |
| Never | 3,639 (40%) |
| (Missing) | 23 (0.3%) |
| **Traumatic Distress** | |
| A lot | 943 (10%) |
| Some | 2,293 (25%) |
| Not very much | 1,246 (14%) |
| None at all | 4,586 (51%) |
| (Missing) | 7 (<0.1%) |
| **Suffering** | |
| A lot | 1,107 (12%) |
| Some | 3,831 (42%) |
| Not very much | 1,611 (18%) |
| None at all | 2,512 (28%) |
| (Missing) | 14 (0.2%) |
| **Loneliness** | 3.4 (3.5) |
| (Missing) | 18 |
| **Discrimination** | |
| Always | 443 (4.9%) |
| Often | 790 (8.7%) |
| Rarely | 3,244 (36%) |
| Never | 4,576 (50%) |
| (Missing) | 22 (0.2%) |
| **Promoting Good** | 8.18 (2.69) |
| (Missing) | 26 |
| **Delayed Gratification** | 7.5 (3.2) |
| (Missing) | 27 |
| **Hope** | 8.35 (2.65) |
| (Missing) | 33 |
| **Gratitude** | 7.85 (2.88) |
| (Missing) | 52 |
| **Love** | 8.72 (2.32) |
| (Missing) | 22 |
| **Forgiveness** | |
| Always | 4,636 (51%) |
| Often | 2,967 (33%) |
| Rarely | 1,291 (14%) |
| Never | 165 (1.8%) |
| (Missing) | 16 (0.2%) |
| **Charitable Giving** | |
| Yes | 2,470 (27%) |
| No | 6,596 (73%) |
| (Missing) | 8 (<0.1%) |
| **Helping** | |
| Yes | 3,054 (34%) |

| Characteristic | N = 9,075[1] |
|---|---|
| No | 5,989 (66%) |
| (Missing) | 31 (0.3%) |
| **Volunteering** | |
| Yes | 1,006 (11%) |
| No | 8,066 (89%) |
| (Missing) | 4 (<0.1%) |
| **Self-Rated Physical Health** | 7.8 (3.0) |
| (Missing) | 13 |
| **Health Limitations** | |
| Yes | 1,440 (16%) |
| No | 7,626 (84%) |
| (Missing) | 9 (0.1%) |
| **Pain** | |
| A lot | 1,128 (12%) |
| Some | 3,585 (40%) |
| Not very much | 1,724 (19%) |
| None at all | 2,634 (29%) |
| (Missing) | 5 (<0.1%) |
| **Smoking** | 0.2358 (1.8192) |
| (Missing) | 193 |
| **Drinking** | 0.48 (4.06) |
| (Missing) | 196 |
| **Exercise** | |
| 0 days | 2,419 (27%) |
| 1 day | 686 (7.6%) |
| 2 days | 944 (10%) |
| 3 days | 1,030 (11%) |
| 4 days | 627 (6.9%) |
| 5 days | 610 (6.7%) |
| 6 days | 543 (6.0%) |
| 7 days/Every day | 2,115 (23%) |
| (Missing) | 101 (1.1%) |
| **Financial Stability** | 5.7 (3.7) |
| (Missing) | 15 |
| **Material Stability** | 5.9 (3.7) |
| (Missing) | 12 |
| **Education** | |
| Up to 8 years | 6,699 (74%) |
| 9-15 years | 2,252 (25%) |
| 16+ years | 122 (1.3%) |
| (Missing) | 2 (<0.1%) |
| **Employment** | |
| Employed for an employer | 513 (5.6%) |
| Self-employed | 4,625 (51%) |
| Retired | 139 (1.5%) |
| Student | 319 (3.5%) |
| Homemaker | 1,796 (20%) |
| Unemployed and looking for a job | 1,491 (16%) |
| None of these/Other | 186 (2.1%) |
| (Missing) | 6 (<0.1%) |
| **Subjective Financial Well-Being** | |
| Living comfortably on present income | 601 (6.6%) |
| Getting by on present income | 3,889 (43%) |
| Finding it difficult on present income | 2,257 (25%) |
| Finding it very difficult on present income | 2,258 (25%) |
| (Missing) | 70 (0.8%) |
| **Housing** | |
| Someone in this household OWNS this home | 6,467 (71%) |
| Someone in this household RENTS this home | 2,135 (24%) |
| Both | 67 (0.7%) |
| Neither | 398 (4.4%) |
| Rent | 0 (0%) |
| Own | 0 (0%) |
| Something else | 0 (0%) |
| (Missing) | 7 (<0.1%) |
| **Self-Reported Religion/Spirituality** | |
| Always | 4,814 (53%) |
| Often | 2,561 (28%) |
| Rarely | 1,535 (17%) |

| Characteristic | N = 9,075[1] |
|---|---|
| Never | 157 (1.7%) |
| (Missing) | 8 (<0.1%) |
| **Religious Service Attendance** | |
| More than once a week | 2,622 (29%) |
| Once a week | 4,268 (47%) |
| One to three times a month | 1,082 (12%) |
| A few times a year | 814 (9.0%) |
| Never | 288 (3.2%) |
| (Missing) | 1 (<0.1%) |
| **Life after Death Belief** | |
| Yes | 5,511 (61%) |
| No | 1,963 (22%) |
| Unsure | 1,586 (17%) |
| (Missing) | 16 (0.2%) |
| **Religious Experience** | |
| Yes | 6,246 (69%) |
| No | 2,797 (31%) |
| (Missing) | 32 (0.3%) |
| **Religious Reading** | |
| More than once a day | 2,320 (26%) |
| About once a day | 3,996 (44%) |
| Sometimes | 2,281 (25%) |
| Never | 463 (5.1%) |
| (Missing) | 16 (0.2%) |
| **Prayer-Meditation** | |
| More than once a day | 3,138 (35%) |
| About once a day | 3,748 (41%) |
| Sometimes | 1,857 (20%) |
| Never | 323 (3.6%) |
| (Missing) | 9 (<0.1%) |
| **Belief in God** | |
| One God | 8,632 (95%) |
| More than one god | 179 (2.0%) |
| An impersonal spiritual force | 99 (1.1%) |
| None of these | 34 (0.4%) |
| Unsure | 127 (1.4%) |
| (Missing) | 4 (<0.1%) |
| **Intrinsic Religiosity** | |
| Agree | 8,220 (91%) |
| Disagree | 416 (4.6%) |
| Not relevant | 61 (0.7%) |
| Unsure | 377 (4.2%) |
| (Missing) | 2 (<0.1%) |
| **Religious Comfort** | |
| Agree | 8,626 (95%) |
| Disagree | 203 (2.2%) |
| Not relevant | 53 (0.6%) |
| Unsure | 193 (2.1%) |
| (Missing) | 0 (<0.1%) |
| **Loved by God** | |
| Agree | 8,826 (97%) |
| Disagree | 119 (1.3%) |
| Not relevant | 12 (0.1%) |
| Unsure | 113 (1.2%) |
| (Missing) | 6 (<0.1%) |
| **Spiritual Punishment** | |
| Agree | 3,149 (35%) |
| Disagree | 4,197 (46%) |
| Not relevant | 272 (3.0%) |
| Unsure | 1,426 (16%) |
| (Missing) | 31 (0.3%) |
| **Religious Criticism** | |
| Agree | 4,184 (46%) |
| Disagree | 3,436 (38%) |
| Not relevant | 237 (2.6%) |
| Unsure | 1,207 (13%) |
| (Missing) | 12 (0.1%) |
| **Evangelism** | |
| Agree | 7,553 (83%) |

| Characteristic | N = 9,075[1] |
|---|---|
| Disagree | 958 (11%) |
| Not relevant | 226 (2.5%) |
| Unsure | 335 (3.7%) |
| (Missing) | 4 (<0.1%) |
| **Children** | 2.64 (2.04) |
| (Missing) | 1 |

[1]Mean (SD); n (%)

**Table S19c. Demographic variation across outcomes for Tanzania**

| Outcome | Male | Female | Other | Male vs Female p-value | Global p-value |
|---|---|---|---|---|---|
| *Flourishing Index and Domains* | | | | | |
| Flourishing Index | 7.46 (7.36,7.56) | 7.49 (7.39,7.60) | 7.47 (7.36,7.57) | 8.69e-01 | 5.38e-01 |
| Secure Flourishing Index | 7.18 (7.08,7.28) | 7.20 (7.10,7.31) | 7.18 (7.07,7.28) | 8.81e-01 | 6.28e-01 |
| Happiness & Life Satisfaction | 5.71 (5.55,5.86) | 6.18 (6.03,6.33) | 5.92 (5.77,6.08) | 2.24e-04 | 6.58e-09 |
| Social Relationship Quality | 7.55 (7.42,7.68) | 7.55 (7.42,7.68) | 7.73 (7.60,7.86) | 3.04e-01 | 9.86e-01 |
| Meaning and Purpose | 7.63 (7.50,7.76) | 7.69 (7.56,7.81) | 7.58 (7.46,7.71) | 9.1e-01 | 4.26e-01 |
| Character & Virtue | 7.92 (7.78,8.06) | 7.77 (7.63,7.90) | 7.76 (7.62,7.90) | 1.45e-01 | 2.83e-02 |
| Self-Rated Health | 8.50 (8.39,8.61) | 8.28 (8.17,8.39) | 8.34 (8.23,8.44) | 7.27e-05 | 1.35e-03 |
| Financial and Material Worry | 5.76 (5.59,5.93) | 5.75 (5.60,5.91) | 5.74 (5.56,5.91) | 3.1e-01 | 9.19e-01 |
| *Psychological Well-Being* | | | | | |
| Happiness | 6.49 (6.34,6.64) | 6.65 (6.51,6.80) | 6.50 (6.35,6.65) | 2.12e-01 | 6e-02 |
| Life Satisfaction | 4.92 (4.73,5.12) | 5.70 (5.52,5.88) | 5.34 (5.16,5.53) | 8.67e-07 | 2.11e-14 |
| Present Life Evaluation | 4.28 (4.10,4.47) | 4.51 (4.33,4.69) | 4.34 (4.15,4.53) | 1.85e-01 | 8.61e-03 |
| Future Life Evaluation | 6.58 (6.42,6.74) | 6.65 (6.49,6.80) | 6.50 (6.34,6.66) | 3.27e-01 | 4.42e-01 |
| Optimism | 8.76 (8.65,8.88) | 8.74 (8.62,8.85) | 8.68 (8.56,8.79) | 7.85e-01 | 6.82e-01 |
| Freedom | 8.55 (8.43,8.67) | 8.36 (8.24,8.48) | 8.45 (8.34,8.56) | 1.25e-02 | 7.78e-03 |
| Peace | 0.63 (0.61,0.66) | 0.60 (0.57,0.62) | 0.62 (0.59,0.64) | 2.69e-02 | 1.64e-03 |
| Balance in Life | 0.45 (0.43,0.47) | 0.43 (0.40,0.45) | 0.44 (0.42,0.46) | 5.36e-01 | 1.02e-01 |
| Mastery | 0.67 (0.65,0.70) | 0.64 (0.62,0.66) | 0.64 (0.62,0.66) | 8.02e-03 | 6.13e-03 |
| Meaning | 7.22 (7.06,7.37) | 7.38 (7.22,7.53) | 7.23 (7.08,7.38) | 7.58e-01 | 6.75e-02 |
| Purpose | 8.04 (7.89,8.19) | 7.99 (7.85,8.13) | 7.94 (7.80,8.08) | 8.87e-01 | 5.81e-01 |
| Self-Rated Mental Health | 9.01 (8.91,9.12) | 8.97 (8.87,9.08) | 8.97 (8.87,9.06) | 4.06e-02 | 4.55e-01 |
| *Social Well-Being* | | | | | |
| Content with My Relationships | 7.76 (7.62,7.90) | 7.75 (7.60,7.90) | 7.86 (7.72,8.00) | 5.23e-01 | 8.69e-01 |
| Satisfying Relationships | 7.34 (7.19,7.49) | 7.36 (7.22,7.50) | 7.60 (7.46,7.73) | 2.37e-01 | 8.42e-01 |
| Social Support | 7.24 (7.10,7.39) | 7.51 (7.37,7.64) | 7.33 (7.19,7.48) | 6.75e-02 | 1.01e-03 |
| Intimate Friend | 0.78 (0.76,0.80) | 0.73 (0.71,0.75) | 0.76 (0.74,0.78) | 7.06e-03 | 1.01e-04 |
| Government Approval | 0.87 (0.86,0.89) | 0.89 (0.88,0.91) | 0.88 (0.87,0.90) | 3.14e-01 | 4.55e-02 |
| Political Voice | 0.67 (0.64,0.69) | 0.68 (0.65,0.70) | 0.67 (0.65,0.70) | 5.34e-01 | 3.41e-01 |
| Belonging | 8.25 (8.11,8.39) | 8.21 (8.07,8.34) | 8.21 (8.09,8.34) | 2.8e-01 | 6.21e-01 |
| City Satisfaction | 0.82 (0.81,0.84) | 0.89 (0.87,0.90) | 0.87 (0.85,0.88) | 8.38e-05 | 1.68e-10 |
| Trust | 0.14 (0.13,0.16) | 0.13 (0.12,0.15) | 0.14 (0.12,0.16) | 7.21e-01 | 4.29e-01 |
| Community Participation | 0.24 (0.22,0.26) | 0.17 (0.15,0.19) | 0.21 (0.19,0.23) | 7.66e-09 | 9.21e-09 |
| *Psychological Distress* | | | | | |
| Traumatic Distress | 0.37 (0.34,0.39) | 0.35 (0.33,0.37) | 0.36 (0.34,0.38) | 1.29e-01 | 1.88e-01 |
| Depression Symptoms | 0.47 (0.44,0.49) | 0.44 (0.42,0.47) | 0.46 (0.44,0.49) | 3.06e-01 | 1.22e-01 |
| Anxiety Symptoms | 0.32 (0.29,0.35) | 0.32 (0.30,0.35) | 0.32 (0.30,0.35) | 3.3e-01 | 9.11e-01 |
| Suffering | 0.57 (0.54,0.59) | 0.53 (0.50,0.55) | 0.55 (0.53,0.58) | 1.99e-02 | 1.53e-03 |
| *Social Distress* | | | | | |
| Loneliness | 3.29 (3.14,3.45) | 3.44 (3.27,3.60) | 3.19 (3.04,3.34) | 3.88e-02 | 1.33e-01 |
| Discrimination | 0.15 (0.13,0.16) | 0.13 (0.11,0.14) | 0.14 (0.12,0.15) | 6.07e-01 | 5.76e-02 |
| *Character & Prosocial Behavior* | | | | | |
| Promoting Good | 8.25 (8.12,8.38) | 8.12 (7.98,8.25) | 8.09 (7.96,8.23) | 1.77e-01 | 1.05e-01 |
| Delayed Gratification | 7.60 (7.41,7.79) | 7.41 (7.25,7.57) | 7.42 (7.25,7.60) | 2.59e-01 | 3.83e-02 |
| Hope | 8.37 (8.23,8.50) | 8.34 (8.22,8.47) | 8.33 (8.21,8.46) | 2.02e-01 | 7.84e-01 |
| Gratitude | 7.82 (7.64,8.00) | 7.88 (7.74,8.02) | 7.81 (7.64,7.98) | 4.86e-01 | 4.67e-01 |
| Love | 8.73 (8.60,8.85) | 8.70 (8.58,8.83) | 8.68 (8.56,8.80) | 3.77e-01 | 7.15e-01 |
| Forgiveness | 0.85 (0.83,0.86) | 0.83 (0.82,0.85) | 0.84 (0.82,0.85) | 5.45e-01 | 1.53e-01 |
| Charitable Giving | 0.34 (0.32,0.36) | 0.21 (0.20,0.23) | 0.27 (0.25,0.28) | 3.33e-15 | 1.6e-16 |
| Helping | 0.37 (0.35,0.40) | 0.30 (0.29,0.32) | 0.34 (0.32,0.36) | 7.22e-05 | 1.25e-07 |
| Volunteering | 0.14 (0.12,0.15) | 0.09 (0.07,0.10) | 0.11 (0.10,0.13) | 2.51e-06 | 3.67e-09 |
| *Physical Health & Health Behavior* | | | | | |
| Self-Rated Physical Health | 7.98 (7.84,8.12) | 7.60 (7.45,7.74) | 7.71 (7.57,7.84) | 1.28e-05 | 2.11e-05 |
| Health Limitations | 0.12 (0.11,0.14) | 0.19 (0.17,0.21) | 0.16 (0.15,0.18) | 5.92e-07 | 1.69e-09 |
| Pain | 0.49 (0.47,0.52) | 0.54 (0.52,0.57) | 0.52 (0.50,0.55) | 2.93e-03 | 9.56e-04 |
| Smoking | 0.44 (0.37,0.52) | 0.03 (0.01,0.05) | 0.24 (0.19,0.30) | 1.38e-11 | 1.6e-16 |
| Drinking | 0.62 (0.52,0.72) | 0.20 (0.14,0.26) | 0.38 (0.31,0.44) | 1.5e-08 | <2e-16 |
| Exercise | 3.97 (3.83,4.12) | 2.62 (2.46,2.77) | 3.40 (3.24,3.55) | 1.6e-16 | 1.6e-16 |
| *Socioeconomic Outcomes* | | | | | |
| Financial Stability | 5.65 (5.46,5.83) | 5.69 (5.52,5.86) | 5.63 (5.44,5.82) | 8.91e-01 | 6.92e-01 |
| Material Stability | 5.88 (5.69,6.07) | 5.82 (5.65,5.99) | 5.84 (5.66,6.01) | 1.05e-01 | 5.57e-01 |
| Education | 0.02 (0.01,0.02) | 0.01 (0.01,0.01) | 0.01 (0.01,0.01) | 3.46e-05 | 1.02e-05 |
| Employment | 0.72 (0.70,0.74) | 0.43 (0.40,0.46) | 0.62 (0.59,0.64) | 1.6e-16 | 1.6e-16 |
| Subjective Financial Well-Being | 0.52 (0.49,0.54) | 0.48 (0.45,0.50) | 0.49 (0.47,0.52) | 1.48e-02 | 2.93e-03 |
| Housing | 0.73 (0.70,0.75) | 0.71 (0.69,0.74) | 0.77 (0.75,0.79) | 2.28e-01 | 3.09e-01 |
| *Religion/Spirituality* | | | | | |
| Self-Reported Religion/Spirituality | 0.79 (0.77,0.81) | 0.83 (0.82,0.85) | 0.81 (0.79,0.83) | 9.54e-03 | 1.33e-05 |

| Outcome | Male | Female | Other | Male vs Female p-value | Global p-value |
|---|---|---|---|---|---|
| Religious Service Attendance | 0.73 (0.71,0.75) | 0.79 (0.77,0.80) | 0.77 (0.76,0.79) | 1.26e-03 | 4.04e-07 |
| Life after Death Belief | 0.64 (0.61,0.66) | 0.58 (0.56,0.61) | 0.63 (0.61,0.65) | 9.4e-03 | 3.1e-05 |
| Religious Experience | 0.68 (0.65,0.70) | 0.70 (0.68,0.73) | 0.69 (0.67,0.71) | 2.41e-01 | 1.7e-02 |
| Religious Reading | 0.69 (0.67,0.71) | 0.70 (0.68,0.73) | 0.71 (0.69,0.74) | 8.81e-01 | 2.38e-01 |
| Prayer-Meditation | 0.74 (0.71,0.76) | 0.78 (0.76,0.80) | 0.76 (0.74,0.79) | 2.24e-02 | 1.27e-04 |
| Belief in God | 0.98 (0.98,0.99) | 0.98 (0.97,0.99) | 0.99 (0.98,0.99) | 9.45e-01 | 3.78e-01 |
| Intrinsic Religiosity | 0.94 (0.93,0.96) | 0.96 (0.95,0.97) | 0.95 (0.94,0.96) | 1.63e-01 | 6.98e-03 |
| Religious Comfort | 0.97 (0.97,0.98) | 0.98 (0.97,0.98) | 0.97 (0.96,0.98) | 5.19e-01 | 2.53e-01 |
| Loved by God | 0.98 (0.98,0.99) | 0.99 (0.98,0.99) | 0.99 (0.98,0.99) | 8.56e-01 | 1.63e-01 |
| Spiritual Punishment | 0.43 (0.41,0.46) | 0.43 (0.40,0.45) | 0.44 (0.41,0.46) | 6.22e-01 | 5.46e-01 |
| Religious Criticism | 0.55 (0.52,0.58) | 0.55 (0.52,0.58) | 0.56 (0.54,0.59) | 3.88e-01 | 7.49e-01 |
| Evangelism | 0.88 (0.87,0.90) | 0.89 (0.87,0.90) | 0.89 (0.87,0.90) | 5.19e-01 | 5.2e-01 |
| *Family Factors* | | | | | |
| Ever Married | 0.68 (0.66,0.70) | 0.76 (0.74,0.77) | 1.00 (1.00,1.00) | 6.16e-05 | 1.93e-08 |
| Divorced | 0.01 (0.00,0.01) | 0.02 (0.01,0.02) | 0.01 (0.00,0.01) | 4.16e-04 | 2.53e-04 |
| Children | 2.59 (2.48,2.70) | 2.69 (2.59,2.80) | 2.87 (2.77,2.97) | 6.43e-01 | 7.76e-02 |

**Table S20a. Nationally representative descriptive statistics for Turkey**

| Characteristic | N = 1,473[1] |
|---|---|
| **Age group** | |
| 18-24 | 222 (15%) |
| 25-29 | 152 (10%) |
| 30-39 | 315 (21%) |
| 40-49 | 312 (21%) |
| 50-59 | 225 (15%) |
| 60-69 | 164 (11%) |
| 70-79 | 65 (4.4%) |
| 80 or older | 18 (1.2%) |
| (Missing) | 0 (0%) |
| **Gender** | |
| Male | 754 (51%) |
| Female | 719 (49%) |
| Other | 0 (0%) |
| (Missing) | 0 (0%) |
| **Marital status** | |
| Married | 936 (64%) |
| Separated | 13 (0.9%) |
| Divorced | 64 (4.3%) |
| Widowed | 64 (4.3%) |
| Single, never married | 379 (26%) |
| Domestic Partner | 0 (0%) |
| (Missing) | 17 (1.1%) |
| **Employment** | |
| Employed for an employer | 413 (28%) |
| Self-employed | 255 (17%) |
| Retired | 205 (14%) |
| Student | 107 (7.3%) |
| Homemaker | 347 (24%) |
| Unemployed and looking for a job | 87 (5.9%) |
| None of these/Other | 59 (4.0%) |
| (Missing) | 0 (0%) |
| **Religious service attendance** | |
| More than 1/week | 493 (33%) |
| 1/week | 271 (18%) |
| 1-3/month | 174 (12%) |
| A few times a year | 255 (17%) |
| Never | 274 (19%) |
| (Missing) | 6 (0.4%) |
| **Education** | |
| Up to 8 years | 436 (30%) |
| 9-15 years | 711 (48%) |
| 16+ years | 326 (22%) |
| (Missing) | 0 (0%) |
| **Immigration** | |
| Born in this country | 1,415 (96%) |
| Born in another country | 58 (4.0%) |
| (Missing) | 0 (0%) |
| **Religious affiliation** | |
| Christianity | 2 (0.1%) |
| Islam | 1,381 (94%) |
| Hinduism | 0 (0%) |
| Buddhism | 1 (<0.1%) |
| Judaism | 1 (<0.1%) |
| Sikhism | 1 (<0.1%) |
| Baha'i | 0 (0%) |
| Jainism | 0 (0%) |
| Shinto | 0 (0%) |
| Taoism | 0 (0%) |
| Confucianism | 0 (0%) |
| Primal, Animist, or Folk religion | 1 (<0.1%) |
| Spiritism | 0 (0%) |
| Umbanda, Candomble, and other African-derived religions | 0 (0%) |
| Chinese folk/traditional religion | 0 (0%) |
| Some other religion | 1 (<0.1%) |
| No religion/Atheist/Agnostic | 66 (4.5%) |
| (Missing) | 19 (1.3%) |
| **Race/Ethnicity** | |

| Characteristic | N = 1,473[1] |
|---|---|
| (Missing) | 9 (0.6%) |
| Albanian | 8 (0.5%) |
| Arab | 51 (3.5%) |
| Armenian | 1 (<0.1%) |
| Azeri | 9 (0.6%) |
| Bosnian | 5 (0.3%) |
| Circassian | 19 (1.3%) |
| Georgian | 4 (0.3%) |
| Greek | 1 (<0.1%) |
| Kurdish/Zaza | 252 (17%) |
| Laz | 25 (1.7%) |
| Other | 58 (3.9%) |
| Turkish | 1,030 (70%) |
| Uyghur | 1 (<0.1%) |

[1]n (%)

*Table S20b. Descriptive statistics of outcome variables for Turkey*

| Characteristic | N = 1,473[1] |
|---|---|
| **Flourishing Index** | 6.58 (1.94) |
| (Missing) | 25 |
| **Secure Flourishing Index** | 6.31 (1.97) |
| (Missing) | 28 |
| **Happiness & Life Satisfaction** | 5.4 (2.9) |
| (Missing) | 6 |
| **Social Relationship Quality** | 6.99 (2.64) |
| (Missing) | 6 |
| **Meaning and Purpose** | 6.67 (2.62) |
| (Missing) | 11 |
| **Character & Virtue** | 7.43 (2.15) |
| **Self-Rated Health** | 6.49 (2.50) |
| (Missing) | 2 |
| **Financial and Material Worry** | 5.0 (3.3) |
| (Missing) | 3 |
| **Happiness** | 5.5 (2.9) |
| **Life Satisfaction** | 5.2 (3.2) |
| (Missing) | 5 |
| **Present Life Evaluation** | 5.18 (2.56) |
| (Missing) | 3 |
| **Future Life Evaluation** | 6.20 (2.96) |
| (Missing) | 100 |
| **Optimism** | 7.65 (2.50) |
| (Missing) | 5 |
| **Freedom** | 7.28 (2.97) |
| (Missing) | 1 |
| **Peace** | |
| Always | 349 (24%) |
| Often | 372 (25%) |
| Rarely | 625 (42%) |
| Never | 118 (8.0%) |
| (Missing) | 8 (0.6%) |
| **Balance in Life** | |
| Always | 293 (20%) |
| Often | 402 (27%) |
| Rarely | 654 (44%) |
| Never | 112 (7.6%) |
| (Missing) | 13 (0.9%) |
| **Mastery** | |
| Always | 627 (43%) |
| Often | 454 (31%) |
| Rarely | 325 (22%) |
| Never | 61 (4.1%) |
| (Missing) | 6 (0.4%) |
| **Meaning** | 6.1 (3.1) |
| (Missing) | 3 |
| **Purpose** | 7.20 (2.97) |
| (Missing) | 8 |
| **Self-Rated Mental Health** | 6.3 (3.0) |
| **Content with My Relationships** | 7.28 (2.90) |
| **Satisfying Relationships** | 6.71 (2.90) |
| (Missing) | 5 |
| **Social Support** | 5.5 (3.5) |
| (Missing) | 1 |
| **Intimate Friend** | |
| Yes | 1,137 (77%) |
| No | 327 (22%) |
| (Missing) | 9 (0.6%) |
| **Government Approval** | |
| Strongly approve | 278 (19%) |
| Somewhat approve | 319 (22%) |
| Neither approve nor disapprove | 188 (13%) |
| Somewhat disapprove | 158 (11%) |
| Strongly disapprove | 495 (34%) |
| (Missing) | 35 (2.4%) |
| **Political Voice** | |
| Agree | 421 (29%) |
| Disagree | 684 (46%) |

| Characteristic | N = 1,473[1] |
|---|---|
| Unsure | 359 (24%) |
| (Missing) | 9 (0.6%) |
| **Belonging** | 6.9 (3.4) |
| (Missing) | 15 |
| **City Satisfaction** | |
| Satisfied | 940 (64%) |
| Dissatisfied | 323 (22%) |
| Unsure | 204 (14%) |
| (Missing) | 6 (0.4%) |
| **Trust** | |
| All | 65 (4.4%) |
| Most | 150 (10%) |
| Some | 317 (22%) |
| Not very many | 510 (35%) |
| None | 413 (28%) |
| (Missing) | 18 (1.2%) |
| **Community Participation** | |
| More than once a week | 165 (11%) |
| Once a week | 138 (9.4%) |
| One to three times a month | 214 (15%) |
| A few times a year | 329 (22%) |
| Never | 618 (42%) |
| (Missing) | 9 (0.6%) |
| **Traumatic Distress** | |
| A lot | 395 (27%) |
| Some | 365 (25%) |
| Not very much | 249 (17%) |
| None at all | 456 (31%) |
| (Missing) | 8 (0.6%) |
| **Suffering** | |
| A lot | 360 (24%) |
| Some | 526 (36%) |
| Not very much | 336 (23%) |
| None at all | 244 (17%) |
| (Missing) | 7 (0.5%) |
| **Loneliness** | 4.6 (3.4) |
| (Missing) | 7 |
| **Discrimination** | |
| Always | 200 (14%) |
| Often | 222 (15%) |
| Rarely | 637 (43%) |
| Never | 405 (27%) |
| (Missing) | 10 (0.6%) |
| **Promoting Good** | 7.69 (2.37) |
| **Delayed Gratification** | 7.18 (2.65) |
| **Hope** | 7.76 (2.68) |
| (Missing) | 11 |
| **Gratitude** | 6.95 (2.73) |
| (Missing) | 62 |
| **Love** | 7.89 (2.62) |
| **Forgiveness** | |
| Always | 311 (21%) |
| Often | 292 (20%) |
| Rarely | 513 (35%) |
| Never | 345 (23%) |
| (Missing) | 11 (0.8%) |
| **Charitable Giving** | |
| Yes | 453 (31%) |
| No | 1,015 (69%) |
| (Missing) | 4 (0.3%) |
| **Helping** | |
| Yes | 883 (60%) |
| No | 571 (39%) |
| (Missing) | 19 (1.3%) |
| **Volunteering** | |
| Yes | 219 (15%) |
| No | 1,254 (85%) |
| (Missing) | 0 (0%) |
| **Self-Rated Physical Health** | 6.70 (2.72) |

| Characteristic | N = 1,473[1] |
|---|---|
| (Missing) | 2 |
| **Health Limitations** | |
| Yes | 199 (14%) |
| No | 1,256 (85%) |
| (Missing) | 18 (1.2%) |
| **Pain** | |
| A lot | 351 (24%) |
| Some | 420 (29%) |
| Not very much | 323 (22%) |
| None at all | 373 (25%) |
| (Missing) | 5 (0.3%) |
| **Smoking** | 10 (13) |
| (Missing) | 10 |
| **Drinking** | 1.16 (5.20) |
| (Missing) | 20 |
| **Exercise** | |
| 0 days | 537 (36%) |
| 1 day | 101 (6.8%) |
| 2 days | 129 (8.7%) |
| 3 days | 176 (12%) |
| 4 days | 86 (5.8%) |
| 5 days | 58 (3.9%) |
| 6 days | 32 (2.2%) |
| 7 days/Every day | 338 (23%) |
| (Missing) | 17 (1.2%) |
| **Financial Stability** | 4.6 (3.5) |
| (Missing) | 2 |
| **Material Stability** | 5.3 (3.6) |
| (Missing) | 2 |
| **Education** | |
| Up to 8 years | 436 (30%) |
| 9-15 years | 711 (48%) |
| 16+ years | 326 (22%) |
| (Missing) | 0 (0%) |
| **Employment** | |
| Employed for an employer | 413 (28%) |
| Self-employed | 255 (17%) |
| Retired | 205 (14%) |
| Student | 107 (7.3%) |
| Homemaker | 347 (24%) |
| Unemployed and looking for a job | 87 (5.9%) |
| None of these/Other | 59 (4.0%) |
| (Missing) | 0 (0%) |
| **Subjective Financial Well-Being** | |
| Living comfortably on present income | 190 (13%) |
| Getting by on present income | 567 (38%) |
| Finding it difficult on present income | 368 (25%) |
| Finding it very difficult on present income | 329 (22%) |
| (Missing) | 19 (1.3%) |
| **Housing** | |
| Someone in this household OWNS this home | 765 (52%) |
| Someone in this household RENTS this home | 442 (30%) |
| Both | 61 (4.1%) |
| Neither | 182 (12%) |
| Rent | 0 (0%) |
| Own | 0 (0%) |
| Something else | 0 (0%) |
| (Missing) | 23 (1.5%) |
| **Self-Reported Religion/Spirituality** | |
| Always | 716 (49%) |
| Often | 220 (15%) |
| Rarely | 323 (22%) |
| Never | 186 (13%) |
| (Missing) | 28 (1.9%) |
| **Religious Service Attendance** | |
| More than once a week | 493 (33%) |
| Once a week | 271 (18%) |
| One to three times a month | 174 (12%) |
| A few times a year | 255 (17%) |

| Characteristic | N = 1,473[1] |
|---|---|
| Never | 274 (19%) |
| (Missing) | 6 (0.4%) |
| **Life after Death Belief** | |
| Yes | 983 (67%) |
| No | 225 (15%) |
| Unsure | 261 (18%) |
| (Missing) | 4 (0.3%) |
| **Religious Experience** | |
| Yes | 428 (29%) |
| No | 1,022 (69%) |
| (Missing) | 23 (1.6%) |
| **Religious Reading** | |
| More than once a day | 278 (19%) |
| About once a day | 286 (19%) |
| Sometimes | 694 (47%) |
| Never | 208 (14%) |
| (Missing) | 7 (0.5%) |
| **Prayer-Meditation** | |
| More than once a day | 760 (52%) |
| About once a day | 254 (17%) |
| Sometimes | 320 (22%) |
| Never | 135 (9.2%) |
| (Missing) | 4 (0.3%) |
| **Belief in God** | |
| One God | 1,212 (82%) |
| More than one god | 14 (1.0%) |
| An impersonal spiritual force | 24 (1.6%) |
| None of these | 139 (9.5%) |
| Unsure | 75 (5.1%) |
| (Missing) | 8 (0.6%) |
| **Intrinsic Religiosity** | |
| Agree | 1,047 (71%) |
| Disagree | 207 (14%) |
| Not relevant | 98 (6.7%) |
| Unsure | 115 (7.8%) |
| (Missing) | 6 (0.4%) |
| **Religious Comfort** | |
| Agree | 1,127 (77%) |
| Disagree | 150 (10%) |
| Not relevant | 91 (6.2%) |
| Unsure | 100 (6.8%) |
| (Missing) | 5 (0.4%) |
| **Loved by God** | |
| Agree | 1,115 (76%) |
| Disagree | 117 (7.9%) |
| Not relevant | 85 (5.8%) |
| Unsure | 146 (9.9%) |
| (Missing) | 10 (0.7%) |
| **Spiritual Punishment** | |
| Agree | 447 (30%) |
| Disagree | 688 (47%) |
| Not relevant | 125 (8.5%) |
| Unsure | 198 (13%) |
| (Missing) | 15 (1.0%) |
| **Religious Criticism** | |
| Agree | 544 (37%) |
| Disagree | 625 (42%) |
| Not relevant | 119 (8.1%) |
| Unsure | 179 (12%) |
| (Missing) | 6 (0.4%) |
| **Evangelism** | |
| Agree | 876 (59%) |
| Disagree | 350 (24%) |
| Not relevant | 109 (7.4%) |
| Unsure | 127 (8.6%) |
| (Missing) | 11 (0.7%) |
| **Children** | |
| 0 | 721 (49%) |
| 1 | 304 (21%) |

| **Characteristic** | **N = 1,473**[1] |
|---|---|
| 2 | 247 (17%) |
| 3 | 133 (9.0%) |
| 4 | 36 (2.4%) |
| 5 | 19 (1.3%) |
| 6 | 3 (0.2%) |
| 7 | 5 (0.3%) |
| 8 | 4 (0.3%) |
| (Missing) | 1 |

[1]Mean (SD); n (%)

**Table S20c. Demographic variation across outcomes for Turkey**

| Outcome | Male | Female | Other | Male vs Female p-value | Global p-value |
|---|---|---|---|---|---|
| *Flourishing Index and Domains* | | | | | |
| Flourishing Index | 6.59 (6.44,6.75) | 6.58 (6.39,6.77) | 6.66 (6.50,6.82) | 8.88e-01 | 8.97e-01 |
| Secure Flourishing Index | 6.36 (6.20,6.52) | 6.27 (6.08,6.47) | 6.37 (6.20,6.53) | 4.03e-01 | 4.93e-01 |
| Happiness & Life Satisfaction | 5.25 (5.01,5.49) | 5.48 (5.19,5.77) | 5.49 (5.24,5.73) | 5.33e-01 | 2.18e-01 |
| Social Relationship Quality | 6.99 (6.78,7.20) | 7.00 (6.74,7.26) | 7.19 (6.98,7.40) | 8.13e-01 | 9.52e-01 |
| Meaning and Purpose | 6.59 (6.38,6.79) | 6.74 (6.47,7.00) | 6.70 (6.48,6.92) | 8.98e-01 | 3.88e-01 |
| Character & Virtue | 7.43 (7.26,7.60) | 7.43 (7.22,7.65) | 7.40 (7.22,7.58) | 6.93e-01 | 9.69e-01 |
| Self-Rated Health | 6.72 (6.52,6.92) | 6.24 (5.99,6.49) | 6.51 (6.30,6.71) | 9.1e-02 | 3.53e-03 |
| Financial and Material Worry | 5.18 (4.92,5.45) | 4.74 (4.43,5.06) | 4.92 (4.65,5.19) | 8.14e-03 | 3.16e-02 |
| *Psychological Well-Being* | | | | | |
| Happiness | 5.42 (5.17,5.67) | 5.66 (5.37,5.95) | 5.65 (5.39,5.90) | 7.81e-01 | 2.15e-01 |
| Life Satisfaction | 5.08 (4.81,5.35) | 5.31 (4.98,5.63) | 5.33 (5.06,5.61) | 3.95e-01 | 2.87e-01 |
| Present Life Evaluation | 5.19 (4.97,5.40) | 5.18 (4.91,5.45) | 5.14 (4.93,5.35) | 8.12e-01 | 9.64e-01 |
| Future Life Evaluation | 6.04 (5.78,6.29) | 6.23 (5.92,6.55) | 5.83 (5.56,6.09) | 8.21e-01 | 3.3e-01 |
| Optimism | 7.36 (7.15,7.56) | 7.97 (7.74,8.19) | 7.75 (7.55,7.94) | 9.8e-03 | 7.25e-05 |
| Freedom | 7.26 (7.02,7.49) | 7.32 (7.03,7.61) | 7.29 (7.04,7.54) | 8.97e-01 | 7.45e-01 |
| Peace | 0.48 (0.44,0.52) | 0.51 (0.46,0.56) | 0.52 (0.48,0.56) | 4.87e-01 | 2.87e-01 |
| Balance in Life | 0.48 (0.44,0.52) | 0.47 (0.42,0.52) | 0.49 (0.45,0.53) | 9.12e-01 | 7.7e-01 |
| Mastery | 0.73 (0.70,0.77) | 0.74 (0.70,0.78) | 0.75 (0.71,0.78) | 3.32e-01 | 7.8e-01 |
| Meaning | 5.97 (5.72,6.22) | 6.31 (6.00,6.61) | 6.19 (5.94,6.44) | 5.11e-01 | 8.97e-02 |
| Purpose | 7.21 (6.97,7.45) | 7.17 (6.86,7.47) | 7.21 (6.96,7.47) | 3.98e-01 | 8.23e-01 |
| Self-Rated Mental Health | 6.44 (6.20,6.67) | 6.10 (5.81,6.40) | 6.38 (6.13,6.62) | 2.69e-02 | 8.06e-02 |
| *Social Well-Being* | | | | | |
| Content with My Relationships | 7.24 (7.01,7.47) | 7.31 (7.02,7.60) | 7.43 (7.19,7.66) | 5.32e-01 | 7.12e-01 |
| Satisfying Relationships | 6.74 (6.51,6.96) | 6.68 (6.39,6.98) | 6.96 (6.73,7.19) | 8.51e-01 | 7.84e-01 |
| Social Support | 5.26 (4.98,5.54) | 5.70 (5.36,6.04) | 5.52 (5.24,5.80) | 8.13e-01 | 4.4e-02 |
| Intimate Friend | 0.73 (0.70,0.77) | 0.82 (0.78,0.86) | 0.81 (0.78,0.84) | 5.07e-02 | 5.4e-04 |
| Government Approval | 0.43 (0.39,0.46) | 0.40 (0.35,0.45) | 0.46 (0.42,0.50) | 5.11e-02 | 4.69e-01 |
| Political Voice | 0.34 (0.30,0.38) | 0.41 (0.36,0.46) | 0.37 (0.33,0.41) | 5.14e-01 | 4.12e-02 |
| Belonging | 6.86 (6.59,7.13) | 6.86 (6.56,7.17) | 7.02 (6.76,7.29) | 5.63e-01 | 9.61e-01 |
| City Satisfaction | 0.68 (0.64,0.71) | 0.78 (0.74,0.81) | 0.76 (0.73,0.80) | 1.89e-02 | 1.73e-04 |
| Trust | 0.17 (0.14,0.20) | 0.12 (0.09,0.16) | 0.16 (0.13,0.19) | 1.32e-01 | 3.76e-02 |
| Community Participation | 0.26 (0.22,0.29) | 0.15 (0.12,0.19) | 0.18 (0.15,0.21) | 6.03e-03 | 2.92e-05 |
| *Psychological Distress* | | | | | |
| Traumatic Distress | 0.48 (0.44,0.52) | 0.56 (0.51,0.61) | 0.52 (0.47,0.56) | 1.62e-01 | 8.85e-03 |
| Depression Symptoms | 0.41 (0.37,0.45) | 0.44 (0.39,0.49) | 0.40 (0.36,0.44) | 8.54e-01 | 3.61e-01 |
| Anxiety Symptoms | 0.41 (0.37,0.45) | 0.43 (0.38,0.47) | 0.39 (0.35,0.43) | 7.18e-01 | 6.76e-01 |
| Suffering | 0.58 (0.54,0.62) | 0.63 (0.59,0.68) | 0.60 (0.56,0.64) | 1.89e-01 | 7.16e-02 |
| *Social Distress* | | | | | |
| Loneliness | 4.48 (4.21,4.76) | 4.63 (4.28,4.97) | 4.29 (4.01,4.57) | 6.45e-02 | 5.16e-01 |
| Discrimination | 0.30 (0.26,0.33) | 0.28 (0.24,0.32) | 0.29 (0.25,0.32) | 5.02e-01 | 5.3e-01 |
| *Character & Prosocial Behavior* | | | | | |
| Promoting Good | 7.64 (7.45,7.84) | 7.74 (7.50,7.97) | 7.68 (7.48,7.89) | 4.82e-01 | 5.56e-01 |
| Delayed Gratification | 7.21 (7.00,7.43) | 7.13 (6.87,7.40) | 7.11 (6.89,7.33) | 9.64e-01 | 6.4e-01 |
| Hope | 7.71 (7.49,7.93) | 7.82 (7.55,8.08) | 7.86 (7.65,8.08) | 5.1e-01 | 5.3e-01 |
| Gratitude | 6.85 (6.64,7.07) | 7.11 (6.83,7.38) | 7.02 (6.79,7.25) | 2.67e-01 | 1.39e-01 |
| Love | 7.64 (7.43,7.86) | 8.14 (7.90,8.38) | 8.02 (7.80,8.23) | 2.34e-02 | 2.12e-03 |
| Forgiveness | 0.44 (0.40,0.48) | 0.39 (0.34,0.43) | 0.44 (0.40,0.48) | 6.21e-01 | 8.7e-02 |
| Charitable Giving | 0.33 (0.30,0.37) | 0.28 (0.24,0.32) | 0.32 (0.28,0.35) | 1.05e-02 | 6.41e-02 |
| Helping | 0.63 (0.59,0.67) | 0.59 (0.54,0.64) | 0.60 (0.56,0.64) | 3.27e-01 | 2.1e-01 |
| Volunteering | 0.16 (0.14,0.19) | 0.13 (0.10,0.16) | 0.12 (0.09,0.14) | 4.02e-01 | 1.31e-01 |
| *Physical Health & Health Behavior* | | | | | |
| Self-Rated Physical Health | 7.00 (6.78,7.22) | 6.38 (6.10,6.66) | 6.63 (6.41,6.86) | 4.98e-01 | 6.67e-04 |
| Health Limitations | 0.12 (0.09,0.15) | 0.16 (0.12,0.19) | 0.13 (0.10,0.16) | 1.42e-01 | 1.26e-01 |
| Pain | 0.48 (0.44,0.52) | 0.57 (0.53,0.62) | 0.55 (0.51,0.59) | 2.89e-01 | 2.83e-03 |
| Smoking | 12.61 (11.54,13.67) | 5.63 (4.83,6.42) | 8.23 (7.40,9.07) | 2.86e-13 | 1.6e-16 |
| Drinking | 1.61 (1.21,2.00) | 0.51 (0.20,0.82) | 0.71 (0.52,0.89) | 4.23e-02 | 1.71e-05 |
| Exercise | 2.85 (2.62,3.09) | 2.76 (2.48,3.04) | 2.70 (2.47,2.94) | 6.96e-01 | 5.97e-01 |
| *Socioeconomic Outcomes* | | | | | |
| Financial Stability | 4.87 (4.58,5.16) | 4.42 (4.09,4.75) | 4.59 (4.30,4.87) | 2.27e-02 | 4.11e-02 |
| Material Stability | 5.49 (5.21,5.78) | 5.07 (4.71,5.43) | 5.26 (4.96,5.56) | 1.01e-02 | 6.1e-02 |
| Education | 0.23 (0.21,0.26) | 0.21 (0.18,0.24) | 0.20 (0.18,0.23) | 4.36e-01 | 2.44e-01 |
| Employment | 0.62 (0.58,0.66) | 0.28 (0.24,0.31) | 0.45 (0.41,0.49) | 1.6e-16 | 1.6e-16 |
| Subjective Financial Well-Being | 0.55 (0.51,0.59) | 0.48 (0.44,0.53) | 0.52 (0.48,0.56) | 5.4e-02 | 2.58e-02 |
| Housing | 0.59 (0.55,0.63) | 0.54 (0.49,0.59) | 0.57 (0.53,0.61) | 2.4e-01 | 1.01e-01 |
| *Religion/Spirituality* | | | | | |
| Self-Reported Religion/Spirituality | 0.63 (0.59,0.66) | 0.67 (0.63,0.72) | 0.68 (0.64,0.71) | 3.51e-01 | 1.14e-01 |

| Outcome | Male | Female | Other | Male vs Female p-value | Global p-value |
|---|---|---|---|---|---|
| Religious Service Attendance | 0.57 (0.53,0.61) | 0.47 (0.42,0.52) | 0.57 (0.53,0.61) | 5.42e-05 | 1.24e-03 |
| Life after Death Belief | 0.66 (0.63,0.70) | 0.67 (0.63,0.72) | 0.70 (0.67,0.74) | 2.86e-01 | 7.74e-01 |
| Religious Experience | 0.30 (0.26,0.34) | 0.29 (0.25,0.33) | 0.29 (0.25,0.32) | 6.54e-01 | 5.71e-01 |
| Religious Reading | 0.33 (0.29,0.36) | 0.44 (0.39,0.49) | 0.41 (0.37,0.45) | 5.46e-02 | 2.36e-04 |
| Prayer-Meditation | 0.62 (0.58,0.66) | 0.76 (0.73,0.80) | 0.75 (0.71,0.78) | 8.42e-03 | 1.29e-07 |
| Belief in God | 0.83 (0.80,0.86) | 0.88 (0.85,0.91) | 0.87 (0.84,0.89) | 1.33e-01 | 1.1e-02 |
| Intrinsic Religiosity | 0.75 (0.72,0.79) | 0.82 (0.79,0.86) | 0.81 (0.78,0.84) | 4.57e-01 | 4.07e-03 |
| Religious Comfort | 0.80 (0.77,0.83) | 0.88 (0.85,0.91) | 0.86 (0.83,0.89) | 7.79e-01 | 3.07e-04 |
| Loved by God | 0.82 (0.79,0.86) | 0.89 (0.86,0.93) | 0.88 (0.85,0.90) | 3.36e-01 | 7.14e-04 |
| Spiritual Punishment | 0.45 (0.41,0.49) | 0.32 (0.27,0.37) | 0.35 (0.31,0.40) | 3.23e-04 | 2.58e-05 |
| Religious Criticism | 0.51 (0.47,0.56) | 0.41 (0.36,0.47) | 0.44 (0.40,0.48) | 3.36e-01 | 1.27e-03 |
| Evangelism | 0.71 (0.67,0.75) | 0.70 (0.64,0.75) | 0.70 (0.65,0.75) | 7.34e-01 | 5.6e-01 |
| *Family Factors* | | | | | |
| Ever Married | 0.70 (0.66,0.73) | 0.78 (0.75,0.82) | 1.00 (1.00,1.00) | 3.78e-03 | 3.08e-04 |
| Divorced | 0.04 (0.03,0.06) | 0.04 (0.03,0.06) | 0.06 (0.03,0.09) | 6.55e-01 | 9.13e-01 |
| Children | 1.03 (0.90,1.16) | 1.04 (0.92,1.16) | 1.27 (1.15,1.39) | 5.12e-01 | 8.64e-01 |

**Table S21a. Nationally representative descriptive statistics for United Kingdom**

| Characteristic | N = 5,368[1] |
|---|---|
| **Age group** | |
| 18-24 | 490 (9.1%) |
| 25-29 | 391 (7.3%) |
| 30-39 | 946 (18%) |
| 40-49 | 827 (15%) |
| 50-59 | 949 (18%) |
| 60-69 | 889 (17%) |
| 70-79 | 711 (13%) |
| 80 or older | 163 (3.0%) |
| (Missing) | 1 (<0.1%) |
| **Gender** | |
| Male | 2,557 (48%) |
| Female | 2,789 (52%) |
| Other | 14 (0.3%) |
| (Missing) | 9 (0.2%) |
| **Marital status** | |
| Married | 2,510 (47%) |
| Separated | 114 (2.1%) |
| Divorced | 435 (8.1%) |
| Widowed | 294 (5.5%) |
| Single, never married | 1,456 (27%) |
| Domestic Partner | 512 (9.5%) |
| (Missing) | 48 (0.9%) |
| **Employment** | |
| Employed for an employer | 2,798 (52%) |
| Self-employed | 469 (8.7%) |
| Retired | 1,262 (24%) |
| Student | 229 (4.3%) |
| Homemaker | 184 (3.4%) |
| Unemployed and looking for a job | 215 (4.0%) |
| None of these/Other | 201 (3.7%) |
| (Missing) | 11 (0.2%) |
| **Religious service attendance** | |
| More than 1/week | 291 (5.4%) |
| 1/week | 499 (9.3%) |
| 1-3/month | 293 (5.5%) |
| A few times a year | 1,165 (22%) |
| Never | 3,110 (58%) |
| (Missing) | 10 (0.2%) |
| **Education** | |
| Up to 8 years | 1,314 (24%) |
| 9-15 years | 2,072 (39%) |
| 16+ years | 1,974 (37%) |
| (Missing) | 8 (0.2%) |
| **Immigration** | |
| Born in this country | 4,659 (87%) |
| Born in another country | 682 (13%) |
| (Missing) | 27 (0.5%) |
| **Religious affiliation** | |
| Christianity | 2,750 (51%) |
| Islam | 218 (4.1%) |
| Hinduism | 61 (1.1%) |
| Buddhism | 30 (0.6%) |
| Judaism | 44 (0.8%) |
| Sikhism | 29 (0.5%) |
| Baha'i | 6 (0.1%) |
| Jainism | 4 (<0.1%) |
| Shinto | 0 (0%) |
| Taoism | 4 (<0.1%) |
| Confucianism | 2 (<0.1%) |
| Primal, Animist, or Folk religion | 36 (0.7%) |
| Spiritism | 0 (0%) |
| Umbanda, Candomble, and other African-derived religions | 0 (0%) |
| Chinese folk/traditional religion | 0 (0%) |
| Some other religion | 61 (1.1%) |
| No religion/Atheist/Agnostic | 2,099 (39%) |
| (Missing) | 25 (0.5%) |
| **Race/Ethnicity** | |

| **Characteristic** | **N = 5,368**[1] |
|---|---|
| (Missing) | 47 (0.9%) |
| Asian | 426 (7.9%) |
| Black | 152 (2.8%) |
| Other | 96 (1.8%) |
| White | 4,647 (87%) |

[1]n (%)

**Table S21b. Descriptive statistics of outcome variables for United Kingdom**

| Characteristic | N = 5,368[1] |
|---|---|
| **Flourishing Index** | 6.88 (1.69) |
| (Missing) | 147 |
| **Secure Flourishing Index** | 6.79 (1.67) |
| (Missing) | 173 |
| **Happiness & Life Satisfaction** | 6.59 (2.11) |
| (Missing) | 48 |
| **Social Relationship Quality** | 7.16 (2.31) |
| (Missing) | 54 |
| **Meaning and Purpose** | 6.77 (2.23) |
| (Missing) | 23 |
| **Character & Virtue** | 7.28 (1.68) |
| (Missing) | 37 |
| **Self-Rated Health** | 6.59 (2.09) |
| (Missing) | 11 |
| **Financial and Material Worry** | 6.33 (2.76) |
| (Missing) | 35 |
| **Happiness** | 6.71 (2.08) |
| (Missing) | 11 |
| **Life Satisfaction** | 6.49 (2.31) |
| (Missing) | 38 |
| **Present Life Evaluation** | 6.56 (2.01) |
| (Missing) | 15 |
| **Future Life Evaluation** | 7.13 (2.10) |
| (Missing) | 13 |
| **Optimism** | 6.99 (2.44) |
| (Missing) | 35 |
| **Freedom** | 7.05 (2.46) |
| (Missing) | 15 |
| **Peace** | |
| Always | 747 (14%) |
| Often | 3,092 (58%) |
| Rarely | 1,337 (25%) |
| Never | 175 (3.3%) |
| (Missing) | 18 (0.3%) |
| **Balance in Life** | |
| Always | 524 (9.8%) |
| Often | 3,206 (60%) |
| Rarely | 1,466 (27%) |
| Never | 150 (2.8%) |
| (Missing) | 22 (0.4%) |
| **Mastery** | |
| Always | 771 (14%) |
| Often | 3,532 (66%) |
| Rarely | 967 (18%) |
| Never | 77 (1.4%) |
| (Missing) | 21 (0.4%) |
| **Meaning** | 6.89 (2.31) |
| (Missing) | 12 |
| **Purpose** | 6.65 (2.64) |
| (Missing) | 12 |
| **Self-Rated Mental Health** | 6.80 (2.48) |
| (Missing) | 10 |
| **Content with My Relationships** | 7.31 (2.33) |
| (Missing) | 31 |
| **Satisfying Relationships** | 6.99 (2.49) |
| (Missing) | 29 |
| **Social Support** | 7.73 (2.47) |
| (Missing) | 15 |
| **Intimate Friend** | |
| Yes | 4,424 (82%) |
| No | 922 (17%) |
| (Missing) | 22 (0.4%) |
| **Government Approval** | |
| Strongly approve | 233 (4.3%) |
| Somewhat approve | 855 (16%) |
| Neither approve nor disapprove | 1,156 (22%) |
| Somewhat disapprove | 1,275 (24%) |
| Strongly disapprove | 1,837 (34%) |

| Characteristic | N = 5,368[1] |
| --- | --- |
| (Missing) | 12 (0.2%) |
| **Political Voice** | |
| Agree | 1,254 (23%) |
| Disagree | 3,117 (58%) |
| Unsure | 988 (18%) |
| (Missing) | 9 (0.2%) |
| **Belonging** | 7.13 (2.53) |
| (Missing) | 20 |
| **City Satisfaction** | |
| Satisfied | 3,780 (70%) |
| Dissatisfied | 1,009 (19%) |
| Unsure | 546 (10%) |
| (Missing) | 34 (0.6%) |
| **Trust** | |
| All | 106 (2.0%) |
| Most | 995 (19%) |
| Some | 2,488 (46%) |
| Not very many | 1,653 (31%) |
| None | 105 (2.0%) |
| (Missing) | 21 (0.4%) |
| **Community Participation** | |
| More than once a week | 553 (10%) |
| Once a week | 623 (12%) |
| One to three times a month | 663 (12%) |
| A few times a year | 998 (19%) |
| Never | 2,525 (47%) |
| (Missing) | 7 (0.1%) |
| **Traumatic Distress** | |
| A lot | 472 (8.8%) |
| Some | 1,319 (25%) |
| Not very much | 1,618 (30%) |
| None at all | 1,933 (36%) |
| (Missing) | 27 (0.5%) |
| **Suffering** | |
| A lot | 773 (14%) |
| Some | 1,942 (36%) |
| Not very much | 1,659 (31%) |
| None at all | 977 (18%) |
| (Missing) | 17 (0.3%) |
| **Loneliness** | 3.51 (2.91) |
| (Missing) | 6 |
| **Discrimination** | |
| Always | 276 (5.1%) |
| Often | 929 (17%) |
| Rarely | 2,181 (41%) |
| Never | 1,970 (37%) |
| (Missing) | 12 (0.2%) |
| **Promoting Good** | 7.61 (1.81) |
| (Missing) | 19 |
| **Delayed Gratification** | 6.96 (2.05) |
| (Missing) | 28 |
| **Hope** | 7.39 (2.24) |
| (Missing) | 117 |
| **Gratitude** | 7.40 (2.36) |
| (Missing) | 119 |
| **Love** | 8.14 (1.98) |
| (Missing) | 10 |
| **Forgiveness** | |
| Always | 918 (17%) |
| Often | 2,915 (54%) |
| Rarely | 1,302 (24%) |
| Never | 220 (4.1%) |
| (Missing) | 13 (0.2%) |
| **Charitable Giving** | |
| Yes | 3,254 (61%) |
| No | 2,108 (39%) |
| (Missing) | 6 (0.1%) |
| **Helping** | |
| Yes | 2,978 (55%) |

| Characteristic | N = 5,368[1] |
|---|---|
| No | 2,375 (44%) |
| (Missing) | 15 (0.3%) |
| **Volunteering** | |
| Yes | 1,590 (30%) |
| No | 3,769 (70%) |
| (Missing) | 8 (0.2%) |
| **Self-Rated Physical Health** | 6.37 (2.29) |
| (Missing) | 4 |
| **Health Limitations** | |
| Yes | 1,640 (31%) |
| No | 3,685 (69%) |
| (Missing) | 44 (0.8%) |
| **Pain** | |
| A lot | 961 (18%) |
| Some | 1,862 (35%) |
| Not very much | 1,580 (29%) |
| None at all | 946 (18%) |
| (Missing) | 18 (0.3%) |
| **Smoking** | 1.8 (5.1) |
| (Missing) | 54 |
| **Drinking** | 4 (8) |
| (Missing) | 38 |
| **Exercise** | |
| 0 days | 1,629 (30%) |
| 1 day | 567 (11%) |
| 2 days | 747 (14%) |
| 3 days | 708 (13%) |
| 4 days | 463 (8.6%) |
| 5 days | 454 (8.5%) |
| 6 days | 190 (3.5%) |
| 7 days/Every day | 600 (11%) |
| (Missing) | 9 (0.2%) |
| **Financial Stability** | 6.1 (3.0) |
| (Missing) | 8 |
| **Material Stability** | 6.60 (2.85) |
| (Missing) | 27 |
| **Education** | |
| Up to 8 years | 1,314 (24%) |
| 9-15 years | 2,072 (39%) |
| 16+ years | 1,974 (37%) |
| (Missing) | 8 (0.2%) |
| **Employment** | |
| Employed for an employer | 2,798 (52%) |
| Self-employed | 469 (8.7%) |
| Retired | 1,262 (24%) |
| Student | 229 (4.3%) |
| Homemaker | 184 (3.4%) |
| Unemployed and looking for a job | 215 (4.0%) |
| None of these/Other | 201 (3.7%) |
| (Missing) | 11 (0.2%) |
| **Subjective Financial Well-Being** | |
| Living comfortably on present income | 1,792 (33%) |
| Getting by on present income | 2,318 (43%) |
| Finding it difficult on present income | 874 (16%) |
| Finding it very difficult on present income | 331 (6.2%) |
| (Missing) | 53 (1.0%) |
| **Housing** | |
| Someone in this household OWNS this home | 3,090 (58%) |
| Someone in this household RENTS this home | 1,531 (29%) |
| Both | 272 (5.1%) |
| Neither | 417 (7.8%) |
| Rent | 0 (0%) |
| Own | 0 (0%) |
| Something else | 0 (0%) |
| (Missing) | 57 (1.1%) |
| **Self-Reported Religion/Spirituality** | |
| Always | 922 (17%) |
| Often | 1,265 (24%) |
| Rarely | 1,492 (28%) |

| Characteristic | N = 5,368[1] |
|---|---|
| Never | 1,670 (31%) |
| (Missing) | 20 (0.4%) |
| **Religious Service Attendance** | |
| More than once a week | 291 (5.4%) |
| Once a week | 499 (9.3%) |
| One to three times a month | 293 (5.5%) |
| A few times a year | 1,165 (22%) |
| Never | 3,110 (58%) |
| (Missing) | 10 (0.2%) |
| **Life after Death Belief** | |
| Yes | 2,074 (39%) |
| No | 1,499 (28%) |
| Unsure | 1,746 (33%) |
| (Missing) | 49 (0.9%) |
| **Religious Experience** | |
| Yes | 1,156 (22%) |
| No | 4,185 (78%) |
| (Missing) | 27 (0.5%) |
| **Religious Reading** | |
| More than once a day | 219 (4.1%) |
| About once a day | 488 (9.1%) |
| Sometimes | 1,409 (26%) |
| Never | 3,225 (60%) |
| (Missing) | 27 (0.5%) |
| **Prayer-Meditation** | |
| More than once a day | 559 (10%) |
| About once a day | 714 (13%) |
| Sometimes | 1,615 (30%) |
| Never | 2,460 (46%) |
| (Missing) | 20 (0.4%) |
| **Belief in God** | |
| One God | 2,037 (38%) |
| More than one god | 151 (2.8%) |
| An impersonal spiritual force | 703 (13%) |
| None of these | 1,560 (29%) |
| Unsure | 881 (16%) |
| (Missing) | 36 (0.7%) |
| **Intrinsic Religiosity** | |
| Agree | 1,499 (28%) |
| Disagree | 906 (17%) |
| Not relevant | 2,316 (43%) |
| Unsure | 625 (12%) |
| (Missing) | 23 (0.4%) |
| **Religious Comfort** | |
| Agree | 1,801 (34%) |
| Disagree | 703 (13%) |
| Not relevant | 2,256 (42%) |
| Unsure | 583 (11%) |
| (Missing) | 26 (0.5%) |
| **Loved by God** | |
| Agree | 1,703 (32%) |
| Disagree | 776 (14%) |
| Not relevant | 2,165 (40%) |
| Unsure | 690 (13%) |
| (Missing) | 33 (0.6%) |
| **Spiritual Punishment** | |
| Agree | 530 (9.9%) |
| Disagree | 2,116 (39%) |
| Not relevant | 2,142 (40%) |
| Unsure | 536 (10.0%) |
| (Missing) | 43 (0.8%) |
| **Religious Criticism** | |
| Agree | 425 (7.9%) |
| Disagree | 1,501 (28%) |
| Not relevant | 2,987 (56%) |
| Unsure | 424 (7.9%) |
| (Missing) | 31 (0.6%) |
| **Evangelism** | |
| Agree | 1,238 (23%) |

| Characteristic | N = 5,368[1] |
|---|---|
| Disagree | 1,278 (24%) |
| Not relevant | 2,499 (47%) |
| Unsure | 328 (6.1%) |
| (Missing) | 25 (0.5%) |
| **Children** | 0.57 (1.04) |
| (Missing) | 17 |

[1]Mean (SD); n (%)

**Table S21c. Demographic variation across outcomes for United Kingdom**

| Outcome | Male | Female | Other | Male vs Female p-value | Global p-value |
|---|---|---|---|---|---|
| *Flourishing Index and Domains* | | | | | |
| Flourishing Index | 6.98 (6.88,7.07) | 6.80 (6.71,6.89) | 5.20 (3.42,6.97) | 9.18e-03 | 2.36e-03 |
| Secure Flourishing Index | 6.92 (6.83,7.01) | 6.68 (6.59,6.76) | 5.30 (3.53,7.06) | 1.07e-03 | 1.32e-04 |
| Happiness & Life Satisfaction | 6.77 (6.65,6.88) | 6.46 (6.35,6.58) | 4.46 (2.80,6.12) | 2.69e-04 | 1.47e-05 |
| Social Relationship Quality | 7.17 (7.04,7.29) | 7.15 (7.03,7.27) | 5.44 (2.83,8.06) | 6.56e-01 | 3.2e-01 |
| Meaning and Purpose | 6.87 (6.74,6.99) | 6.69 (6.57,6.81) | 4.62 (2.56,6.68) | 4.02e-02 | 7.12e-03 |
| Character & Virtue | 7.35 (7.25,7.45) | 7.23 (7.14,7.32) | 6.74 (4.13,9.35) | 9.42e-02 | 1.86e-01 |
| Self-Rated Health | 6.74 (6.62,6.85) | 6.45 (6.34,6.57) | 4.71 (4.12,5.31) | 3.1e-03 | 2.92e-14 |
| Financial and Material Worry | 6.63 (6.49,6.78) | 6.07 (5.92,6.22) | 5.80 (3.71,7.89) | 6.18e-05 | 4.61e-07 |
| *Psychological Well-Being* | | | | | |
| Happiness | 6.89 (6.78,7.00) | 6.55 (6.44,6.66) | 4.53 (3.08,5.99) | 1.09e-04 | 4.26e-07 |
| Life Satisfaction | 6.64 (6.52,6.77) | 6.38 (6.25,6.50) | 4.39 (2.48,6.29) | 1.57e-03 | 5.47e-04 |
| Present Life Evaluation | 6.71 (6.60,6.81) | 6.42 (6.31,6.53) | 5.50 (3.16,7.83) | 7e-04 | 7.54e-04 |
| Future Life Evaluation | 7.12 (7.02,7.23) | 7.13 (7.01,7.24) | 6.73 (4.76,8.71) | 5.26e-01 | 9.01e-01 |
| Optimism | 7.05 (6.91,7.18) | 6.93 (6.80,7.06) | 5.03 (3.98,6.09) | 6.3e-01 | 5.96e-05 |
| Freedom | 7.07 (6.94,7.20) | 7.05 (6.92,7.18) | 5.89 (3.99,7.79) | 7.48e-01 | 3.7e-01 |
| Peace | 0.76 (0.73,0.78) | 0.69 (0.66,0.71) | 0.24 (0.00,0.54) | 9.59e-05 | 4.14e-07 |
| Balance in Life | 0.73 (0.70,0.75) | 0.67 (0.65,0.70) | 0.39 (0.00,0.89) | 4.2e-03 | 5.78e-03 |
| Mastery | 0.82 (0.80,0.84) | 0.79 (0.77,0.81) | 0.80 (0.53,1.07) | 1.48e-01 | 1.79e-01 |
| Meaning | 6.97 (6.85,7.10) | 6.83 (6.71,6.96) | 5.46 (3.00,7.92) | 3.96e-02 | 1.19e-01 |
| Purpose | 6.76 (6.62,6.91) | 6.55 (6.41,6.68) | 3.78 (2.00,5.57) | 8.99e-02 | 1.35e-04 |
| Self-Rated Mental Health | 7.00 (6.87,7.13) | 6.63 (6.49,6.77) | 3.86 (2.67,5.06) | 1.14e-03 | 8.71e-11 |
| *Social Well-Being* | | | | | |
| Content with My Relationships | 7.31 (7.19,7.44) | 7.33 (7.20,7.45) | 5.60 (2.74,8.46) | 7.01e-01 | 3.84e-01 |
| Satisfying Relationships | 7.02 (6.88,7.15) | 6.97 (6.84,7.10) | 5.28 (2.84,7.72) | 6.4e-01 | 2.41e-01 |
| Social Support | 7.68 (7.55,7.81) | 7.77 (7.63,7.91) | 6.92 (4.83,9.02) | 5.27e-01 | 4.29e-01 |
| Intimate Friend | 0.81 (0.79,0.84) | 0.84 (0.82,0.86) | 0.93 (0.82,1.04) | 8.88e-01 | 2.91e-02 |
| Government Approval | 0.25 (0.22,0.27) | 0.17 (0.15,0.19) | 0.02 (0.00,0.07) | 1.05e-04 | 1.6e-16 |
| Political Voice | 0.34 (0.31,0.37) | 0.25 (0.23,0.28) | 0.00 * | 7.52e-07 | 1.6e-16 |
| Belonging | 7.02 (6.89,7.16) | 7.24 (7.11,7.36) | 5.80 (3.85,7.76) | 3.48e-01 | 2.78e-02 |
| City Satisfaction | 0.78 (0.76,0.81) | 0.78 (0.76,0.80) | 0.43 (0.00,0.89) | 3.86e-01 | 1.79e-01 |
| Trust | 0.26 (0.24,0.29) | 0.15 (0.13,0.17) | 0.16 (0.00,0.42) | 4.25e-07 | 6.52e-12 |
| Community Participation | 0.25 (0.23,0.28) | 0.19 (0.17,0.21) | 0.05 (0.00,0.16) | 1.58e-03 | 9.5e-07 |
| *Psychological Distress* | | | | | |
| Traumatic Distress | 0.32 (0.30,0.35) | 0.35 (0.32,0.37) | 0.27 (0.00,0.58) | 1.96e-01 | 3.2e-01 |
| Depression Symptoms | 0.29 (0.26,0.31) | 0.29 (0.27,0.31) | 0.80 (0.54,1.07) | 4.59e-01 | 6.62e-05 |
| Anxiety Symptoms | 0.26 (0.24,0.29) | 0.31 (0.29,0.34) | 0.57 (0.13,1.01) | 2.04e-01 | 8.81e-03 |
| Suffering | 0.48 (0.46,0.51) | 0.53 (0.50,0.56) | 0.90 (0.72,1.08) | 1.07e-01 | 3.49e-07 |
| *Social Distress* | | | | | |
| Loneliness | 3.34 (3.18,3.49) | 3.66 (3.51,3.81) | 5.47 (3.77,7.16) | 5.76e-02 | 4.6e-04 |
| Discrimination | 0.25 (0.23,0.28) | 0.20 (0.18,0.22) | 0.37 (0.00,0.75) | 2.25e-02 | 2.2e-03 |
| *Character & Prosocial Behavior* | | | | | |
| Promoting Good | 7.61 (7.51,7.72) | 7.62 (7.52,7.71) | 6.77 (4.10,9.44) | 5.89e-01 | 7.64e-01 |
| Delayed Gratification | 7.09 (6.97,7.20) | 6.84 (6.74,6.95) | 6.72 (3.97,9.47) | 1.9e-02 | 9.12e-03 |
| Hope | 7.41 (7.28,7.53) | 7.37 (7.25,7.49) | 5.37 (3.57,7.17) | 4.97e-01 | 3.51e-02 |
| Gratitude | 7.35 (7.22,7.48) | 7.46 (7.34,7.58) | 5.76 (3.71,7.81) | 2.78e-01 | 9.17e-02 |
| Love | 7.82 (7.71,7.93) | 8.43 (8.33,8.52) | 7.64 (5.67,9.62) | 1.04e-06 | 1.84e-14 |
| Forgiveness | 0.70 (0.68,0.73) | 0.73 (0.71,0.75) | 0.47 (0.00,0.94) | 7.26e-01 | 1.23e-01 |
| Charitable Giving | 0.61 (0.58,0.63) | 0.61 (0.58,0.63) | 0.43 (0.00,0.87) | 5.78e-01 | 6.44e-01 |
| Helping | 0.56 (0.53,0.59) | 0.55 (0.53,0.58) | 0.77 (0.49,1.04) | 4.21e-01 | 1.92e-01 |
| Volunteering | 0.32 (0.29,0.34) | 0.28 (0.26,0.30) | 0.00 * | 8.29e-02 | 1.6e-16 |
| *Physical Health & Health Behavior* | | | | | |
| Self-Rated Physical Health | 6.48 (6.35,6.60) | 6.28 (6.15,6.40) | 5.57 (4.88,6.25) | 5.34e-02 | 2.26e-03 |
| Health Limitations | 0.28 (0.26,0.31) | 0.33 (0.31,0.36) | 0.57 (0.09,1.06) | 5.4e-02 | 9.35e-03 |
| Pain | 0.50 (0.47,0.53) | 0.55 (0.53,0.58) | 0.46 (0.02,0.89) | 3.15e-01 | 1.79e-02 |
| Smoking | 1.95 (1.68,2.23) | 1.57 (1.32,1.83) | 1.81 (0.00,4.60) | 1.29e-01 | 1.26e-01 |
| Drinking | 4.81 (4.45,5.16) | 3.06 (2.74,3.37) | 1.98 (0.00,4.28) | 6.34e-07 | 7.77e-13 |
| Exercise | 2.70 (2.57,2.83) | 2.42 (2.29,2.54) | 1.91 (0.73,3.08) | 9.83e-02 | 4.8e-03 |
| *Socioeconomic Outcomes* | | | | | |
| Financial Stability | 6.45 (6.29,6.60) | 5.73 (5.56,5.89) | 5.69 (3.31,8.06) | 5.82e-06 | 2.91e-09 |
| Material Stability | 6.82 (6.67,6.97) | 6.41 (6.25,6.56) | 5.92 (3.95,7.88) | 2.64e-03 | 5.6e-04 |
| Education | 0.39 (0.36,0.41) | 0.35 (0.33,0.37) | 0.23 (0.00,0.52) | 1.93e-01 | 5.43e-02 |
| Employment | 0.65 (0.62,0.67) | 0.58 (0.55,0.60) | 0.14 (0.00,0.36) | 3.89e-03 | 1.27e-08 |
| Subjective Financial Well-Being | 0.80 (0.78,0.82) | 0.75 (0.73,0.77) | 0.79 (0.51,1.06) | 3.6e-02 | 1.47e-02 |
| Housing | 0.65 (0.62,0.67) | 0.62 (0.60,0.65) | 0.10 (0.00,0.30) | 1.22e-01 | 3.09e-11 |
| *Religion/Spirituality* | | | | | |
| Self-Reported Religion/Spirituality | 0.41 (0.38,0.43) | 0.41 (0.39,0.44) | 0.15 (0.00,0.38) | 7.88e-01 | 3.54e-02 |

| Outcome | Male | Female | Other | Male vs Female p-value | Global p-value |
|---|---|---|---|---|---|
| Religious Service Attendance | 0.18 (0.16,0.20) | 0.12 (0.10,0.13) | 0.02 (0.00,0.07) | 4.07e-04 | 7.07e-11 |
| Life after Death Belief | 0.37 (0.34,0.39) | 0.41 (0.39,0.44) | 0.29 (0.00,0.68) | 1.41e-03 | 4.25e-02 |
| Religious Experience | 0.23 (0.20,0.25) | 0.21 (0.19,0.23) | 0.02 (0.00,0.07) | 4.13e-01 | 4.44e-15 |
| Religious Reading | 0.16 (0.14,0.18) | 0.11 (0.09,0.13) | 0.02 (0.00,0.07) | 3.31e-03 | 6.22e-08 |
| Prayer-Meditation | 0.24 (0.22,0.26) | 0.24 (0.22,0.26) | 0.00 * | 3.49e-01 | 1.6e-16 |
| Belief in God | 0.52 (0.50,0.55) | 0.56 (0.53,0.59) | 0.23 (0.00,0.51) | 4.64e-01 | 5.74e-03 |
| Intrinsic Religiosity | 0.44 (0.41,0.46) | 0.43 (0.40,0.46) | 0.10 (0.00,0.36) | 4.73e-01 | 9.75e-10 |
| Religious Comfort | 0.46 (0.43,0.49) | 0.51 (0.48,0.54) | 0.27 (0.00,0.84) | 7.8e-02 | 5.31e-03 |
| Loved by God | 0.45 (0.42,0.48) | 0.48 (0.45,0.52) | 0.11 (0.00,0.45) | 5.65e-01 | 1.6e-16 |
| Spiritual Punishment | 0.21 (0.18,0.24) | 0.16 (0.14,0.19) | 0.17 (0.00,0.47) | 3.96e-03 | 5.09e-03 |
| Religious Criticism | 0.23 (0.19,0.26) | 0.18 (0.15,0.21) | 0.28 (0.00,1.65) | 2.95e-03 | 7.56e-03 |
| Evangelism | 0.38 (0.35,0.41) | 0.37 (0.34,0.40) | 0.34 (0.00,1.15) | 4.04e-01 | 4.49e-01 |
| *Family Factors* | | | | | |
| Ever Married | 0.62 (0.60,0.65) | 0.64 (0.61,0.66) | 0.08 (0.00,0.21) | 5.68e-01 | 1.6e-16 |
| Divorced | 0.06 (0.05,0.07) | 0.10 (0.09,0.12) | 0.03 (0.00,0.11) | 3.72e-05 | 4.42e-05 |
| Children | 0.60 (0.54,0.66) | 0.54 (0.48,0.59) | 0.44 (0.00,0.92) | 1.26e-01 | 2.85e-01 |

**Table S22a. Nationally representative descriptive statistics for United States**

| Characteristic | N = 38,312[1] |
|---|---|
| **Age group** | |
| 18-24 | 2,682 (7.0%) |
| 25-29 | 3,540 (9.2%) |
| 30-39 | 7,284 (19%) |
| 40-49 | 5,649 (15%) |
| 50-59 | 6,745 (18%) |
| 60-69 | 6,832 (18%) |
| 70-79 | 4,054 (11%) |
| 80 or older | 1,525 (4.0%) |
| (Missing) | 0 (0%) |
| **Gender** | |
| Male | 18,222 (48%) |
| Female | 19,562 (51%) |
| Other | 392 (1.0%) |
| (Missing) | 136 (0.4%) |
| **Marital status** | |
| Married | 20,360 (53%) |
| Separated | 727 (1.9%) |
| Divorced | 3,636 (9.5%) |
| Widowed | 1,978 (5.2%) |
| Single, never married | 9,431 (25%) |
| Domestic Partner | 1,971 (5.1%) |
| (Missing) | 207 (0.5%) |
| **Employment** | |
| Employed for an employer | 19,502 (51%) |
| Self-employed | 3,445 (9.0%) |
| Retired | 9,016 (24%) |
| Student | 1,145 (3.0%) |
| Homemaker | 2,049 (5.3%) |
| Unemployed and looking for a job | 1,777 (4.6%) |
| None of these/Other | 1,292 (3.4%) |
| (Missing) | 87 (0.2%) |
| **Religious service attendance** | |
| More than 1/week | 2,633 (6.9%) |
| 1/week | 5,887 (15%) |
| 1-3/month | 2,819 (7.4%) |
| A few times a year | 8,870 (23%) |
| Never | 17,975 (47%) |
| (Missing) | 128 (0.3%) |
| **Education** | |
| Up to 8 years | 210 (0.5%) |
| 9-15 years | 25,322 (66%) |
| 16+ years | 12,705 (33%) |
| (Missing) | 75 (0.2%) |
| **Immigration** | |
| Born in this country | 34,865 (91%) |
| Born in another country | 3,020 (7.9%) |
| (Missing) | 427 (1.1%) |
| **Religious affiliation** | |
| Christianity | 22,954 (60%) |
| Islam | 205 (0.5%) |
| Hinduism | 167 (0.4%) |
| Buddhism | 336 (0.9%) |
| Judaism | 638 (1.7%) |
| Sikhism | 24 (<0.1%) |
| Baha'i | 13 (<0.1%) |
| Jainism | 18 (<0.1%) |
| Shinto | 12 (<0.1%) |
| Taoism | 93 (0.2%) |
| Confucianism | 8 (<0.1%) |
| Primal, Animist, or Folk religion | 240 (0.6%) |
| Spiritism | 0 (0%) |
| Umbanda, Candomble, and other African-derived religions | 0 (0%) |
| Chinese folk/traditional religion | 0 (0%) |
| Some other religion | 1,267 (3.3%) |
| No religion/Atheist/Agnostic | 11,870 (31%) |
| (Missing) | 467 (1.2%) |
| **Race/Ethnicity** | |

| Characteristic | N = 38,312[1] |
|---|---|
| (Missing) | 20 (<0.1%) |
| Asian | 2,466 (6.4%) |
| Black | 4,501 (12%) |
| Hispanic | 6,724 (18%) |
| Other | 997 (2.6%) |
| White | 23,605 (62%) |

[1]n (%)

**Table S22b. Descriptive statistics of outcome variables for United States**

| Characteristic | N = 38,312[1] |
|---|---|
| **Flourishing Index** | 7.18 (1.65) |
| (Missing) | 449 |
| **Secure Flourishing Index** | 7.11 (1.66) |
| (Missing) | 498 |
| **Happiness & Life Satisfaction** | 6.93 (1.95) |
| (Missing) | 61 |
| **Social Relationship Quality** | 7.18 (2.31) |
| (Missing) | 128 |
| **Meaning and Purpose** | 7.14 (2.18) |
| (Missing) | 70 |
| **Character & Virtue** | 7.66 (1.60) |
| (Missing) | 187 |
| **Self-Rated Health** | 7.00 (1.83) |
| (Missing) | 42 |
| **Financial and Material Worry** | 6.75 (2.87) |
| (Missing) | 54 |
| **Happiness** | 7.01 (1.92) |
| (Missing) | 20 |
| **Life Satisfaction** | 6.86 (2.14) |
| (Missing) | 41 |
| **Present Life Evaluation** | 6.94 (1.85) |
| (Missing) | 21 |
| **Future Life Evaluation** | 7.73 (1.93) |
| (Missing) | 105 |
| **Optimism** | 7.65 (2.29) |
| (Missing) | 185 |
| **Freedom** | 7.21 (2.53) |
| (Missing) | 41 |
| **Peace** | |
| Always | 6,176 (16%) |
| Often | 23,864 (62%) |
| Rarely | 7,551 (20%) |
| Never | 629 (1.6%) |
| (Missing) | 93 (0.2%) |
| **Balance in Life** | |
| Always | 3,424 (8.9%) |
| Often | 24,818 (65%) |
| Rarely | 9,037 (24%) |
| Never | 933 (2.4%) |
| (Missing) | 100 (0.3%) |
| **Mastery** | |
| Always | 6,832 (18%) |
| Often | 26,031 (68%) |
| Rarely | 4,795 (13%) |
| Never | 445 (1.2%) |
| (Missing) | 209 (0.5%) |
| **Meaning** | 7.30 (2.19) |
| (Missing) | 19 |
| **Purpose** | 6.99 (2.59) |
| (Missing) | 52 |
| **Self-Rated Mental Health** | 7.19 (2.25) |
| (Missing) | 19 |
| **Content with My Relationships** | 7.38 (2.33) |
| (Missing) | 54 |
| **Satisfying Relationships** | 6.97 (2.47) |
| (Missing) | 80 |
| **Social Support** | 8.02 (2.44) |
| (Missing) | 57 |
| **Intimate Friend** | |
| Yes | 33,047 (86%) |
| No | 5,139 (13%) |
| (Missing) | 125 (0.3%) |
| **Government Approval** | |
| Strongly approve | 1,139 (3.0%) |
| Somewhat approve | 7,817 (20%) |
| Neither approve nor disapprove | 5,671 (15%) |
| Somewhat disapprove | 8,833 (23%) |
| Strongly disapprove | 14,777 (39%) |

| Characteristic | N = 38,312[1] |
|---|---|
| (Missing) | 76 (0.2%) |
| **Political Voice** | |
| Agree | 9,708 (25%) |
| Disagree | 20,703 (54%) |
| Unsure | 7,847 (20%) |
| (Missing) | 54 (0.1%) |
| **Belonging** | 7.07 (2.65) |
| (Missing) | 181 |
| **City Satisfaction** | |
| Satisfied | 26,185 (68%) |
| Dissatisfied | 8,624 (23%) |
| Unsure | 3,298 (8.6%) |
| (Missing) | 205 (0.5%) |
| **Trust** | |
| All | 57 (0.1%) |
| Most | 3,593 (9.4%) |
| Some | 16,636 (43%) |
| Not very many | 17,053 (45%) |
| None | 840 (2.2%) |
| (Missing) | 133 (0.3%) |
| **Community Participation** | |
| More than once a week | 2,463 (6.4%) |
| Once a week | 2,616 (6.8%) |
| One to three times a month | 4,823 (13%) |
| A few times a year | 11,381 (30%) |
| Never | 16,976 (44%) |
| (Missing) | 53 (0.1%) |
| **Traumatic Distress** | |
| A lot | 2,717 (7.1%) |
| Some | 7,731 (20%) |
| Not very much | 12,806 (33%) |
| None at all | 14,989 (39%) |
| (Missing) | 69 (0.2%) |
| **Suffering** | |
| A lot | 3,597 (9.4%) |
| Some | 12,928 (34%) |
| Not very much | 14,503 (38%) |
| None at all | 7,158 (19%) |
| (Missing) | 125 (0.3%) |
| **Loneliness** | 3.29 (2.84) |
| (Missing) | 26 |
| **Discrimination** | |
| Always | 1,756 (4.6%) |
| Often | 8,170 (21%) |
| Rarely | 19,279 (50%) |
| Never | 8,999 (23%) |
| (Missing) | 109 (0.3%) |
| **Promoting Good** | 7.87 (1.70) |
| (Missing) | 55 |
| **Delayed Gratification** | 7.45 (1.96) |
| (Missing) | 143 |
| **Hope** | 7.68 (2.16) |
| (Missing) | 1,779 |
| **Gratitude** | 8.12 (2.21) |
| (Missing) | 1,776 |
| **Love** | 8.56 (1.71) |
| (Missing) | 25 |
| **Forgiveness** | |
| Always | 7,303 (19%) |
| Often | 22,578 (59%) |
| Rarely | 7,375 (19%) |
| Never | 968 (2.5%) |
| (Missing) | 88 (0.2%) |
| **Charitable Giving** | |
| Yes | 18,722 (49%) |
| No | 19,547 (51%) |
| (Missing) | 43 (0.1%) |
| **Helping** | |
| Yes | 22,619 (59%) |

| Characteristic | N = 38,312[1] |
|---|---|
| No | 15,655 (41%) |
| (Missing) | 39 (0.1%) |
| **Volunteering** | |
| Yes | 10,395 (27%) |
| No | 27,856 (73%) |
| (Missing) | 61 (0.2%) |
| **Self-Rated Physical Health** | 6.81 (1.93) |
| (Missing) | 32 |
| **Health Limitations** | |
| Yes | 9,367 (24%) |
| No | 28,857 (75%) |
| (Missing) | 88 (0.2%) |
| **Pain** | |
| A lot | 5,277 (14%) |
| Some | 14,353 (37%) |
| Not very much | 13,285 (35%) |
| None at all | 5,361 (14%) |
| (Missing) | 37 (<0.1%) |
| **Smoking** | 1.25 (4.61) |
| (Missing) | 226 |
| **Drinking** | 2.7 (5.8) |
| (Missing) | 165 |
| **Exercise** | |
| 0 days | 10,147 (26%) |
| 1 day | 4,561 (12%) |
| 2 days | 5,293 (14%) |
| 3 days | 6,007 (16%) |
| 4 days | 3,740 (9.8%) |
| 5 days | 3,728 (9.7%) |
| 6 days | 1,486 (3.9%) |
| 7 days/Every day | 3,293 (8.6%) |
| (Missing) | 56 (0.1%) |
| **Financial Stability** | 6.49 (3.10) |
| (Missing) | 21 |
| **Material Stability** | 7.02 (2.93) |
| (Missing) | 35 |
| **Education** | |
| Up to 8 years | 210 (0.5%) |
| 9-15 years | 25,322 (66%) |
| 16+ years | 12,705 (33%) |
| (Missing) | 75 (0.2%) |
| **Employment** | |
| Employed for an employer | 19,502 (51%) |
| Self-employed | 3,445 (9.0%) |
| Retired | 9,016 (24%) |
| Student | 1,145 (3.0%) |
| Homemaker | 2,049 (5.3%) |
| Unemployed and looking for a job | 1,777 (4.6%) |
| None of these/Other | 1,292 (3.4%) |
| (Missing) | 87 (0.2%) |
| **Subjective Financial Well-Being** | |
| Living comfortably on present income | 15,724 (41%) |
| Getting by on present income | 15,131 (39%) |
| Finding it difficult on present income | 5,447 (14%) |
| Finding it very difficult on present income | 1,926 (5.0%) |
| (Missing) | 83 (0.2%) |
| **Housing** | |
| Someone in this household OWNS this home | 25,750 (67%) |
| Someone in this household RENTS this home | 8,910 (23%) |
| Both | 1,457 (3.8%) |
| Neither | 2,127 (5.6%) |
| Rent | 0 (0%) |
| Own | 0 (0%) |
| Something else | 0 (0%) |
| (Missing) | 67 (0.2%) |
| **Self-Reported Religion/Spirituality** | |
| Always | 9,740 (25%) |
| Often | 12,379 (32%) |
| Rarely | 9,093 (24%) |

| Characteristic | N = 38,312[1] |
|---|---|
| Never | 7,027 (18%) |
| (Missing) | 73 (0.2%) |
| **Religious Service Attendance** | |
| More than once a week | 2,633 (6.9%) |
| Once a week | 5,887 (15%) |
| One to three times a month | 2,819 (7.4%) |
| A few times a year | 8,870 (23%) |
| Never | 17,975 (47%) |
| (Missing) | 128 (0.3%) |
| **Life after Death Belief** | |
| Yes | 21,221 (55%) |
| No | 6,644 (17%) |
| Unsure | 10,303 (27%) |
| (Missing) | 144 (0.4%) |
| **Religious Experience** | |
| Yes | 13,546 (35%) |
| No | 24,589 (64%) |
| (Missing) | 177 (0.5%) |
| **Religious Reading** | |
| More than once a day | 2,500 (6.5%) |
| About once a day | 5,419 (14%) |
| Sometimes | 13,658 (36%) |
| Never | 16,533 (43%) |
| (Missing) | 202 (0.5%) |
| **Prayer-Meditation** | |
| More than once a day | 7,841 (20%) |
| About once a day | 8,252 (22%) |
| Sometimes | 13,202 (34%) |
| Never | 8,928 (23%) |
| (Missing) | 89 (0.2%) |
| **Belief in God** | |
| One God | 21,947 (57%) |
| More than one god | 1,034 (2.7%) |
| An impersonal spiritual force | 4,714 (12%) |
| None of these | 5,629 (15%) |
| Unsure | 4,849 (13%) |
| (Missing) | 138 (0.4%) |
| **Intrinsic Religiosity** | |
| Agree | 16,798 (44%) |
| Disagree | 6,520 (17%) |
| Not relevant | 10,910 (28%) |
| Unsure | 3,901 (10%) |
| (Missing) | 183 (0.5%) |
| **Religious Comfort** | |
| Agree | 21,610 (56%) |
| Disagree | 3,997 (10%) |
| Not relevant | 9,790 (26%) |
| Unsure | 2,823 (7.4%) |
| (Missing) | 92 (0.2%) |
| **Loved by God** | |
| Agree | 21,177 (55%) |
| Disagree | 3,617 (9.4%) |
| Not relevant | 9,620 (25%) |
| Unsure | 3,762 (9.8%) |
| (Missing) | 135 (0.4%) |
| **Spiritual Punishment** | |
| Agree | 3,383 (8.8%) |
| Disagree | 22,290 (58%) |
| Not relevant | 9,276 (24%) |
| Unsure | 3,240 (8.5%) |
| (Missing) | 123 (0.3%) |
| **Religious Criticism** | |
| Agree | 2,895 (7.6%) |
| Disagree | 15,311 (40%) |
| Not relevant | 16,092 (42%) |
| Unsure | 3,873 (10%) |
| (Missing) | 142 (0.4%) |
| **Evangelism** | |
| Agree | 13,960 (36%) |

| **Characteristic** | **N = 38,312**[1] |
|---|---|
| Disagree | 10,913 (28%) |
| Not relevant | 11,290 (29%) |
| Unsure | 1,998 (5.2%) |
| (Missing) | 152 (0.4%) |
| **Children** | 0.59 (1.28) |
| (Missing) | 232 |

[1]Mean (SD); n (%)

**Table S22c. Demographic variation across outcomes for United States**

| Outcome | Male | Female | Other | Male vs Female p-value | Global p-value |
|---|---|---|---|---|---|
| *Flourishing Index and Domains* | | | | | |
| Flourishing Index | 7.20 (7.14,7.27) | 7.18 (7.12,7.25) | 6.19 (5.69,6.69) | 7.01e-01 | 4.31e-04 |
| Secure Flourishing Index | 7.16 (7.09,7.22) | 7.09 (7.02,7.15) | 6.03 (5.58,6.48) | 7.05e-01 | 3.68e-06 |
| Happiness & Life Satisfaction | 6.94 (6.87,7.01) | 6.95 (6.87,7.02) | 5.82 (5.18,6.45) | 2.85e-01 | 2.32e-03 |
| Social Relationship Quality | 7.17 (7.08,7.26) | 7.21 (7.12,7.29) | 6.13 (5.37,6.90) | 2.15e-01 | 2.25e-02 |
| Meaning and Purpose | 7.13 (7.04,7.22) | 7.19 (7.11,7.27) | 5.54 (4.97,6.11) | 2.83e-01 | 1.31e-07 |
| Character & Virtue | 7.68 (7.62,7.74) | 7.64 (7.58,7.71) | 7.30 (6.68,7.92) | 7.52e-01 | 3.57e-01 |
| Self-Rated Health | 7.09 (7.03,7.16) | 6.92 (6.85,6.99) | 6.15 (5.59,6.72) | 4.37e-02 | 3.32e-05 |
| Financial and Material Worry | 6.92 (6.81,7.04) | 6.63 (6.52,6.73) | 5.23 (4.56,5.90) | 1.49e-02 | 3.37e-08 |
| *Psychological Well-Being* | | | | | |
| Happiness | 7.02 (6.95,7.09) | 7.02 (6.94,7.09) | 5.99 (5.13,6.84) | 2.96e-01 | 5.82e-02 |
| Life Satisfaction | 6.86 (6.78,6.94) | 6.87 (6.79,6.96) | 5.65 (5.15,6.15) | 3.11e-01 | 1.39e-05 |
| Present Life Evaluation | 6.93 (6.85,7.00) | 6.96 (6.89,7.03) | 6.09 (5.71,6.47) | 8.05e-02 | 4.56e-05 |
| Future Life Evaluation | 7.67 (7.60,7.73) | 7.80 (7.72,7.87) | 7.09 (6.58,7.59) | 2.79e-02 | 1.45e-03 |
| Optimism | 7.56 (7.47,7.65) | 7.77 (7.69,7.85) | 6.06 (5.17,6.95) | 5.53e-03 | 6.14e-06 |
| Freedom | 7.18 (7.08,7.27) | 7.27 (7.18,7.36) | 5.48 (4.93,6.03) | 8.46e-03 | 2.34e-09 |
| Peace | 0.81 (0.79,0.82) | 0.77 (0.76,0.79) | 0.56 (0.40,0.72) | 8.16e-02 | 2.23e-04 |
| Balance in Life | 0.74 (0.72,0.76) | 0.74 (0.72,0.76) | 0.54 (0.37,0.70) | 4.29e-01 | 4.52e-02 |
| Mastery | 0.87 (0.85,0.89) | 0.86 (0.84,0.87) | 0.77 (0.64,0.90) | 9.47e-01 | 1.82e-01 |
| Meaning | 7.26 (7.18,7.35) | 7.35 (7.27,7.43) | 6.02 (5.43,6.60) | 1.47e-01 | 2.89e-05 |
| Purpose | 6.99 (6.89,7.10) | 7.03 (6.94,7.12) | 5.06 (4.36,5.77) | 5.49e-01 | 3.98e-07 |
| Self-Rated Mental Health | 7.36 (7.28,7.44) | 7.05 (6.96,7.14) | 5.77 (4.86,6.68) | 1.8e-03 | 4.57e-08 |
| *Social Well-Being* | | | | | |
| Content with My Relationships | 7.37 (7.28,7.46) | 7.41 (7.33,7.50) | 6.43 (5.51,7.36) | 1.89e-01 | 1.05e-01 |
| Satisfying Relationships | 6.97 (6.87,7.06) | 7.00 (6.91,7.09) | 5.83 (5.06,6.61) | 2.69e-01 | 1.26e-02 |
| Social Support | 7.94 (7.82,8.05) | 8.11 (8.01,8.20) | 7.49 (6.75,8.24) | 3.11e-02 | 2.59e-02 |
| Intimate Friend | 0.83 (0.82,0.85) | 0.89 (0.88,0.91) | 0.90 (0.82,0.99) | 1.95e-04 | 7.44e-09 |
| Government Approval | 0.22 (0.21,0.23) | 0.25 (0.24,0.26) | 0.10 (0.04,0.16) | 5.67e-04 | 4.79e-07 |
| Political Voice | 0.33 (0.32,0.35) | 0.32 (0.31,0.34) | 0.26 (0.08,0.45) | 3.69e-01 | 4.27e-01 |
| Belonging | 7.06 (6.95,7.16) | 7.15 (7.05,7.24) | 4.38 (3.73,5.03) | 2.7e-01 | 1.33e-15 |
| City Satisfaction | 0.76 (0.74,0.78) | 0.74 (0.72,0.75) | 0.66 (0.50,0.82) | 4.17e-01 | 1.36e-01 |
| Trust | 0.12 (0.11,0.13) | 0.08 (0.07,0.09) | 0.02 (0.00,0.03) | 8.47e-03 | 1.6e-16 |
| Community Participation | 0.14 (0.13,0.15) | 0.12 (0.11,0.13) | 0.28 (0.14,0.41) | 1.35e-02 | 1.56e-03 |
| *Psychological Distress* | | | | | |
| Traumatic Distress | 0.24 (0.22,0.25) | 0.31 (0.29,0.32) | 0.35 (0.21,0.49) | 3.5e-05 | 1.41e-09 |
| Depression Symptoms | 0.20 (0.18,0.22) | 0.21 (0.19,0.22) | 0.26 (0.13,0.39) | 3.6e-01 | 5.33e-01 |
| Anxiety Symptoms | 0.20 (0.19,0.22) | 0.28 (0.26,0.29) | 0.57 (0.41,0.73) | 7.33e-05 | 7.35e-13 |
| Suffering | 0.39 (0.37,0.41) | 0.47 (0.45,0.48) | 0.63 (0.46,0.81) | 9.78e-06 | 3.77e-10 |
| *Social Distress* | | | | | |
| Loneliness | 3.21 (3.09,3.32) | 3.35 (3.24,3.45) | 4.51 (3.60,5.42) | 6.33e-01 | 6.8e-03 |
| Discrimination | 0.25 (0.24,0.27) | 0.26 (0.25,0.28) | 0.54 (0.37,0.71) | 4.82e-01 | 3.58e-03 |
| *Character & Prosocial Behavior* | | | | | |
| Promoting Good | 7.90 (7.84,7.96) | 7.84 (7.76,7.93) | 7.66 (7.04,8.28) | 7.71e-01 | 4.32e-01 |
| Delayed Gratification | 7.46 (7.39,7.54) | 7.44 (7.37,7.51) | 6.94 (6.20,7.68) | 7.83e-01 | 3.63e-01 |
| Hope | 7.66 (7.58,7.74) | 7.76 (7.67,7.85) | 5.94 (5.20,6.68) | 4.25e-02 | 4.14e-06 |
| Gratitude | 7.96 (7.87,8.05) | 8.34 (8.26,8.42) | 6.57 (5.72,7.43) | 3.54e-05 | 9.05e-12 |
| Love | 8.32 (8.25,8.40) | 8.79 (8.72,8.85) | 8.08 (7.52,8.64) | 5.24e-12 | 1.6e-16 |
| Forgiveness | 0.78 (0.76,0.79) | 0.79 (0.78,0.81) | 0.57 (0.40,0.73) | 2.76e-01 | 1.11e-02 |
| Charitable Giving | 0.47 (0.45,0.49) | 0.51 (0.49,0.53) | 0.42 (0.26,0.59) | 2.53e-02 | 1.48e-03 |
| Helping | 0.58 (0.56,0.59) | 0.60 (0.59,0.62) | 0.62 (0.47,0.77) | 7.13e-02 | 5.46e-02 |
| Volunteering | 0.26 (0.25,0.27) | 0.28 (0.27,0.30) | 0.26 (0.13,0.39) | 3.34e-02 | 4.63e-02 |
| *Physical Health & Health Behavior* | | | | | |
| Self-Rated Physical Health | 6.83 (6.76,6.90) | 6.79 (6.72,6.86) | 6.54 (5.94,7.14) | 8.94e-01 | 5.07e-01 |
| Health Limitations | 0.22 (0.20,0.23) | 0.27 (0.26,0.28) | 0.29 (0.16,0.42) | 2.65e-05 | 2.09e-06 |
| Pain | 0.48 (0.46,0.50) | 0.54 (0.52,0.55) | 0.67 (0.53,0.82) | 7.53e-04 | 1.06e-06 |
| Smoking | 1.29 (1.13,1.44) | 1.20 (1.02,1.38) | 0.48 (0.00,0.96) | 1.56e-01 | 3.25e-03 |
| Drinking | 3.49 (3.32,3.66) | 1.84 (1.73,1.94) | 1.41 (0.70,2.11) | 1.6e-16 | 1.6e-16 |
| Exercise | 2.73 (2.65,2.81) | 2.44 (2.37,2.50) | 2.84 (1.94,3.74) | 2.42e-03 | 2.51e-07 |
| *Socioeconomic Outcomes* | | | | | |
| Financial Stability | 6.71 (6.60,6.83) | 6.31 (6.20,6.42) | 5.07 (4.40,5.73) | 5.28e-03 | 9.91e-10 |
| Material Stability | 7.13 (7.02,7.25) | 6.94 (6.84,7.05) | 5.40 (4.69,6.11) | 6.53e-02 | 2.33e-06 |
| Education | 0.32 (0.31,0.34) | 0.34 (0.33,0.35) | 0.31 (0.19,0.44) | 4.27e-02 | 6.97e-02 |
| Employment | 0.66 (0.64,0.67) | 0.55 (0.53,0.56) | 0.57 (0.40,0.74) | 8.75e-14 | 1.6e-16 |
| Subjective Financial Well-Being | 0.84 (0.82,0.85) | 0.78 (0.76,0.79) | 0.80 (0.69,0.92) | 1.33e-03 | 1.38e-06 |
| Housing | 0.73 (0.71,0.74) | 0.70 (0.69,0.72) | 0.45 (0.28,0.61) | 1.15e-01 | 8.2e-04 |
| *Religion/Spirituality* | | | | | |
| Self-Reported Religion/Spirituality | 0.53 (0.51,0.55) | 0.63 (0.61,0.65) | 0.34 (0.17,0.52) | 1.36e-12 | 1.6e-16 |

| Outcome | Male | Female | Other | Male vs Female p-value | Global p-value |
|---|---|---|---|---|---|
| Religious Service Attendance | 0.21 (0.19,0.22) | 0.24 (0.23,0.25) | 0.03 (0.01,0.05) | 4.77e-03 | 1.6e-16 |
| Life after Death Belief | 0.51 (0.50,0.53) | 0.60 (0.59,0.62) | 0.30 (0.13,0.48) | 2.25e-06 | 6.35e-14 |
| Religious Experience | 0.34 (0.32,0.35) | 0.37 (0.36,0.38) | 0.35 (0.18,0.52) | 1.81e-01 | 1.9e-02 |
| Religious Reading | 0.18 (0.16,0.19) | 0.24 (0.22,0.25) | 0.05 (0.01,0.09) | 4.48e-05 | 1.6e-16 |
| Prayer-Meditation | 0.37 (0.35,0.38) | 0.48 (0.46,0.49) | 0.26 (0.08,0.43) | 5.22e-14 | 1.6e-16 |
| Belief in God | 0.68 (0.66,0.70) | 0.78 (0.76,0.79) | 0.42 (0.26,0.58) | 1.83e-09 | 1.6e-16 |
| Intrinsic Religiosity | 0.54 (0.52,0.56) | 0.62 (0.59,0.64) | 0.35 (0.15,0.55) | 1.38e-06 | 4.64e-11 |
| Religious Comfort | 0.64 (0.62,0.66) | 0.73 (0.72,0.75) | 0.46 (0.28,0.63) | 4.07e-10 | <2e-16 |
| Loved by God | 0.63 (0.61,0.64) | 0.73 (0.72,0.75) | 0.43 (0.26,0.60) | 1.03e-11 | 1.6e-16 |
| Spiritual Punishment | 0.15 (0.13,0.17) | 0.15 (0.13,0.17) | 0.21 (0.00,0.44) | 5.67e-01 | 6.08e-01 |
| Religious Criticism | 0.22 (0.20,0.24) | 0.21 (0.19,0.23) | 0.47 (0.25,0.70) | 1.31e-01 | 3.77e-03 |
| Evangelism | 0.50 (0.48,0.52) | 0.53 (0.51,0.55) | 0.54 (0.37,0.70) | 1.15e-01 | 6.56e-02 |
| *Family Factors* | | | | | |
| Ever Married | 0.70 (0.68,0.72) | 0.71 (0.69,0.73) | 0.31 (0.19,0.43) | 2.86e-02 | 3.02e-10 |
| Divorced | 0.08 (0.07,0.08) | 0.11 (0.11,0.12) | 0.06 (0.01,0.11) | 7.18e-09 | 6.5e-13 |
| Children | 0.51 (0.47,0.54) | 0.65 (0.59,0.72) | 0.66 (0.16,1.17) | 7.91e-03 | 2.39e-04 |

**Supplementary Text: Further Considerations of Sex and Gender**

Categorizations of sex have been somewhat fraught and contested in recent years, and there is no uniform or unambiguous agreement as to what defines these groupings (Rehmann-Sutter et al., 2023). Perhaps the most common and well-accepted way though of differentiating male and female is in terms of gametes (reproductive cells), not least because this binary division is found throughout the animal kingdom. As Goymann et al. (2023) put it, "Biological sex is defined as a binary variable in every sexually reproducing plant and animal species. With a few exceptions, all sexually reproducing organisms generate exactly two types of gametes that are distinguished by their difference in size: females, by definition, produce large gametes (eggs) and males, by definition, produce small and usually motile gametes (sperm)." This distinction is the basis, for example, of an updated definition of sex formulated by the US Department of Health and Human Services (2025), who state that "An individual human is either female or male based on whether the person is of the sex characterized by a reproductive system with the biological function of producing eggs (ova) or sperm." The distinction still applies for people who cannot actually produce gametes for some reason, and is more about a person's body being organized around the *potential* to produce gametes (e.g., a woman who has had her ovaries removed is still female). Although recent years have seen an emerging trend to say sex is "assigned at birth" (Alpert et al., 2021)—often implying it is done superficially or with uncertainty (e.g., based merely on looking at genitals)—it is still generally understood that sex is determined shortly after conception, based on gene expression in the developing foetus, and relatedly is detectable very soon after based on technologies such as DNA testing (Erickson, 1997; Orzack et al., 2015).

But even while sex is generally acknowledged as being conceptually binary, not all people fit neatly into the binary categories—with the sex distribution technically "bimodal"—with "intersex" defined by the WHO as "an umbrella term for individuals born with natural variations in biological or physiological characteristics (including sexual anatomy, reproductive organs and/or chromosomal patterns) that do not fit traditional definitions of male or female." Estimates of the percentage of intersex people varies depending on how broadly one defines it, with Fausto-Sterling (2000) influentially claiming the prevalence might be up to 1.7%. Sax (2002) though argued that this figure includes conditions which most clinicians do not recognize as intersex—such as Klinefelter syndrome, a genetic condition where a male has an additional copy of the X chromosome—and the label should be "restricted to those conditions in which chromosomal sex is inconsistent with phenotypic sex, or in which the phenotype is not classifiable as either male or female," in which case the prevalence is about 0.018%. Whatever the exact prevalence though, intersex is generally understood as not constituting a "third sex", nor a plurality of sexes. There are still just two sexes, but a small minority of people do not fall neatly or obviously into one category or another, as physically they may possess reproductive elements of both sexes. But the condition still substantiates the idea of two basic gamete-driven categories, even if some people could be seen as overlapping or combining these categories in idiosyncratic ways. Then, for the vast majority of people who are not intersex, their sexual categorization is generally uncomplicated and uncontroversial. Indeed, the ability to perceive and categorize people according to sex is one of the earliest capacities of infants, being linked to considerations such as the caregiving role (e.g., providing milk) typically offered by females (Ramsey et al., 2005).

The picture becomes even more complicated though, as the very terms "women" and men" have become disputed, especially in certain places, like the USA. There is still relatively widespread agreement that the terms "male" and "female" should be reserved for the binary sex categories outlined above,

although even here some scholars are seeking to complicate and dispute this labelling (Rosenthal, 2024), so for clarity and emphasis people might also use the adjective "biological." But the woman/man labels have become sites of discursive battles, above all in relation to the issue of transgenderism. While this is a hugely complicated and increasingly fraught topic, the issue essentially rests on the claim that some people do not believe that their gender aligns with their sex. A person may be biologically male, for instance, but come to feel misaligned with the norms and roles associated with masculinity in their culture and instead are more attuned with feminine ideals and roles. But what makes the issue especially highly-charged is how it challenges the conventional way in which the social categories of men and women—with all their socio-cultural ramifications—are overlaid upon the biological sex categories of male and female. The gender non-conforming male, for instance, might not only challenge expectations and embrace conventional signifies of femininity (e.g., wearing a dress), but assert they actually *are* a woman, and should moreover be treated as one. That could include, for example, being allowed to enter spaces usually reserved for women, such as female changing rooms or sporting competitions. However, some females have challenged such developments—though others have been supportive (Worthen, 2022)—in a stance sometimes known as as "gender critical feminism" (Amery, 2024).

It has been argued, for instance, that one reason for the creation of such spaces is that males overall can pose risks to females in various ways, given that males are on average bigger, stronger, and more aggressive (e.g., due to biological factors such as higher testosterone levels). As such, gender critical feminists have argued that spaces created for women, and likewise the conceptual category of woman itself, should be reserved for—as has generally been the case historically—members of the biological *sex* category of femaleness (i.e., females), rather than moving to a newer arrangement based around questions of gender identity or expression (e.g., admitting males who identify as women). Suffice to say this is a complex issue with which societies are current wrestling legally and politically, with authorities having to navigate and resolve various competing claims and agendas. However, there are indications that, after some years of uncertainty and confusion, authorities seem increasingly persuaded of the necessity of preserving exclusively female spaces and categories, at least in some instances. In the sporting arena, for example, the new president of the International Olympic Committee, Kirsty Coventry, addressed the issue at her first official news conference in June 2025, in which she said there is "overwhelming support" by IOC members for keeping competition separated by biological sex, and that "it was very clear from the members that we have to protect the female category, first and foremost to ensure fairness" (Thompson, 2025). Later in November it was subsequently reported (e.g., by Wilson, 2025) that "The Olympics is increasingly likely to ban transgender athletes from all female competition following a science-based review of evidence," and that although no final decision had yet been made, an update to IOC members "reportedly stated that scientific evidence showed there were physical advantages to being born male that remained even after reducing testosterone levels" (with the latter point referencing the fact that the IOC's current policy is that individual sports should decide their own rules, and while sports like athletics and swimming do not allow male transgender athletes into female competition, others like football currently *do* permit that as long as competitors have taken measures to reduce their testosterone levels).

A final complication to mention is that, despite the WHO (2021) stating that sex and gender "are distinct and should not be used interchangeably," they are indeed often treated as synonymous, and it is common for people to use "gender" when they are in fact referring to sex. One reason is that because sex can also refer to the act of procreation, which is often shrouded in cultures of modesty and silence (Fortenberry & Hensel, 2022), and therefore to avoid that kind of connotation, gender has historically

served as an alternative euphemistic label. Indeed, before the 1950s sex and gender *were* generally synonymous; the first recorded use in the OED of "gender" with implications of male/female is dated to 1474 with a reference to "his heirs of the masculine gender" (Debuk, 2016). From the 1950s onwards though, propelled by scholars like John Money (1955), the concepts began to diverge in the ways now articulated by the WHO. Despite such divergence though, in common language, outside the rarified discourse of academia, sex and gender *have* still often functioned synonymously, hence people often using "gender" in place of sex. An Independent Report commissioned by the UK's Department for Science, Innovation and Technology in fact found that these terms have become *increasingly* conflated since the 1990s, including even in official surveys, in ways that are damaging and problematic (Sullivan, 2025). As the report summarizes, "The meaning of sex is no longer stable in administrative or major survey data. This instability is evident across key policy areas including health and justice." Moreover, "In some cases, the conflation of sex and gender identity has been embedded in shared IT systems, posing a barrier to organisations wishing to collect data on sex." These kinds of conflations have led to a "widespread loss of data on sex," which "poses risks to individuals" that are "especially high in the case of minors." Suffice to say this is a highly-charged, complex, and fast-evolving arena of discourse that is of considerable societal consequence, hence the level of attention and disputation in societies around how best to navigate these issues and dynamics.

## References

Alpert, A. B., Ruddick, R., & Manzano, C. (2021). Rethinking sex-assigned-at-birth questions. *BMJ*, n1261. https://doi.org/10.1136/bmj.n1261

Amery, F. (2024). 'Gender critical' feminism as biopolitical project. *Sexualities*. https://doi.org/10.1177/13634607241257397

Debuk. (2016, December 15). A brief history of 'gender.' *Word Press*. https://debuk.wordpress.com/2016/12/15/a-brief-history-of-gender/

Department of Health and Human Services. (2025). Defining Sex: Guidance for Federal Agencies, External Partners, and the Public, Implementing Executive Order 14168, Defending Women from Gender Ideology Extremism and Restoring Biological Truth to the Federal Government. *Department of Health and Human Services*.

Erickson, R. P. (1997). Does sex determination start at conception? *BioEssays*, *19*(11), 1027–1032. https://doi.org/10.1002/bies.950191113

Fausto-Sterling, A. (2000). *Sexing the Body: Gender Politics and the Construction of Sexuality*. Basic Books.

Goymann, W., Brumm, H., & Kappeler, P. M. (2023). Biological sex is binary, even though there is a rainbow of sex roles. *BioEssays*, *45*(2). https://doi.org/10.1002/bies.202200173

Money, J. (1955). Hermaphroditism, gender and precocity in hyperadrenocorticism: Psychologic findings . *Bulletin of the Johns Hopkins Hospital*, *96*(6), 253–264.

Orzack, S. H., Stubblefield, J. W., Akmaev, V. R., Colls, P., Munné, S., Scholl, T., Steinsaltz, D., & Zuckerman, J. E. (2015). The human sex ratio from conception to birth. *Proceedings of the National Academy of Sciences*, *112*(16). https://doi.org/10.1073/pnas.1416546112

Ramsey, J. L., Langlois, J. H., & Marti, N. C. (2005). Infant categorization of faces: Ladies first. *Developmental Review*, *25*(2), 212–246. https://doi.org/10.1016/j.dr.2005.01.001

Rehmann-Sutter, C., Hiort, O., Krämer, U. M., Malich, L., & Spielmann, M. (2023). Is sex still binary? *Medizinische Genetik*, *35*(3), 173–180. https://doi.org/10.1515/medgen-2023-2039

Rosenthal, G. S. (2024, December 3). Are trans women 'biologically male'? The answer is complicated. *The Conversation*. https://theconversation.com/are-trans-women-biologically-male-the-answer-is-complicated-244465

Sax, L. (2002). How common is lntersex? A response to Anne Fausto-Sterling. *The Journal of Sex Research*, *39*(3), 174–178. https://doi.org/10.1080/00224490209552139

Sullivan, A. (2025, March 19). Independent report: Independent review of data, statistics and research on sex and gender. *Department for Science, Innovation & Technology*. https://www.gov.uk/government/publications/independent-review-of-data-statistics-and-research-on-sex-and-gender

Thompson, J. (2025, June 27). New Olympics chief calls for 'protecting' women's category amid global trans athlete wave. *New York Post*. https://nypost.com/2025/06/27/sports/ioc-chief-kirsty-coventry-calls-for-protecting-womens-athletics-during-global-trans-athlete-wave/

Wilson, J. (2025, October 10). Olympics ready to ban transgender athletes from all women's events. *The Telegraph*. https://www.telegraph.co.uk/olympics/2025/11/10/olympics-to-ban-trans-athletes-from-all-womens-events/

World Health Organization. (2021, May 21). Gender and Health. *World Health Organization*. https://www.who.int/news-room/questions-and-answers/item/gender-and-health

Worthen, M. G. F. (2022). This is my TERF! Lesbian Feminists and the Stigmatization of Trans Women. *Sexuality & Culture*, *26*(5), 1782–1803. https://doi.org/10.1007/s12119-022-09970-w
